# Leveraging Noncovalent Interactions for the Binding of CO by a Weakly Lewis Acidic Borane

**DOI:** 10.1002/anie.202501774

**Published:** 2025-04-14

**Authors:** Agamemnon E. Crumpton, Caitilín McManus, Simon Aldridge

**Affiliations:** ^1^ Inorganic Chemistry Laboratory Department of Chemistry University of Oxford South Parks Road Oxford OX1 3QR UK

**Keywords:** Borane, Boron, Carbon monoxide, Frustrated Lewis pair, Quantum chemical calculations

## Abstract

Known boron carbonyl complexes either exploit very high Lewis acidity or a low oxidation state boron centre in order to capture CO. By contrast, we report a carbonyl complex featuring a simple tri‐coordinate borane, characterized by a Lewis acidity which is only marginally higher than B(NMe_2_)_3_. {(Ph_2_P)xanth}_3_B features a solid‐state structure in which two of the three B‐bound xanth(PPh_2_) units are projected above the BC_3_ plane, generating an *up*, *up*, *down* conformation. Quantum chemical methods, however, reveal that the alternative *up*, *up*, *up* alignment, characterized by a cage‐like geometry and enhanced intramolecular noncovalent interactions, is favored significantly in silico (by ca. 33.0 kcal mol^−1^). Although this conformation is optimal for binding polar *C*_3_‐symmetric H‐bond donors such as NH_3_ (and related guests such as H_2_O and MeNH_2_) the binding of essentially nonpolar substrates such as CO would be expected to be weak at best. However, exposure of {(Ph_2_P)xanth}_3_B to CO under mild conditions (1 bar, 25 °C) reversibly yields {(Ph_2_P)xanth}_3_B·CO, a tractable cage‐like borane carbonyl adduct featuring a central BCO moiety shrouded by xanth(PPh_2_) moieties. Dispersion forces are critical to substrate binding: the two binding modes in which the B‐bound CO guest is located inside/outside the host cage differ in energy by 59.5 kcal mol^−1^.

## Graphical abstract

We report a carbonyl complex featuring a simple tri‐coordinate borane, characterized by a Lewis acidity which is only marginally higher than B(NMe_2_)_3_. Exposure of {(Ph_2_P)xanth}_3_B to carbon monoxide under mild conditions (1 bar, 25 °C) reversibly yields {(Ph_2_P)xanth}_3_B·CO. Dispersion forces are critical to substrate binding: the two binding modes in which the B‐bound CO guest is located inside/outside the host cage differ in energy by 59.5 kcal mol^−1^.

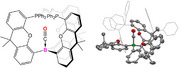

## Introduction

Transition metal complexes are central to widely‐employed catalytic processes, in a large part due to their partially filled *d*‐orbital manifold and facile oxidation state switching.^[^ ^1^ ^]^ The availability of σ and π symmetry frontier orbitals allows for the synergistic binding (and activation) of key ambiphilic small molecule substrates, such as CO, H_2_, and alkenes.^[^ ^2^ ^]^ Although *d*‐block compounds (particularly of the Platinum Group Metals) are therefore integral to current catalytic protocols, issues of scarcity, toxicity and geopolitics have led to the emergence of metal‐free systems as alternative paradigms for small molecule capture and activation.^[^ ^3^, ^4^, ^5^ ^]^ Moulding the electronic structure of such systems to bind molecules such as CO has therefore attracted significant interest.^[^ ^6^, ^7^, ^8^, ^9^, ^10^ ^]^ Within this field, boron carbonyls represent a landmark development, with some such complexes having been shown to mimic the bonding of CO in transition metal complexes.^[^ ^11^, ^12^, ^13^ ^]^

**Figure 1:**
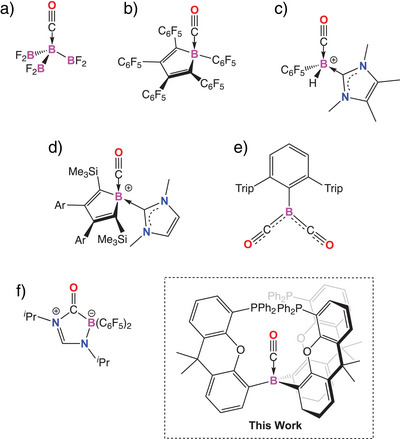
Selected examples of CO complexation by boron‐containing systems.

The weakly‐bound borane‐carbonyl complex, H_3_B·CO has been known since 1937, characterized by its blue‐shifted carbonyl stretching vibration, characteristic of a σ‐dominated bonding interaction.^[^ ^14^, ^15^ ^]^ A handful of other simple borane carbonyl adducts have since been structurally characterized (e.g., Figure 1a–d),^[^ ^16^, ^17^, ^18^, ^19^, ^20^, ^21^, ^22^, ^23^, ^24^, ^25^ ^]^ focusing, at least initially, on the use of electron‐withdrawing perfluoroaryl groups at boron.^[^ ^20^, ^21^ ^]^ Given the primary role of CO in these complexes as a σ‐donor, recent work has focused on enhancing the Lewis acidity of the borane; boro‐cations and anti‐aromatic boroles have been employed to bind gaseous CO (e.g., b–d).^[^ ^22^, ^23^, ^24^, ^25^ ^]^ Interestingly, Sindlinger and coworkers have even reported a cationic borole system (Figure 1d) which gives rise to a red‐shifted carbonyl stretching vibration (with respect to free CO) indicative of back‐bonding from the filled π type orbitals of the borole scaffold.^[^ ^25^ ^]^ Low‐valent boron systems, of course, offer greater possibilities in this regard and the synergistic transition‐metal‐like coordination of carbon monoxide by borylene systems is accompanied by significant activation of the CO molecule (e.g., e).^[^ ^11^, ^12^, ^13^ ^]^

In parallel with these investigations, the emergence of frustrated Lewis pair (FLP) chemistry has led to the discovery of a number of mixed Lewis acid/base systems displaying reactivity toward carbon monoxide (e.g., f).^[^ ^5^, ^26^, ^27^, ^28^, ^29^, ^30^, ^31^, ^32^, ^33^, ^34^, ^35^, ^36^, ^37^, ^38^, ^39^, ^40^, ^41^, ^42^, ^43^, ^44^, ^45^, ^46^, ^47^ ^]^ These FLPs offer the possibility for the synergistic binding of CO involving a donor interaction from the Lewis base component. In this context, we have been interested in using intramolecular P/B FLPs derived from a semi‐flexible xanthene scaffold for small molecule binding.^[^ ^46^, ^48^, ^49^, ^50^ ^]^ In the current study, however, we report a unique mode of stabilization of a boron carbonyl complex using a very weakly Lewis acidic borane, that does not rely on ancillary P‐coordination. Instead, encapsulation of the substrate within a flexible binding pocket augments binding through noncovalent interactions, in a manner reminiscent of biological supramolecular chemistry.

## Results and Discussion

### Synthesis of Cage‐Like Molecular Host

**Scheme 1:**
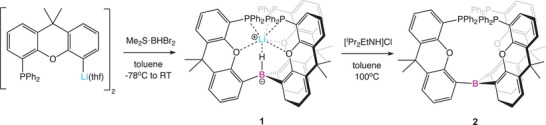
Two‐step synthesis of **2**: Initial reaction of [xanth(PPh_2_)Li(thf)]_2_ with Me_2_S·BHBr_2_ to give intermediate **1**, followed by removal of LiH using [^i^Pr_2_NH(Et)]Cl.

**Figure 2:**
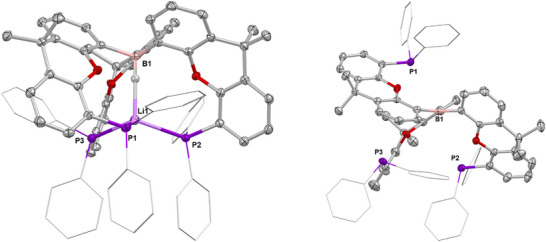
Molecular structures of **1** (left) and **2** (right) in the solid state as determined by X‐ray crystallography. Ellipsoids set to 50% probability, phenyl groups shown in wireframe format and most hydrogen atoms omitted for clarity. Key bond lengths (Å) and angles (°): (for **1**) B–H 1.00(12), Li─H 1.8679(19), P─Li 2.560(2), 2.567(2), and 2.598(2); (for **2**) B─C 1.560(2), 1.572(2), 1.575(2), C─B─C 116.2(1), 118.0(1), and 125.7(1).

{(Ph_2_P)xanth}_3_B (**2**) was synthesized via a two‐step process from [xanth(PPh_2_)Li(thf)]_2_ (Scheme 1).^[^ ^48^, ^49^, ^50^, ^51^, ^52^, ^53^, ^54^, ^55^, ^56^, ^57^, ^58^, ^59^, ^60^ ^]^ The reaction of [xanth(PPh_2_)Li(thf)]_2_ with Me_2_S·BHBr_2_ from −78 °C to room temperature initially gives Li[{(Ph_2_P)xanth}_3_BH] (**1**). **1** features a cage‐like arrangement based on a tetrahedral hydroborate core, with the encapsulated lithium ion coordinated by three phosphine donors (Figure 2). Retention of this structure in benzene‐d_6_ solution is implied by the ^31^P NMR spectrum which shows a 1:1:1:1 quartet at δ_P_ = −17.5 ppm, consistent with a single phosphine environment on the NMR timescale, and with coupling to ^7^Li (*I* = 3/2). In the second step, removal of the encapsulated LiH moiety was achieved through reaction with the weak acid [*^i^* Pr_2_EtNH]Cl at 100 °C in toluene, a process which liberates H_2_ (together with *^i^* Pr_2_EtN and LiCl; Scheme 1). Using this methodology, {(Ph_2_P)xanth}_3_B (**2**) can be isolated in 55–60% overall yield (for the two steps), and crystals suitable for single crystal X‐ray diffraction (SC‐XRD) were obtained from toluene solution at 25 °C.

The solid‐state structure of **2** reveals the expected planar tri‐coordinate boron centre (sum of angles at B(1), 359.9°) with the torsional alignment of the B‐bound aryl groups generating a conformation in which two xanth(PPh_2_) units are projected above the BC_3_ plane, and the third points downward (*up*, *up*, *down* conformation; Figure 2). The ^31^P NMR spectrum reveals a single signal at δ_P_ = −18.3 ppm, implying either rapid fluxional exchange on the NMR timescale at room temperature, or the adoption of a more symmetrical structure in benzene‐d_6_ solution.

DFT analysis (using the R^2^SCAN‐3c method) was employed to investigate the relative energies of different conformations of **2**, in particular the *up*, *up*, *down* form seen in the solid state and an alternative *up, up*, *up* arrangement featuring all three xanth(PPh_2_) units projected in the same direction. These quantum chemical results reveal that the cage‐like *up*, *up*, *up* conformation is favored significantly in silico (by Δ*G* = 33.0 kcal mol^−1^). The energy difference can be attributed primarily to enhanced intramolecular noncovalent interactions (NCI, Figures S55–S58), particularly between the ─PPh_2_ units and the xanthene backbone in the “closed cage” conformation. This large energetic preference implies that **2** is likely to exist in solution exclusively as the *up*, *up*, *up* form, with the alternative structure found in the solid state presumably resulting from enhanced *inter*molecular interactions enabled by the flatter conformation.

### Binding of Protic Guest Molecules

**Figure 3:**
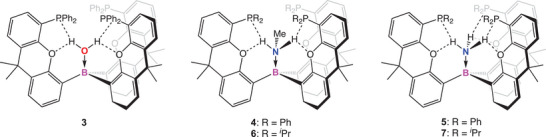
Binding of protic guest molecules by **2** and its ─P *^i^* Pr_2_ analogue.

**Figure 4:**
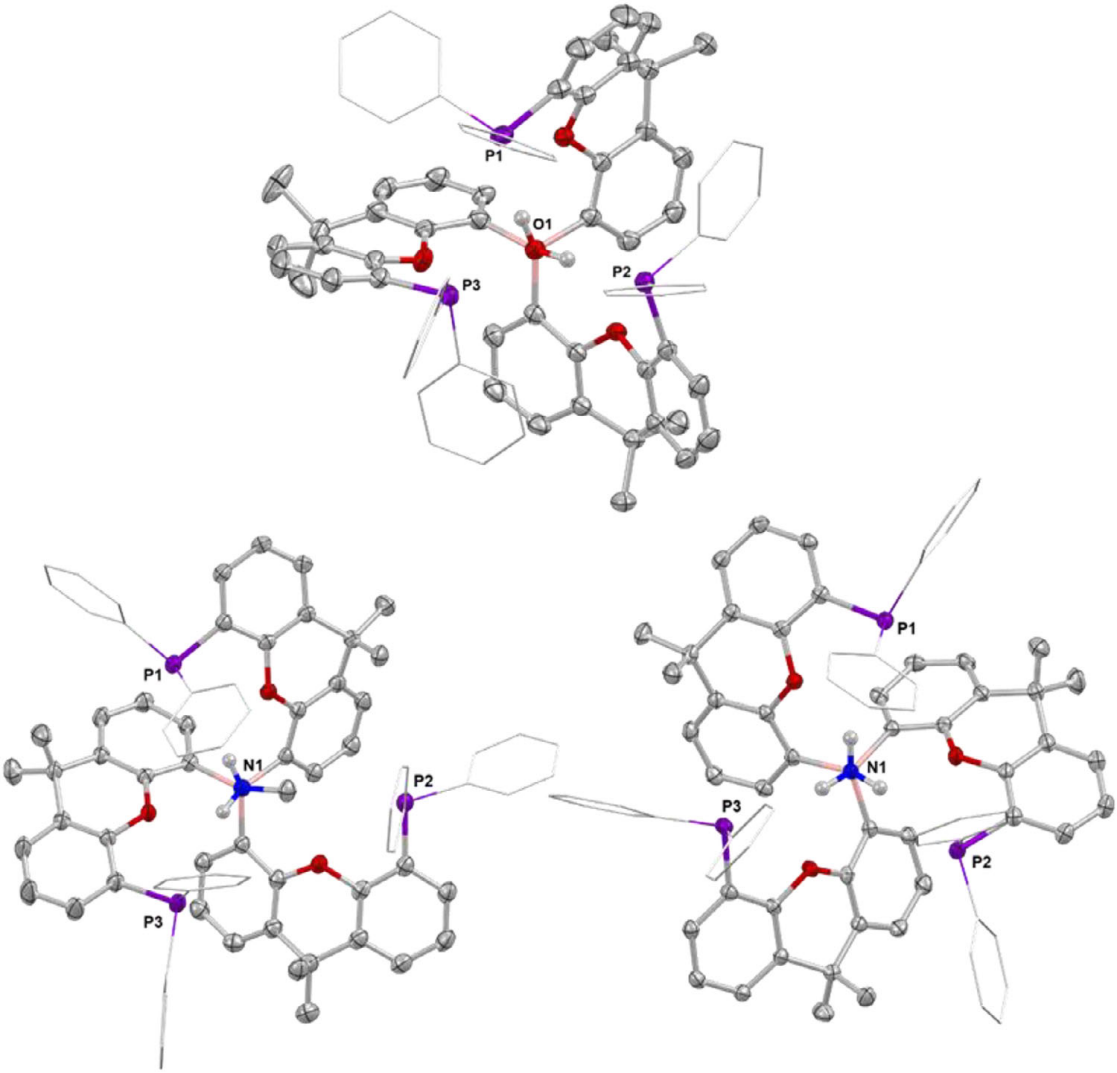
Molecular structures of adducts **3** (upper), **4** (lower left), and **5** (lower right) in the solid state as determined by X‐ray crystallography, with the B─N/O bond aligned toward the viewer. Ellipsoids set to 50% probability, phenyl groups shown in wireframe format and most hydrogen atoms omitted for clarity. Key bond lengths (Å): (for **3**) B─O 1.588(2), P─H 2.31(2), 2.34(2); O─H 2.47(2), 2.58(2); (for **4**) B─N 1.649(2), P─H 2.88(2), 3.09(2); O─H 2.20(3), 2.40(2); (for **5**) B─N 1.627(2), P─H 3.1819(4), 2.6651(5), and 2.7987(5); O─H 2.2949(9), 2.3353(12), and 2.3055(11).

The energetic preference for a cage‐like conformation for **2** and the deployment of the Lewis acid acceptor and three Lewis base donor functions in convergent fashion, suggest that it could be used to trap ambiphilic guest molecules (as in formal LiH host/guest species, **1**). As such, **2** proves to be a highly effective scavenger of trace water, leading to the formation of {(Ph_2_P)xanth}_3_B·OH_2_ (**3**; Figure 3). Bulk preparation of **3** and crystallization from hexanes yields crystals suitable for SC‐XRD (Figure 4). The structure shows a H_2_O unit bound within the cage, with the protons of the water molecule directed toward two of the phosphine units, consistent with the presence of OH···P hydrogen bonding interactions (2.34(2) and 2.31(2) Å). The retention of these interactions in solution is indicated by the downfield shifted nature of the H_2_O ^1^H NMR resonance (*δ*_H_ = 10.69 ppm).^[^ ^51^ ^]^ Given the propensity of **2** to form **3** even with trace amounts of water, a reliable method to remove water was sought. Heating **3** under vacuum for 24 h yielded no change by in situ ^1^H NMR monitoring, but the addition of P_2_O_5_ to a toluene solution of **3** and heating at 80 °C for 48 h gave complete conversion to **2**.

The reactions of **2** with other protic donor molecules were also investigated. **2** reacts immediately with MeNH_2_ to form {(Ph_2_P)xanth}_3_B·NH_2_Me (**4**), characterized by a very similar ^31^P NMR shift to **3** (both −19.6 ppm). SC‐XRD studies indicate a similar overall structure to **3**, albeit featuring a more distorted cage geometry with one xanthene unit rotated to accommodate the bulkier MeNH_2_ guest (∠N–B–(xanthene plane) 161.7(1), 145.7(1), and 146.0(1)°; Figure 4). The NH···P contacts are notably longer (2.88(2) and 3.09(3) Å) than the OH···P interactions in **3**, reflecting both the structural distortion of the cage and the poorer hydrogen bond donor capabilities of MeNH_2_ (over H_2_O). Short NH···O contacts (2.20(3) and 2.40(2) Å) are also observed featuring the ether function of the xanthene units, implying that instead of two strong hydrogen bonds (as in **3**), four weaker interactions are possible in **4**. Informatively, it is found that **3** can readily be converted to **4** via the addition of excess MeNH_2_, revealing that despite the structural distortion and weaker H‐bonds, the overall binding of MeNH_2_ is stronger than that of H_2_O.

Given that the tri‐xanthene cage moiety of **2** features three hydrogen bond acceptors, the binding of NH_3_ was also explored. Exposure of **2** to 1 bar of NH_3_ leads to immediate conversion to {(Ph_2_P)xanth}_3_B·NH_3_ (**5**), as determined by in situ ^31^P NMR spectroscopy (*δ*_P_ = −21.7 ppm). Crystallographic studies reveal the expected binding motif (Figure 4), featuring a relatively short N─B distance (1.627(2) vs. 1.649(2) Å for **4**) and a combination of both NH···P (3.1819(4), 2.6651(5), 2.7987(5) Å), and NH···O interactions (2.2949(9), 2.3353(12), and 2.3055(11) Å); Figure 4). Immediate conversion to **5** was also observed upon addition of NH_3_ to either **3** or **4**, consistent with stronger binding of NH_3_.

**Figure 5:**
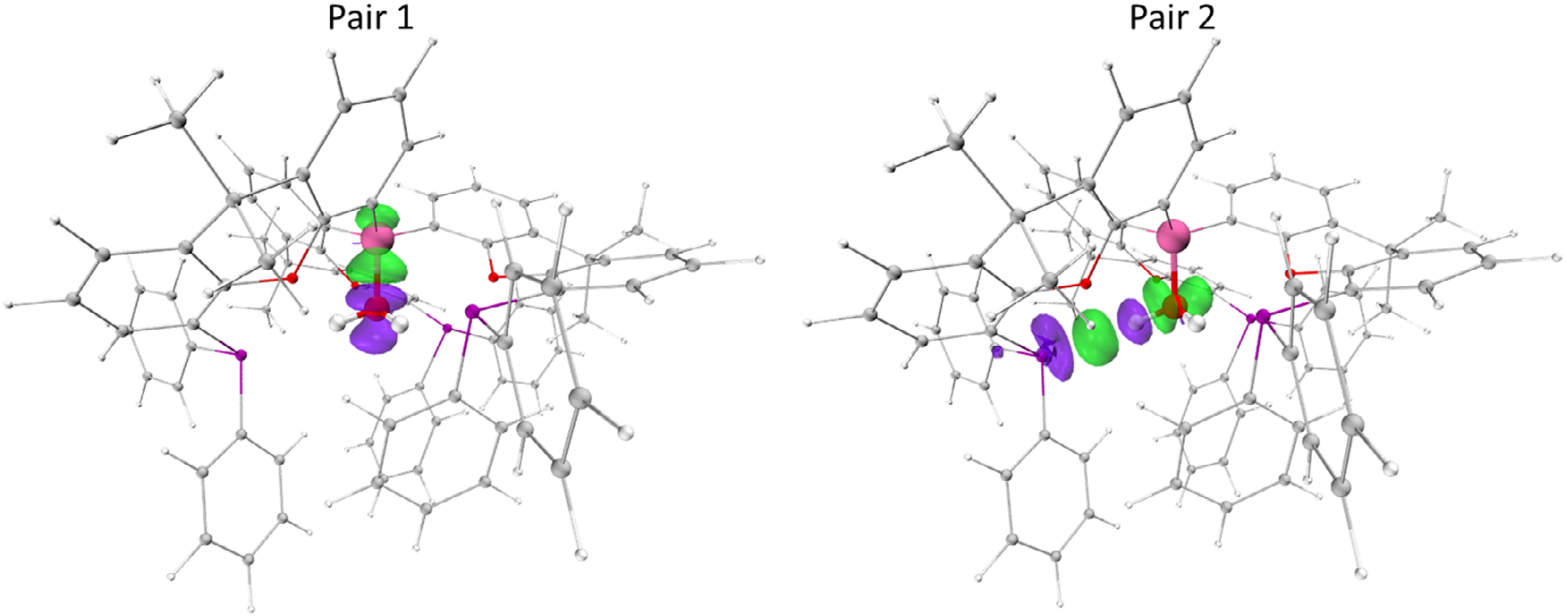
NOCV pair deformation densities for complex **3**, with iso‐surfaces set to 0.005 for pair 1 and 0.002 a.u for pairs 2. Key: green, electron gain; purple, electron depletion.

In order to better understand the binding of protic guest donors by **2**, DFT calculations were carried out and energy decomposition analyses were employed to partition the host–guest interaction. The sobEDA method returns free energies of binding of −10.2, −21.9, and −25.4 kcal mol^−1^ for the H_2_O, MeNH_2_, and NH_3_ adducts **3**–**5**, in accordance with the relative binding strengths determined experimentally. ETS‐NOCV analysis was used to decompose the covalent component into the most significant orbital deformation densities.^[^ ^52^, ^53^, ^54^ ^]^ Intuitively, the primary pair density associated with each system is the donor/acceptor bond between the lone pair at N/O and the vacant orbital at B boron (Figure 5); the more nucleophilic nitrogen donors give rise to significantly stronger donor/acceptor interactions (**3**, −50.3; **4**, −76.0; and **5**, −76.2 kcal mol^−1^).^[^ ^55^ ^]^ Secondary interactions constitute 27%, 22%, and 19% of the total orbital interaction for **3**–**5**, respectively (**3**, −18.7; **4**, −21.8; and **5**, −18.8 kcal mol^−1^). Although each of **3**–**5** displays pair densities associated with charge movement from the protons to the E─B bond, only **3** gives rise to a pair density that could unequivocally be designated as a true hydrogen bond (Figure 5, pair 2).^[^ ^56^ ^]^ Second order perturbation theory within NBO,^[^ ^57^ ^]^ was used to further quantify the magnitude of the host–guest interactions in **3**–**5**. As such, **3** features an OH···P hydrogen bond of 22.5 kcal mol^−1^ with minimal OH···O interactions, while **4** and **5** have much weaker interactions, individually <10 kcal mol^−1^ (**4**, 8.8 kcal mol^−1^; **5**, 7.4 kcal mol^−1^). This finding concurs with experimental observations (from crystallographic and NMR data) that N/OH···P hydrogen bonding is most significant in the case of H_2_O adduct **3**.

### Variation of Cage Substituents

**Figure 6:**
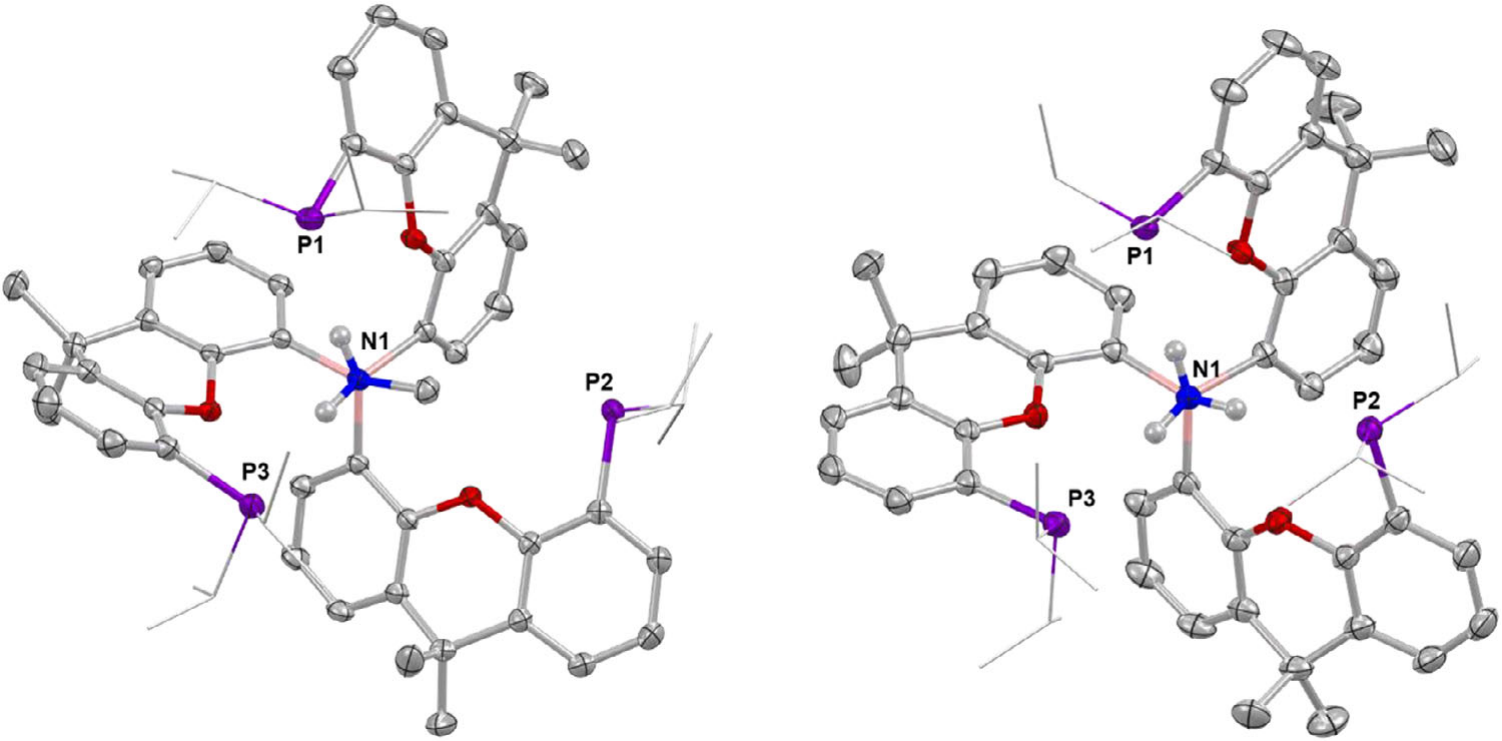
Molecular structures of one of the two independent molecules in the asymmetric unit of **6** (left) and of **7** (right) in the solid state as determined by X‐ray crystallography, with the B─N/O bond aligned toward the viewer. Ellipsoids set to 50% probability, isopropyl groups shown in wireframe format and most hydrogen atoms omitted for clarity. Key bond lengths (Å): (for **6**) B─N 1.622(2), 1.625(2), P─H 2.62(2), 2.62(2), 2.57(2), and 2.57(2); O─H 2.26(2), 2.35(2), 2.37(2), and 2.25(2); (for **7**) B–N 1.609(2), P─H 2.7588(5), 2.6112(5), and 2.5749(7); O─H 2.3154(10), 2.3233(9), and 2.3557(11).

In order to explore potential effects relating to the phosphine substituents, the synthesis of corresponding –P^i^Pr_2_ variant was attempted.^[^ ^48^ ^]^ A synthetic route analogous to that used to synthesize **1** resulted in a complex reaction mixture that resisted further purification. An alternative procedure, involving the reaction of [xanth(P*^i^* Pr_2_)Li]_4_ with BBr_3_ also generated a mixture of products; in this case, however, addition of MeNH_2_ led to the formation of a single product {(^i^Pr_2_P)xanth}_3_B·NH_2_Me (**6**), which allows for direct comparison with its –PPh_2_ counterpart. Crystals suitable for SC‐XRD were obtained from benzene at 25 °C and the structure was found to be analogous to **4**, albeit with two units in the asymmetric unit (Figure 6).

While the B─N separation is slightly shorter for **6** than for **4** (1.622(2)/1.625(2) vs. 1.649(2) Å), the NH···P distances are markedly shorter for the ─P ^i^Pr_2_ variant (2.57(2), 2.57(2), 2.62(2), and 2.62(2) Å), as predicted by increased phosphine basicity.^[^ ^58^ ^]^ Shorter NH···O interactions are also seen (2.25(2), 2.37(2), 2.26(2), and 2.35(2) Å), although whether this is due to the tighter geometry afforded by the shorter NH···P distances or vice versa is difficult to deconvolute. The MeNH_2_ guest is less labile within **6** than **4**: no reaction is observed on addition of NH_3_ to **6** at 25 °C, while heating at 80 °C for 72 h leads only to 40% MeNH_2_/NH_3_ substitution. Despite this, single crystals of the ammonia adduct {(^i^Pr_2_P)xanth}_3_B·NH_3_ (**7**), could be isolated and the geometry of the product confirmed by SC‐XRD (Figure 6). Here too, shorter NH···P distances are observed (2.7588(5), 2.6112(5), and 2.5749(7) Å) compared to the ─PPh_2_ analogue **5**, consistent with stronger hydrogen bonding interactions.

The poorer conversion of **6** into **7** (cf. **4** into **5**) is attributed to tighter binding of MeNH_2_, facilitated by stronger hydrogen bonding interactions, which offer an increased kinetic barrier to the exchange process. EDA‐NOCV analyses reveal N─B donor/acceptor pairs which amount to −79.6 kcal mol^−1^ for compound **6** and 80.8 kcal mol^−1^ for compound **7**, i.e., values which are very similar to those observed for **4** (−76.0 kcal mol^−1^) and **5** (−76.2 kcal mol^−1^). On the other hand, the strengths of the respective hydrogen bonds, calculated using second‐order perturbation theory, are significantly enhanced for compounds **6** (18.8 kcal mol^−1^) and **7** (18.6 kcal mol^−1^) (*cf*. 8.8 and 7.4 kcal mol^−1^ for **4** and **5**), corroborating the shorter NH···P distances determined through crystallographically.

### Binding of Carbon Monoxide and Related Molecules

Although the combination of a Lewis acid and three Lewis basic sites within **2** lends itself to binding substrates such as NH_3_, the binding of weakly polar nonprotic guests such as CO would be expected to be precluded on the basis of an acceptor number of 13.6 (determined by the Gutmann–Beckett method),^[^ ^59^ ^]^ which is only marginally higher than those reported for B(NMe_2_)_3_ (9.1) and B(O*^t^* Bu)_3_ (11.8).^[^ ^60^ ^]^ Fluoride ion affinities (FIAs, Table S8) calculated for **2** tell a similar story, with the values obtained (−82.1 kcal mol^−1^ (inside the cavity); −63.6 kcal mol^−1^ (outside)) also reflecting somewhat higher Lewis acidity than that predicted for B(NMe_2_)_3_ (−36.7 kcal mol^−1^).

**Figure 7:**
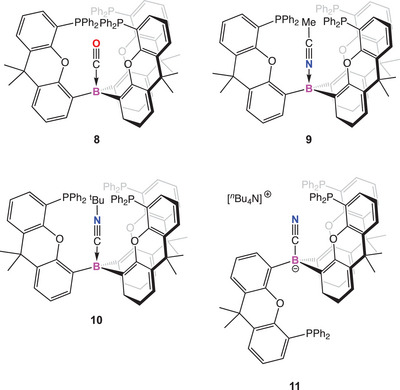
Adducts of **2** formed with CO (**8**), MeCN (**9**), *^t^* BuNC (**10**), and CN^−^ (**11**).

**Figure 8:**
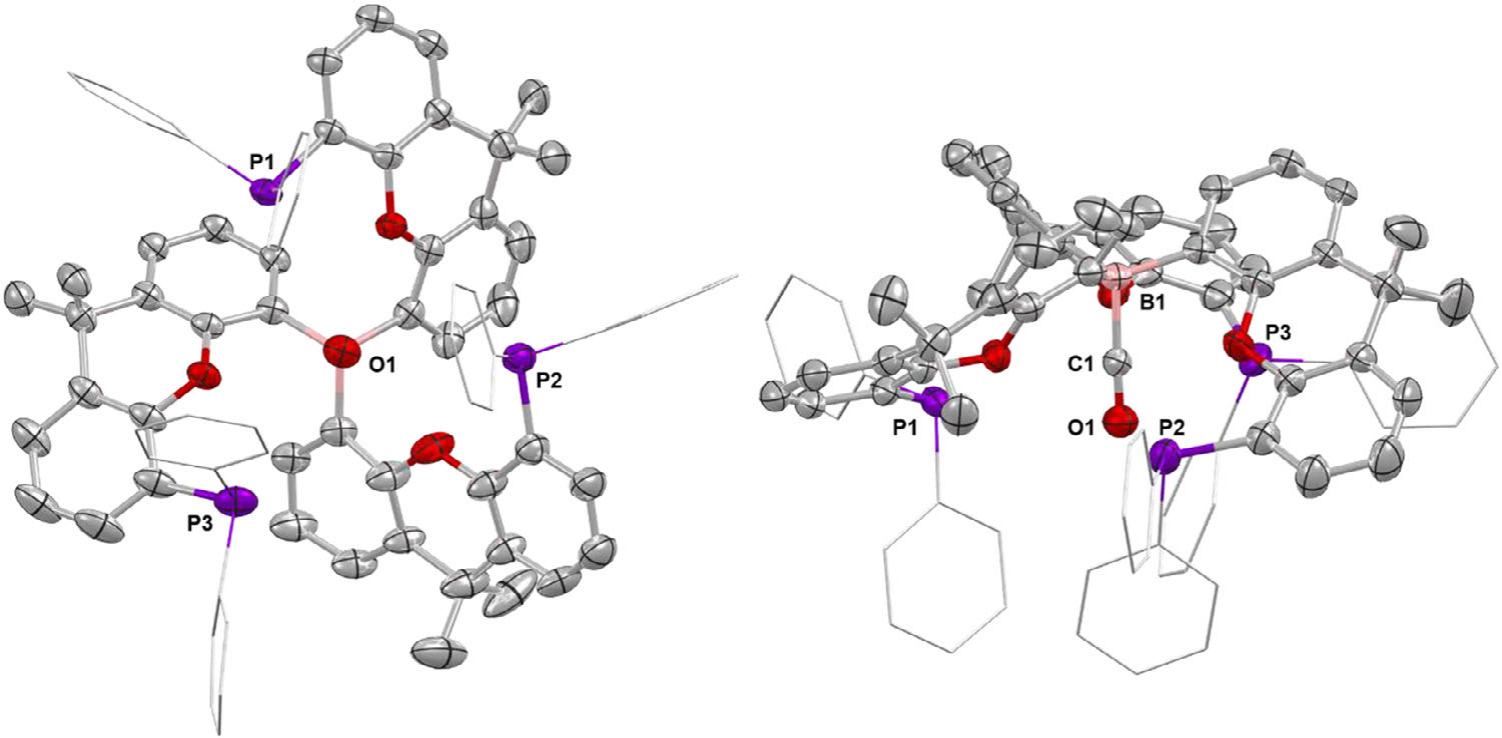
Molecular structure of one of the two independent molecules in the asymmetric unit of **8** in the solid state as determined by X‐ray crystallography (top and side views). Ellipsoids set to 50% probability, isopropyl groups shown in wireframe format and most hydrogen atoms omitted for clarity. Key bond lengths (Å) and angles (°): B─C(O) 1.619(3), 1.619(3), C─O 1.117(3), 1.122(3), P─C(O) 3.564(2), 3.629(2), 3.715(2), 3.551(2), 3.717(2), and 3.841(2), O─C(O) 2.699(2), 2.818(2), 2.711(3), 2.770(2), 2.754(2), and 2.739(2), B─C─O 177.7(2), 174.4(2).

Exposure of **2** to CO (1 bar) at 25 °C in *ortho*‐difluorobenzene, however, yields colorless crystals, which can be shown by SC‐XRD to contain {(Ph_2_P)xanth}_3_B·CO (**8**; Figure 7), a borane carbonyl adduct featuring a central BCO moiety shrouded by xanthene phosphine moieties (Figure 8). The solid‐state structure features two molecules in the asymmetric unit, each featuring a near linear B─C─O unit (177.7(2)° and 174.7(2)°), and the B─C bond lengths (both 1.619(3) Å) fall within the range of previous borane–carbon monoxide adducts.^[^ ^14^, ^16^, ^17^, ^18^, ^19^, ^20^, ^21^, ^22^, ^23^, ^24^, ^25^ ^]^

The phosphine donors are remote from the carbon atom of the CO guest, with separations of > 3.5 Å (3.551(2)–3.841(2) Å), i.e., over double the sum of the respective covalent radii (1.73 Å) and even in excess of the sum of the van der Waals radii (3.5 Å).^[^ ^61^ ^]^ In addition, the orientations of the phosphine groups suggest that the lone pairs are directed toward the xanthene backbone, rather than the CO guest; these metrical data (together with a ^31^P NMR shift close to that of **2** (−21.8 ppm)) effectively precludes the possibility of an FLP mode of CO binding. The capture of CO between the donor and acceptor units, as seen with other FLP systems, is typically accompanied by a large downfield shift in the ^31^P resonance.

**Figure 9:**
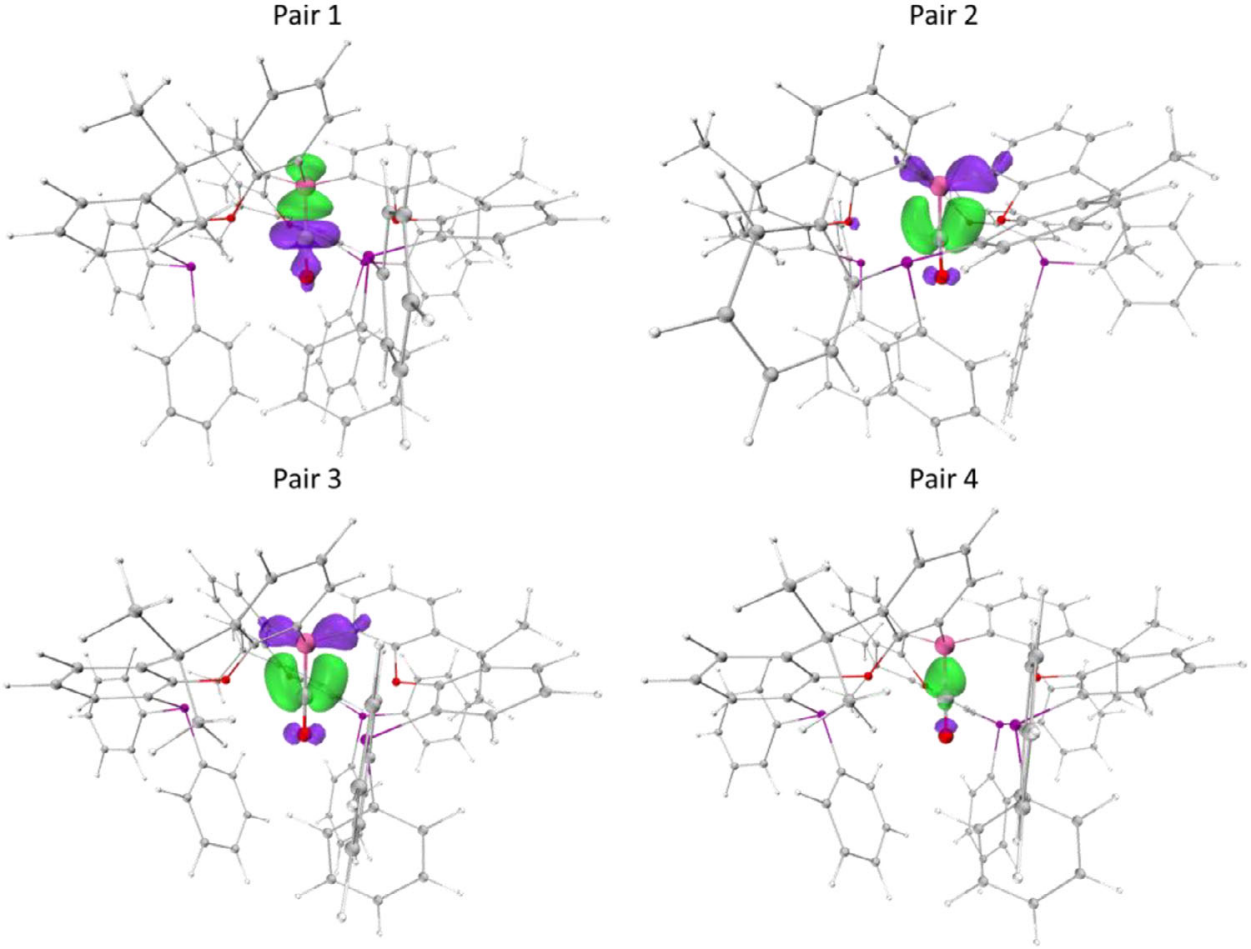
NOCV pair deformation densities for carbonyl complex **8**, iso‐surface set to 0.005 for pair 1 and 0.002 a.u for pairs 2–4. Key: green, electron gain; purple, electron depletion.

The C─O bond lengths measured for **8** (1.117(3), 1.122(3) Å) are not statistically different from that of free CO (1.1281 Å),^[^ ^62^ ^]^ and the IR stretching frequency (2179 cm^−1^) falls within the range previously reported for borane‐CO complexes.^[^ ^14^, ^16^, ^17^, ^18^, ^19^, ^20^, ^21^, ^22^, ^23^, ^24^, ^25^ ^]^ As such, it is indicative of σ‐dominated bonding with minimal back‐bonding, similar to that of so‐called “non‐classical” transition metal complexes.^[^ ^15^ ^]^ This is further corroborated by the ^13^C NMR resonance (171.7 ppm), which is shifted upfield compared to that of free CO (184.1 ppm). EDA‐NOCV analysis was carried out to quantify both bonding and back‐bonding interactions. Consistently, the OC─B σ bond accounts for 74.2% of the total orbital interaction energy with back‐bonding accounting for only 16.5%; back‐bonding occurs to both CO π* orbitals with electron density being transferred from the B─C σ bonds (Figure 9).

The reaction of **8** with CO is reversible: **2** is regenerated on removal of the CO atmosphere. In solution the release of CO can be driven at elevated temperatures, and VT‐NMR measurements allow Δ*H* and Δ*S* to be determined from a van't Hoff analysis. The values obtained (Δ*H* = −10.9 kcal mol^−1^; Δ*S* = 3.2 cal mol^−1^ K^−1^) for CO release, are consistent with the corresponding DFT‐calculated values (assuming a closed cage *up*, *up*, *up* conformation for **2**): Δ*H*_calc_ = −16.2 kcal mol^−1^; Δ*S*_calc_ = 3.3 cal mol^−1^ K^−1^.

**Figure 10:**
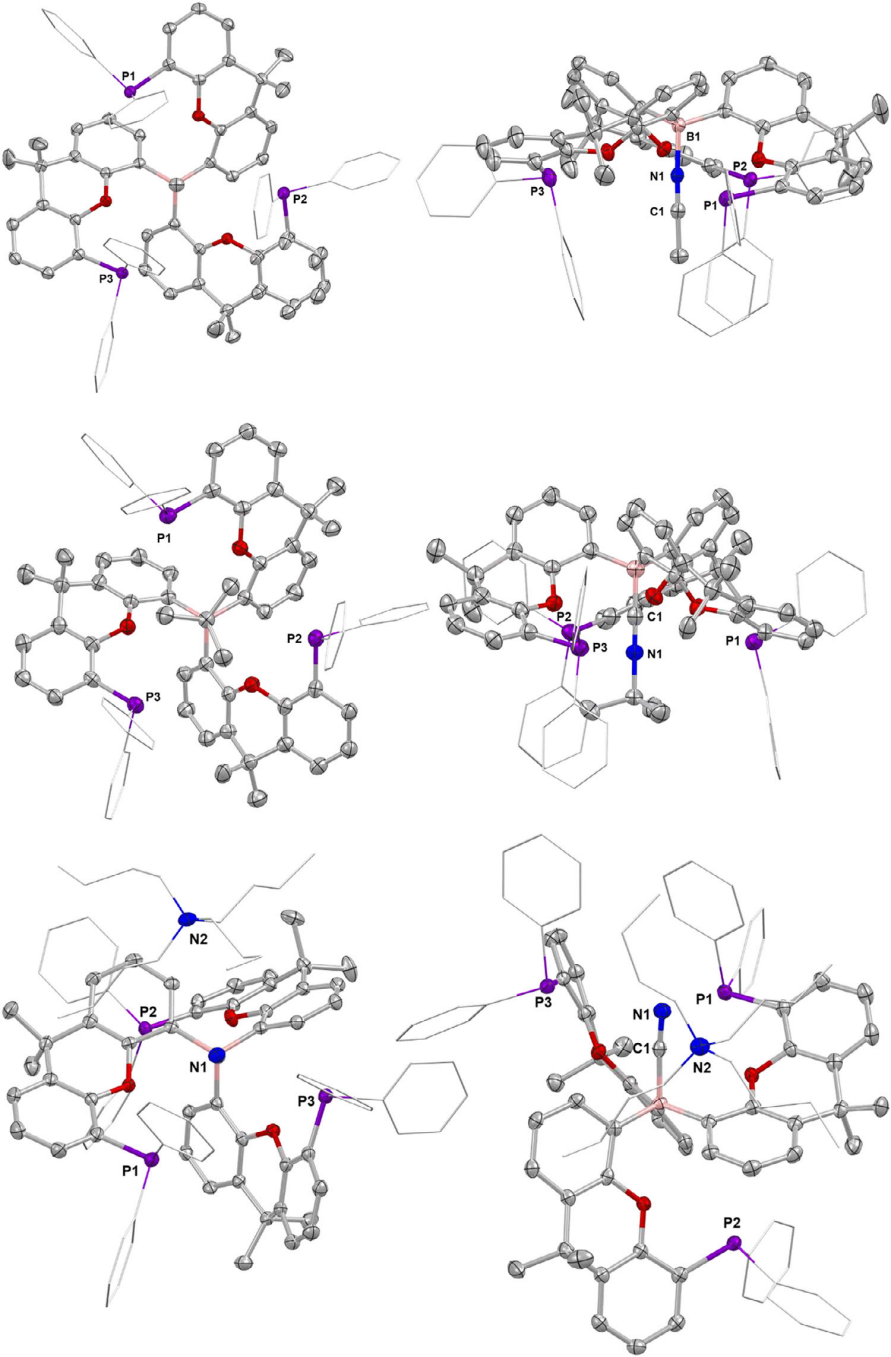
Molecular structure of **9** (upper), one of the two independent molecules in the asymmetric unit of **10** (centre) and **11** (lower) in the solid state as determined by X‐ray crystallography (top and side views of each). Ellipsoids set to 50% probability, isopropyl groups shown in wireframe format and most hydrogen atoms omitted for clarity. Key bond lengths (Å) and angles (°): (for **9**) B─N 1.591(3), C─N 1.138(2), and P─N 3.943(1), 4.032(1), and 4.081(1), O─N 2.828(2), 2.834(2), and 2.945(2), B─N─C 176.5(2), N─C─C 179.2(2); (for **10**) B─C(N) 1.619(12), 1.523(12), C─N 1.094(12), 1.194(10), P─C(N) 3.947(2), 3.966(2), O─C(N) 2.804(4), 2.832(3), B─C─N 180.0, 180.0, C─N─C 180.0, 180.0; (for **11**) B─C(N) 1.625(4), C─N 1.148(3), P─C(N) 3.733(3), 3.777(2), O─C(N) 2.908(3), 3.049(3), B─C─N 173.4(3).

The capture of a range of related guest molecules/anions by **2** was also investigated, including *^t^* BuNC, MeCN, and CN^−^ (as the [*^n^* Bu_4_N]^+^ salt) (Figure 7). **2** takes up acetonitrile in benzene leading to complete conversion to {(Ph_2_P)xanth}_3_B·NCMe (**9**); as with CO, complete removal of the guest is observed under continuous vacuum. The structure of **9** in the solid state contains a single molecule in the asymmetric unit featuring a linear B−N−C−C unit (Figure 10), and long P−N distances (ca. 4 Å). The CN stretching vibration (at 2356 cm^−1^) is significantly higher than that of uncoordinated acetonitrile (2253 cm^−1^),^[^ ^63^ ^]^ implying that bonding is dominated by σ donation and EDA‐NOCV calculations confirm that the total orbital interaction energy is dominated by σ donation (71.0%).

With the more sterically demanding *tert*‐butyl isocyanide, host–guest complex formation is less facile: heating to 80 °C for 18 h is required for full conversion to {(Ph_2_P)xanth}_3_B·CN^t^Bu (**10**). The binding of *^t^* BuNC also appears to be less reversible than with CO or MeCN, with no release of the guest molecule observed under continuous vacuum. The structure of **10** determined by SC‐XRD features a crystallographically imposed linear B─C─N─C unit (Figure 10), and wide P─C separations (3.947(2), 3.966(2), Å). The CN stretching frequency at 2285 cm^−1^ (as measured by IR spectroscopy) is also higher than that of free *^t^* BuNC (2140 cm^−1^),^[^ ^18^ ^]^ indicating that here too the bonding is predominantly driven by σ interactions. EDA‐NOCV confirms that **10** features the smallest proportion of back bonding, 10.2% (77% σ donation), implying that *tert*‐butyl isocyanide is a weaker π acceptor in this context than acetonitrile and CO).^[^ ^64^ ^]^ Assimilation of the cyanide anion by **2** also requires more forcing conditions: reaction with tetrabutylammonium cyanide in benzene requires heating at 80 °C for 18 h to generate [*^n^* Bu_4_N][{(Ph_2_P)xanth}_3_B·CN] (**11**). The structure of **11** in solution is much less symmetrical than the other adducts, as indicated by ^1^H (three xanthene backbone methyl group signals) and ^31^P NMR spectroscopy (three phosphine resonances at −18.0, −20.2, and −23.7 ppm). Consistently, SC‐XRD reveals not the cage geometry seen for all other host–guest species, but an adduct based on the alternative *up*, *up*, *down* conformer of the host (Figure 10).

The ^11^B NMR chemical shifts of the C‐donor adducts **8**, **10,** and **11** fall within a relatively narrow range (−14.8 (CO), −16.2 (CN*^t^* Bu), and −13.6 ppm (CN^−^)), upfield of N‐donor complexes **4**, **5,** and **9** (0.3 (NH_2_Me), −4.7 (NH_3_) and −5.3 ppm (MeCN)) and similar to that of hydride adduct **1** (−16.7 ppm), consistent with the expected trend in ligand σ‐donor strength.

### Quantum Chemical Analysis of CO Binding

**Figure 11:**
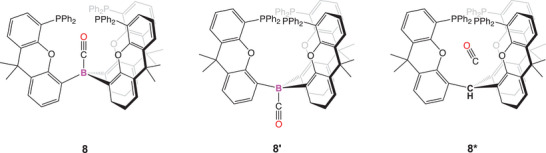
Binding of CO inside the cage (**8**, left), binding outside the cage (**8′**, middle) and as a host–guest interaction with the modified cage featuring a CH unit instead of B (8*, right).

Previous examples of boron‐centred carbonyl complexes have leveraged very high Lewis acidity, a low oxidation state boron centre or extensive ligand back‐donation in order to facilitate binding of CO.^[^ ^11^, ^12^, ^13^, ^14^, ^16^, ^17^, ^18^, ^19^, ^20^, ^21^, ^22^, ^23^, ^24^, ^25^ ^]^ **2** demonstrates none of these features – it is a very modest Lewis acid offering a small degree of ligand back‐donation. Hypothesizing that stabilization in this case might be afforded by secondary interactions with the shrouding xanthene groups, a series of calculations was carried on **8** and related adducts. A combination of geometric factors (orientation of the P‐ and O‐donors; P─C and O─C distances) in both the solid state and calculated structures of **8** and second order perturbation theory/NOCV analyses rule out significant P‐ or O‐donation into the CO π* orbital, i.e., binding via an FLP‐type mechanism. As such, the potential role(s) of noncovalent interactions in stabilizing host–guest complexes were probed. This was achieved by consideration of **8** against a model system in which the CO guest is bound outside the cage (**8′**; Figure 11). Binding is only thermodynamically viable within the host cage, and the value of ΔΔ*G* (−59.5 kcal mol^−1^;Table 1) implies that the stabilization of the host–guest complex from noncovalent interactions within the host cavity is very significant. To further probe the differences in the binding inside and outside the cage energy decomposition analysis was performed using SobEDA method (Table 1).^[^ ^65^ ^]^

**Table 1:** Decomposition of the host–guest interactions in **8** and **8′** using the sobEDA approach.^[^ ^65^ ^]^

	8	8′	Δ^b)^
Gibbs free energy of binding Δ*G*_B_ ^a)^	−6.1	53.4	−59.5
Total interaction energy (Δ*E*_int_)	−34.2	−4.7	−29.5
Electrostatic (Δ*E*_elst_)	−76.4	−66.6	−9.8
Exchange‐repulsion (Δ*E*_xrep_)	211.3	237.5	−26.2
Orbital (Δ*E*_orb_)	−115.1	−113.0	−2.1
Coulomb correlation (Δ*E*_c_)	−54.0	−62.5	8.5
DFT correlation (Δ*E*_DFTc_)	−52.9	−61.9	9.0
Dispersion correction (Δ*E*_dc_)	−1.1	−0.6	−0.5

^a)^ Energies given in kcal mol^−1^.

^b)^ Defined as Δ*E* for compound **8** minus Δ*E* for compound **8′**.

**Figure 12:**
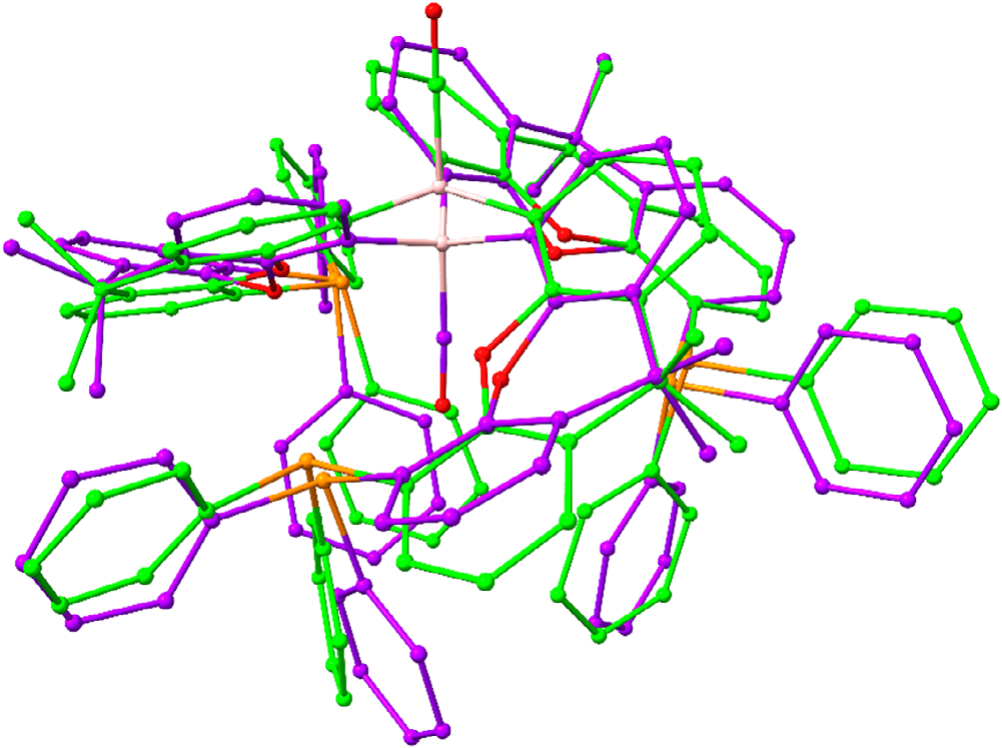
Geometries of optimized structures **8** (purple) and **8′** (green) overlapped to demonstrate the effects on the cage architecture on pyramidalization of the boron centre.

The SobEDA approach^[^ ^65^ ^]^ decomposes the interaction energy between fragments (in this case the substrate/guest and cage/host) into electrostatic (Δ*E*_elst_), exchange repulsion (Δ*E*_xrep_), orbital interaction (Δ*E*_orb_), and Coulomb correlation terms (Δ*E*_c_), such that Δ*E*_int_ = Δ*E*_elst_ + Δ*E*_xrep_ + Δ*E*_orb_ + Δ*E*_c_. The total interaction energy between the cage host and CO guest fragments (in their geometries as found in the host–guest complex) is 29.5 kcal mol^−1^ less favorable for **8′** when compared to **8** (Table 1). This corresponds to a moderate decrease in the attractive Δ*E*_elst_ (ΔΔ*E*_elst_ = 9.7 kcal mol^−1^) when comparing **8′** to **8**, that can be attributed to the loss of interactions between the (weakly) polar CO molecule with the electron rich and deficient regions within cage. The repulsive Δ*E*_xrep_ term increases for **8′** compared to **8**, which can be rationalized by pyramidalization at boron which pushes the xanthene groups together when the guest molecule is bound *outside* the cage, increasing the degree of steric repulsion. By contrast, when binding occurs within the cage the xanthene groups are pushed apart effectively opening the cage, and reducing the steric clash (Figure 12).

The attractive Δ*E*_orb_ term stays approximately the same (between **8′** and **8**), as might be expected given that this term is dependent on orbital overlap, which is more‐or‐less consistent between binding inside or outside the cage. The Coulomb correlation term (Δ*E*_c_) can be can be decomposed further into dispersion (Δ*E*_dc_) and DFT correlation (Δ*E*_DFTc_) terms. For **8**, the dispersion term is stronger than for molecule **8′**, indicating greater attractive interactions due to London dispersion forces within the cage framework. Conversely, the Δ*E*_DFTc_ term, associated with charge redistribution effects, becomes less negative (less stabilizing) when binding occurs inside the cage. However, interpreting the overall chemical meaning of Δ*E*_c_ is challenging because it reflects a mix of short‐range (electronic repulsion) and medium‐range (dispersion) interactions, which are inherent limitations of the employed DFT functional.^[^ ^65^ ^]^

As an alternative approach, the effects of secondary interactions on the stabilization of the host–guest complex were probed by examining the CH (for boron) substituted system 8* (Figure 11), in which the CO guest is bound solely by noncovalent interactions. Incorporation of CO within this system is associated with a slightly exothermic value of Δ*H* (−1.1 kcal mol^−1^). 8* features no direct donor/acceptor bond between the host and guest, which allows for repartitioning of the chemically nonintuitive Δ*E*_DFTc_ term in the modified SobEDAw method.^[^ ^65^ ^]^ In SobEDAw Δ*E*_DFTc_ is redistributed into the Δ*E*_xrep_ and the Δ*E*_dc_ terms giving a dispersion term which is more comparable to that calculated by symmetry‐adapted perturbation theory (SAPT); the interaction energy is reformulated instead as Δ*E*_int_ = Δ*E*_elst_ + Δ*E*_xrep_ + Δ*E*_orb_ + Δ*E*_dc_. This approach delivers a value for Δ*E*_int_ of −8.5 kcal mol^−1^, with the dominating attractive interaction being the dispersion contribution (ΔE_dc_ = −19.8 kcal mol^−1^). As such, the importance of dispersion interactions in host binding within the cage architecture of systems of this type is clearly demonstrated.

## Conclusions

{(Ph_2_P)xanth}_3_B (**2**) has been synthesized in good yield (55%–60%) via a two‐step process from [xanth(PPh_2_)Li(thf)]_2_, and features a solid‐state structure in which two of the three B‐bound xanth(PPh_2_) units are projected above the BC_3_ plane, generating an *up*, *up*, *down* conformation. Quantum chemical calculations, however, reveal that the alternative *up*, *up*, *up* cage‐like geometry is favored significantly in silico (by ca. 33.0 kcal mol^−1^) due to enhanced intramolecular noncovalent interactions. The combination of a Lewis acid and three Lewis basic sites within the {(Ph_2_P)xanth}_3_B cage enables strong binding of polar protic guests in solution through a combination of donor/acceptor and hydrogen bonding interactions. A binding hierarchy H_2_O < MeNH_2_ < NH_3_ is established, reflecting the interplay between the Lewis base donor strength and the complementarity of the H‐bond architecture.

By contrast, the assimilation by **2** of weakly polar nonprotic guests such as CO would be expected to be precluded on the basis of an acceptor number determined by the Gutmann–Beckett method (13.6) similar to that of B(NMe_2_)_3_. Exposure of **2** to CO under ambient conditions (1 bar, 25 °C) however, reversibly yields {(Ph_2_P)xanth}_3_B·CO (**8**), a borane carbonyl adduct featuring a central BCO moiety shrouded by xanth(PPh_2_) moieties. Geometric factors (notably the orientation of the P‐ and O‐donors and the P···C and O···C distances) in both the solid state and calculated structures effectively rule out significant P‐ or O‐donation into the CO π* orbital (i.e., binding via an FLP‐type mechanism). Structural and spectroscopic properties of **8** (a C─O bond length not statistically different from that of free CO, a blue shifted carbonyl stretching frequency and an upfield shifted carbonyl ^13^C NMR resonance) imply that σ‐donation from CO to boron dominates the OC─B interaction. This hypothesis is supported by EDA‐NOCV analysis which shows that the OC─B σ bond accounts for 74.2% of the total orbital interaction energy.

Structural characterization of a series of related host–guest complexes featuring B‐bound MeCN, *^t^* BuNC, or CN^−^ guests showcases the ability of {(Ph_2_P)xanth}_3_B to encapsulate substrates by modulating the shape and size of the pocket to the guest. Consistently, we find that dispersion forces are critical to substrate binding, especially in the context of an overall free energy of binding for {(Ph_2_P)xanth}_3_B·CO which is marginally exergonic at room temperature (Δ*G*_298_ = −9.30 kcal mol^−1^ (expt), −5.76 kcal mol^−1^ (calc)). Alternative binding modes in which the B‐bound CO guest is located either inside or outside the host cage differ in energy by 59.5 kcal mol^−1^.

## Experimental

Synthetic and characterizing data for key compounds, together with representative spectra and details of crystallographic and quantum chemical studies are included in the Supporting Information.^[^ ^66^ ^]^

## Conflict of Interests

The authors declare no conflict of interest.

## Supporting information



Supporting Information

Supporting Information

## Data Availability

The data that support the findings of this study are available in the supplementary material of this article.
