# Supplementary material for: Leveraging Noncovalent Interactions for the Binding of CO by a Weakly Lewis Acidic Borane
Source: Angew Chem Int Ed Engl. 2025 Apr 14;64(22):e202501774. doi: 10.1002/anie.202501774 (PMC12105714; doi:10.1002/anie.202501774)
Supplement: Supplementary file 1 — Supporting Information [file ANIE-64-e202501774-s001.pdf]

Supplementary Materials

Contents

Experimental methods....2

General Considerations....2

Synthesis of novel compounds....2

1 - Li[{(Ph_2P)xanth}$_3$BH]....2

2 - {(Ph_2P)xanth}$_3$B....2

3 - {(Ph_2P)xanth}$_3$B·OH_2....3

4 - {(Ph_2P)xanth}$_3$B·NH_2Me....3

5 - {(Ph_2P)xanth}$_3$B·NH_3....4

8 - {(Ph_2P)xanth}$_3$B·CO....4

9 - {(Ph_2P)xanth}$_3$B·NCMe....4

10 - {(Ph_2P)xanth}$_3$B·CN^{t}Bu....5

11 - [^{n}Bu$_4$N][{(Ph_2P)xanth}$_3$B·CN]....5

6 - {(^{i}Pr$_2$P)xanth}$_3$B·NH_2Me....6

7 - {(^{i}Pr$_2$P)xanth}$_3$B·NH_3....6

Spectra of new compounds....7

NMR....7

NMR studies of CO uptake by **2**....25

VT NMR of CO uptake by **2**....28

IR spectroscopy....29

Crystallographic Data....30

Methods....30

Table S1: X-ray crystallography....32

DFT....33

General procedure....33

Weak bonding analysis....33

Energy Decomposition analysis....39

Xyz coordinates....49

References....74

Experimental methods

General Considerations

All manipulations were carried out using Schlenk line or glovebox techniques under an atmosphere of argon or dinitrogen. Solvents were dried by passage through activated alumina columns, degassed before use and stored over potassium. NMR spectra were measured in C_6D_6 or C_7D_8 (d_8-toluene) which were dried over 3 Å molecular sieves, with the solvent stored under nitrogen in a Teflon valve ampoule. CO was stored in a Teflon valve ampoule over P_2O_5. NMR samples were prepared under nitrogen in 5 mm Wilmad 507-PP tubes fitted with J. Young Teflon NMR spectra were measured on a Bruker Avance III HD Nanobay 400 MHz NMR spectrometer equipped with a 9.4 T magnet or a Bruker Avance III NMR 500 MHz NMR spectrometer. ^{1}H and ^{13}C NMR spectra were referenced internally to residual protio-solvent (^{1}H) or solvent (^{13}C) resonances and are reported relative to tetramethylsilane (δ = 0 ppm). Variable temperature ^{31}P spectra were referenced relative to a standard of (Ph_2P)xanthH in C_7D_8. Chemical shifts are quoted in δ (ppm) and coupling constants in Hz. FTIR spectra were measured on a Bruker Alpha spectrometer with Platinum-ATR module. Elemental analyses were carried out by London Metropolitan University; measurements on phosphorus-containing compounds can be subject to artificial lowering of the % carbon.[1] [xanth(PPh_2)Li(thf)]$_2$ and [xanth($P^{i}Pr_2$)Li]$_4$ were prepared according to literature procedures.[2] [$^{i}Pr_2$EtNH]Cl was synthesized via a modified literature procedure using dry HCl in Et_2O.[3]

Synthesis of novel compounds

1 - Li[{(Ph_2P)xanth}$_3$BH]

Toluene (50 mL) was added dropwise to [xanth(PPh_2)Li(thf)]$_2$ (500 mg, 1.25 mmol of monomer) and dibromoborane dimethyl sulfide complex (300 mg, 1.28 mmol) added at -78 °C under vigorous stirring. The resulting solution was stirred at -78°C for 30 min and the reaction mixture then warmed to room temperature and stirred for a further 16 h. The resulting cloudy solution was then heated to 100°C and filtered via canula where the resulting solution was concentrated to the point of incipient crystallisation. storage of this solution overnight at -30°C resulted in very large cubic crystals which were washed with Et_2O. Yield: 456 mg (91 %) crystals suitable for X-ray diffraction could be obtained by recrystallisation from toluene at room temperature. **Spectroscopic data: ^{1}H** NMR (500 MHz, C_6D_6) δ 7.26 – 7.18 (m, 9H, ArH), 7.06 (t, J = 7.5 Hz, 3H, ArH), 6.90 (t, J = 7.2 Hz, 12H, ArH), 6.82 (t, J = 7.5 Hz, 6H, ArH), 6.79 – 6.67 (m, 12H, ArH), 6.55 (t, J = 7.6 Hz, 6H, ArH), 4.42 – 2.95 (m, 1H, BH), 1.60 (s, 9H, Xan-CH_3), 1.39 (s, 9H, Xan-CH_3). **^{7}Li** NMR (156 MHz, C_6D_6) δ -1.1. **^{11}B** NMR (160 MHz, C_6D_6) δ -16.7 (d, J = 70.4 Hz). **^{13}C** NMR (126 MHz, C_6D_6) δ 155.9, 154.4, 135.7, 135.2, 135.2, 135.1, 135.0, 133.3, 132.9, 132.6, 132.6, 132.6, 132.5, 131.6, 128.6, 127.5, 123.4, 122.7, 121.9, 120.6, 35.2 (Xan-CH_3), 34.8 (Xan-CH_3), 26.4 (Xan-C). **^{31}P** NMR (202 MHz, C6D6) δ -17.5 (q, J = 47.3 Hz). **Elemental microanalysis:** $C_{81}H_{67}BLiO_3P_3$+0.5(Et_2O), calc: C 80.19 %, H 6.10 %; meas: 80.24 %, 6.12 %.

2 - {(Ph_2P)xanth}$_3$B

Toluene (25 mL) was added dropwise to a mixture of **1** (200 mg, 0.17 mmol) and [$^{i}Pr_2$EtNH]Cl (28 mg, 0.17 mmol) under vigorous stirring and then heated at 100°C for 2 h. The resulting solution was cooled to 2°C and left to stand for 6 h, after which it was then filtered to remove unreacted [$^{i}Pr_2$EtNH]Cl, and volatiles then removed in vacuo. The resulting solid was washed with hexanes, extracted into toluene (50 mL) and concentrated to incipient crystallisation. Storage at -30°C resulted in crystals suitable for X-ray diffraction. Yield: 125 mg (63 %).

Some degree of contamination with the water bound product is always seen; in order to obtain spectroscopically pure material for reactivity studies, further purification is needed. **2** (25 mg, 0.02 mmol) and P_2O_5 (25 mg, 0.09 mmol) were added to an NMR tube fitted with a J. Young's valve and dissolved in toluene (ca. 0.5 mL). The reaction mixture was heated at 50°C for 3 h with regular

agitation. The resulting solution was filtered through a syringe filter and volatiles removed in vacuo. This material was then used for subsequent reactions.

Spectroscopic data: **^{1}H** NMR (500 MHz, C_6D_6) δ 7.27 (dd, J = 7.5, 1.9 Hz, 3H, ArH), 7.09 – 7.05 (m, 9H, ArH), 6.97 – 6.87 (m, 21H, ArH), 6.86 – 6.74 (m, 15H, ArH), 1.52 (s, 18H, Xan-CH_3). **^{13}C** NMR (126 MHz, C_6D_6) δ 156.6, 155.6, 139.6, 139.5, 138.4, 134.3, 134.1, 133.4, 132.0, 129.5, 129.3, 128.6, 128.4, 126.2, 123.3, 123.3, 34.9 (Xan-C), 30.8 (Xan-CH_3). **^{31}P** NMR (162 MHz, C_6D_6) δ -18.3. No ^{11}B resonance could be detected. **ES-MS accurate mass:** $C_{81}H_{66}BO_3P_3$ (M+K$^+$) calc: m/z = 1229.3950; meas: m/z = 1229.3994. **Elemental microanalysis:** $C_{81}H_{66}BO_3P_3$, calc: C 82.01 %, H 5.65 %; meas: C 79.14 %, H 5.75 %.

3 - {(Ph_2P)xanth}$_3$B·OH_2

Method A: In an NMR tube fitted with a J. Young's valve, **2** (15 mg, 12 µmol), or any of its adducts (except **4** or **5**) was dissolved in C_6D_6 (ca. 0.5 mL) and trace water was added (for example wet methanol). Quantitative conversation was seen in all cases by in situ NMR monitoring.

Method B: To a solution of [xanth(PPh_2)Li(thf)]$_2$ (500 mg, 1.25 mmol) in toluene (50 mL) at -78°C was added dropwise a solution of boron tribromide (0.83 mL of a 1 M solution in toluene, 0.83 mmol). The resulting solution was stirred for 1 h at -78°C, then warmed to room temperature before stirring for a further 16 h. Bench methanol (1 mL, 24 mmol) was then added (as a water surrogate) and the resulting cloudy solution became clear. Volatiles were removed in vacuo and the resulting sticky white powder was extracted into toluene and filtered. Volatiles were removed from the filtrate in vacuo. Yield: 324 mg (64 %). Crystals suitable for X-ray diffraction were obtained by recrystallisation from hexanes at -30°C.

Spectroscopic data: **^{1}H** NMR (500 MHz, C_6D_6) δ 10.69 (q, J = 2.3 Hz, 2H, H_2O), 7.31 – 7.27 (m, 3H, ArH), 7.22 (dd, J = 7.6, 1.8 Hz, 3H, ArH), 7.02 (t, J = 8.3 Hz, 6H, ArH), 6.94 (dd, J = 7.4, 1.7 Hz, 3H, ArH), 6.89 – 6.77 (m, 24H, ArH), 6.74 (t, J = 7.6 Hz, 3H, ArH), 6.63 (t, J = 7.5 Hz, 6H, ArH), 1.64 (s, 9H, Xan-CH_3), 1.37 (s, 9H, Xan-CH_3). **^{11}B** NMR (160 MHz, C_6D_6) δ 0.0. **^{13}C** NMR (126 MHz, C_6D_6) δ 155.4, 135.0, 134.9, 134.8, 132.8, 132.8, 132.7, 132.7, 131.9, 128.8, 128.6, 128.6, 126.5, 123.2, 123.0, 122.6, 35.0 (Xan-CH_3), 34.7 (Xan-CH_3), 25.3 (Xan-C). **^{31}P** NMR (202 MHz, C_6D_6) δ -19.6. **ES-MS accurate mass:** $C_{81}H_{68}BO_4P_3$ (M+H$^+$) calc: m/z = 1209.4496; meas: 1209.4521. **Elemental microanalysis:** $C_{81}H_{66}BO_3P_3$, calc: C 80.83 %, H 5.73 %; meas: C 80.45 %, H 5.93 %.

4 - {(Ph_2P)xanth}$_3$B·NH_2Me

Method A : In an NMR tube fitted with a J. Young's valve, **2** (15 mg, 12 µmol), or any of its adducts (except **5**) was dissolved in C_6D_6 (ca. 0.5 mL) and 1 drop of methylamine solution in THF (1 M) was added. Quantitative conversation was seen in all cases by in situ NMR monitoring.

Method B: To a solution of [xanth(PPh_2)Li(thf)]$_2$ (500 mg, 1.25 mmol) in toluene (50 mL) at -78°C was added dropwise a solution of boron tribromide (0.83 mL of a 1 M solution in toluene, 0.83 mmol). The resulting solution was stirred for 1 h at -78°C, then warmed to room temperature before stirring for a further 16 h. Methylamine (5 mL of a 1 M solution in THF, 5 mmol) was then added and the resulting cloudy solution became less turbid. Volatiles were removed in vacuo and the sticky white powder extracted into toluene and filtered. Volatiles were removed from the filtrate in vacuo. Yield: 212 mg (42 %). Crystals suitable for X-ray diffraction were obtained by recrystallisation from benzene at room temperature.

Spectroscopic data: **^{1}H** NMR (500 MHz, C_6D_6) δ 7.26 (dd, J = 7.4, 1.8 Hz, 3H, ArH), 7.20 (ddd, J = 9.8, 7.7, 1.8 Hz, 6H, ArH), 7.09 – 6.91 (m, 26H, ArH and NH_2), 6.87 (ddt, J = 7.6, 5.7, 1.6 Hz, 9H, ArH), 6.76 (td, J = 7.6, 5.3 Hz, 6H, ArH), 2.47 (t, J = 6.3 Hz, 3H, NCH_3), 1.61 (s, 9H, Xan-CH_3), 1.40 (s, 9H, Xan-CH_3). **^{11}B** NMR (160 MHz, C_6D_6) δ 0.3. **^{13}C** NMR (126 MHz, C_6D_6) δ 155.9, 138.2, 138.1, 137.2, 134.3, 134.1,

133.5, 133.4, 133.1, 132.7, 129.8, 128.6, 128.4, 126.9, 125.7, 124.0, 123.9, 123.8, 123.5, 123.2, 35.0 (Xan-CH_3), 33.8 (Xan-CH_3), 30.4 (NCH_3), 28.3 (Xan-C). **^{31}P** NMR (202 MHz, C_6D_6) δ -19.6. **ES-MS accurate mass:** $C_{82}H_{71}BNO_3P_3$ (M+H$^+$) calc: m/z = 1222.4813; meas: m/z = 1222.4862. **Elemental microanalysis:** $C_{81}H_{66}BO_3P_3$, calc: C 80.95 %, H 5.91 %, N 1.11 %; meas: C 80.86 %, H 6.07 %, N 1.19 %.

5 - {(Ph_2P)xanth}$_3$B·NH_3

Method A: In an NMR tube fitted with a J. Young's valve, **2** (15 mg, 12 µmol), or any of its adducts was dissolved in C_6D_6 (ca. 0.5 mL) and degassed by three freeze-pump-thaw cycles, after which 1 bar of NH_3 was added. Quantitative conversation was seen in all cases by in situ NMR monitoring.

Method B: To a solution of [xanth(PPh_2)Li(thf)]$_2$ (500 mg, 1.25 mmol) in toluene (50 mL) at -78°C was added dropwise a solution of boron tribromide (0.83 mL of a 1 M solution in toluene, 0.83 mmol). The resulting solution was stirred for 1 h at -78°C, then warmed to room temperature before stirring for a further 16 h. Bench methanol (1 mL, 24 mmol) was then added (as a water surrogate) and the cloudy solution became clear. Volatiles were removed in vacuo and the resulting sticky white powder was extracted into toluene and filtered. Ammonia gas was bubbled through the solution for 10 min, which was then concentrated to incipient crystallisation and stored at 30°C to produce crystalline material. Yield 242 mg (48 %). Crystals suitable for X-ray crystallography were obtained by recrystallisation from benzene at room temperature.

Spectroscopic data: **^{1}H** NMR (400 MHz, C_6D_6) δ 7.24 – 7.14 (m, 9H, ArH), 7.12 (dd, J = 6.9, 1.7 Hz, 6H, ArH), 7.01 – 6.76 (m, 30H, ArH), 6.71 (t, J = 7.6 Hz, 3H, ArH), 6.70 (s, 3H, NH3), 1.50 (s, 9H, Xan-CH_3), 1.33 (s, 9H, Xan-CH_3). **^{11}B** NMR (128 MHz, C_6D_6) δ -4.7. **^{13}C** NMR (126 MHz, C_6D_6) δ 154.4 (d, J = 8.2 Hz, Ph-P), 136.5 (d, J = 10.9 Hz, PhP), 136.1 (d, J = 10.9 Hz, Ph-P), 134.4, 132.8, 132.6, 132.5, 132.3, 131.7, 131.2, 127.8, 127.7, 127.7, 127.4, 127.2, 126.0, 122.4, 122.1, 122.1, 121.8, 33.7 (Xan-C), 30.9 (Xan-CH_3), 29.5 (Xan-CH_3). **^{31}P** NMR (202 MHz, C_6D_6) δ -21.7. **ES-MS accurate mass:** $C_{81}H_{69}BNO_3P_3$ (M+H$^+$) calc: m/z = 1208.4656; meas: m/z = 1208.4679. **Elemental microanalysis:** $C_{81}H_{69}BNO_3P_3$, calc: C 80.89 %, H 5.82 %, N 1.12 %; meas: C 80.08 %, H 6.00 %, N 1.32 %.

8 - **{(Ph_2P)xanth}$_3$B·CO**

In an NMR tube fitted with a J. Young's valve, a solution of **2** (15 mg, 12 µmol), in *ortho*difluorobenzene (ca. 0.5 mL) was degassed by three freeze-pump-thaw cycles, after which 1 bar of CO was added. Quantitative conversation was seen in this concentration regime by in situ NMR monitoring. Crystals suitable for X-ray diffraction were formed upon standing. Upon removal of solvent in vacuo complete conversion back to **2** was observed. VT NMR studies were carried out using a sample prepared in similar fashion dissolved in C_7D_8 (d_8-toluene) using (Ph_2P)xanthH as the standard.

Spectroscopic data: **^{1}H** NMR (500 MHz, C_6D_6) δ 7.26 (dd, J = 7.7, 1.7 Hz, 3H, ArH), 7.11 (t, J = 7.3 Hz, 12H, ArH), 7.05 (q, J = 6.9 Hz, 6H, ArH), 6.98 (d, J = 6.1 Hz, 6H, ArH), 6.91 (dd, J = 5.7, 1.8 Hz, 3H, ArH), 6.88 – 6.72 (m, 18H, ArH), 1.56 (s, 9H, Xan-CH_3), 1.05 (s, 9H, Xan-CH_3). **^{11}B** NMR (160 MHz, C_6D_6) δ -14.8. **^{13}C** NMR (126 MHz, C_6D_6) δ 171.7 (CO), 155.6, 155.6, 155.5, 140.8 (d, J = 15.9 Hz, Ph-P), 136.9 (d, J = 17.3 Hz, Ph-P), 135.1, 134.2, 134.0, 133.7, 133.5, 132.5, 129.9, 128.8, 128.7, 127.6, 126.9, 125.7 (d, J = 18.6 Hz, Ph-P), 125.1, 124.1, 123.3, 35.0 (Xan-CH_3), 34.5 (Xan-CH_3), 27.5 (Xan-C). **^{31}P** NMR (162 MHz, C_6D_6) δ -21.8. **IR:** 2179 cm^{-1}, ν(CO). **ES-MS accurate mass:** $C_{82}H_{69}BNO_4P_3$ (M+K$^+$) calc: m/z = 1257.3899; meas m/z = 1257.3896.

9 - **{(Ph_2P)xanth}$_3$B·NCMe**

In an NMR tube fitted with a J. Young's valve **2** (15 mg, 12 µmol), was dissolved in C_6D_6 (ca. 0.5 mL) and acetonitrile (0.21 mL of a 0.1 in C_6D_6, 0.02 mmol) was added. Quantitative conversation was seen

by in situ NMR monitoring and crystals suitable for X-ray diffraction were obtained upon standing. Upon removal of solvent in vacuo complete conversion back to **2** was observed.

Spectroscopic data: **^{1}H** NMR (500 MHz, C_6D_6) δ 7.32 (dd, J = 7.7, 1.7 Hz, 3H, ArH), 7.21 (dd, J = 7.4, 1.7 Hz, 3H, ArH), 7.18 (d, J = 1.7 Hz, 3H, ArH), 7.15 – 6.99 (m, 18H), 6.87 – 6.76 (m, 15H, ArH), 6.74 (t, J = 7.6 Hz, 3H, ArH), 1.65 (s, 9H, Xan-CH_3), 1.34 (s, 9H, Xan-CH_3), 0.60 (s, 3H, NC-CH_3 (overlapped with free MeCN)). **^{11}B** NMR (160 MHz, C_6D_6) δ -5.3. **^{13}C** NMR (126 MHz, C_6D_6) δ 156.8 , 140.5 (d, J = 16.8 Hz, PhP), 140.4 (d, J = 19.1 Hz, PhP), 137.9 (Me-CN) 135.8, 134.3, 134.1, 133.8, 133.2 (d, J = 2.8 Hz, PhP), 133.0, 132.9, 129.5, 128.6 (d, J = 4.6 Hz, PhP), 127.4, 126.8, 124.4, 124.3, 123.8, 123.3, 123.1, 115.9, 35.1 (Xan-CH_3), 34.7 (Xan-CH_3), 27.0 (Xan-C), 0.1 (NC*C*H_3). **^{31}P** NMR (202 MHz, C_6D_6) δ -19.7. **IR:** 2356 cm^{-1} ν(CN). **ES-MS accurate mass:** $C_{83}H_{69}BNO_3P_3$ ($M+H^+$) calc: m/z = 1232.4656; meas: m/z = 1232.4268.

10 - {(Ph_2P)xanth}$_3$B·CN^{t}Bu

In an NMR tube fitted with a J. Young's valve **2** (15 mg, 12 µmol) was dissolved in toluene (ca. 0.5 mL) and tert-butyl isocyanide (0.1 mL of a 1 M solution in toluene, 0.1 mmol) added. The solution was heated at 80°C for 18 h, at which point quantitative conversation was indicated by in situ NMR monitoring. Volatiles were then removed in vacuo and the resulting solid redissolved in C_6D_6. Crystals suitable for X-ray diffraction were obtained by slow evaporation of solvent.

Spectroscopic data: **^{1}H** NMR (500 MHz, C_6D_6) δ 7.26 – 7.17 (m, 9H, ArH), 7.11 (d, J = 5.6 Hz, 3H, ArH), 7.01 (dp, J = 8.6, 2.7 Hz, 21H, ArH), 6.94 – 6.89 (m, 9H, ArH), 6.72 (dt, J = 16.8, 7.5 Hz, 6H, ArH), 1.55 (s, 9H, Xan-CH_3), 1.26 (s, 9H, Xan-CH_3), 0.52 (s, 9H, CN-^{t}Bu). **^{11}B** NMR (160 MHz, C_6D_6) δ -16.2. **^{13}C** NMR (126 MHz, C_6D_6) δ 158.3, 158.1(NC), 157.1, 142.0 (d, J = 18.7 Hz, PhP), 139.3 (d, J = 18.8 Hz, PhP), 136.6, 136.2, 134.7 (d, J = 3.3 Hz, PhP), 133.7, 133.6, 133.4, 133.3, 129.5, 128.6, 127.5 (d, J = 5.5 Hz, PhP), 126.3, 124.1, 124.0, 123.7, 123.5, 123.2, 35.3 (Xan-CH_3), 34.0 ($C(CH_3)_3$), 30.2 (Xan-C), 28.1 (Xan-CH_3), 24.8 (CN-^{t}Bu). **^{31}P** NMR (202 MHz, C_6D_6) δ -22.0. **IR:** 2285 cm^{-1} ν(NC). **ES-MS accurate mass:** $C_{86}H_{75}BNO_3P_3$ ($M+H^+$) calc: m/z = 1274.5126; meas: m/z = 1274.5138.

11 - [^{n}Bu$_4$N][{(Ph_2P)xanth}$_3$B·CN]

In order to prevent the formation of HCN gas, a modified preparation of **2** was used. **2** (25 mg, 0.021 mmol) and P_2O_5 (25 mg, 0.088 mmol) were added to an NMR tube fitted with a J. Young's valve and dissolved in toluene. The reaction mixture was heated at 50°C for 3 h with regular agitation. After filtration, LiH (10 mg, 1.26 mmol) was added and the mixture agitated for a further 15 min. The resulting suspension was filtered into an NMR tube fitted with a J. Young's valve containing tetrabutylammonium cyanide (5.64 mg, 0.021 mmol). The resulting solution was heated at 80°C for 18 h, at which point quantitative conversation was indicated by in situ NMR monitoring; volatiles were removed in vacuo. Crystals suitable for X-ray diffraction where obtained by heating a suspension of **11** until it fully dissolved, followed by slow cooling to room temperature.

Spectroscopic data: **^{1}H** NMR (500 MHz, C_6D_6) δ 9.75 (d, J = 6.9 Hz, 2H, ArH), 8.72 (s, 2H, ArH), 7.93 (t, J = 6.6 Hz, 3H, ArH), 7.62 (t, J = 6.7 Hz, 3H, ArH), 7.52 (d, J = 7.4 Hz, 2H, ArH), 7.45 (t, J = 6.9 Hz, 5H, ArH), 7.37 (t, J = 7.1 Hz, 3H, ArH), 7.25 (ddd, J = 22.0, 14.6, 7.2 Hz, 6H, ArH), 7.19 – 7.12 (m, 4H, ArH), 7.10 (s, 6H, ArH), 7.14 – 7.03 (m, 3H, ArH), 7.03 – 6.89 (m, 3H, ArH), 6.79 – 6.61 (m, 5H, ArH), 6.34 (t, J = 7.3 Hz, 1H, ArH), 2.66 – 2.59 (m, 8H, N-*CH_2*), 1.71 (d, J = 3.5 Hz, 6H, Xan-*CH_3*), 1.60 (s, 3H, Xan-*CH_3*), 1.30 (s, 3H, Xan-*CH_3*), 1.08 (p, J = 7.5 Hz, 8H, CH_2-*CH_2*-CH_2), 0.99 (p, J = 7.1 Hz, 11H, CH_2-*CH_2*-CH_3), 0.91 (s, 3H), 0.73 (t, J = 7.2 Hz, 12H, CH_2-*CH_3*). **^{11}B** NMR (128 MHz, C_6D_6) δ -13.6. **^{13}C** NMR (126 MHz, C_6D_6) δ 155.5 (CN), 154.4, 154.0, 142.3, 141.4, 140.8, 139.5, 139.4, 138.9, 137.2, 136.7, 136.6, 136.1, 134.3, 134.2, 133.8, 133.7, 133.6, 133.4, 133.3, 132.7, 132.5, 132.4, 131.3, 129.0, 127.2, 126.1, 125.6, 125.5, 125.3, 123.3, 121.4, 121.1, 121.0, 120.6, 120.5, 120.2, 56.9 (N-*CH_2*), 35.0 (Xan-*CH_3*), 33.5 (Xan-*CH_3*), 33.2 (Xan-*CH_3*), 29.6 (Xan-*C*), 27.8 (Xan-*C*), 27.6 (Xan-*C*), 22.9 (CH_2-*CH_2*-CH_2), 18.5 (CH_2-*CH_2*-CH_3), 12.7

(CH_2-CH_3). ^{31}**P** NMR (202 MHz, C_6D_6) δ -18.0, -20.2, -23.7. **ES-MS accurate mass:** $C_{82}H_{66}BNO_3P_3$ (M^+) calc: m/z = 1216.4354; meas: m/z = 1216.4397.

6 - {(^{i}Pr$_2$P)xanth}$_3$B·NH$_2$Me

To a solution of [xanth(P^{i}Pr$_2$)Li]$_4$ (500 mg, 1.50 mmol of monomer) in toluene (50 mL) at -78°C was added dropwise a solution of boron tribromide (1 mL of a 1 M solution in toluene, 1.00 mmol). The resulting solution was stirred for 1 h at -78°C, then warmed to room temperature and stirred for a further 16 h. Methylamine (5 mL of a 1 M solution in THF, 5 mmol) was then added and the resulting cloudy solution became less turbid. Volatiles were removed in vacuo and the sticky white powder was extracted into toluene (ca. 20 mL) and filtered. Volatiles were removed from the filtrate in vacuo. Yield: 432 mg (85 %). Crystals suitable for X-ray diffraction were obtained by recrystallisation from benzene at room temperature.

Spectroscopic data: ^{1}**H** NMR (500 MHz, C_6D_6) δ 7.99 (s, 1H, NH), 7.62 (s, 1H,NH), 7.45 – 7.39 (m, 1H, ArH), 7.35 – 7.30 (m, 3H, ArH), 7.09 (dt, *J* = 7.51, 2.25 Hz, 3H, ArH), 7.03 – 6.88 (m, 6H, ArH), 6.82 (t, *J* = 7.53 Hz, 3H, ArH), 3.48 (d, *J* = 6.02 Hz, 3H, NCH_3), 2.47 – 2.28 (m, 3H, CH), 1.84 (pd, *J* = 7.04, 3.10 Hz, 3H, CH), 1.67 (s, 9H, Xan-CH_3), 1.58 (s, 9H, Xan-CH_3), 1.23 (q, *J* = 7.02 Hz, 2H, CH_2CH_3), 1.05 – 0.91 (m, 18H, CH_2CH_3), 0.69 – 0.54 (m, H, CH_2CH_3). ^{11}**B** NMR (160 MHz, C_6D_6) δ -1.4. ^{13}**C** NMR (126 MHz, C_6D_6) δ 155.0, 136.8, 131.7, 130.5, 130.1, 128.8, 128.0, 126.1, 123.4, 122.9, 121.7, 116.4, 34.5 (Xan-C), 31.3 (Xan-CH_3), 31.3 (Xan-CH_3), 24.5 (CH), 24.4 (CH), 23.7 (CH), 23.6 (CH), 23.5 (NCH_3), 20.8 (CH_3), 20.6 (CH_3), 20.0 (CH_3), 19.9 (CH_3), 19.9 (CH_3), 19.8 (CH_3), 19.8 (CH_3), 19.7 (CH_3), 19.6 (CH_3), 18.4 (CH_3), 18.3 (CH_3). ^{31}**P** NMR (202 MHz, C_6D_6) δ -12.0. **ES-MS accurate mass:** $C_{64}H_{83}BNO_3P_3$ ($M+H^+$) calc: m/z = 1018.5752; meas: m/z = 1018.5775. **Elemental microanalysis:** $C_{64}H_{83}BNO_3P_3$, calc: C 76.12 %, H 8.20 %, N 1.32 %; meas: C 75.48 %, H 8.03 %, N 1.27 %.

7 - {(^{i}Pr$_2$P)xanth}$_3$B·NH$_3$

In an NMR tube fitted with a J. Young's valve **6** (25 mg, 0.0246 mmol) was dissolved in C_6D_6 and degassed by three freeze-pump-thaw cycles after which 1 bar of NH_3 was added. The solution was then heated at 80°C for 72 h. Crystals suitable for X-ray diffraction could be obtained by slow evaporation of the solvent. In all cases <50% conversation is seen, and **6** co-crystallises such that separation is not possible. NMR and mass data for 7 are reported form this product mixture. The ^{1}H NMR spectrum could not be assigned as signals overlap significantly with those of **6**, especially in the ^{i}Pr region of the spectrum.

Spectroscopic data: ^{11}**B** NMR (128 MHz, C_6D_6) δ -5.3. ^{31}**P** NMR (162 MHz, C_6D_6) δ -12.9. **ES-MS accurate mass:** $C_{63}H_{81}BNO_3P_3$ ($M+K^+$) calc: m/z = 1042.5154; meas: m/z = 1042.5148.

Spectra of new compounds

NMR

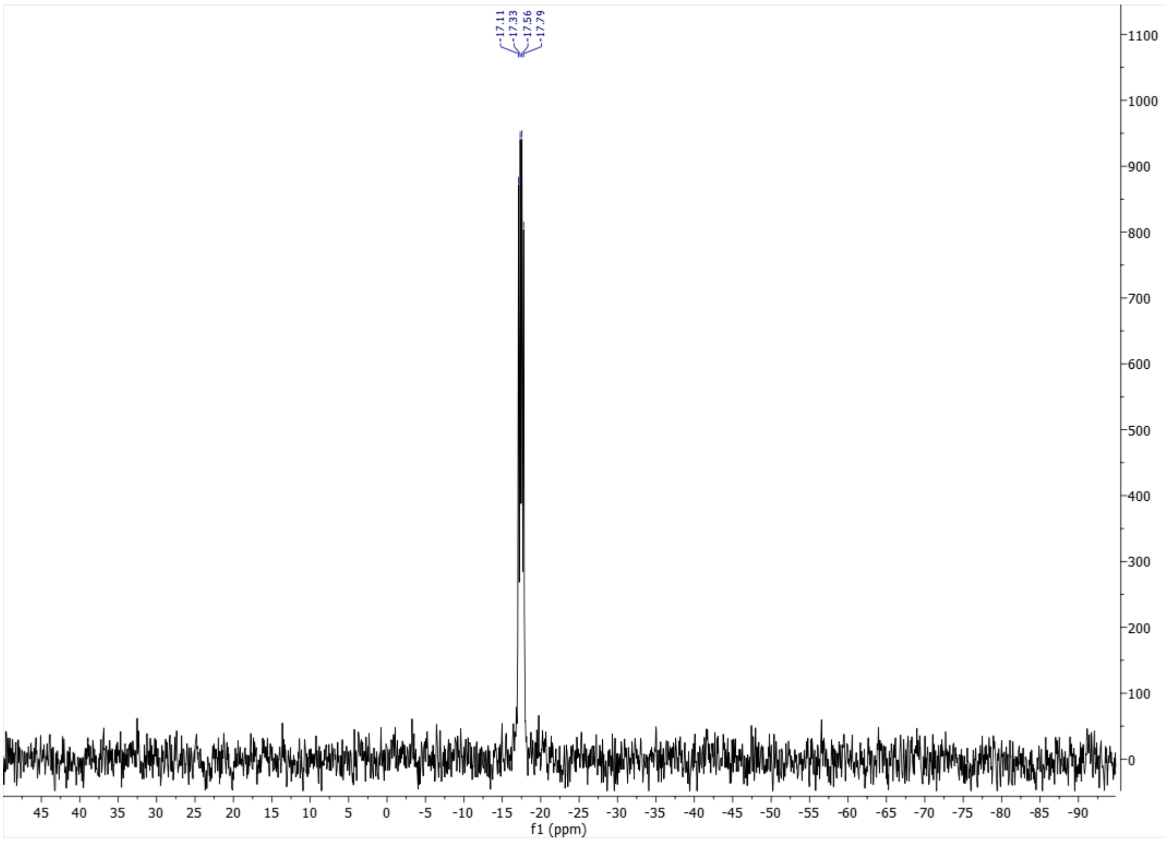

Figure S1: $^{31}P\{^{1}H\}$ NMR spectrum of **1**

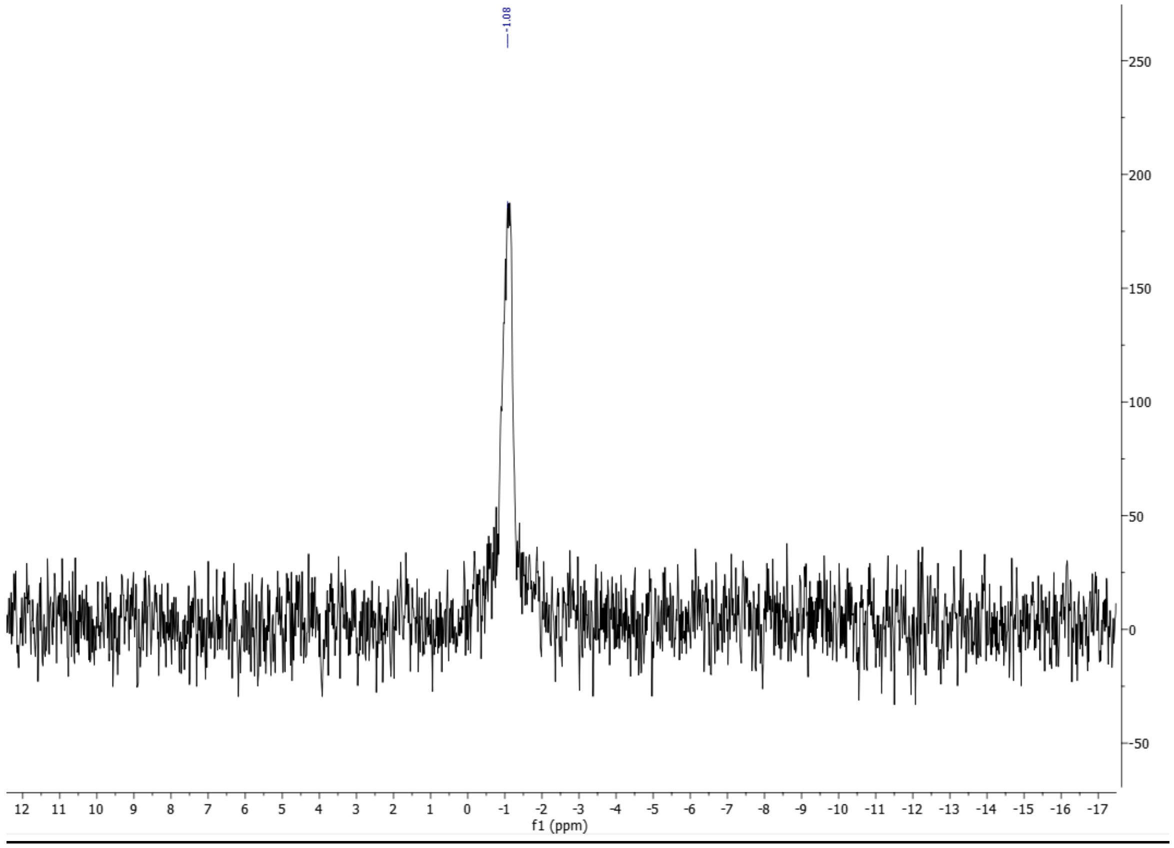

Figure S2: $^{7}Li\{^{1}H\}$ NMR spectrum of **1**

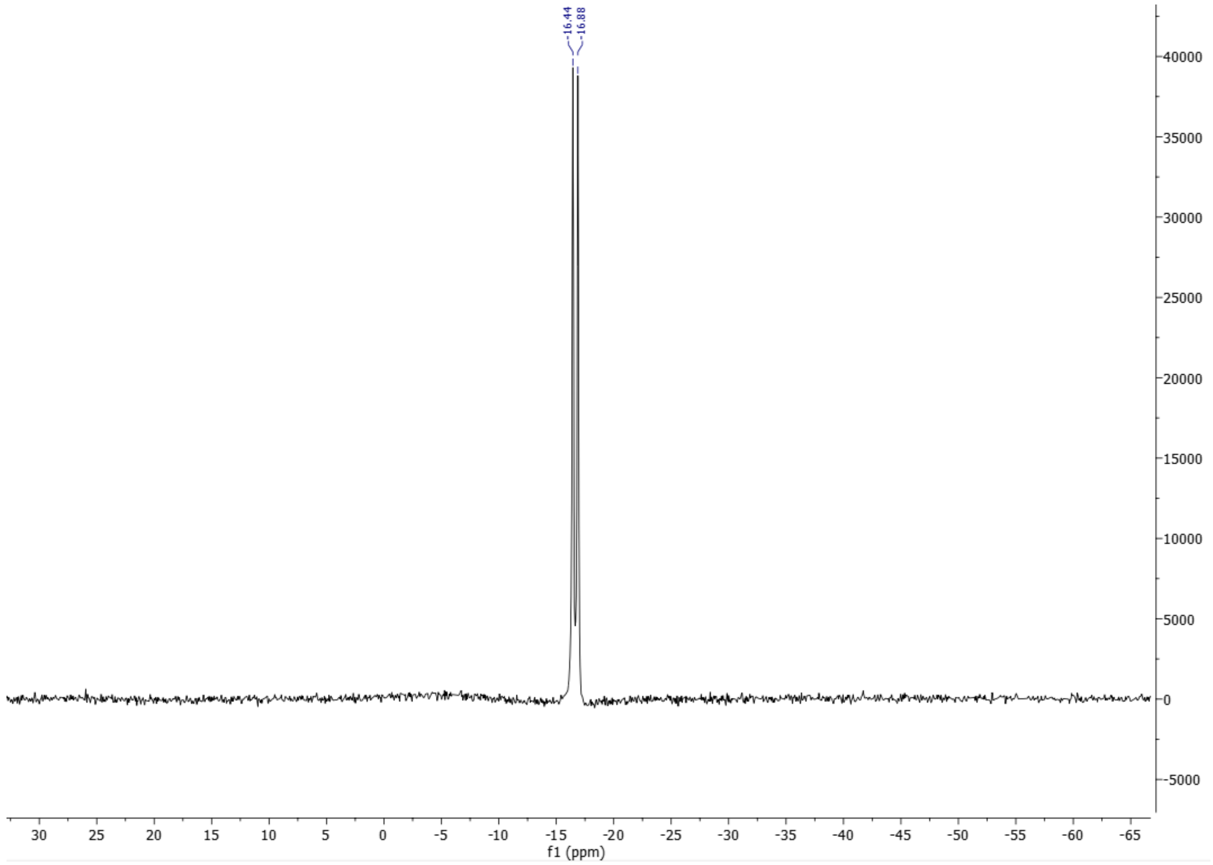

Figure S3: ^{11}B NMR spectrum of **1**

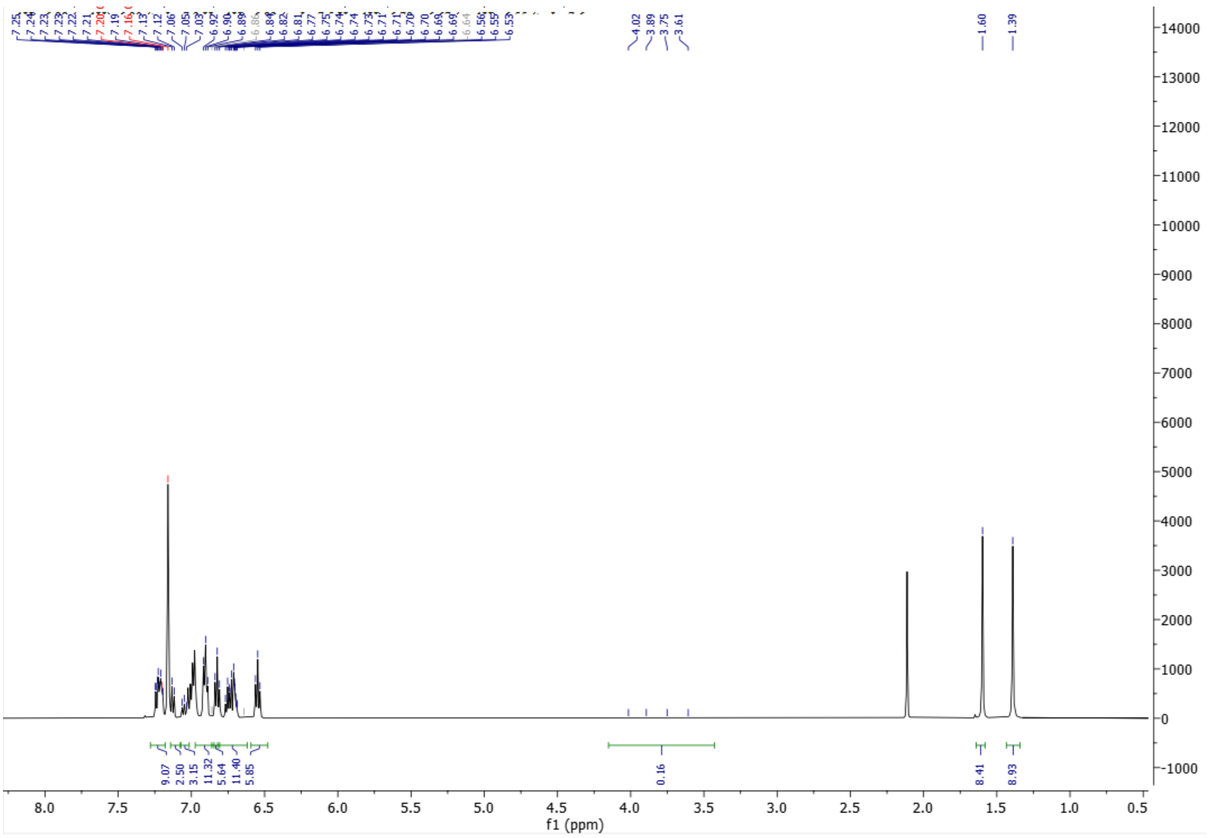

Figure S4: ^{1}H NMR spectrum of **1**

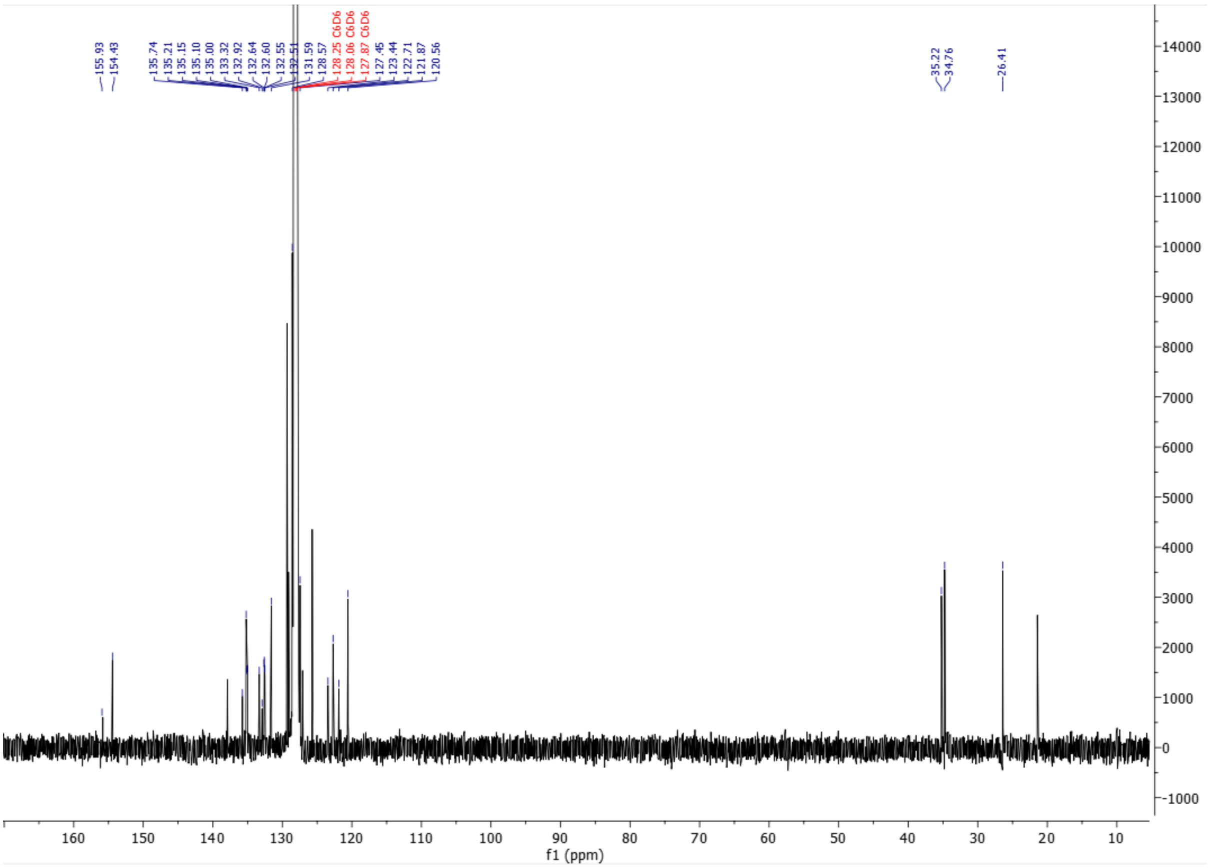

Figure S5: $^{13}C\{^1H\}$ NMR spectrum of **1**

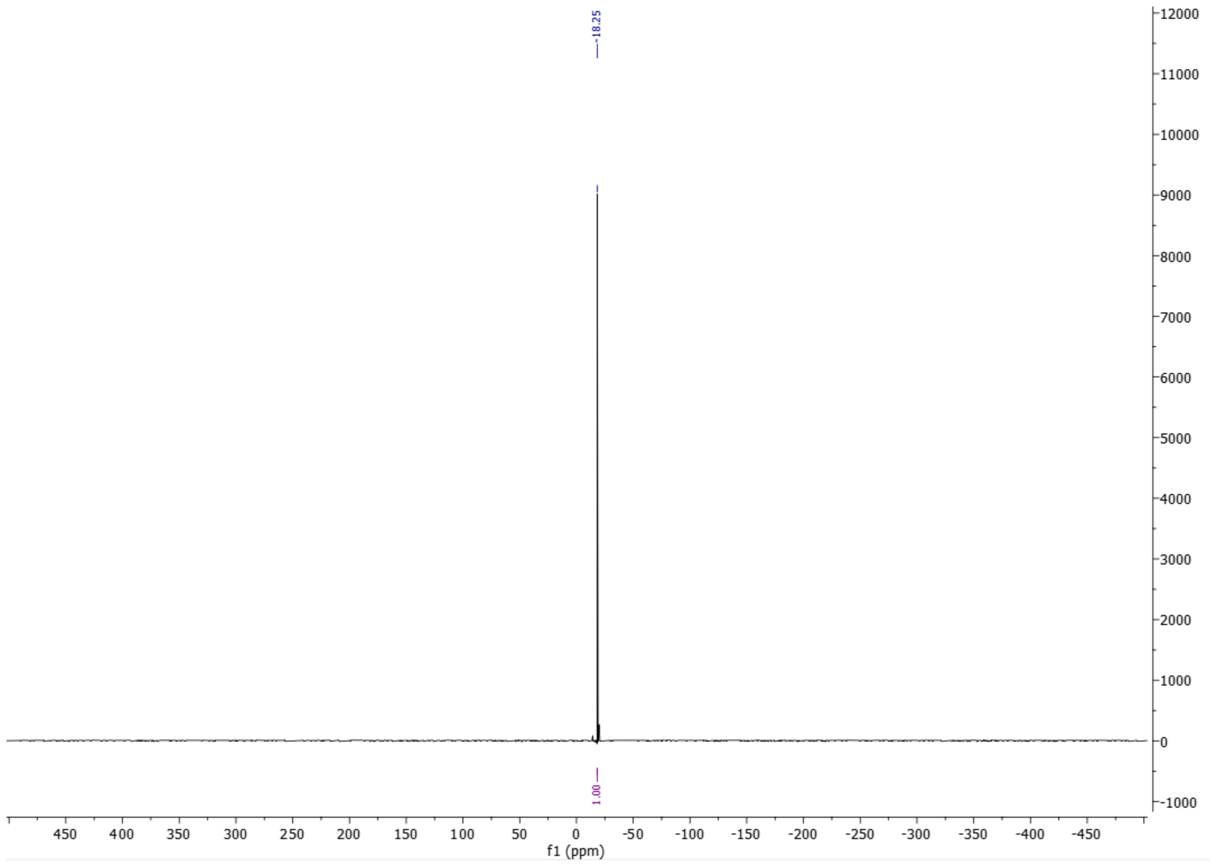

Figure S6: $^{31}P\{^{1}H\}$ NMR spectrum of **2**

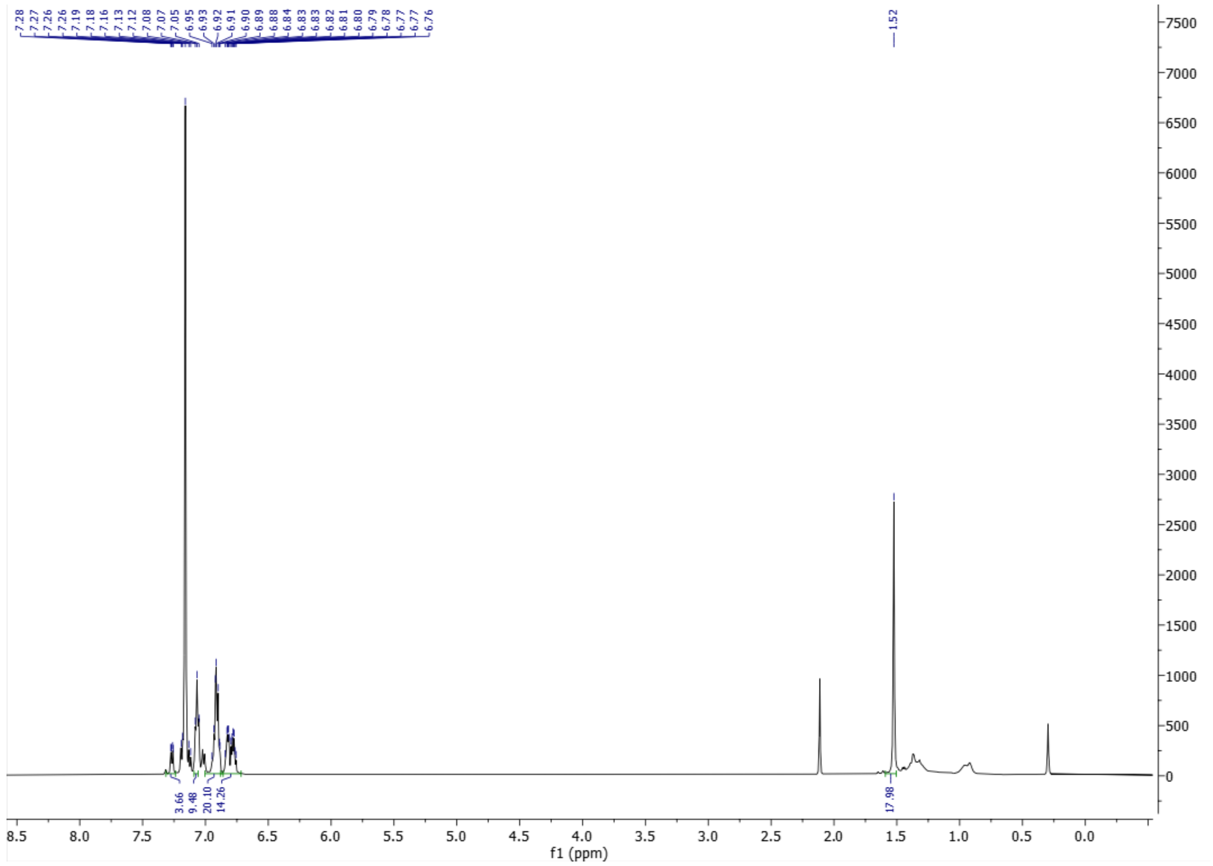

Figure S7: ^{1}H NMR spectrum of **2**

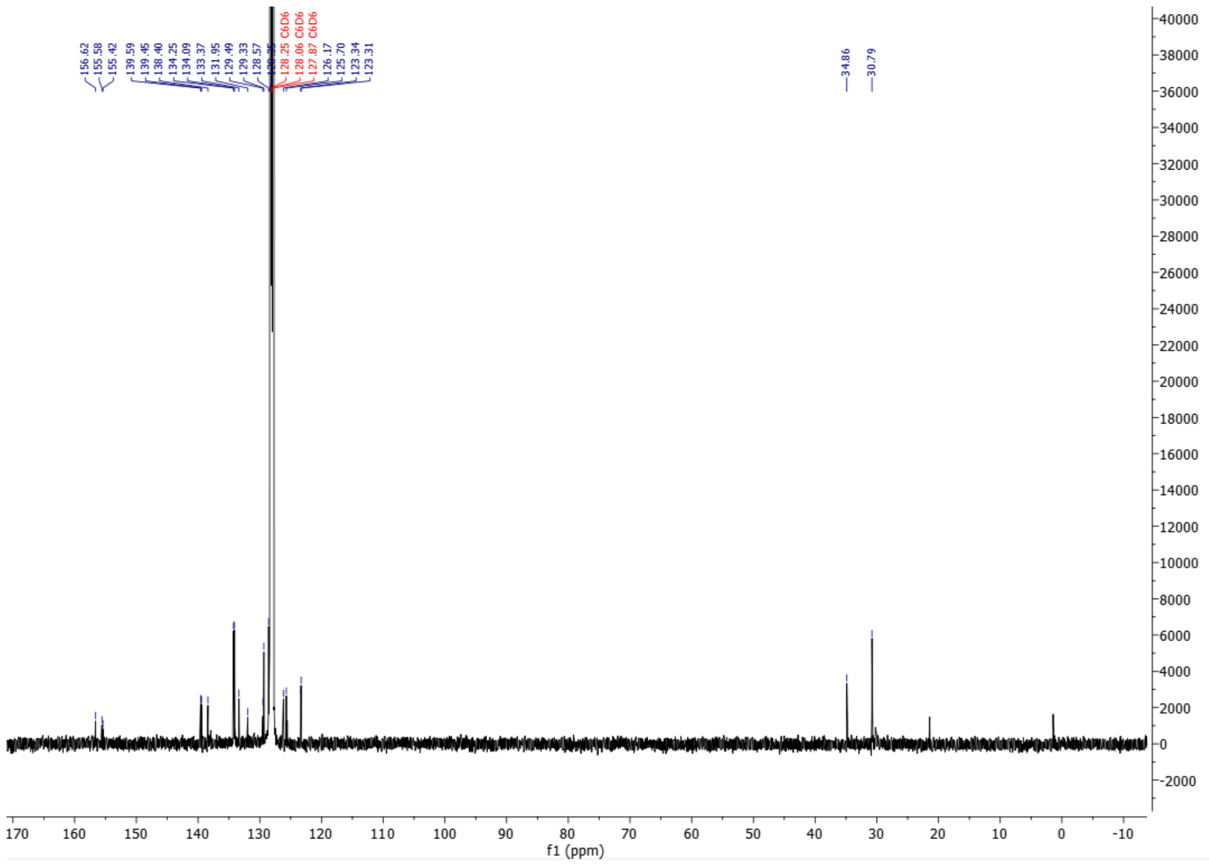

Figure S8: $^{13}C\{^1H\}$ NMR spectrum of **2**

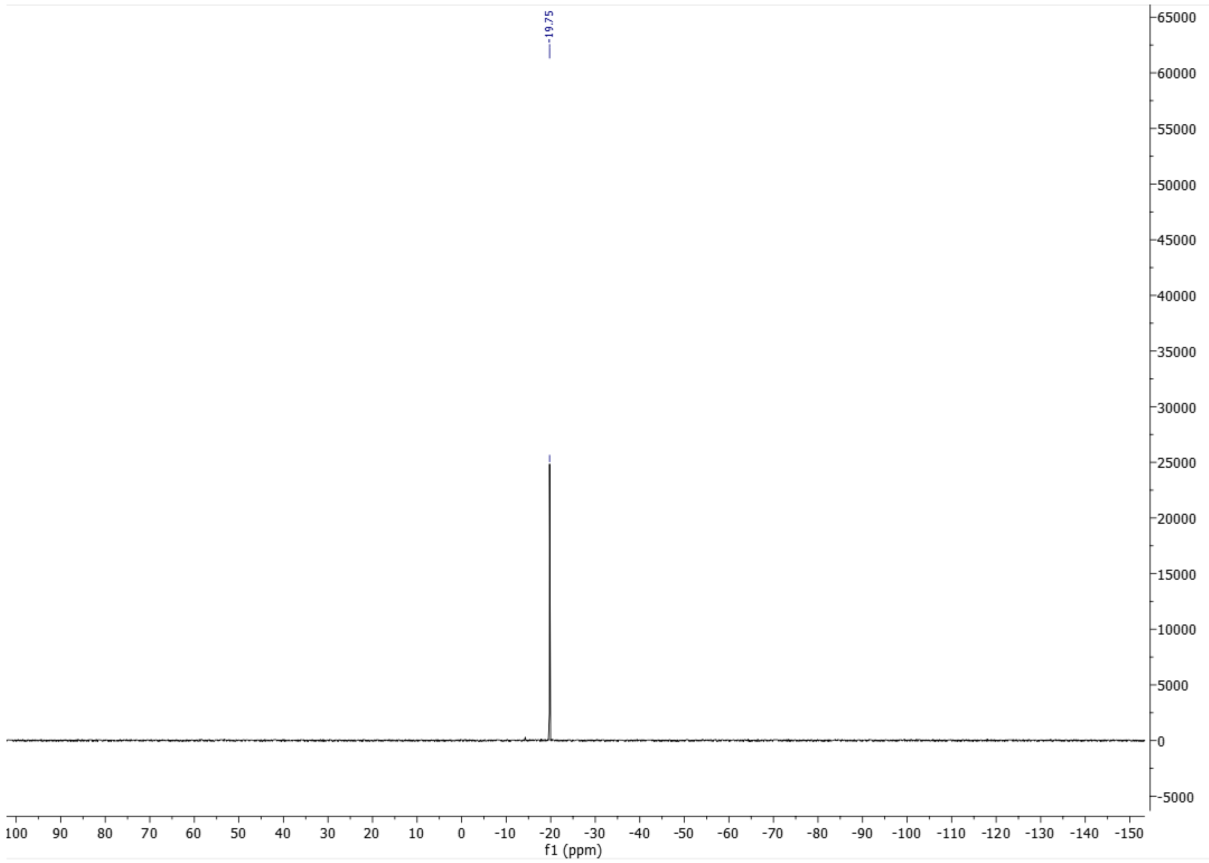

Figure S9: $^{31}P\{^1H\}$ NMR spectrum of **3**

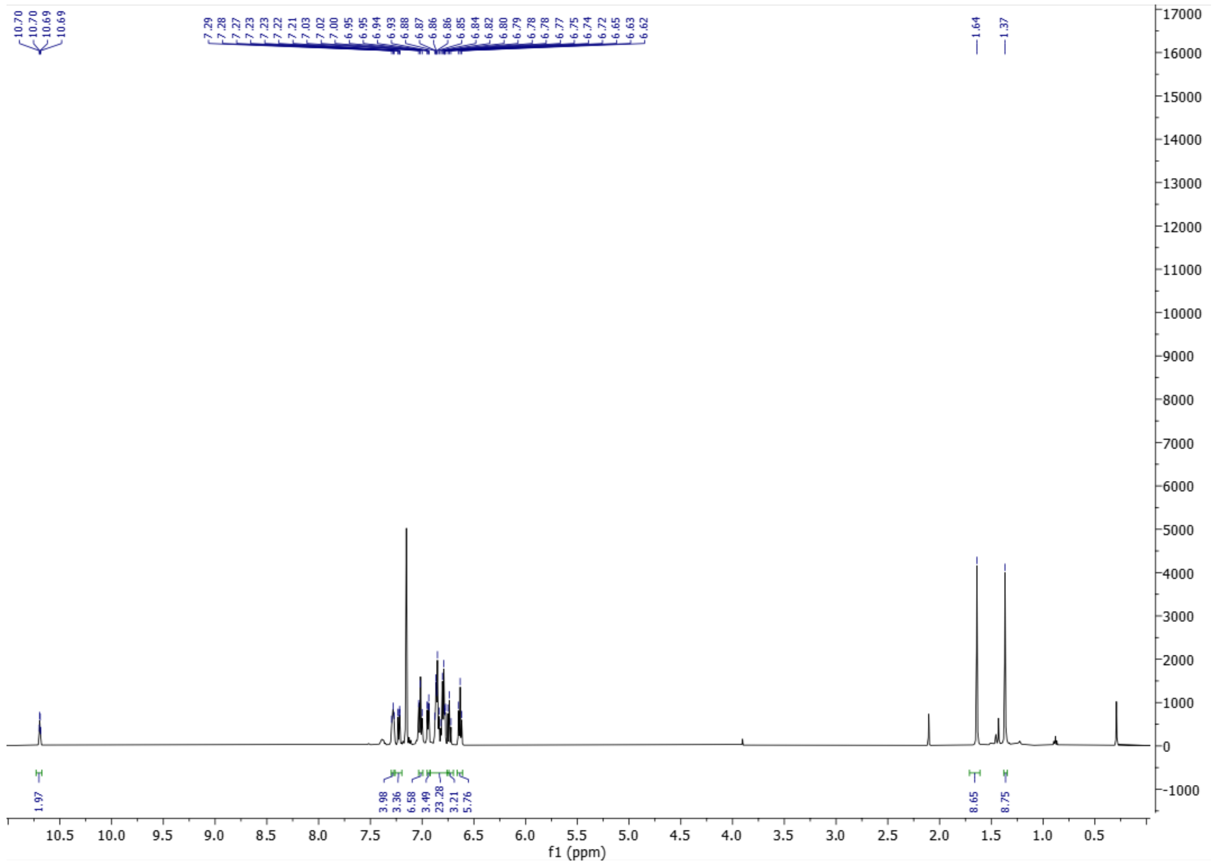

Figure S10: ^{1}H NMR spectrum of **3**

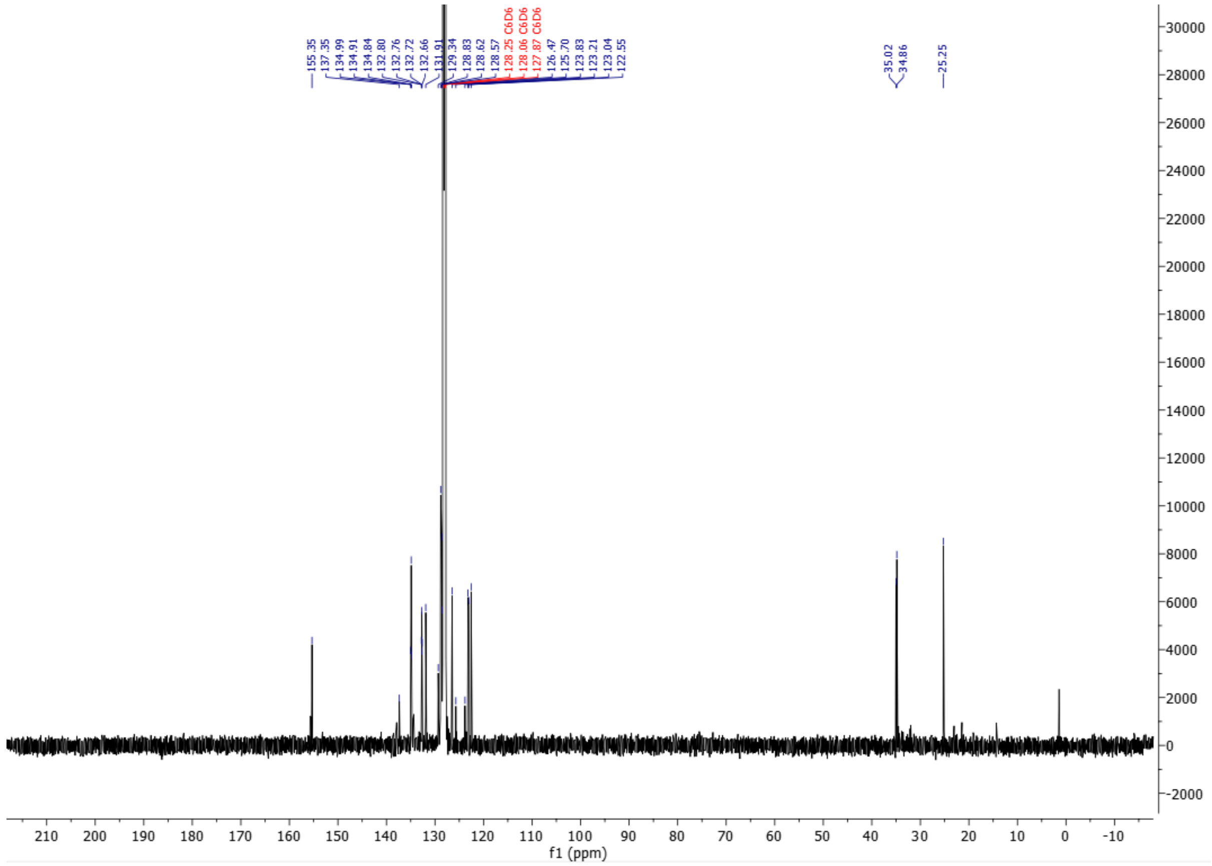

Figure S11: $^{13}C\{^{1}H\}$ NMR spectrum of **3**

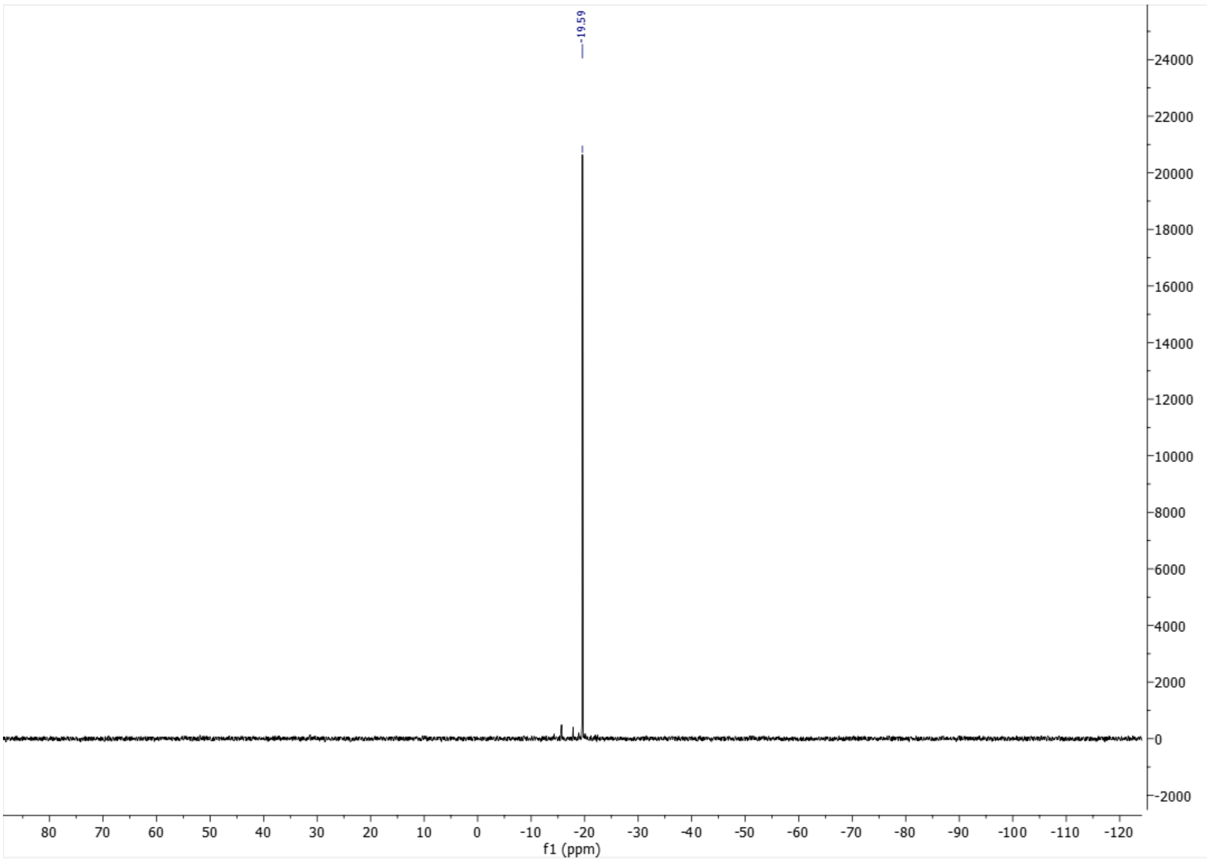

Figure S12: $^{31}P\{^{1}H\}$ NMR spectrum of **4**

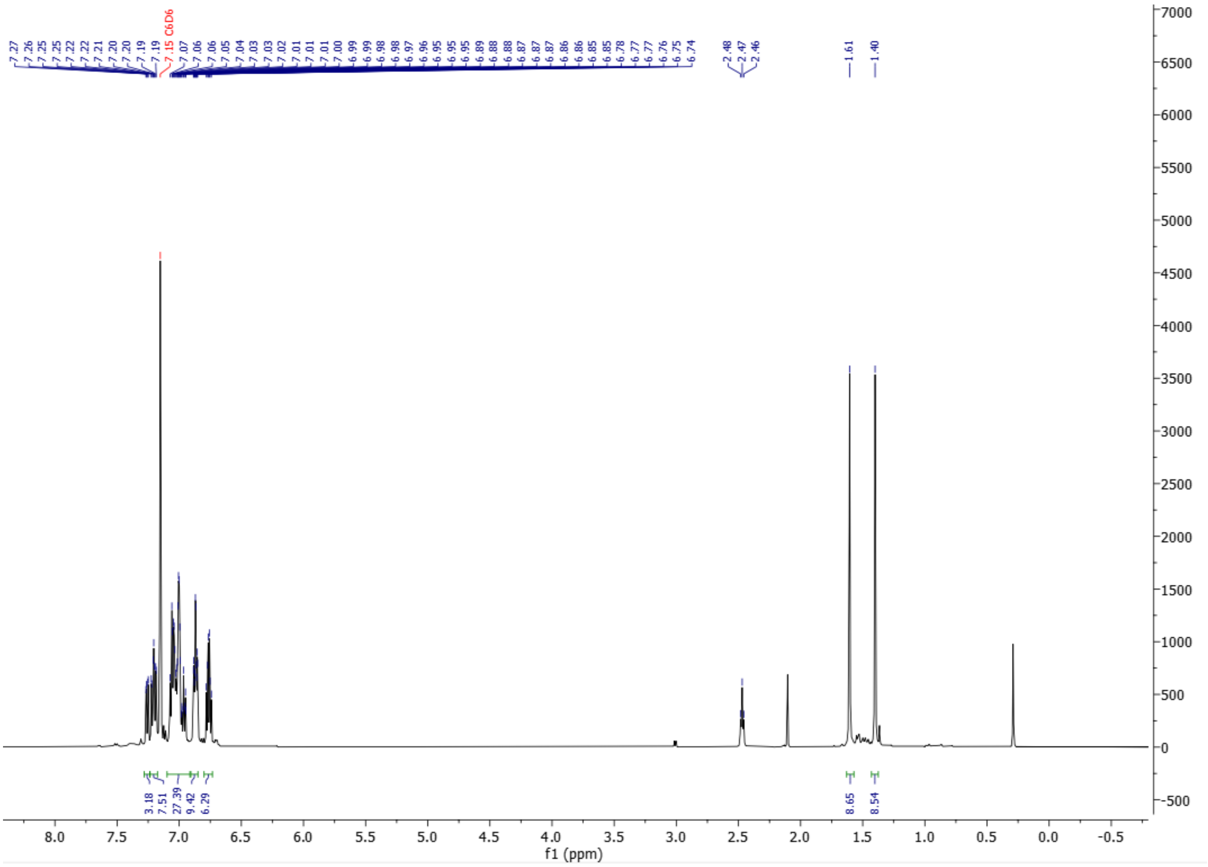

Figure S13: ^{1}H NMR spectrum of **4**

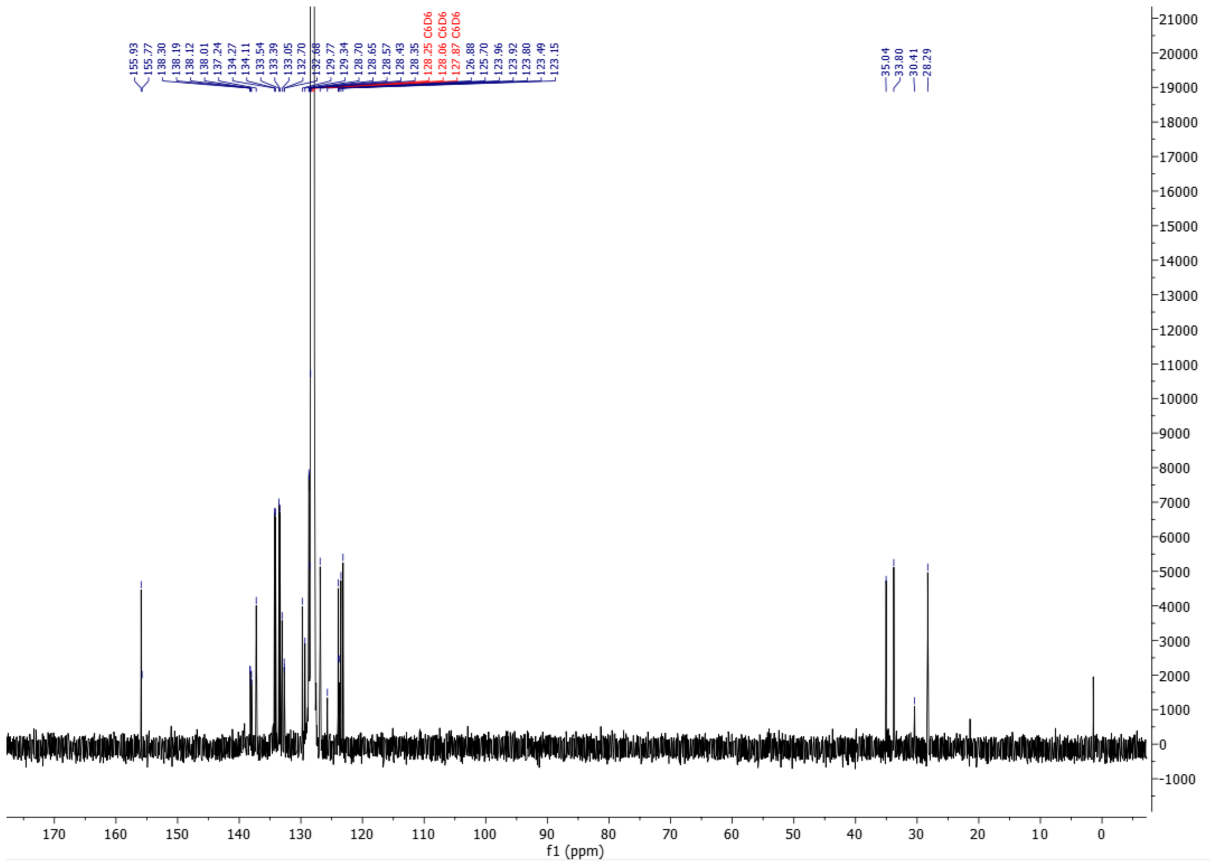

Figure S14: ^{13}C {^{1}H} NMR spectrum of **4**

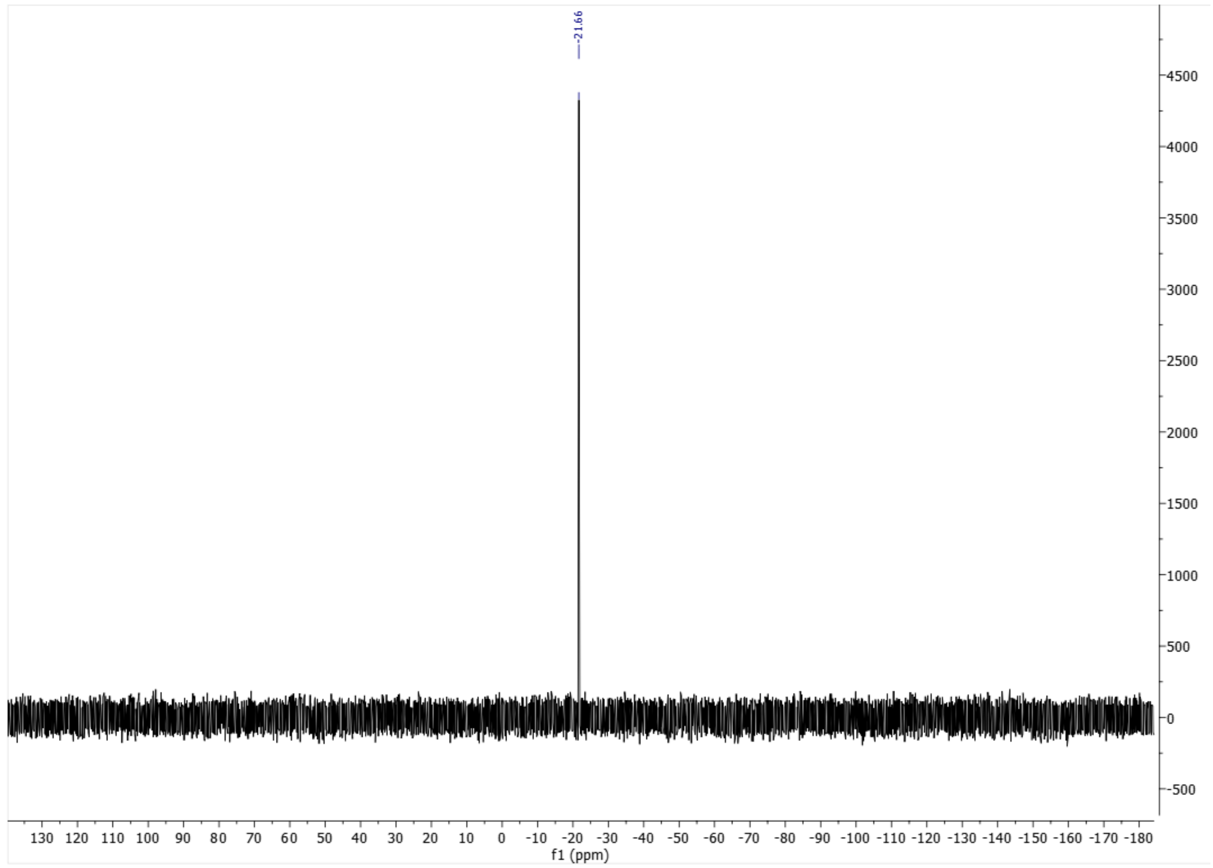

Figure S15: ^{31}P {^{1}H} NMR spectrum of **5**

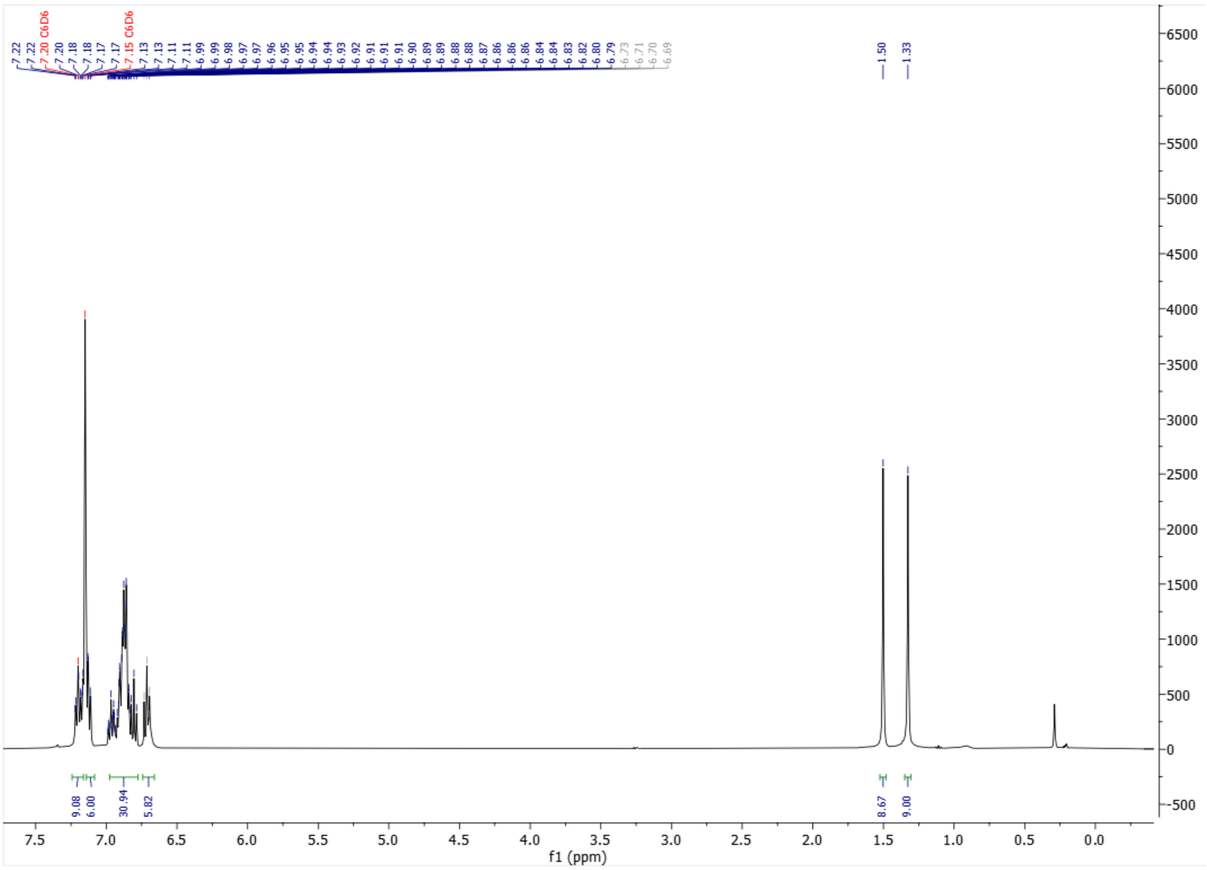

Figure S16: ^{1}H NMR spectrum of **5**

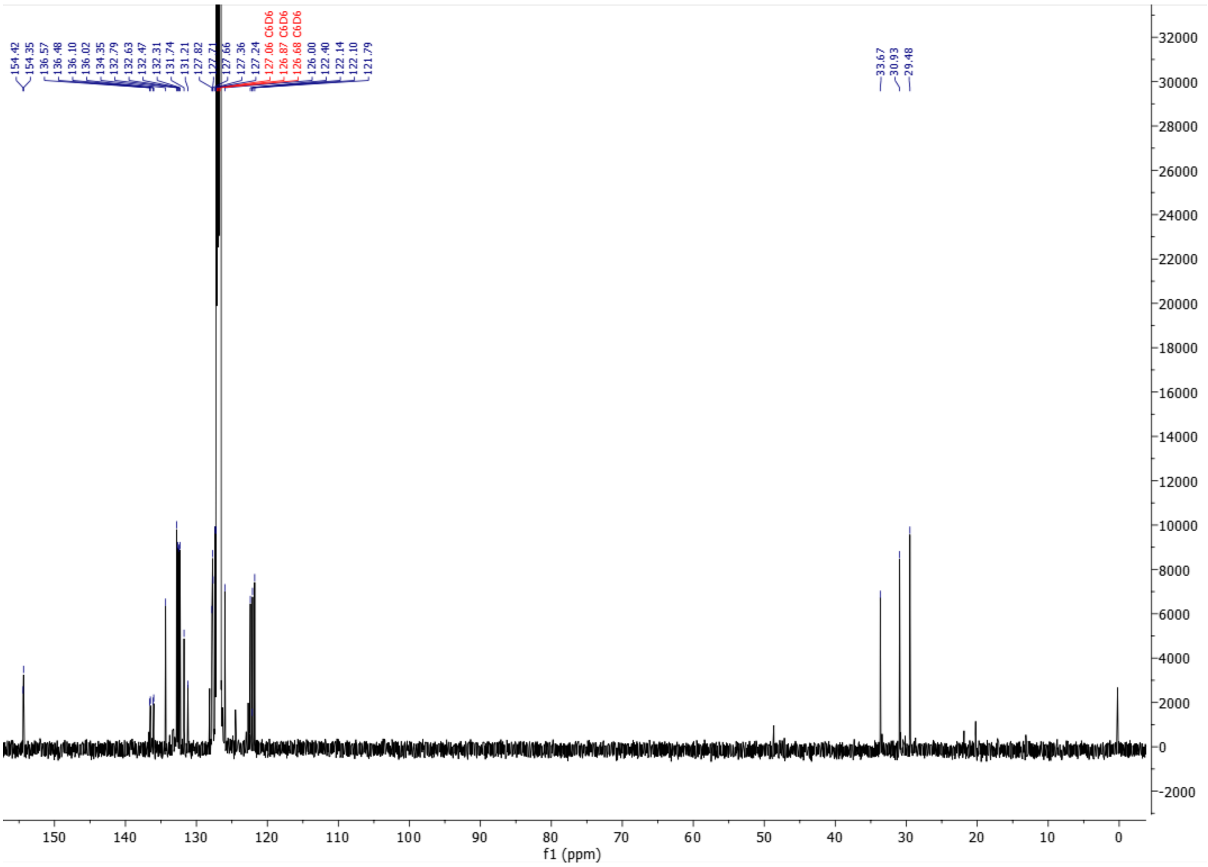

Figure S17: $^{13}C\{^{1}H\}$ NMR spectrum of **5**

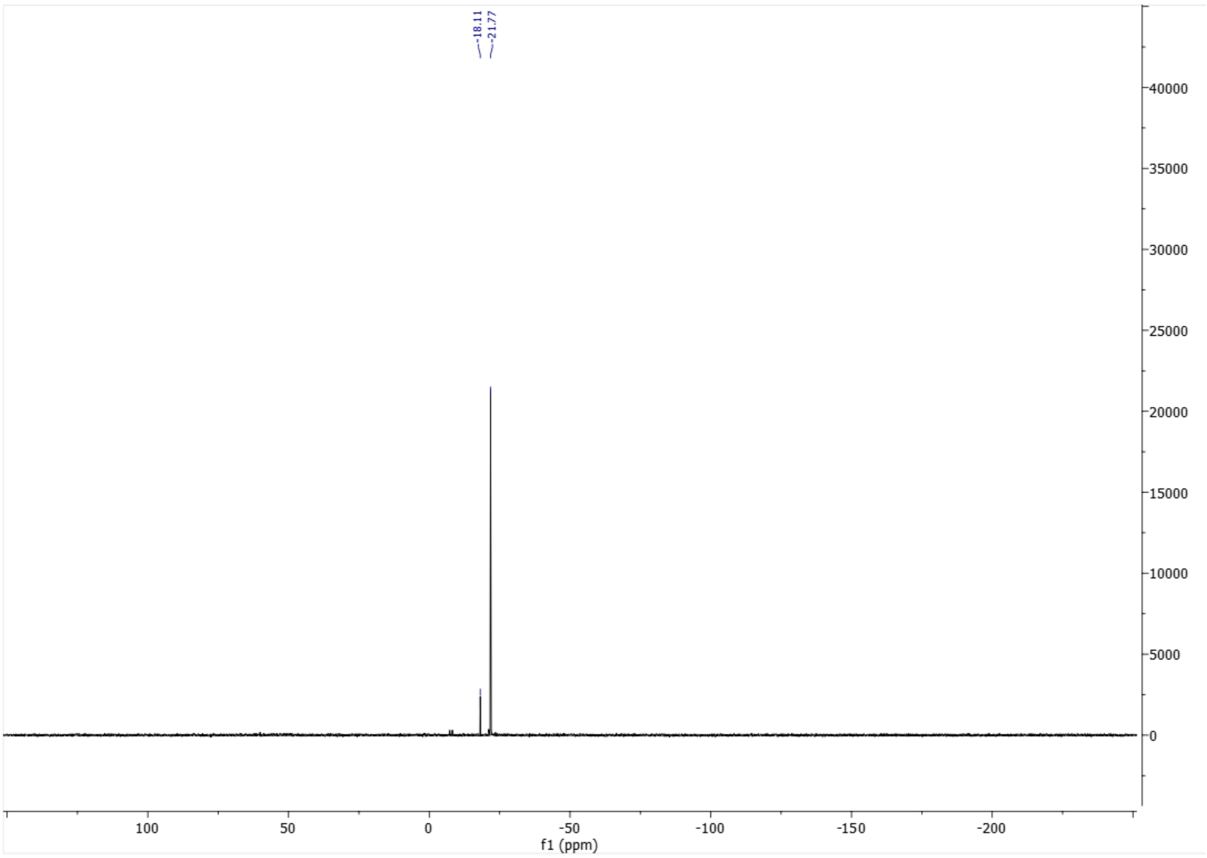

Figure S18: $^{31}P\{^1H\}$ NMR spectrum of **8** with a trace **2**

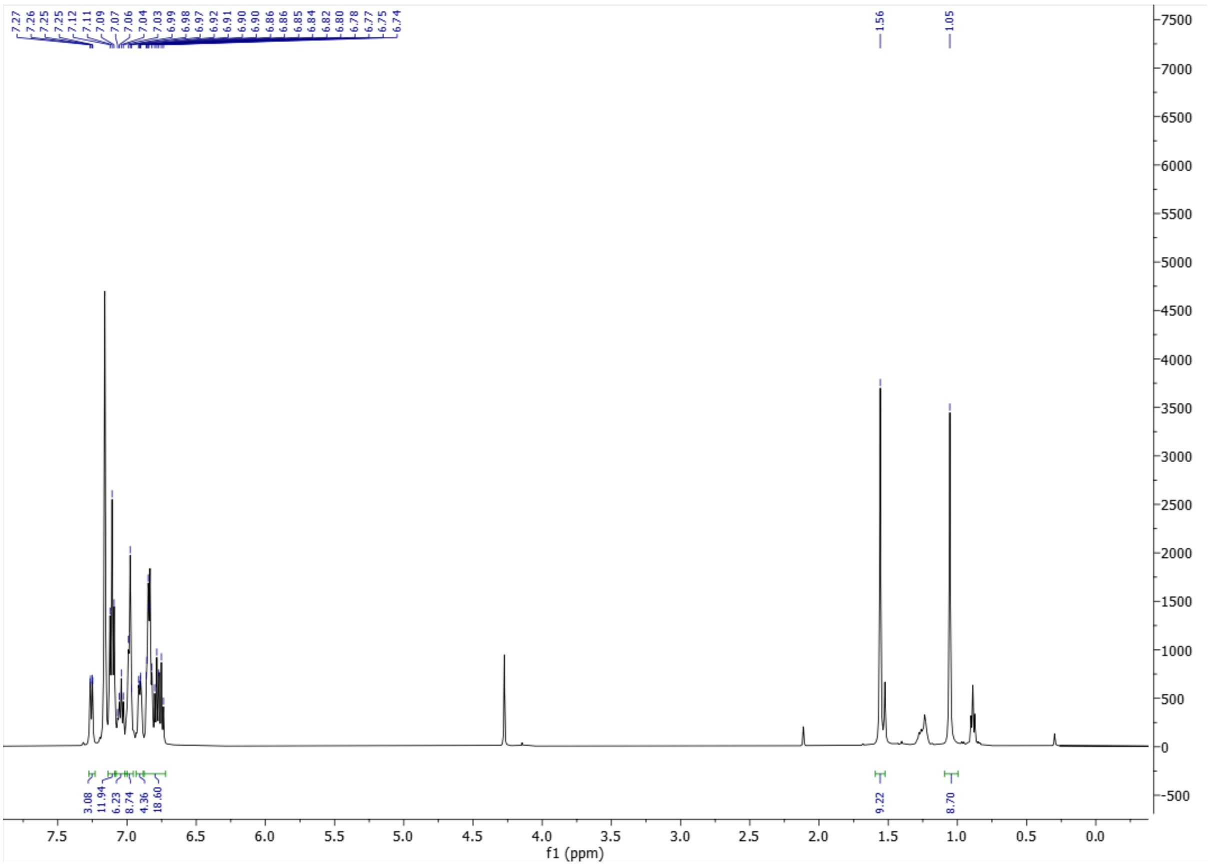

Figure S19: ^1H NMR spectrum of **8**

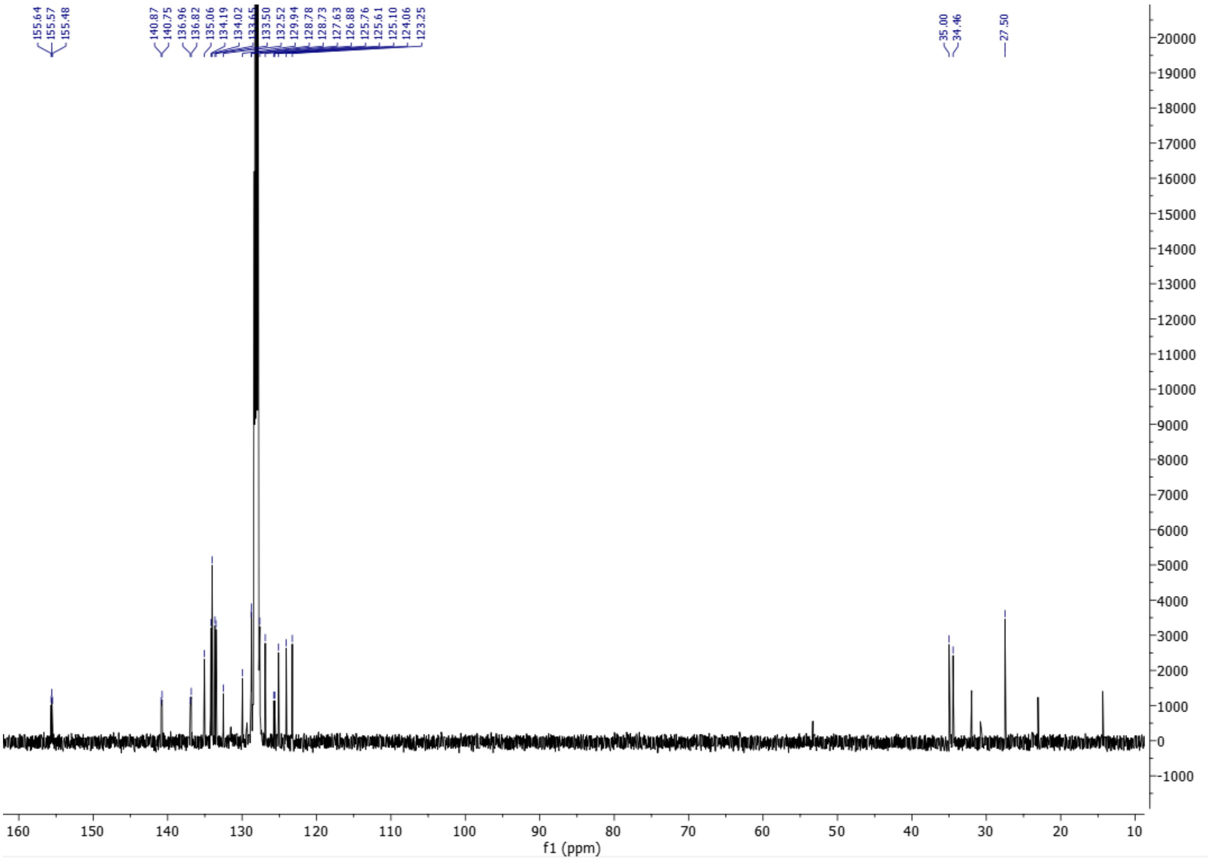

Figure S20: $^{13}C\{^{1}H\}$ NMR spectrum of **8**

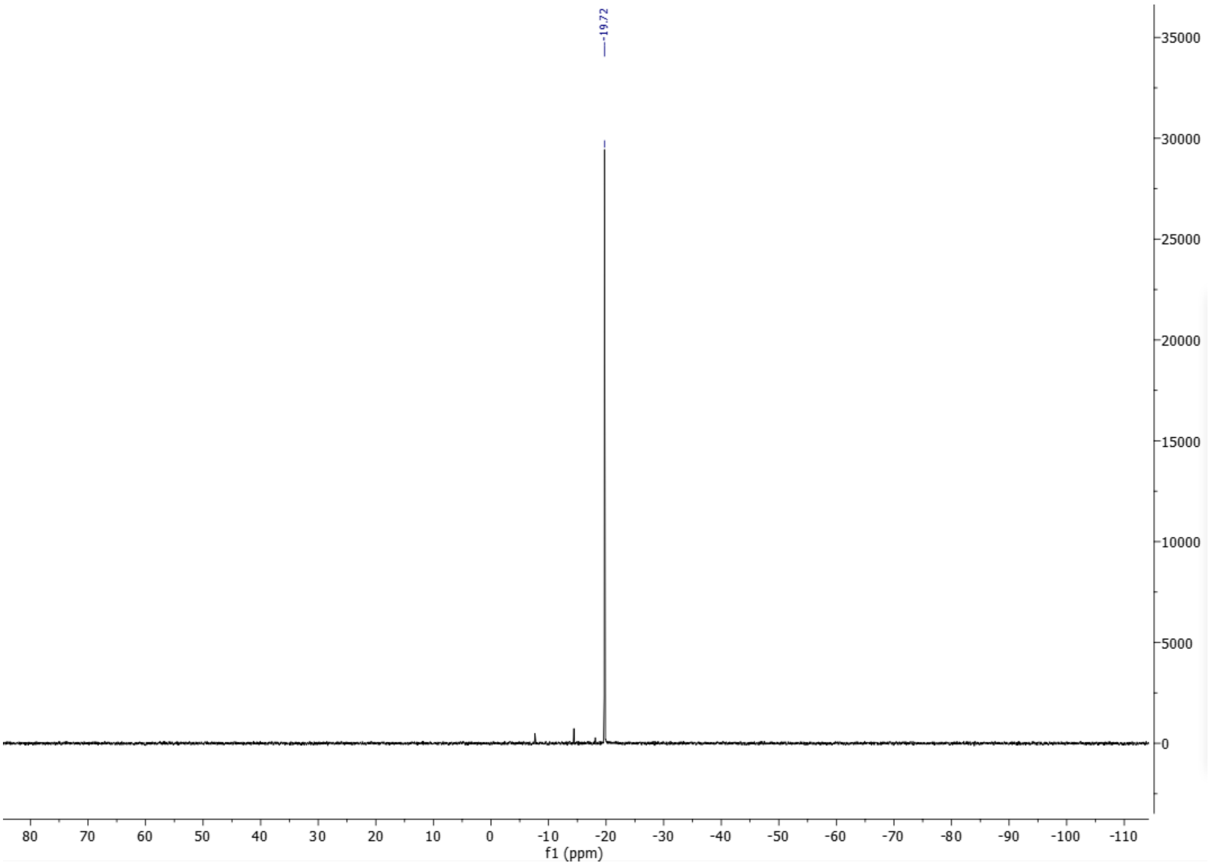

Figure S21: $^{31}P\{^{1}H\}$ NMR spectrum of **9**

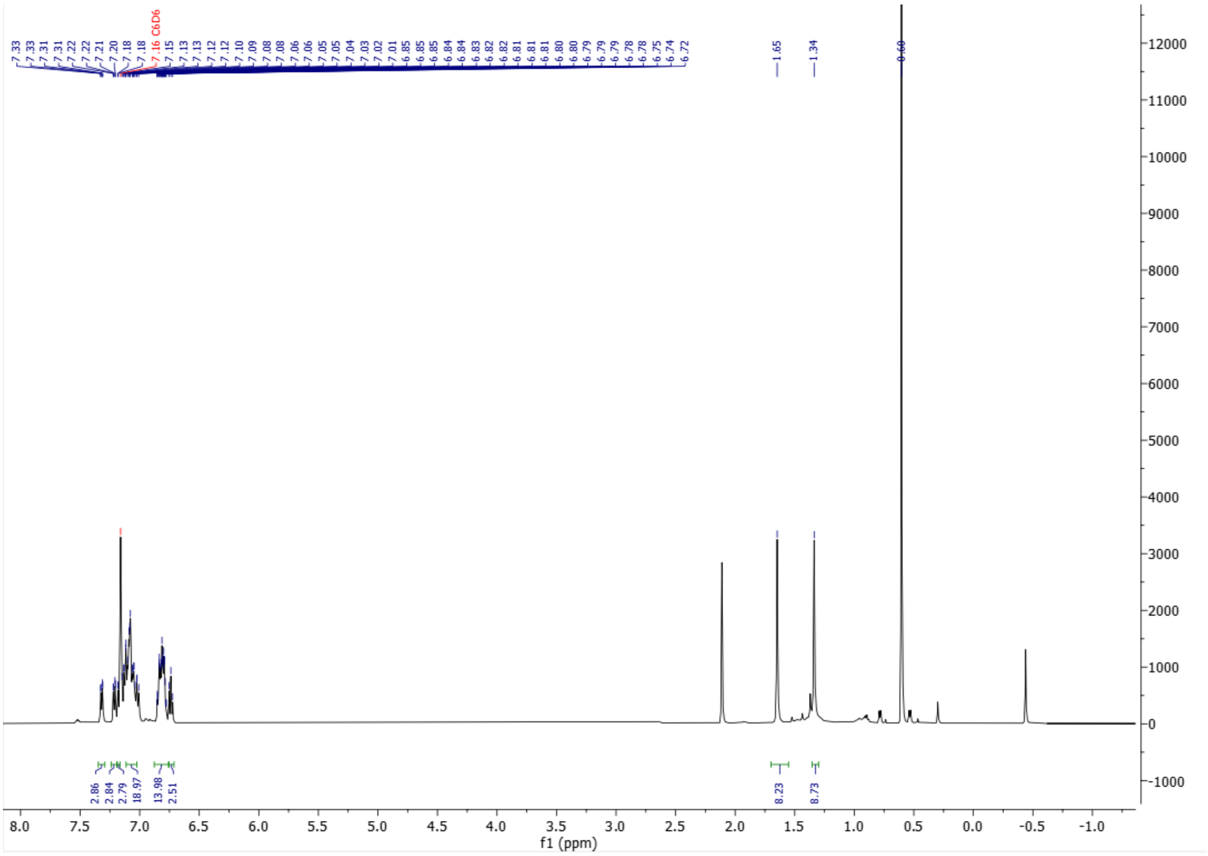

Figure S22: ^{1}H NMR spectrum of **9**

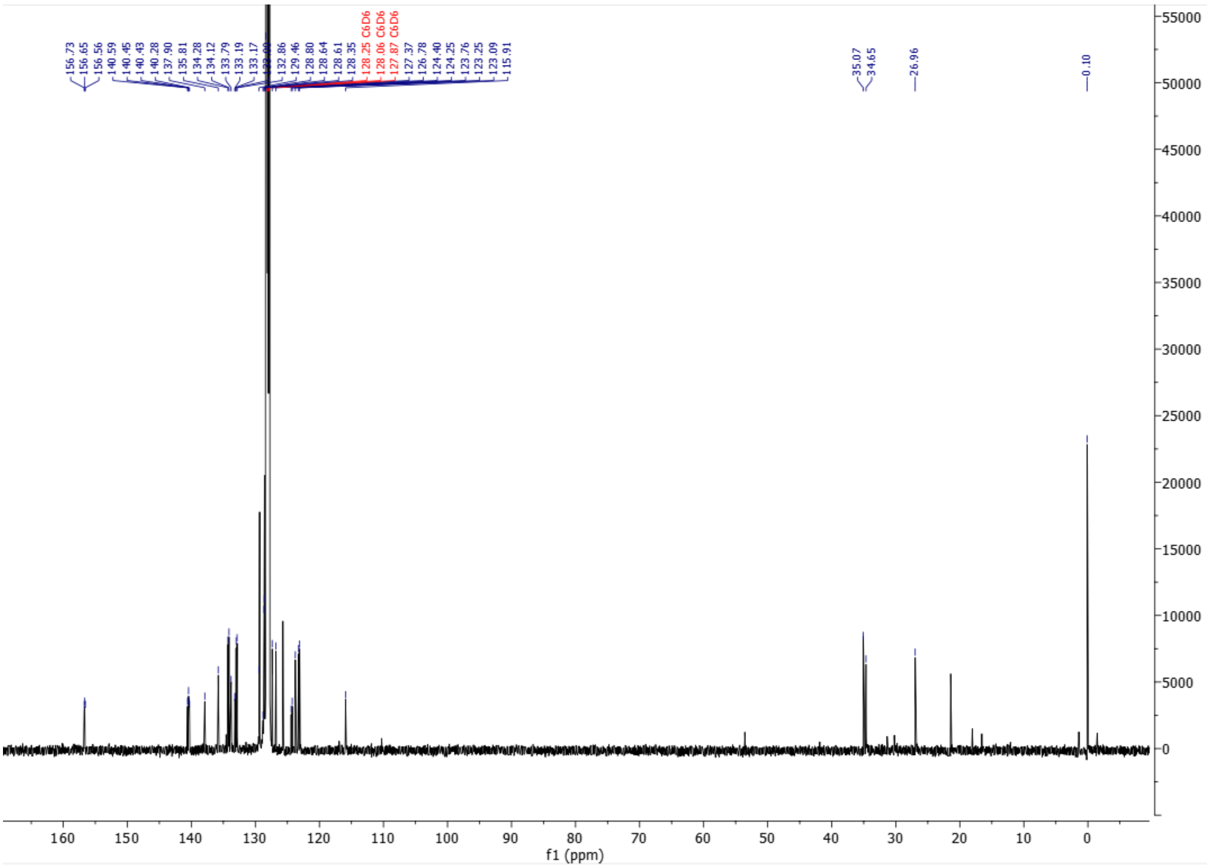

Figure S23: $^{13}C\{^{1}H\}$ NMR spectrum of **9**

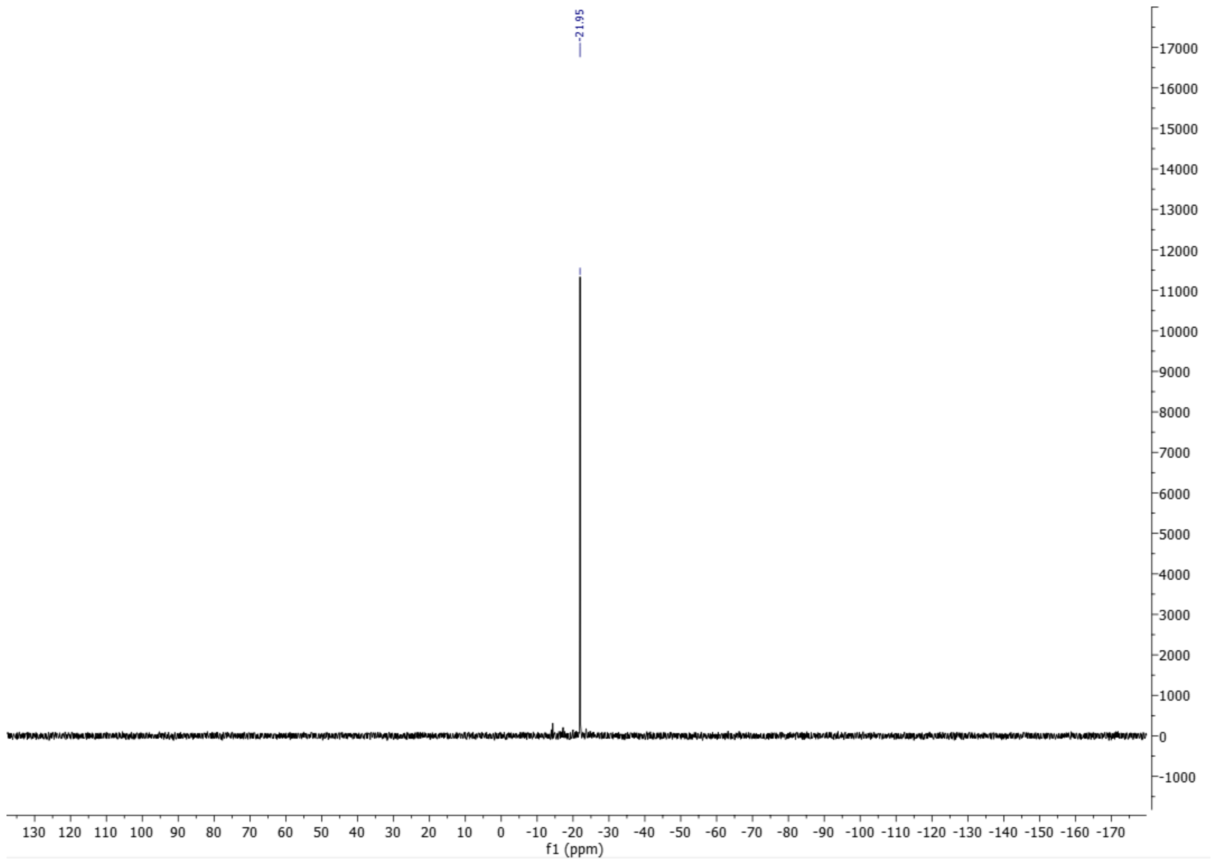

Figure S24: $^{31}P\{^1H\}$ NMR spectrum of **10**

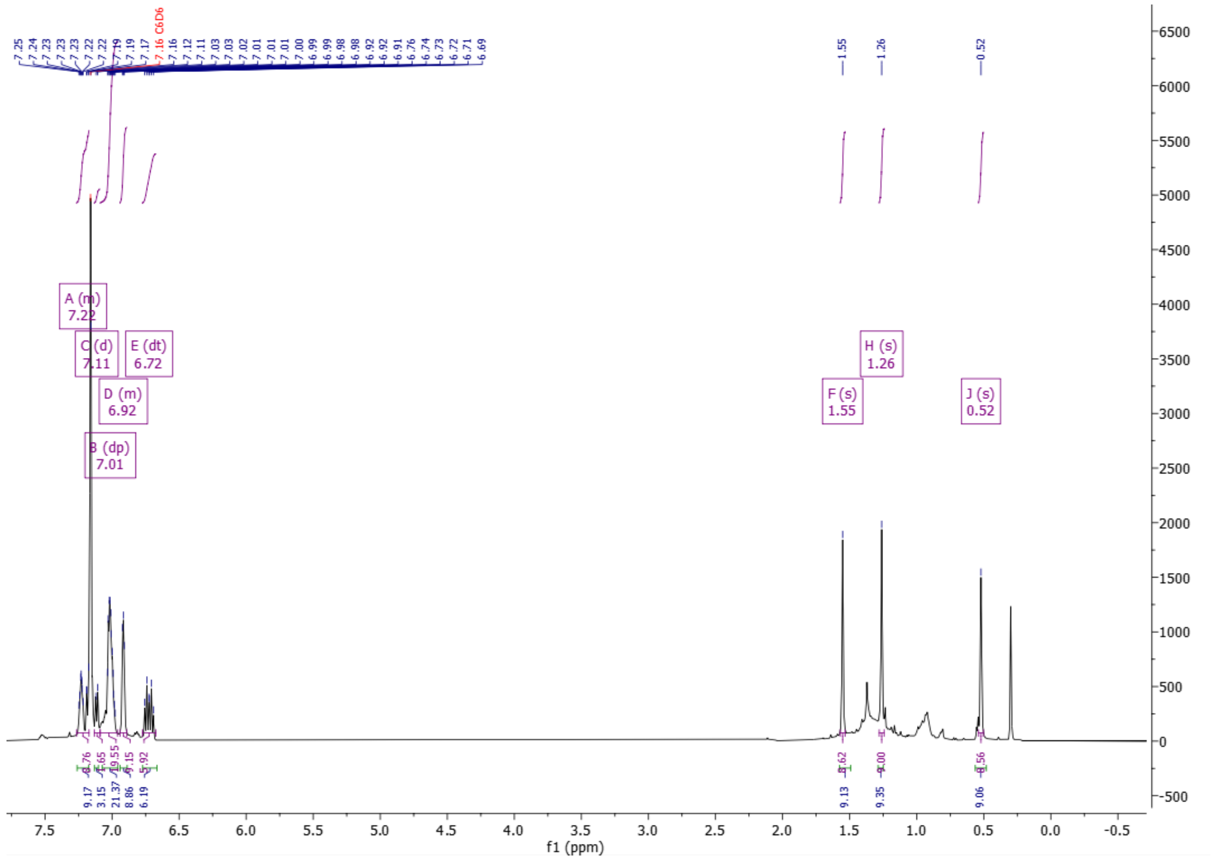

Figure S25: ^{1}H NMR spectrum of **10**

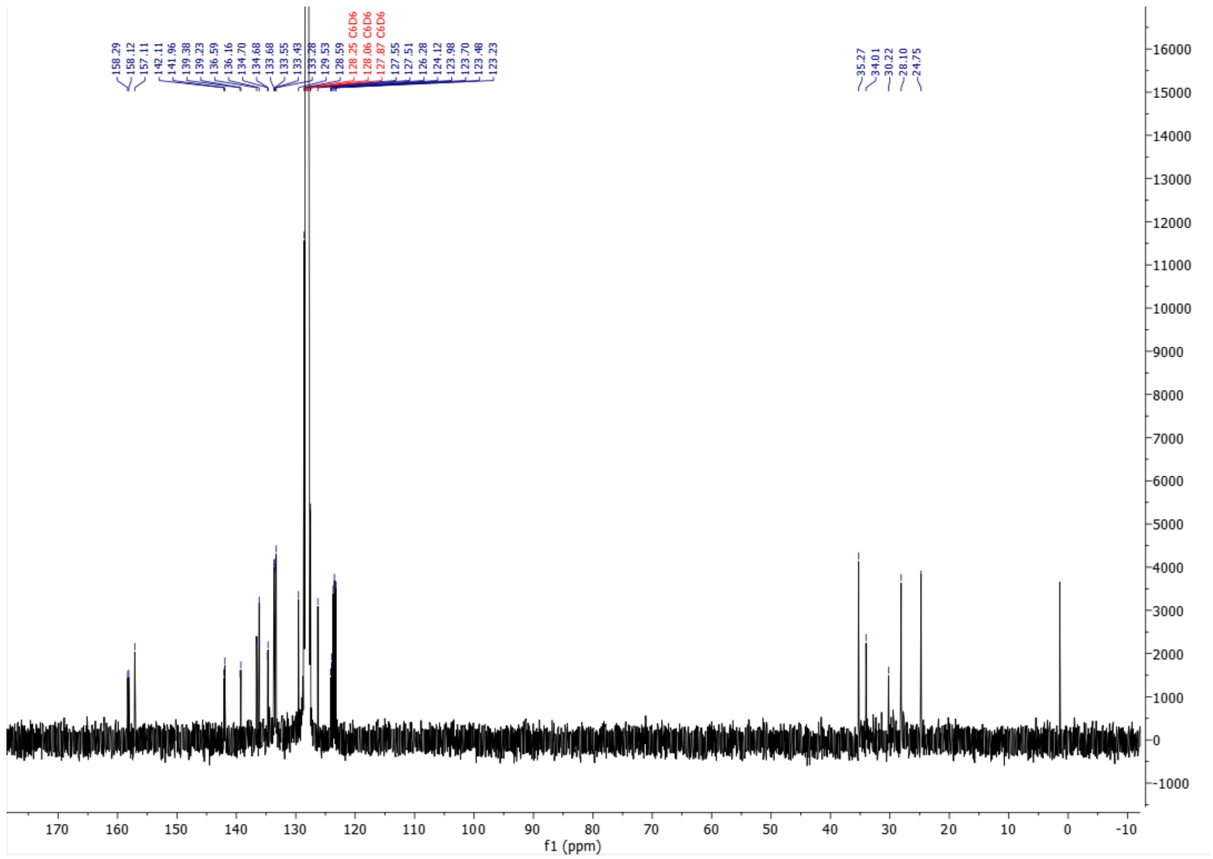

Figure S26: $^{13}C\{^{1}H\}$ NMR spectrum of **10**

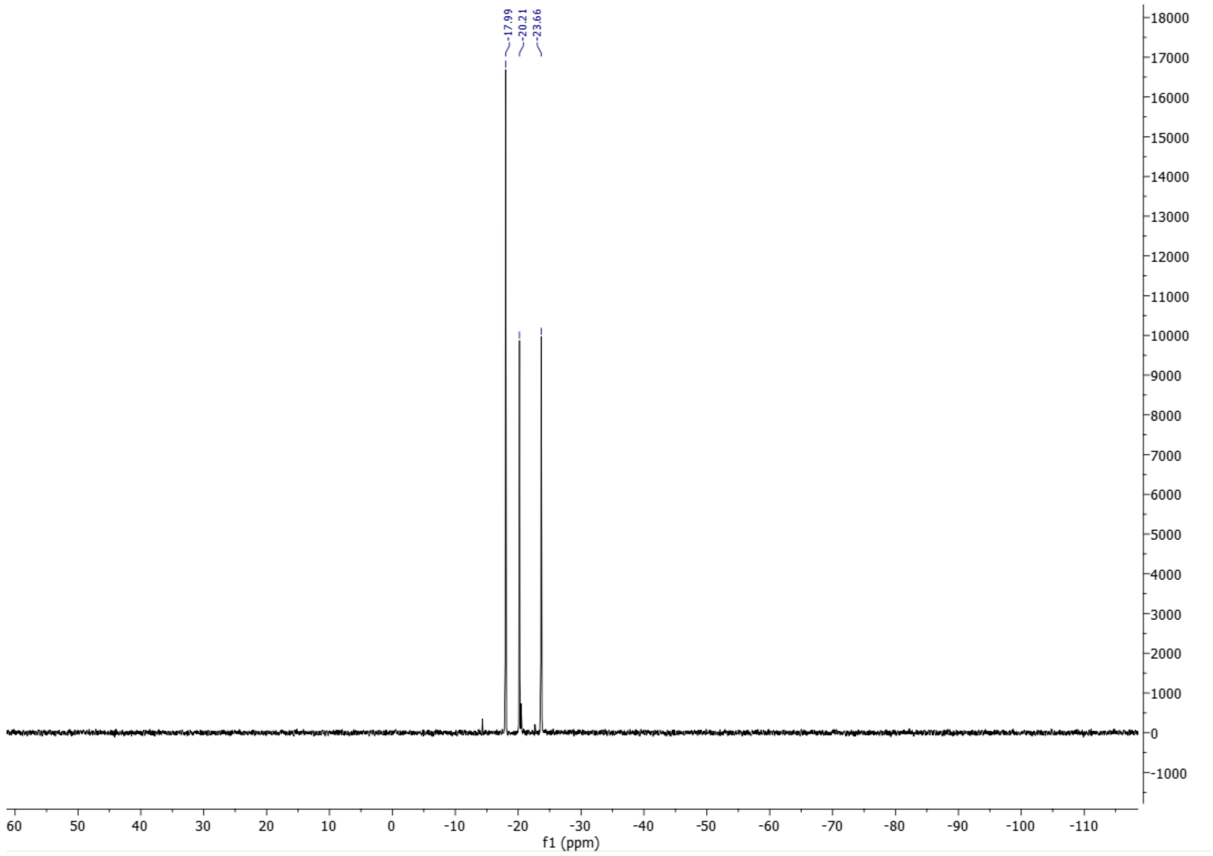

Figure S27: $^{31}P\{^{1}H\}$ NMR spectrum of **11**

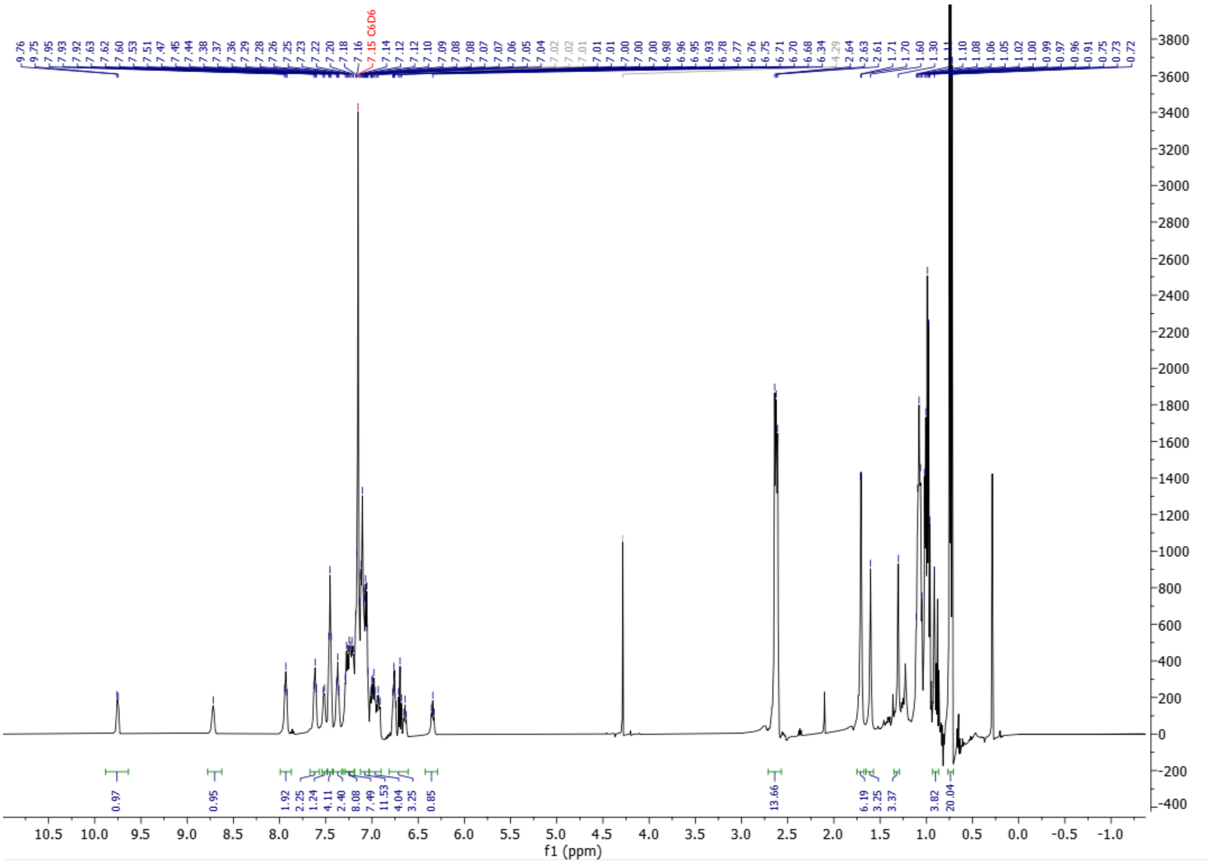

Figure S28: ^{1}H NMR spectrum of **11**

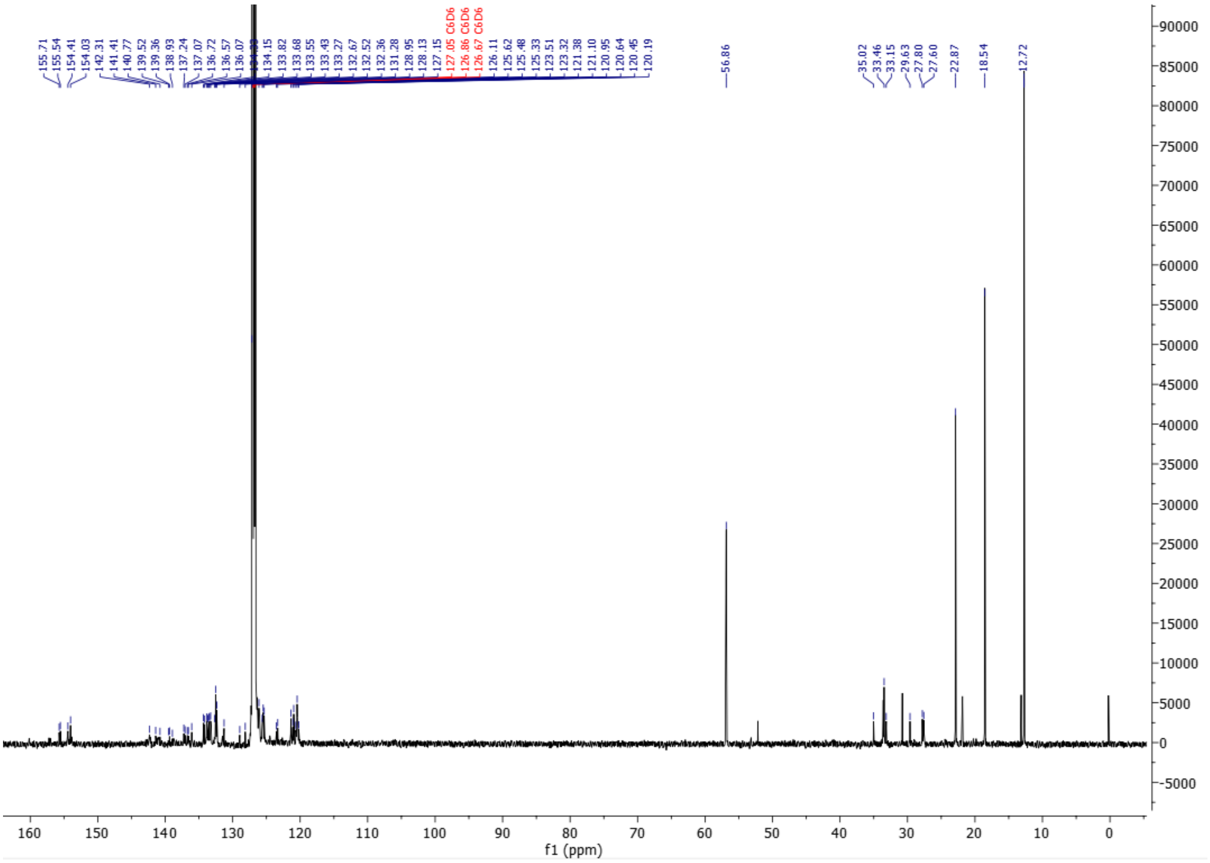

Figure S29: ^{13}C{^{1}H} NMR spectrum of **11**

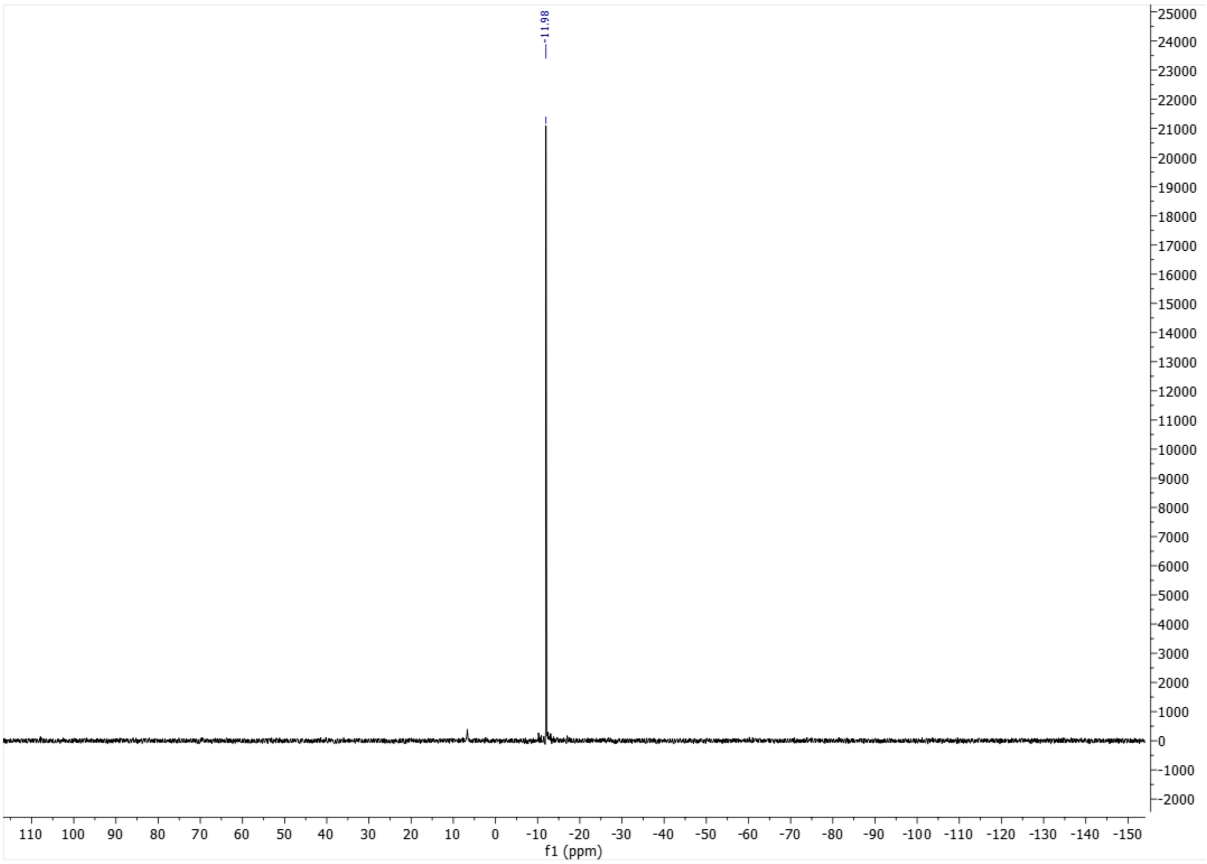

Figure S30: $^{31}P\{^{1}H\}$ NMR spectrum of **6**

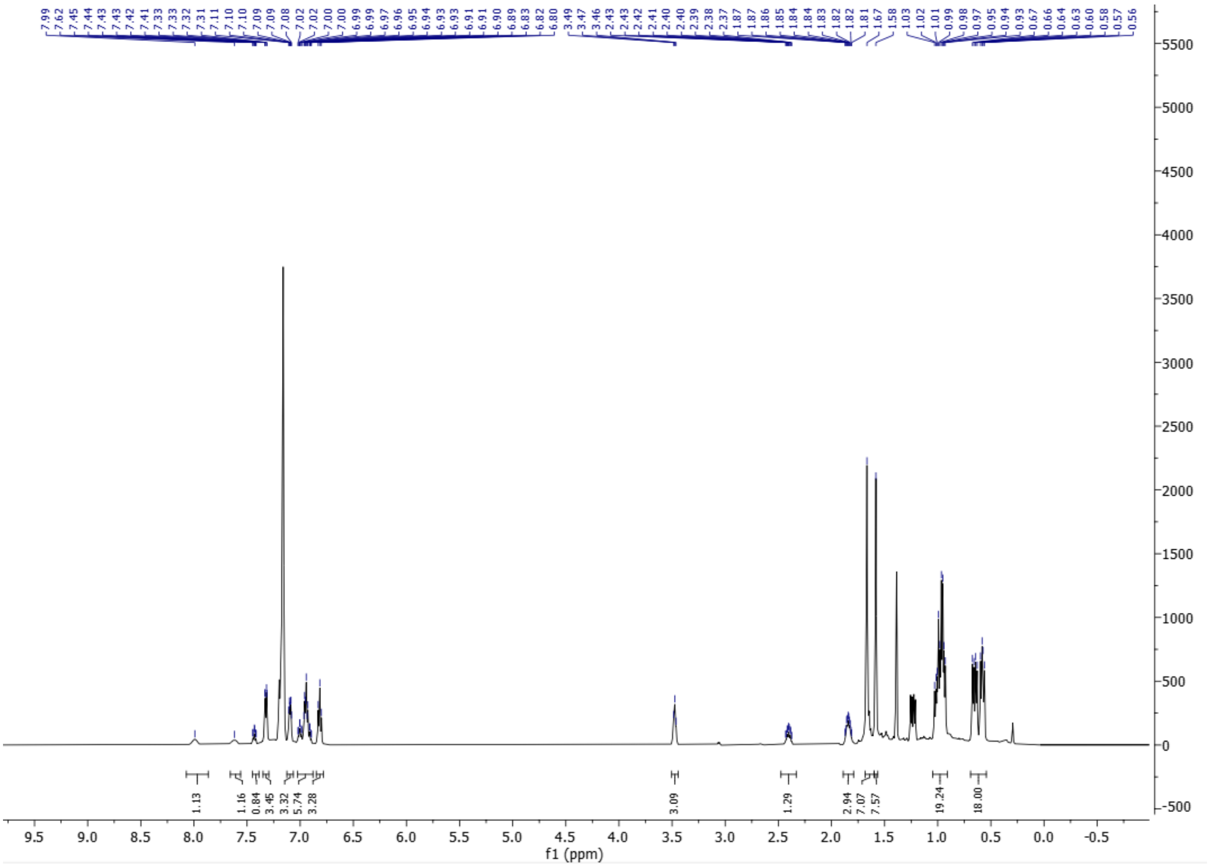

Figure S31: ^{1}H NMR spectrum of **6**

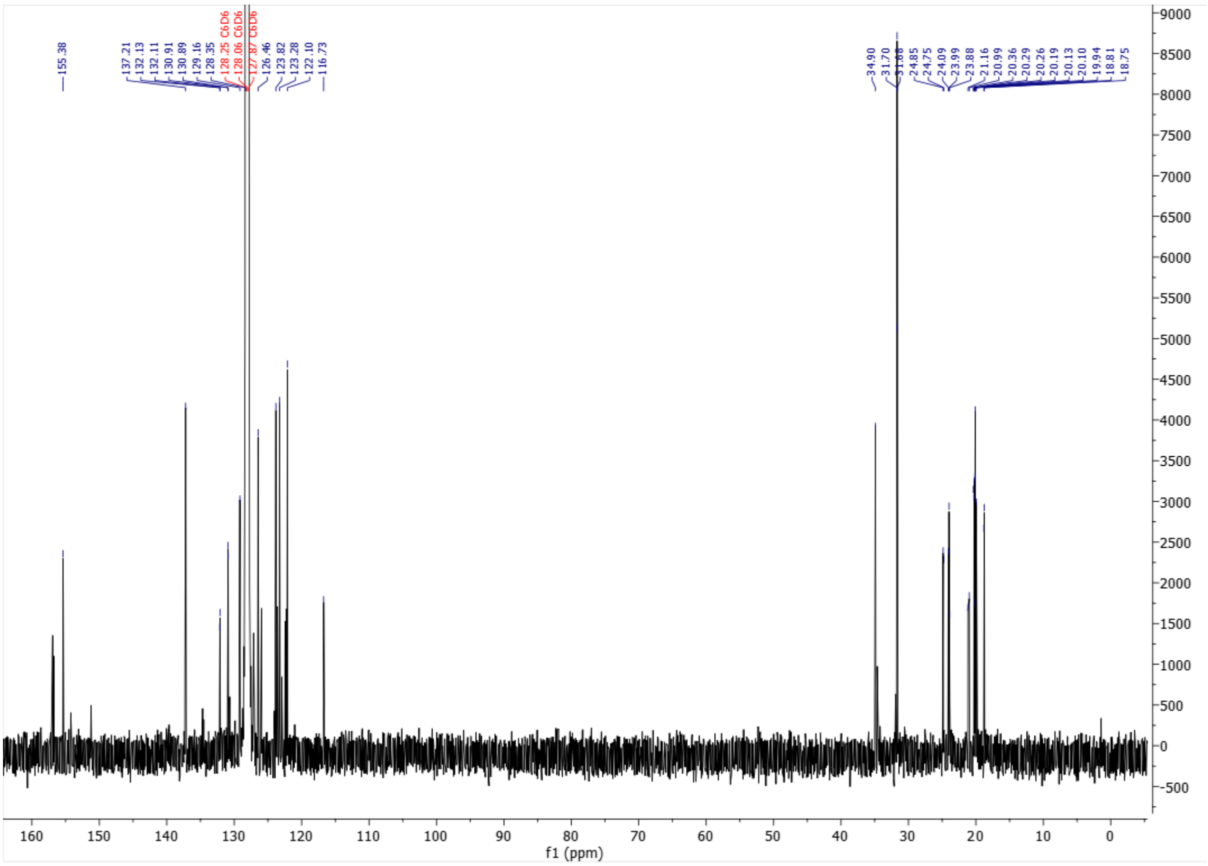

Figure S32: $^{13}C\{^{1}H\}$ NMR spectrum of **6**

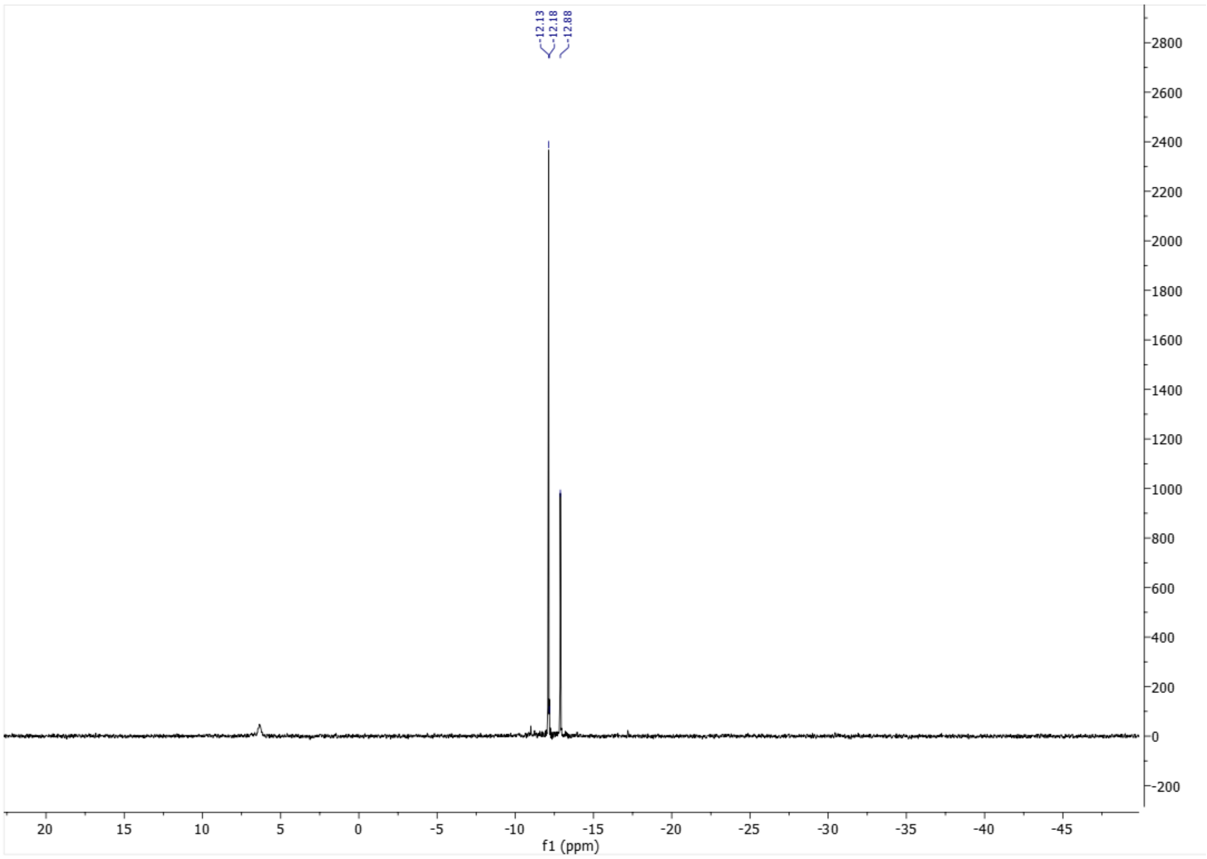

Figure S33: $^{31}P\{^{1}H\}$ NMR spectrum of **6**/**7**

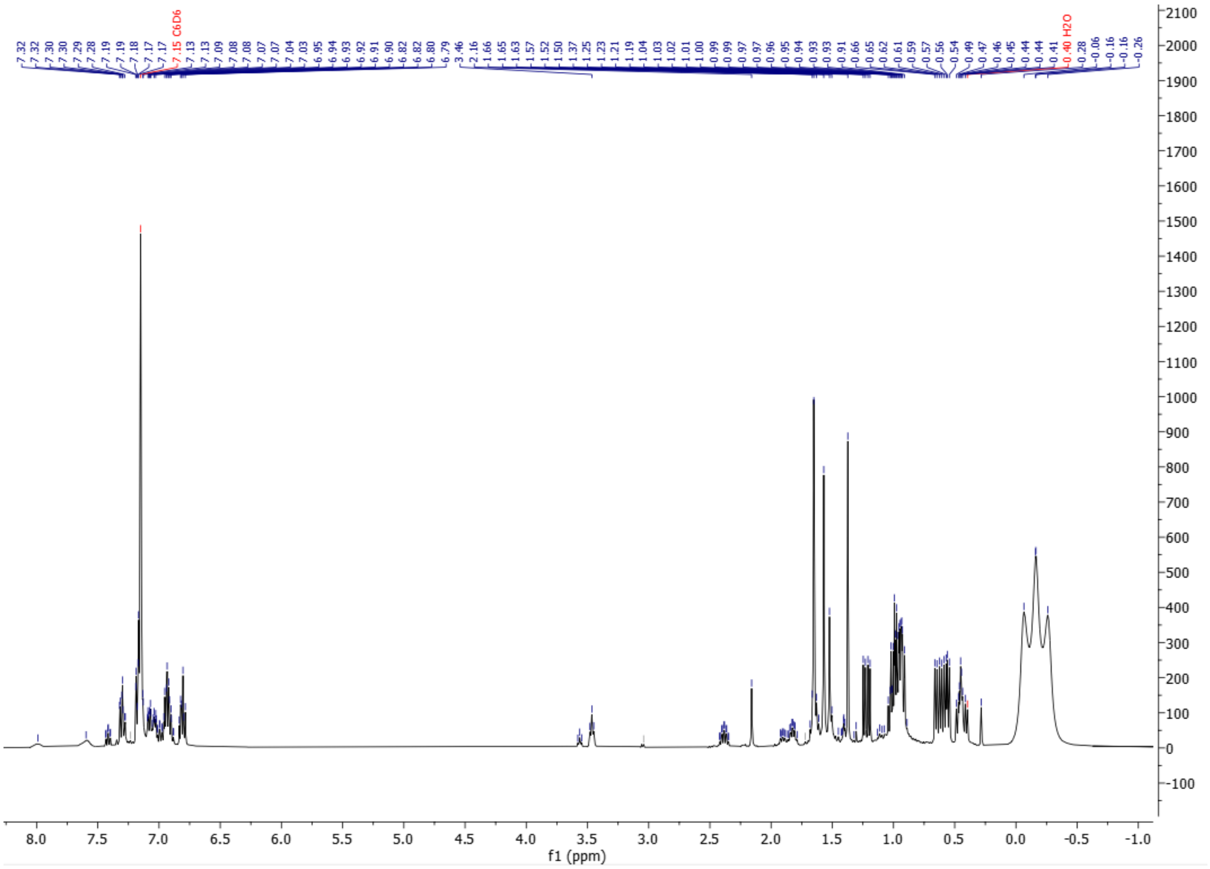

Figure S34: ^{1}H NMR spectrum of **6/7**

NMR studies of CO uptake by **2**

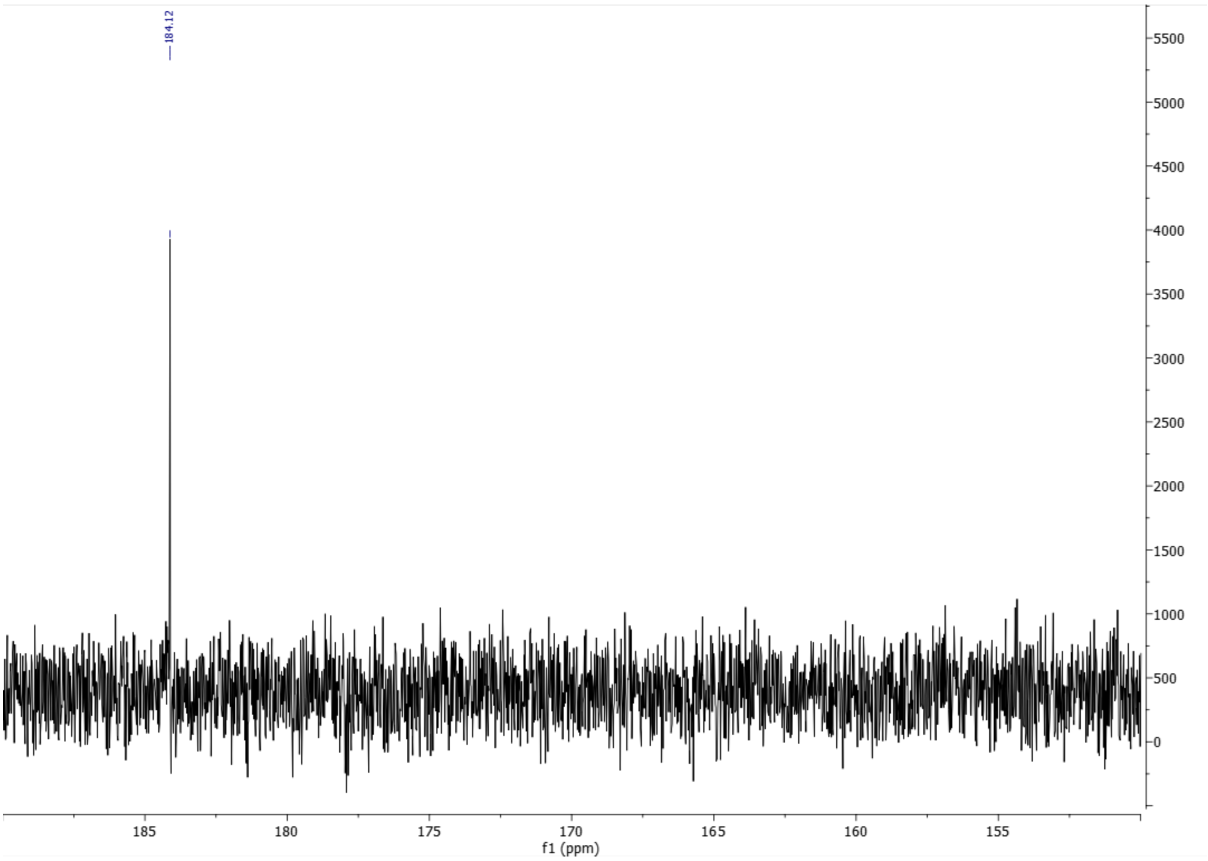

Figure S35 $^{13}C\{^{1}H\}$ NMR spectrum of CO in d_6-benzene using a 2 s relaxation delay (standard Bruker pulse sequence) after 3000 scans

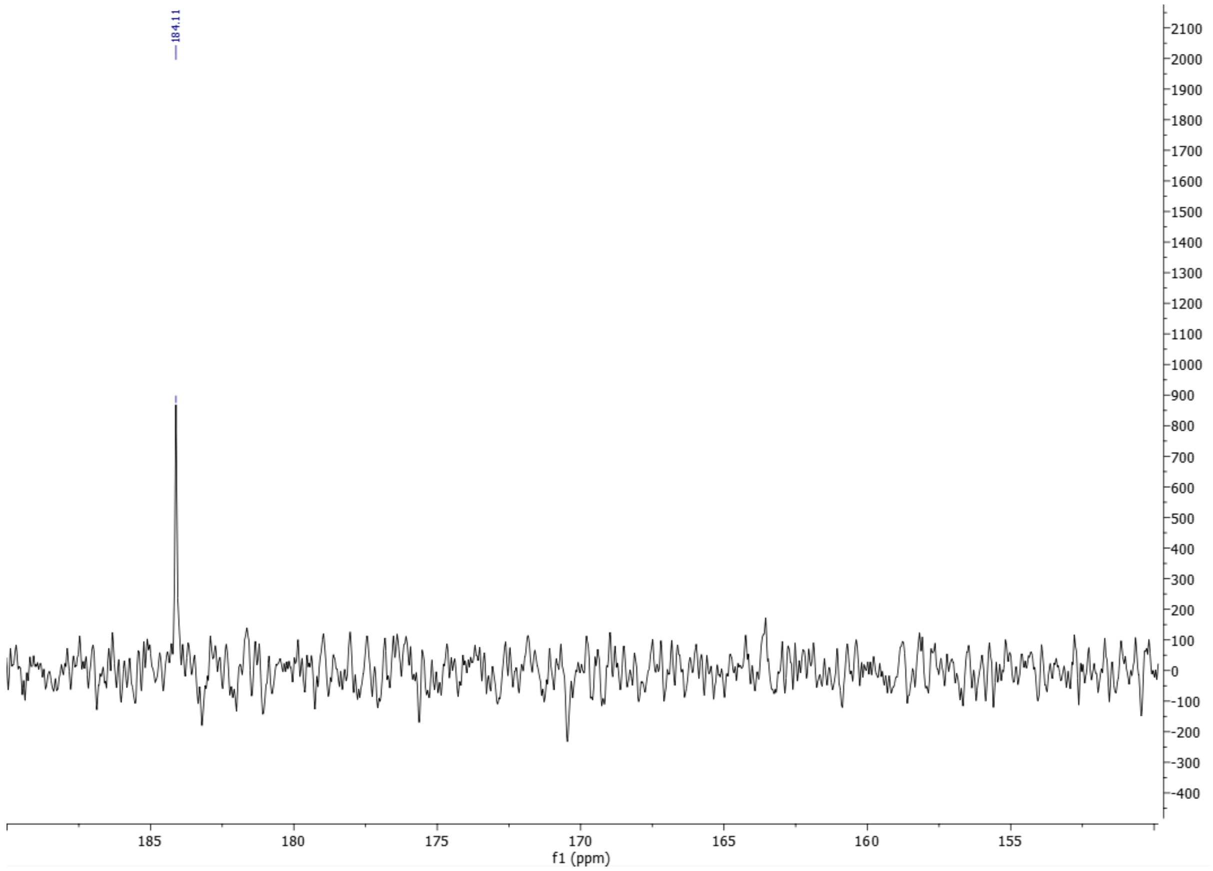

Figure S36: $^{13}C\{^{1}H\}$ NMR spectrum of CO in d_6-benzene using shortened 0.10 s delay after 3000 scans

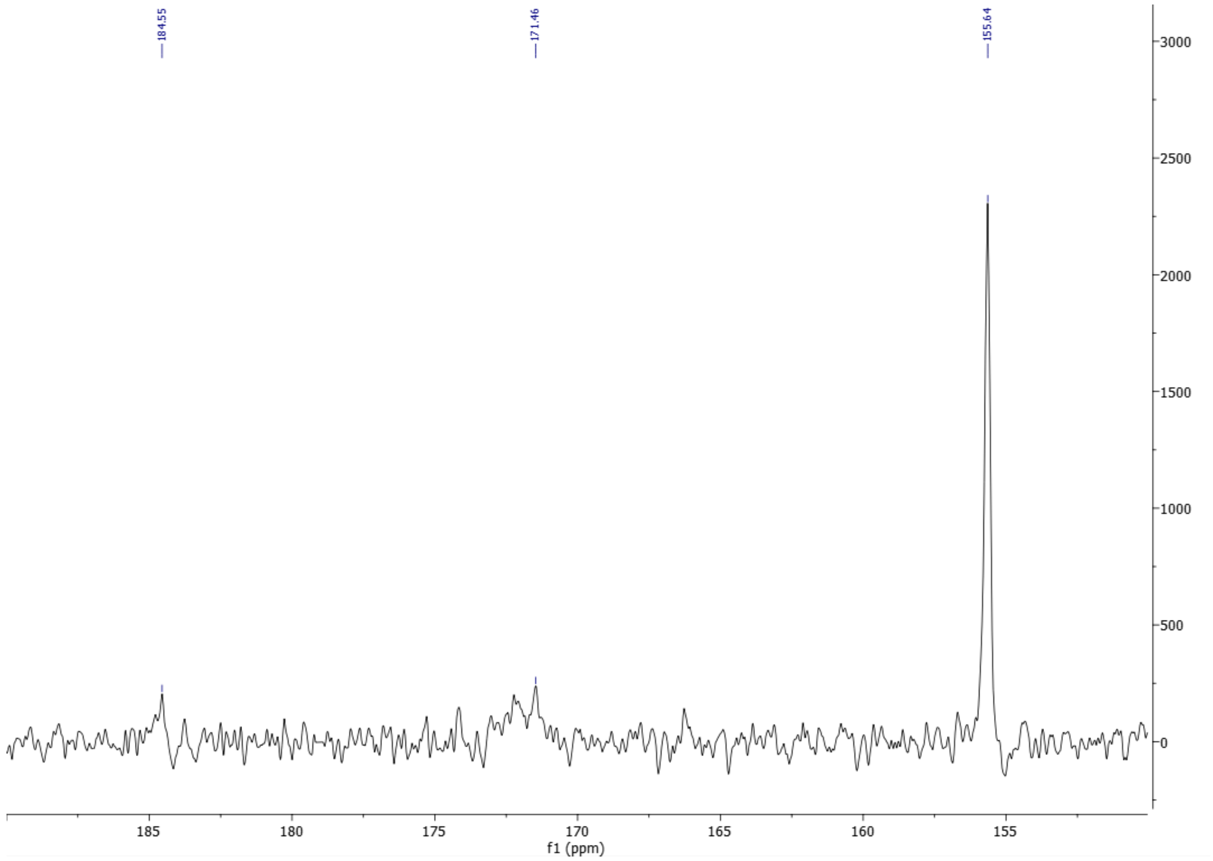

Figure S37: $^{13}C\{^{1}H\}$ NMR spectrum of **2** in d_6-benzene using a 2 s relaxation delay (standard Bruker pulse sequence) after 10,000 scans

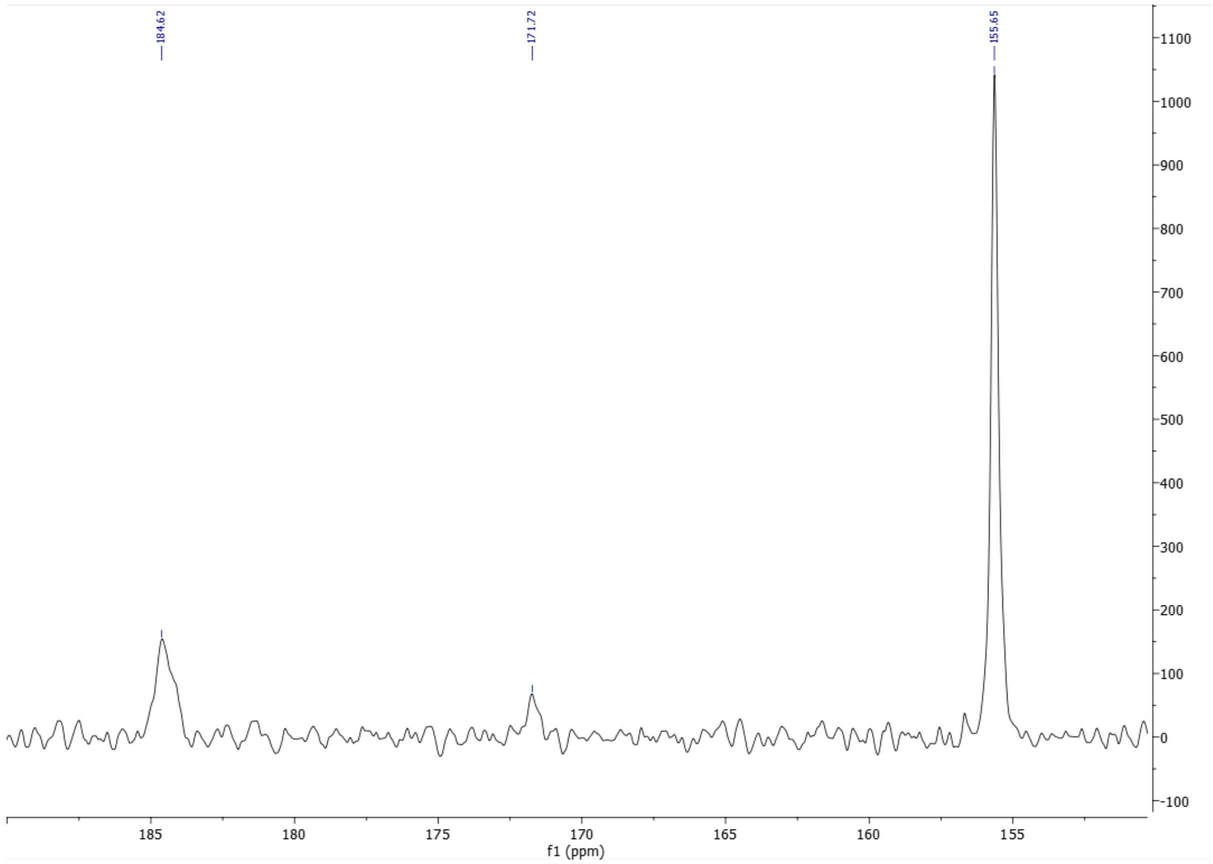

Figure S38: $^{13}C\{^1H\}$ NMR spectrum of **2** in d_6-benzene using shortened 0.10 s delay after 100,000 scans

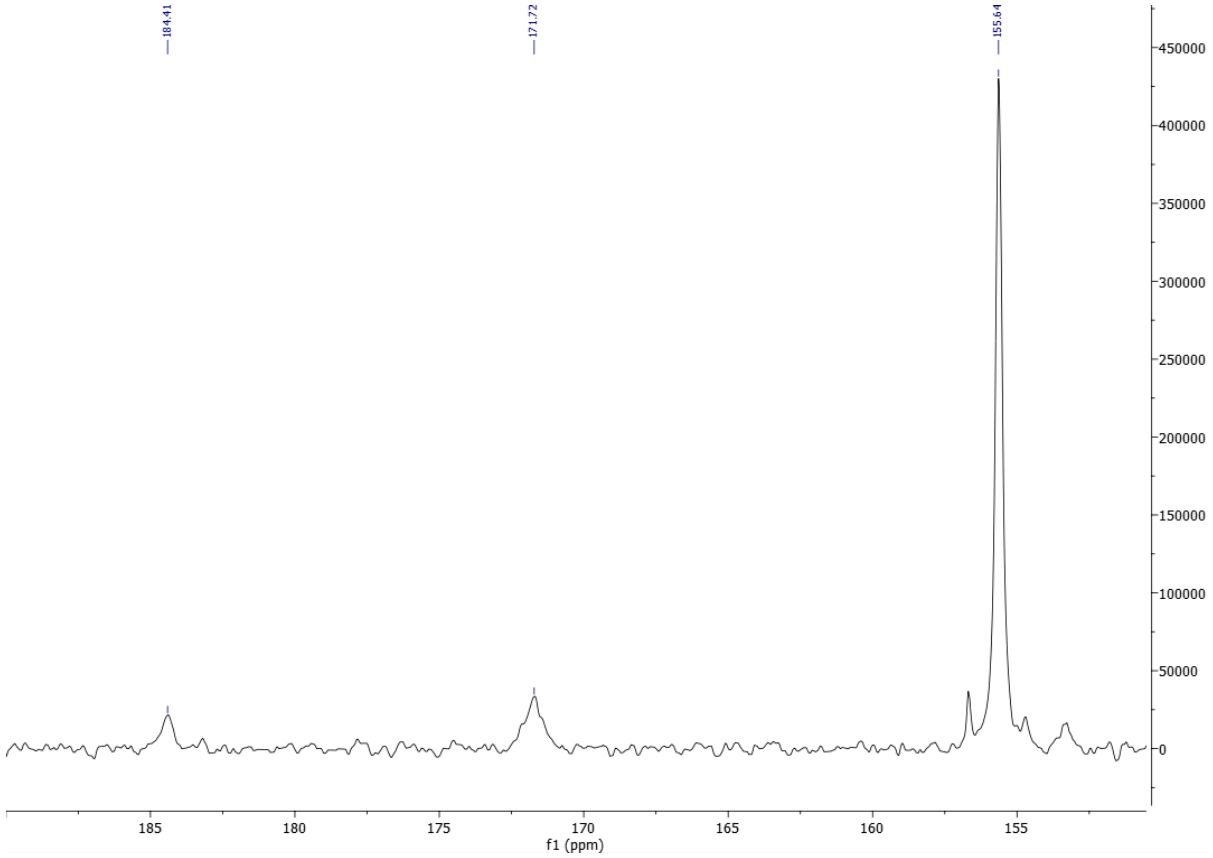

Figure S39: ^{13}C $\{^1H\}$ NMR spectrum of **2** dissolved in d_6-benzene using shortened 0.10 s delay after 250,000 scans with more concentrated sample (35 mg per 0.5 mL of d_6-benzene). Trace **3** can be seen around the resonance at 155.6 ppm.

In order to determine the position of the broad ^{13}C CO resonance of **8** we made use of the fact that CO is bound to quadrupolar $^{11/10}B$ nuclei and used a short T_1 experiment (0.16 s delay) in the region of interest. The standard Bruker ^{13}C experiment (2 s relaxation delay) does not yield a strong free CO or bound CO resonance after 10 h (Figure S37) however, using the shortened T_1 delay a resonance can be observed at 171.7 ppm with free CO also visible at 184.4 ppm after 100,000 scans (Figure S38). In order to obtain a better signal:noise ratio, the same region was probed on a more concentrated sample for 250,000 scans (Figure S39). The resonance at 171.7 ppm is clearly distinguishable from free CO, and is shifted upfield consistent with the CO IR stretch indicating a strengthening of the C-O bond.

VT NMR of CO uptake by **2**

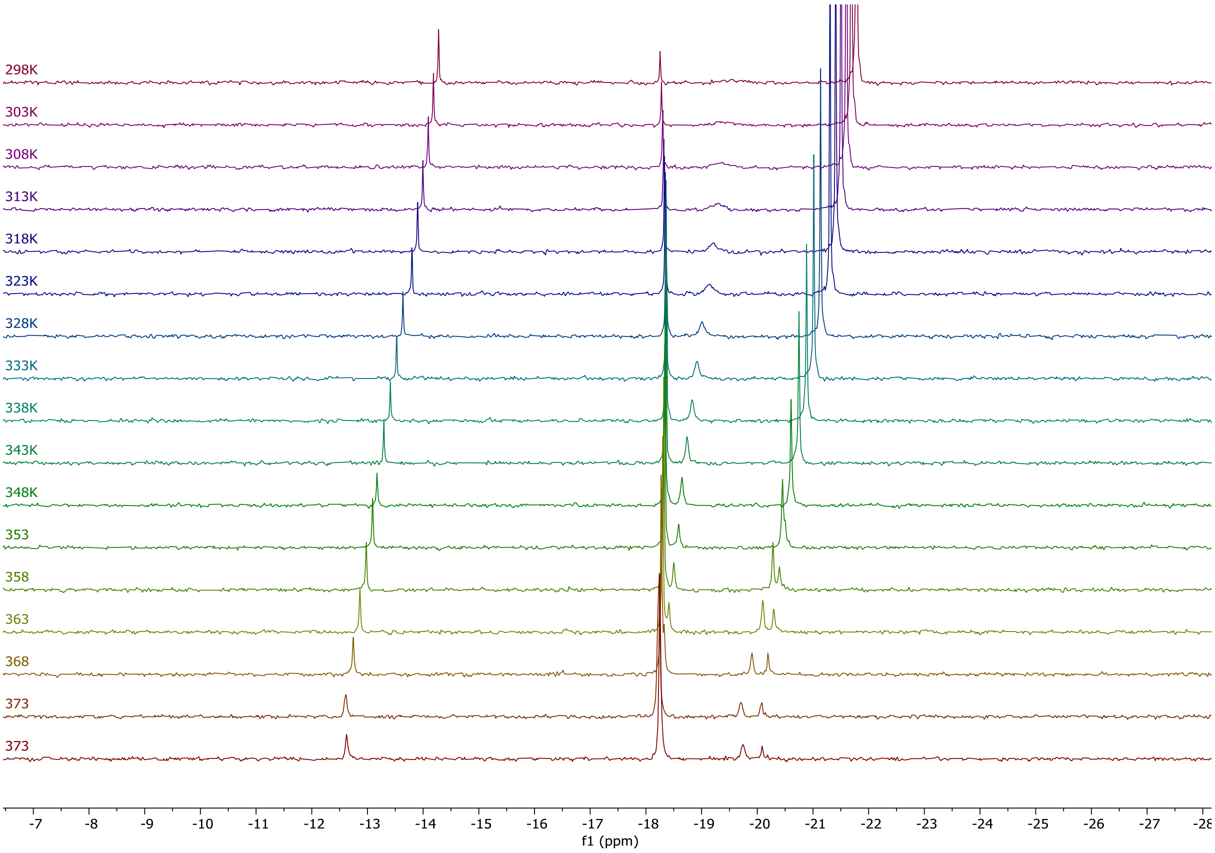

Figure S40: VT ^{31}P NMR spectra of CO adduct **8** at temperatures in the range 298-373K

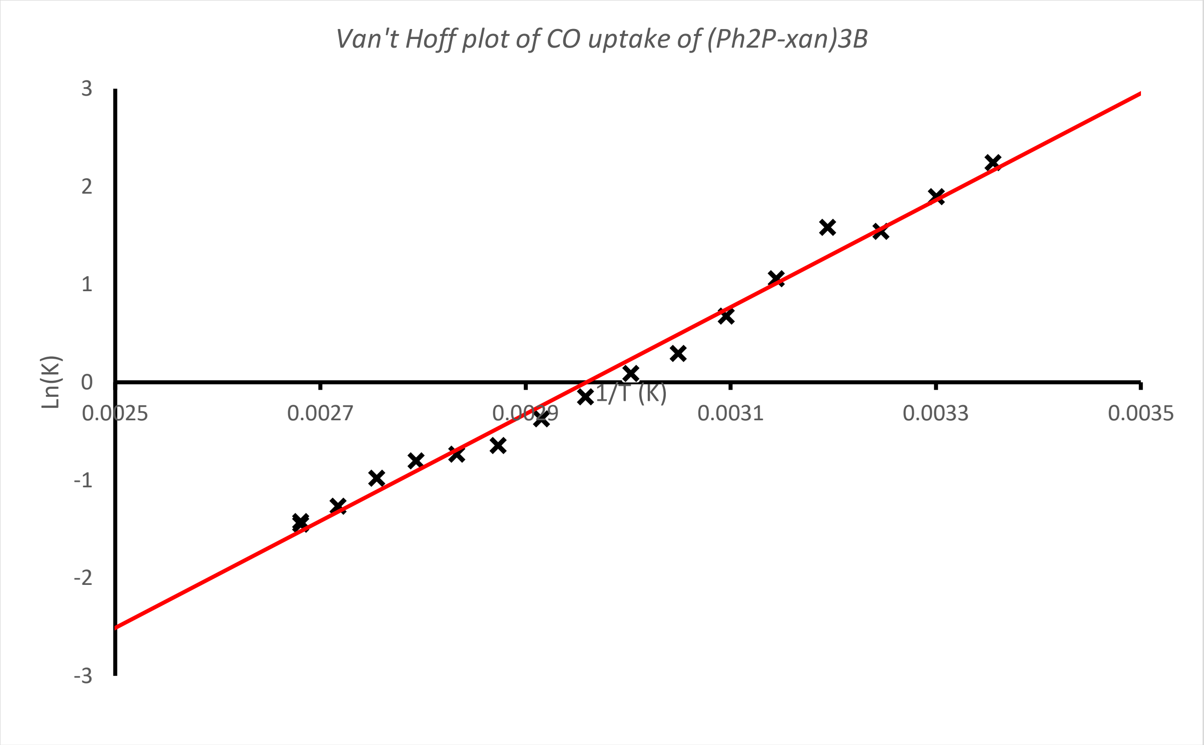

Figure S41: Van't Hoff plot for CO uptake by **2**, yielding ΔH = -10.9 kcal mol^{-1} and ΔS = -32.1 cal mol^{-1} K^{-1}

IR spectroscopy

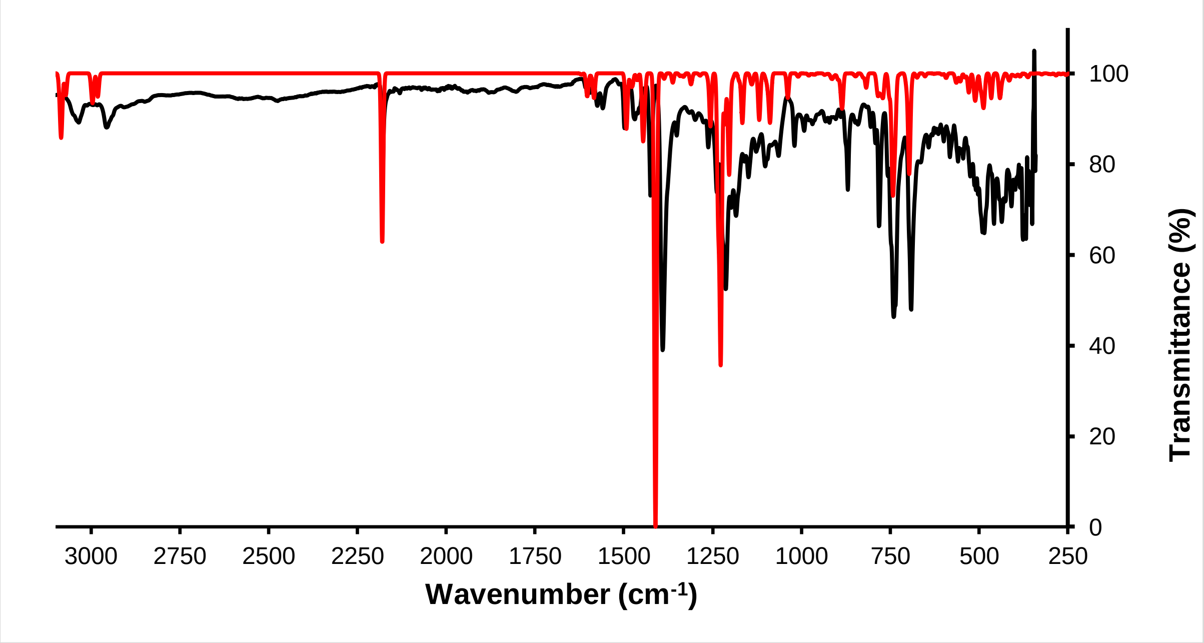

Figure S42: Measured FT-IR spectrum of **8** (black) and calculated spectra (red). R^{2}scan functional - details as set out below, 0.990 linear scale factor applied. C-O stretch at 2179 cm^{-1}.

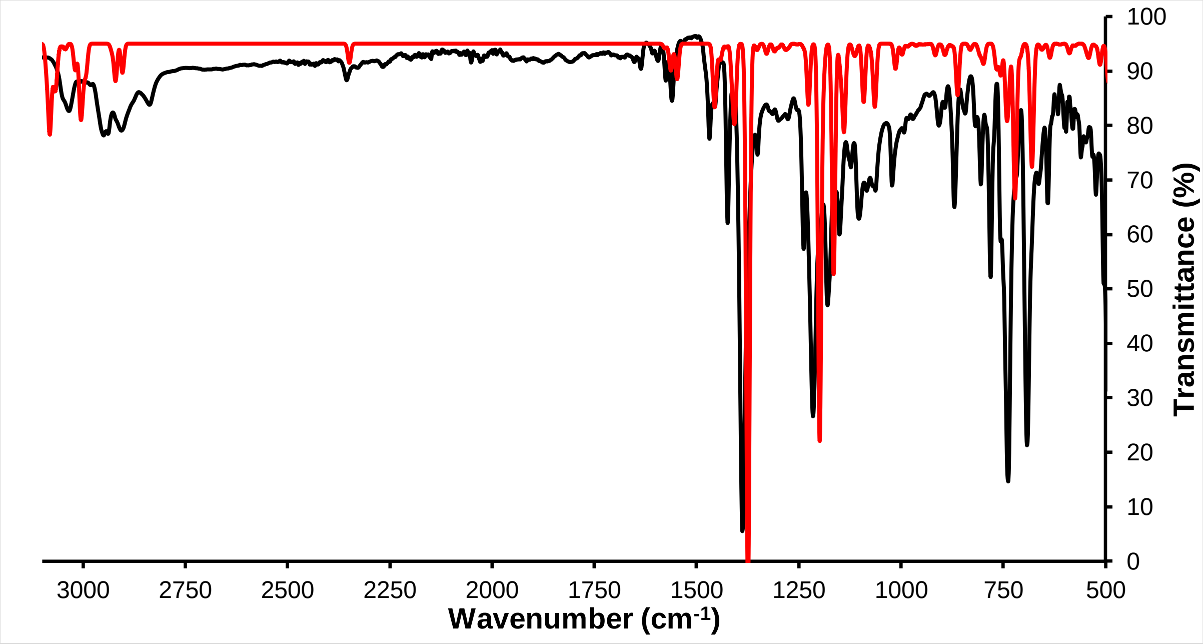

Figure S43: Measured FT-IR spectrum of **9** (black) and calculated spectrum (red). R^2scan functional – details as set out below, 0.975 linear scale factor applied. C-N stretch at 2356 cm^{-1}.

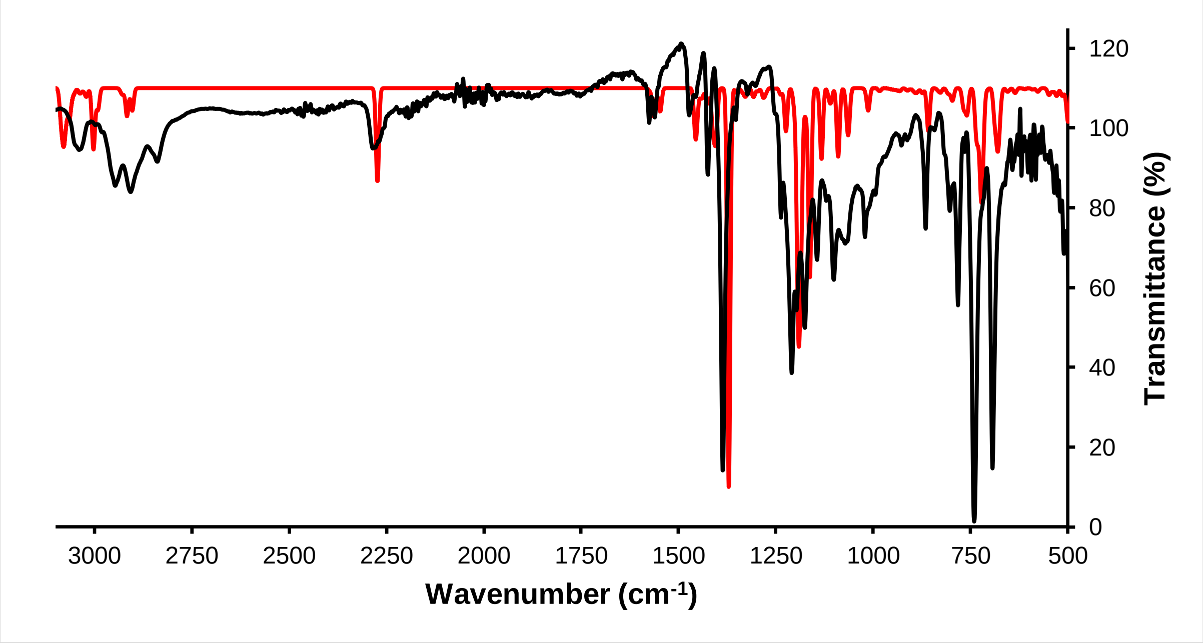

Figure S44: Measured FT-IR spectrum of **10** (black) and calculated spectrum (red). R^2scan functional - details as set out below, 0.965 linear scale factor applied. C-N stretch at 2285 cm^{-1}.

Crystallographic Data

Methods

All data was collected either on a Rigaku XtaLAB Synergy-DW VHF diffractometer equipped with a PhotonJet-R dual wavelength rotating anode and HyPix-Arc 150° detector or an Oxford Diffraction/Agilent SuperNova. Crystals were prepared with Paratone-N or perfluorinated oil, mounted on MiTeGen Micromount loops, and quench-cooled with an Oxford Cryosystems open flow N_2 cooling device.[4] Specific data collection details are in Table S1. Data processing involved CrysAlisPro[5] for unit cell refinement, SCALE3 ABSPACK inter-frame scaling, merging equivalent

reflections, and diffraction pattern processing. Structures were solved using SHELXT and refined with SHELXL in OLEX2.[6–8] Crystallographic data is available in supplementary CIF files available free of charge via the Cambridge Crystallographic Data Centre (CCDC): http://www.ccdc.cam.ac.uk/data_request/cif. CCDC deposition codes are listed in Table S1.

Table S1: X-ray crystallography

	1	2	3	4	5	6	7	8	9	10	11
chemical formula	$C_{95}H_{83}BLiO_3P_3$	$C_{81}H_{66}BO_3P_3$	$C_{81}H_{68}BO_4P_3$	$C_{82}H_{71}BNO_3P_3$	$C_{93}H_{81}BNO_3P_3$	$C_{64}H_{83}BNO_3P_3$	$C_{66}H_{84}BNO_3P_3$	$C_{82}H_{66}BO_4P_3$	$C_{83}H_{69}BNO_3P_3$	$C_{86}H_{75}BNO_3P_3$	$C_{98}H_{102}BN_2O_3P_3$
Molecular weight	1383.27	1191.05	1209.07	1222.11	1364.3	1018.03	1043.06	1219.06	1232.11	1274.19	1459.53
Space group system	triclinic	triclinic	monoclinic	triclinic	triclinic	monoclinic	monoclinic	triclinic	triclinic	trigonal	monoclinic
Space group (Hall)	P -1	P -1	P 1 21/n 1	P -1	P -1	P 1 21/n 1	C 1 2/c 1	P -1	P -1	P 3 1 c	P 1 21 1
a	12.41167(6)	15.1342(4)	12.08526(4)	10.97770(10)	12.1687(2)	11.55435(4)	41.6461(8)	11.90675(7)	11.2305(2)	19.1556(4)	12.53360(10)
b	12.60674(7)	15.2088(4)	15.08018(5)	14.3258(2)	13.4038(3)	49.6560(2)	11.7710(2)	13.32636(10)	14.9413(2)	19.1556(4)	21.26540(10)
c	23.43695(12)	15.7792(4)	38.06644(12)	21.8808(3)	24.8061(6)	20.45834(8)	24.2165(5)	43.7452(2)	21.0548(2)	28.8526(5)	15.29760(10)
α	95.7034(4)	101.097(2)	90	81.6380(10)	85.415(2)	90	90	82.2865(5)	92.3870(10)	90	90
β	92.7202(4)	102.429(2)	94.2325(3)	77.8480(10)	85.545(2)	99.5935(4)	90.411(2)	89.6844(5)	94.8310(10)	90	93.4700(10)
γ	93.8046(4)	94.734(2)	90	70.2100(10)	64.878(2)	90	90	69.1539(6)	109.9740(10)	120	90
V	3635.60(3)	3451.27(16)	6918.61(4)	3154.99(7)	3647.30(15)	11573.66(8)	11871.0(4)	6421.44(8)	3299.28(8)	9168.7(4)	4069.82(5)
Z	2	2	4	2	2	8	8	4	2	4	2
T (K)	100.00(10)	150.00(16)	100.00(10)	100.00(10)	150.00(10)	100.01(10)	150.01(10)	100.00(10)	99.99(10)	100.00(10)	100.00(10)
Unique reflections	129526	14826	199490	54774	25340	167096	17235	122492	39694	19949	66623
Collected reflections	163951	37317	338660	139362	63406	400055	43339	293614	84732	71726	120883
Mu	1.166	1.154	1.169	1.28	1.161	1.284	1.264	1.265	1.229	0.896	1.072
Radiation	Cu K\a	Cu K\a	Cu K\a	Cu K\a	Cu K\a	Cu K\a	Cu K\a	Cu K\a	Cu K\a	CuK\a	Cu K\a
λ	1.54184	1.54184	1.54184	1.54184	1.54184	1.54184	1.54184	1.54184	1.54184	1.54184	1.54184
Parameters	1002	799	890	826	972	1351	659	1946	870	568	975
R_{all}	0.0326	0.0516	0.0394	0.0497	0.0523	0.0418	0.0473	0.0623	0.0501	0.0783	0.0407
R_{gt}	0.0321	0.0402	0.0356	0.0441	0.0384	0.0365	0.0387	0.0554	0.0443	0.0612	0.0388
wR	0.0821	0.1058	0.0991	0.1231	0.1011	0.0989	0.1043	0.1456	0.1236	0.1735	0.1009
R_{int}	0.0229	0.0413	0.035	0.0333	0.043	0.0362	0.0331	0.0368	0.0435	0.0406	0.0835
goof	1.038	1.041	1.028	1.032	1.012	1.022	1.03	1.058	1.028	0.996	1.024
Local code	157aec23	134aeccm21	156aec23	153aec23	123aec23	163aec23	194aec23	280aec23	332aec23	301aec23	330aec23
CCDC deposition number	2355676	2355678	2355732	2355736	2355737	2356128	2355738	2355747	2355748	2355751	2355757

DFT

General procedure

All calculations performing with the ORCA (Revision 5.0.4) program.[9–11] All compounds where first optimised using the meta-generalized-gradient approximation (mGGA) functional R2-SCAN[12] in conjunction with the Def2-TZVPPm basis set[13] with the D4 dispersion correction[14], and employing the geometrical counterpoise correction gCP[15] (together known as the R^{2}SCAN-3c method)[13]. Frequency calculations were performed to confirm the nature of minima by the lack of imaginary frequencies. Frequency calculations were also used to generate simulated IR spectra using the Avogadro visualiser.[16] Single point calculations where then performed using the ωB97x range separated hybrid functional[17,18] with the D4 dispersion correction alongside the Def2-TZVP basis set[19,20] and solvation of benzene with the conductor-like polarizable continuum model, CPCM for Benzene.[21]

Weak bonding analysis

Bonding analysis was performed on the wavefunctions using the Multiwfn(QTAIM/NCI) and NBO7 programmes.[22–24] In order to visualise the weak interactions causing the assembly of the cage conformation and its ability to accept molecules into the pocket, a non-covalent indicator [25] (NCI) surface coupled with the topological bond critical paths and bond critical points (BCP) from the atoms and molecules framework (QTAIM) [26] was used.

Analysis of the NCI surface reveals why the cage conformation is favoured in both the ^{i}Pr and Ph systems (by ΔG = -11.9 and -32.7 kcal mol^{-1}, respectively): increased Van der Waal regions, shown by the green isosurfaces, are in evidence between the xanthene backbone groups compared to the open conformation. Supporting this analysis, the increased presence of bond critical paths linking the adjacent xanthene backbones indicates more regions of intramolecular interactions. This difference can be seen to be less prevalent for the ^{i}Pr cage with the open conformation still maintaining a significant number of intramolecular interaction regions.

Blue regions on the NCI surface can be seen in the case of the protic bond systems (NH_3, H_2O and $MeNH_2$) which are indicative of hydrogen bonding. [27] Second order perturbation theory (SOPT) within NBO also identified these hydrogen bonding interactions. For the CO complex a shroud of Van der Waal interactions surrounding the guest molecule is found, with bond critical points indicating the importance of weak forces in the formation of these complexes.

Table S2: NBO Second order perturbation theory values for the EH···P interaction in kcal mol^{-1}. EH···P (1) is the first hydrogen bond, EH···P (2) is the second hydrogen bond, total is the sum of the hydrogen bonds to phosphorus

	**3**	**5**	**4**	**7**	**6**
EH···P (1)	22.48	7.37	1.3	18.56	14.89
EH···P (2)		5.61	8.84	12.52	18.79
EH···P (3)				8.42	
total	22.48	12.98	10.14	39.5	33.68

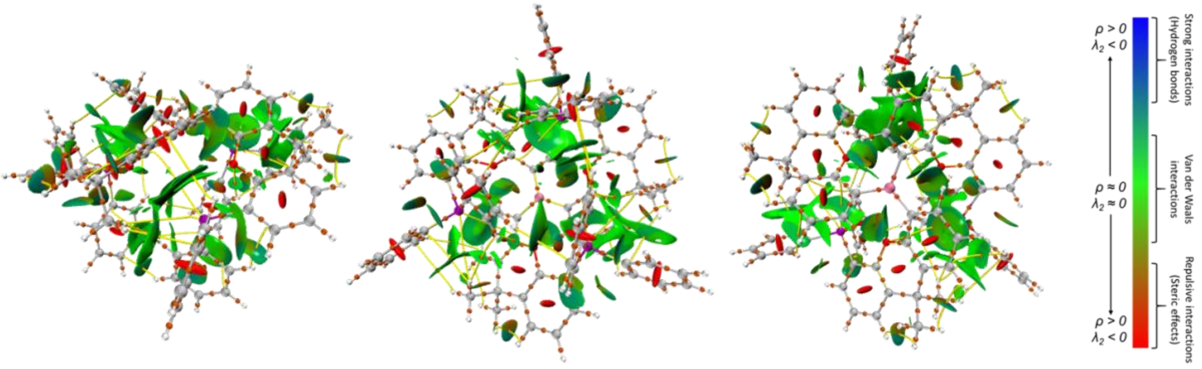

Figure S45: NCI plot of **2** in cage (*up,up,up*) conformation with the isosurface set at 0.5 a.u, and BGR from -0.03 to 0.02. QTAIM bond paths are shown in yellow and BCPs in orange.

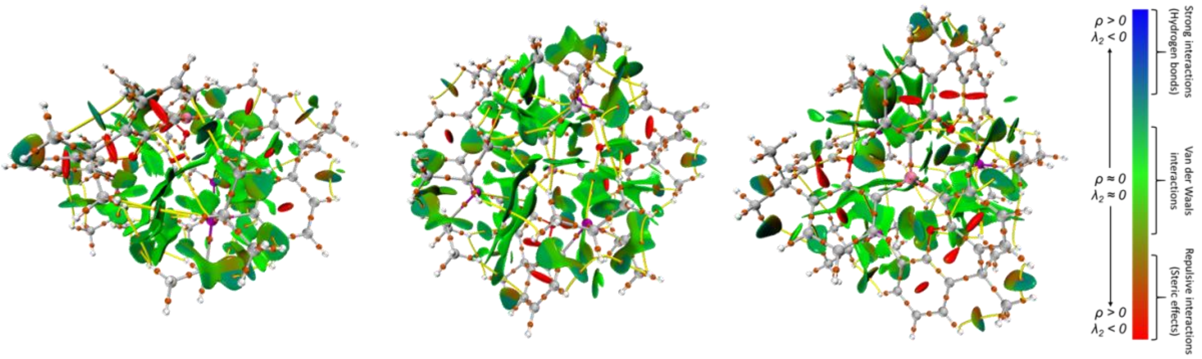

Figure S46: NCI plot of (^{i}Pr2P-xan)$_3$B in cage (*up,up,up*) conformation with the isosurface set at 0.5 a.u, and BGR from -0.03 to 0.02. QTAIM bond paths are shown in yellow and BCPs in orange.

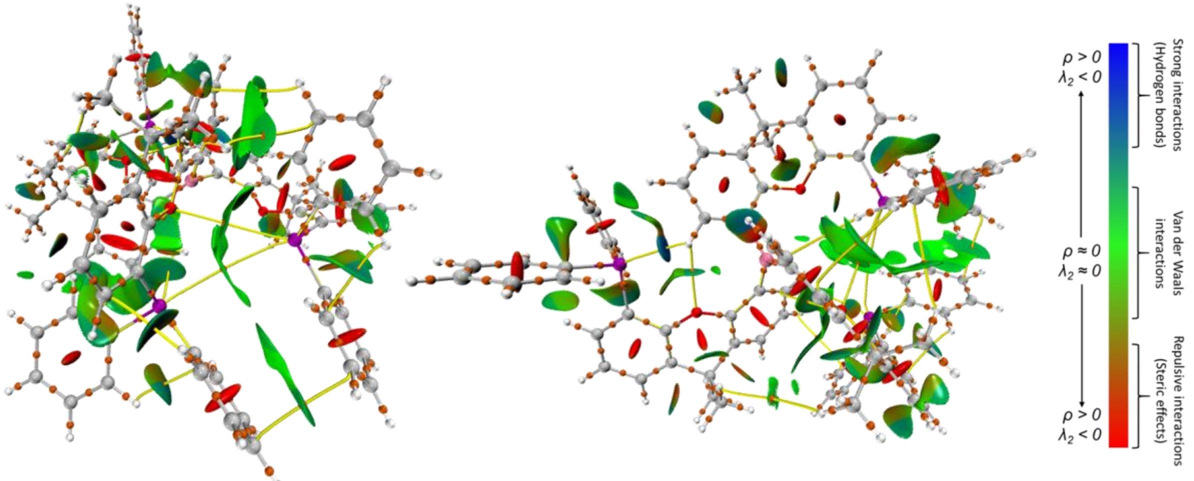

Figure S47: NCI plot of **2** in open (*up,up,down*) conformation with the isosurface set at 0.5 a.u, and BGR from -0.03 to 0.02. QTAIM bond paths are shown in yellow and BCPs in orange.

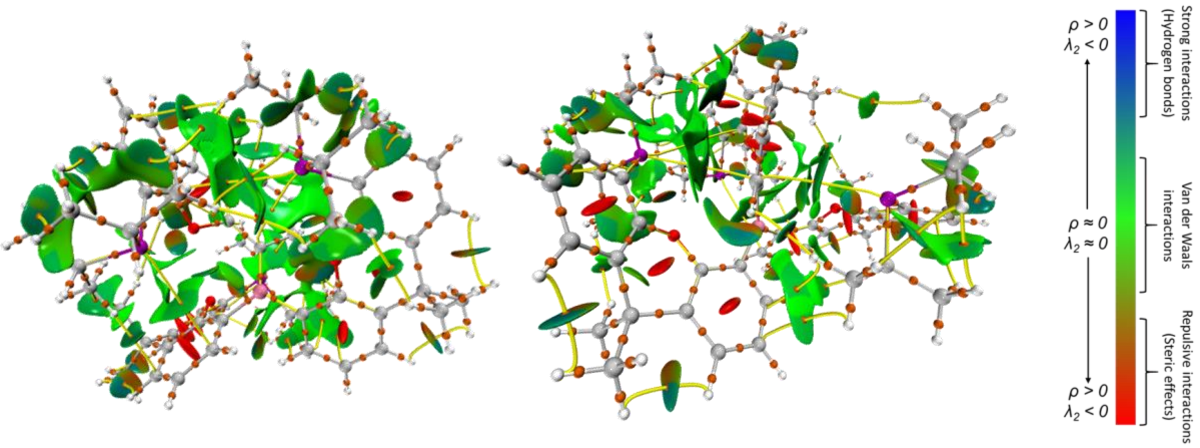

Figure S48: NCI plot of ($^{i}Pr_2P$-xan)$_3$B in open (*up,up,down*) conformation with the isosurface set at 0.5 a.u, and BGR from -0.03 to 0.02. QTAIM bond paths are shown in yellow and BCPs in orange.

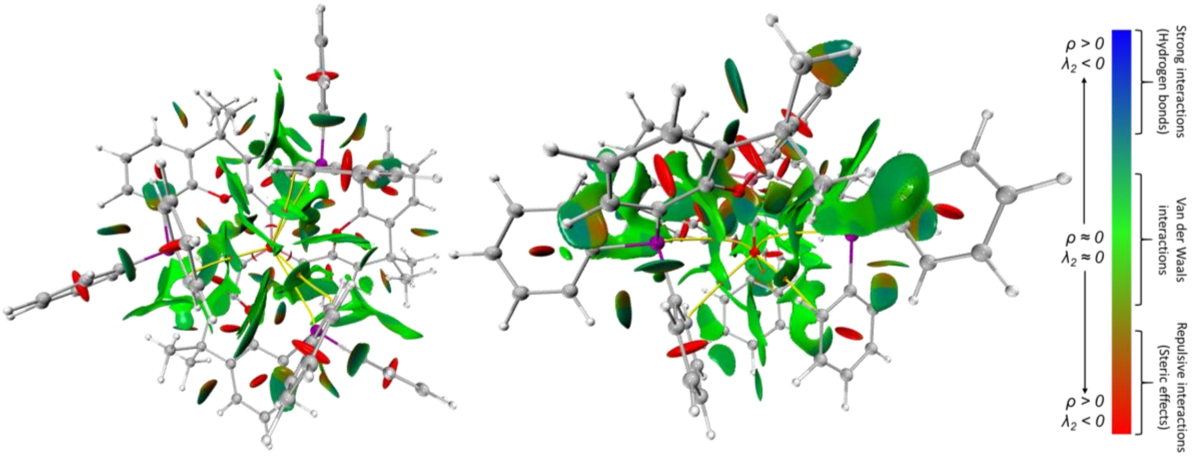

Figure S49: NCI plot of complex **8** with the isosurface set at 0.5 a.u, and BGR from -0.03 to 0.02. QTAIM bond paths are shown in yellow and BCPs in orange.

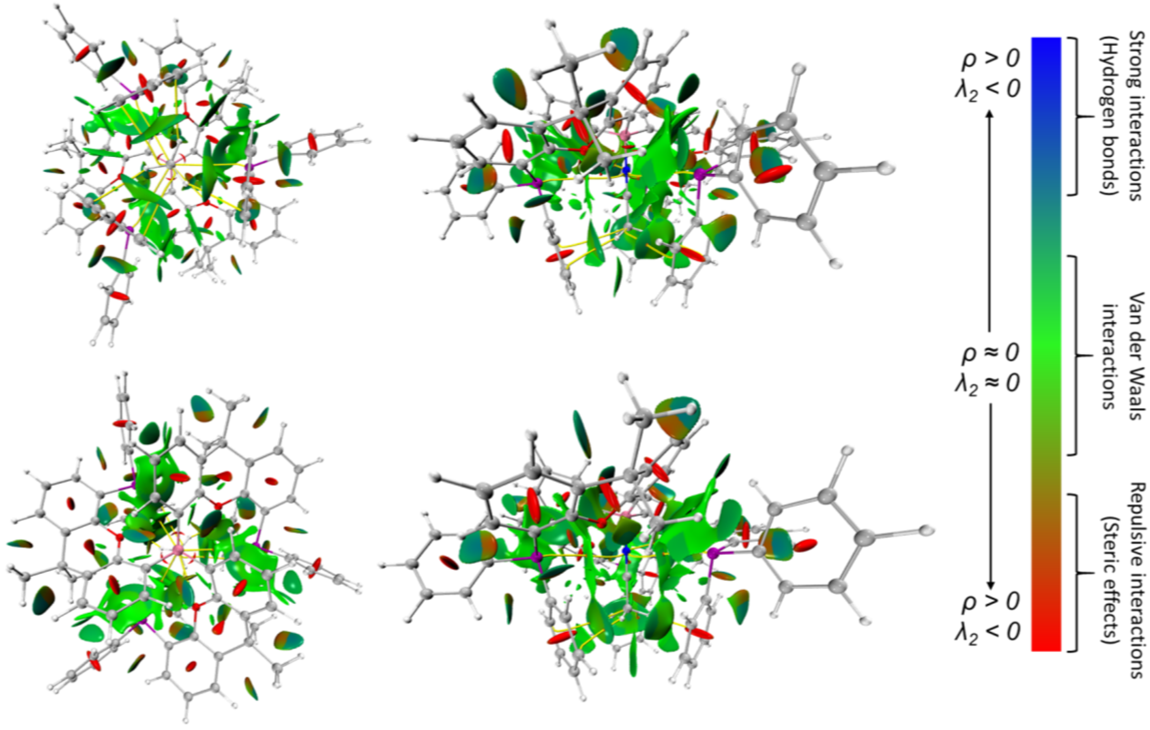

Figure S50: NCI of complex **9** with the isosurface set at 0.5 a.u, and BGR from -0.03 to 0.02. QTAIM bond paths are shown in yellow and BCPs in orange.

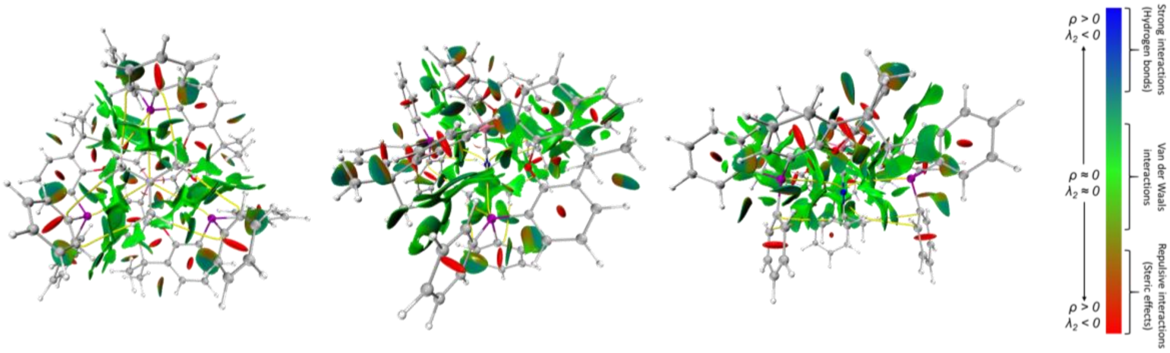

Figure S51: NCI of complex **10** with the isosurface set at 0.5 a.u, and BGR from -0.03 to 0.02. QTAIM bond paths are shown in yellow and BCPs in orange.

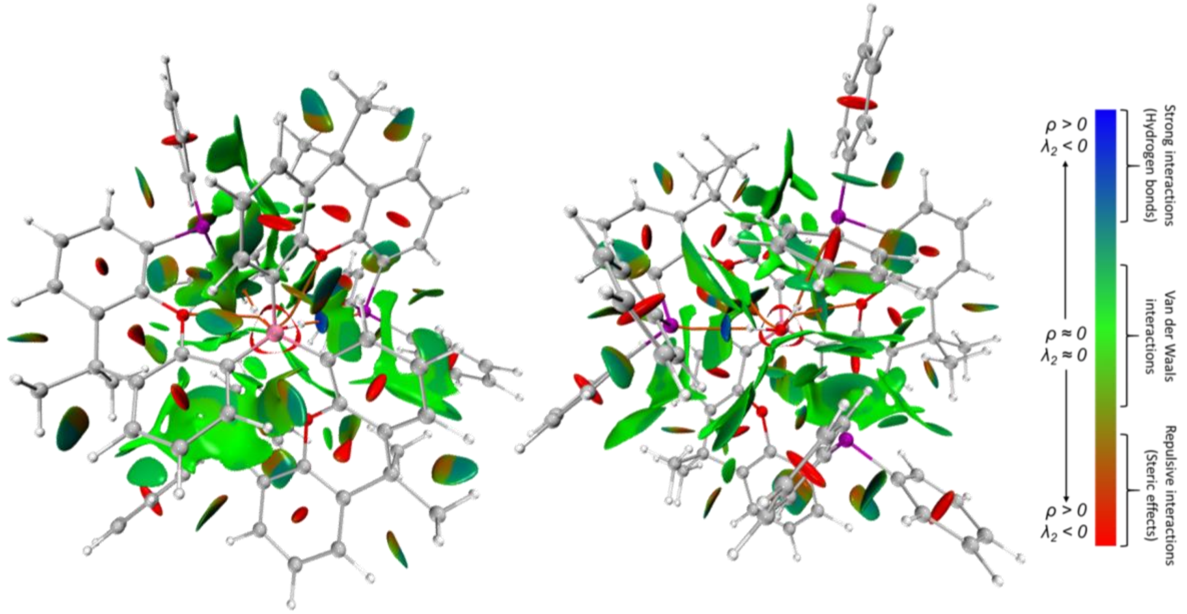

Figure S52: NCI plot of complex **3** with the isosurface set at 0.5 a.u, and BGR from -0.03 to 0.02. QTAIM bond paths are shown in yellow and BCPs in orange.

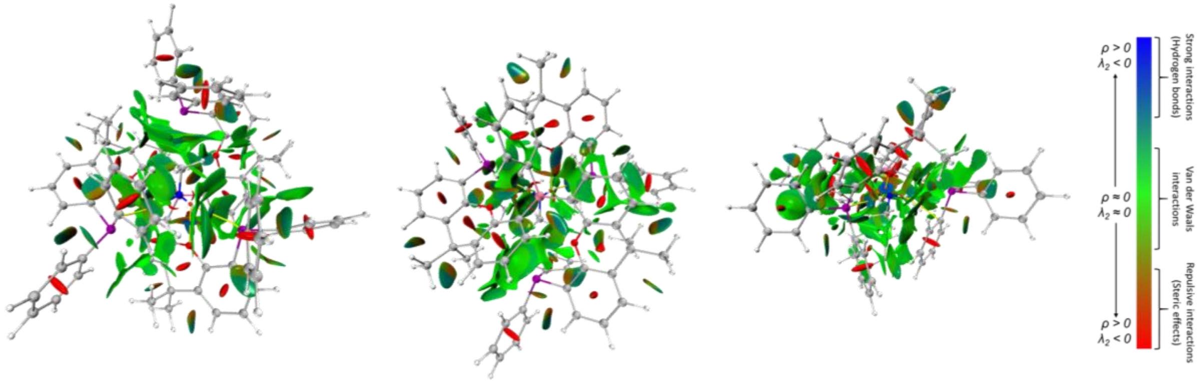

Figure S53: NCI of complex **4** with the isosurface set at 0.5 a.u, and BGR from -0.03 to 0.02. QTAIM bond paths are shown in yellow and BCPs in orange.

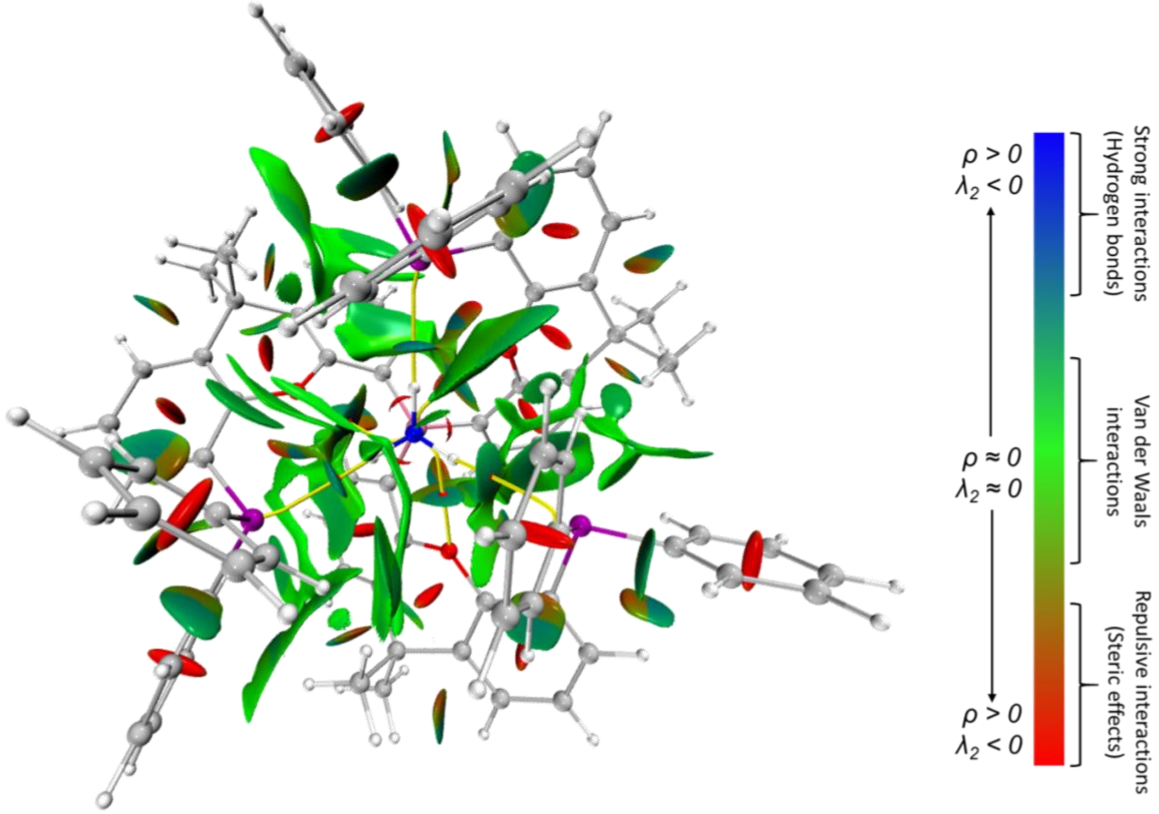

Figure S54: NCI of complex **5** with the isosurface set at 0.5 a.u, and BGR from -0.03 to 0.02. QTAIM bond paths are shown in yellow and BCPs in orange.

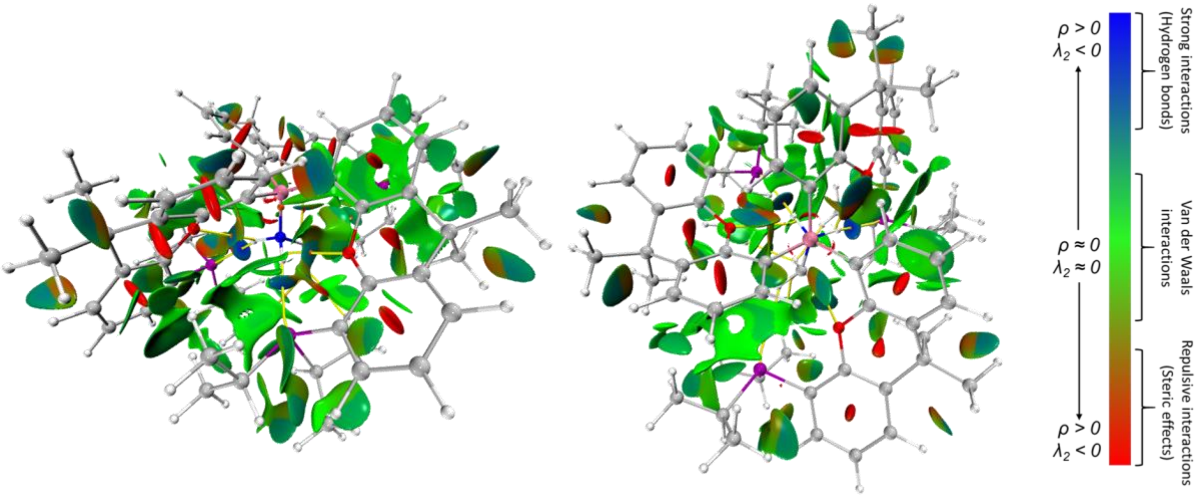

Figure S55: NCI of complex **6** with the isosurface set at 0.5 a.u, and BGR from -0.03 to 0.02. QTAIM bond paths are shown in yellow and BCPs in orange.

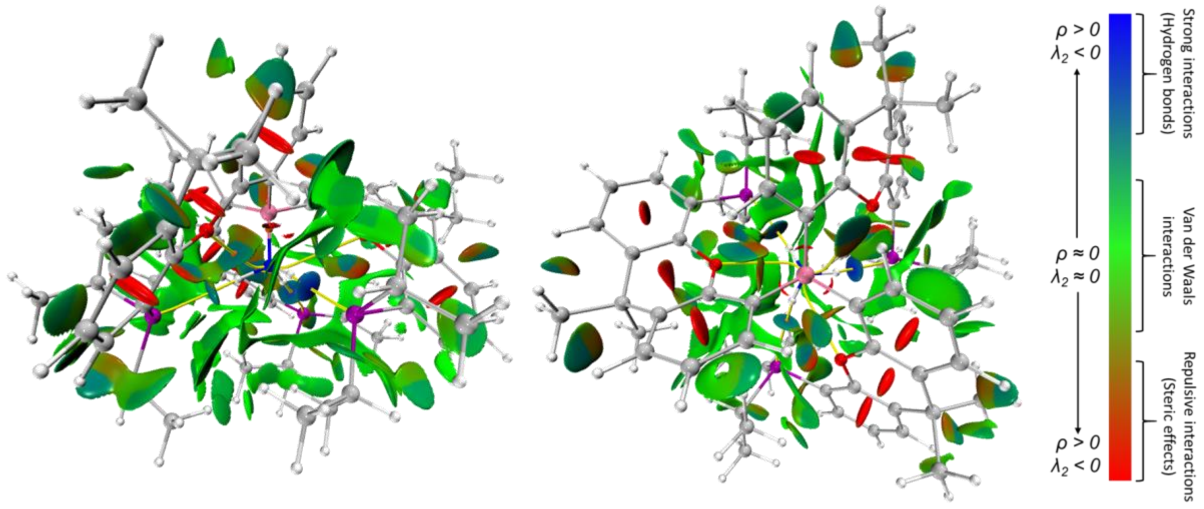

Figure S56: NCI of complex **7** with the isosurface set at 0.5 a.u, and BGR from -0.03 to 0.02. QTAIM bond paths are shown in yellow and BCPs in orange.

Energy Decomposition analysis

In order to further investigate the binding of molecules to {(Ph_2P)xanth}$_3$B (**2**), DFT calculations were performed on each complex. Energy Decomposition analysis (EDA) can be used to break down the interactions between two fragment wavefunctions into more chemically intuitive quantities.[28] Two complementary EDA methods where employed here; SobEDA was used to split the interaction into electrostatic (ΔE_{elst}), exchange-repulsion (ΔE_{xrep}, linked to steric repulsion and Pauli repulsion), orbital (ΔE_{orb}, orbital overlap effects) and Coulomb correlation (ΔE_c, DFT and dispersion). [29] Extended Transition State - Natural Orbitals for Chemical Valence (ETS-NOCV) analysis was then used as a complementary technique in order to decompose the Orbital component into the most significant orbital deformation densities that make up the orbital component.[30,31]

The repulsive $\Delta E_{X\text{-}R}$ term scales as expected with the steric bulk of the bound molecule with water having the smallest and ^{t}BuNC complex having the largest. The magnitude of the ΔE_{orb} and ΔE_{elst} terms are very similar (within 1-2 kcal mol^{-1}) the CO analogues whereas the protic donors have slightly larger deviation with ΔE_{elst} being larger than ΔE_{orb} (with a difference of ~15 kcal mol^{-1}) fitting with the presence of more electrostatic interactions between the phosphine and oxygen donors and the polarised E-H bonds. The trend for ΔE_{orb} of the protic donors follows the nucleophilicity of the lone pair. For the CO analogues the isonitrile is significantly more nucleophilic than the nitrile and thus has a higher value of ΔE_{orb}. The overall interaction energies however follow the expected trend.

Breaking down ΔE_{orb} into deformation density pairs allows Figures S57-S62 to be constructed, in which the purple regions correspond to depletion (donation) of electron density into the green regions (acceptance) with the eigenvalue corresponding to the amount of transferred electron density. The difference between the nitrile and CO complexes is related to σ donating ability with the CO being a better σ donor; π back bonding in the CO complex is also almost double that of the isonitrile and nitrile systems. This back bonding occurs between the B-C bonds and the π* orbitals. The protic complexes all show a primary ΔE_{orb} deformation density pair associated with the N/O lone pair and the vacant *p* orbital on boron, with the other pair densities comprising of a mixture of E-H back bonding (E-H σ* to B-C σ) and hydrogen bonding from the phosphine donors to the E-H bond. The water complex demonstrates the clearest hydrogen bond; this is corroborated by the second order perturbation theory which quantifies this hydrogen bond at 22.5 kcal mol^{-1}. The ammonia complex shows primarily

back donation in the NOCV, however SOPT indicates two hydrogen bonds of 7.4 and 5.6 kcal mol^{-1}. The methylamine adduct shows both hydrogen bonding and backdonation in the NOCV with SOPT analyses, with a H-bond of 8.9 kcal mol^{-1}. The EDA analysis predicts that the methylamine guest binds more strongly, however the Gibbs free energy of binding is larger for the ammonia complex likely due to the large distortion of the cage upon binding of the $MeNH_2$.

The ^{i}Pr cage system displays increased hydrogen bonding when compared to the Ph system as seen by significantly larger hydrogen bonding interaction (e.g. 39.5 vs 13.0 kcal mol^{-1} for the NH_3 complexes) as expected for a more basic phosphine.[32] The Gibbs energy of binding is larger for the ammonia complex as with the Ph system but incomplete conversion is attributed to a large kinetic barrier.

Table S3: SobEDA analysis with all values in kcal mol^{-1}.

	**8**	**9**	**10**	**3**	**5**	**4**	**7**	**6**
Gibbs free energy of binding ΔG_B	-6.06	-6.96	-10.16	-10.23	-25.38	-21.88	-20.35	-29.47
Total interaction energy	-34.17	-51.67	-66.44	-46.24	-65.79	-72.51	-73.87	-71.59
Electrostatic (ΔE_{elst})	-76.35	-103.37	-120.7	-81.76	-108.47	-124.3	-131.78	-119.66
Exchange (ΔE_X)	-116.7	-109.49	-129.3	-82.37	-114.81	-121.3	-129.81	-128.74
Pauli repulsion (ΔE_R)	327.96	311.88	359.85	230.14	311.22	335.9	351.97	343.78
Exchange-repulsion (ΔE_{xrep})	211.26	202.39	230.55	147.77	196.41	214.6	222.16	215.04
Orbital (ΔE_{orb})	-115.08	-104.01	-125.49	-72.32	-98.79	-101.71	-107.77	-108.02
DFT correlation (ΔE_{DFTc})	-52.92	-44.38	-46.06	-38.66	-53.34	-58.48	-53.92	-57.34
Dispersion correction (ΔE_{dc})	-1.08	-2.31	-4.75	-1.27	-1.61	-2.63	-2.56	-1.62
Coulomb correlation (ΔE_c)	-54	-46.69	-50.8	-39.93	-54.94	-61.1	-56.49	-58.95

Table S4: EDA-NOCV analysis with all values in kcal mol^{-1}. The NOCV pairs reported are those with the largest 4 eigenvalues.

		**pair 1 (E_{orb1})**		**pair 2 (E_{orb2})**		**pair 3 (E_{orb3})**		**pair 4 (E_{orb4})**	
	Total	eigenvalue	energy	eigenvalue	energy	eigenvalue	energy	eigenvalue	energy
**3**	-68.908	0.520376	-50.256	0.204207	-5.171	0.122998	-4.350	0.118998	-2.492
**5**	-95.097	0.628502	-76.216	0.14806	-3.796	0.144957	-4.015	0.130867	-3.126
**4**	-97.786	0.634393	-75.979	0.15877	-4.34	0.143403	-4.258	0.117546	-2.959
**9**	-99.088	0.582712	-70.346	0.211771	-5.91	0.211249	-5.899	0.169625	-8.107
**10**	-121.474	0.681344	-93.782	0.222287	-6.177	0.221822	-6.157	0.173991	-7.593
**8**	-112.619	0.64694	-83.526	0.305397	-9.495	0.299321	-9.106	0.171388	-7.46
**7**	-104.21	0.633548	-80.843	0.17822	-4.743	0.158678	-3.874	0.14064	-4.981
**6**	-104.132	0.640261	-79.61	0.187142	-5.164	0.157003	-4.256	0.144139	-5.128

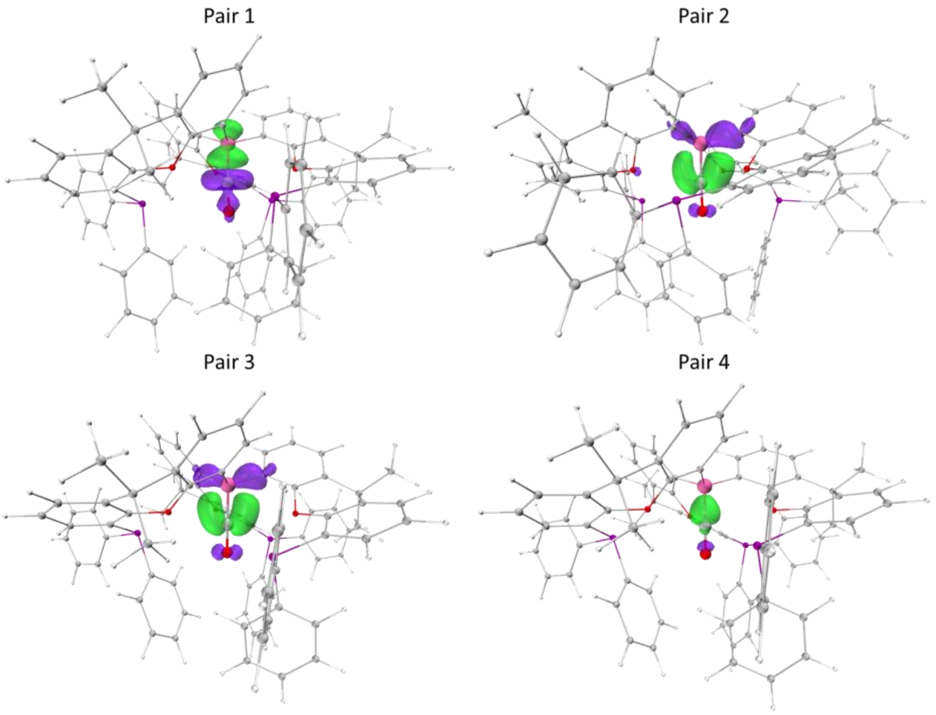

Figure S57: NOCV pair deformation densities for complex **8,** isosurface set to 0.005 for pair 1 and 0.002 a.u for pairs 2-4. Electron donation from purple to green.

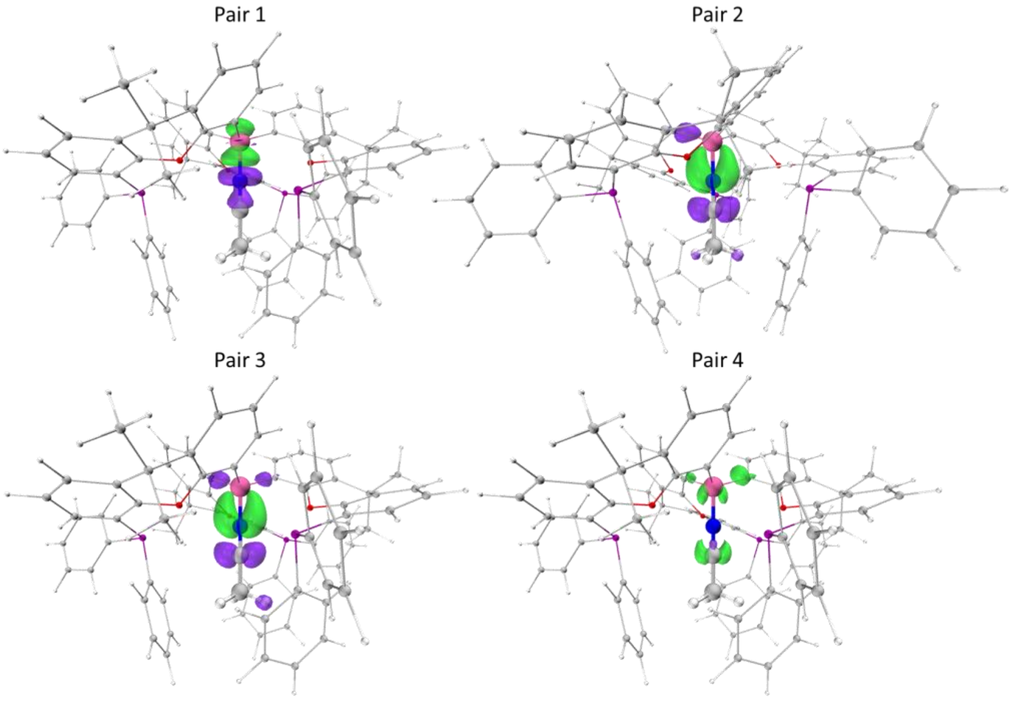

Figure S58: NOCV pair deformation densities for complex **9,** isosurface set to 0.005 for pair 1 and 0.002 a.u for pairs 2-4. Electron donation from purple to green.

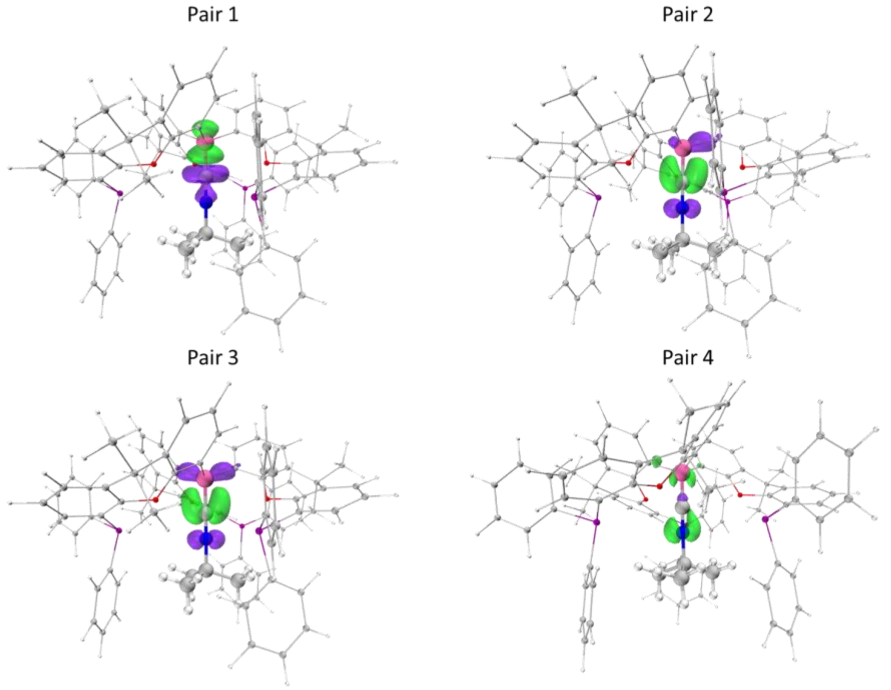

Figure S59: NOCV pair deformation densities for complex **10**, isosurface set to 0.005 for pair 1 and 0.002 a.u for pairs 2-4. Electron donation from purple to green.

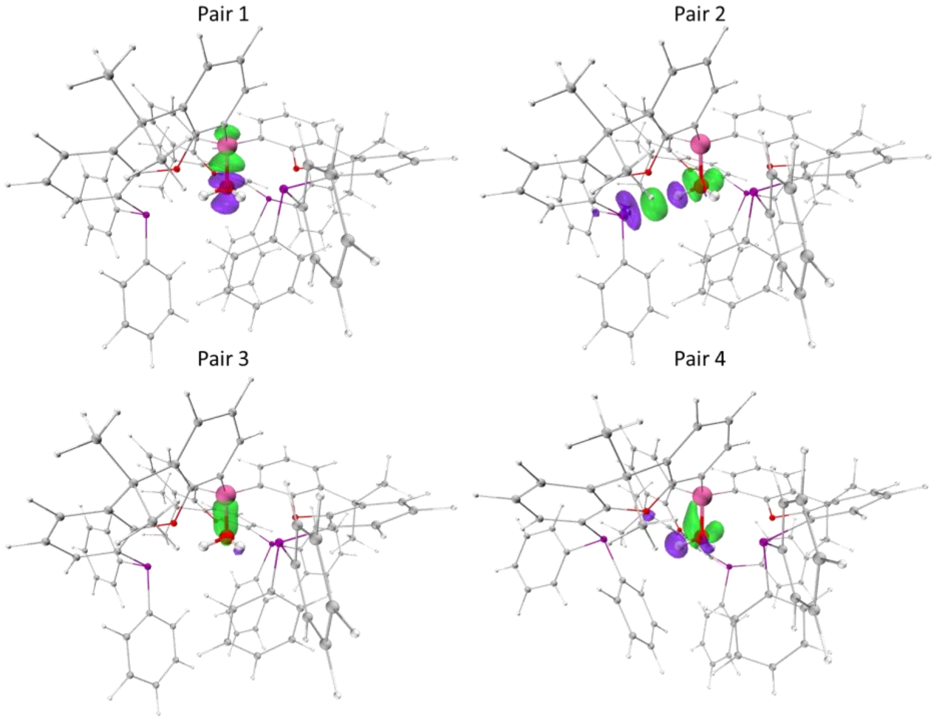

Figure S60: NOCV pair deformation densities for complex **3,** isosurface set to 0.005 for pair 1 and 0.002 a.u for pairs 2-4. Electron donation from purple to green.

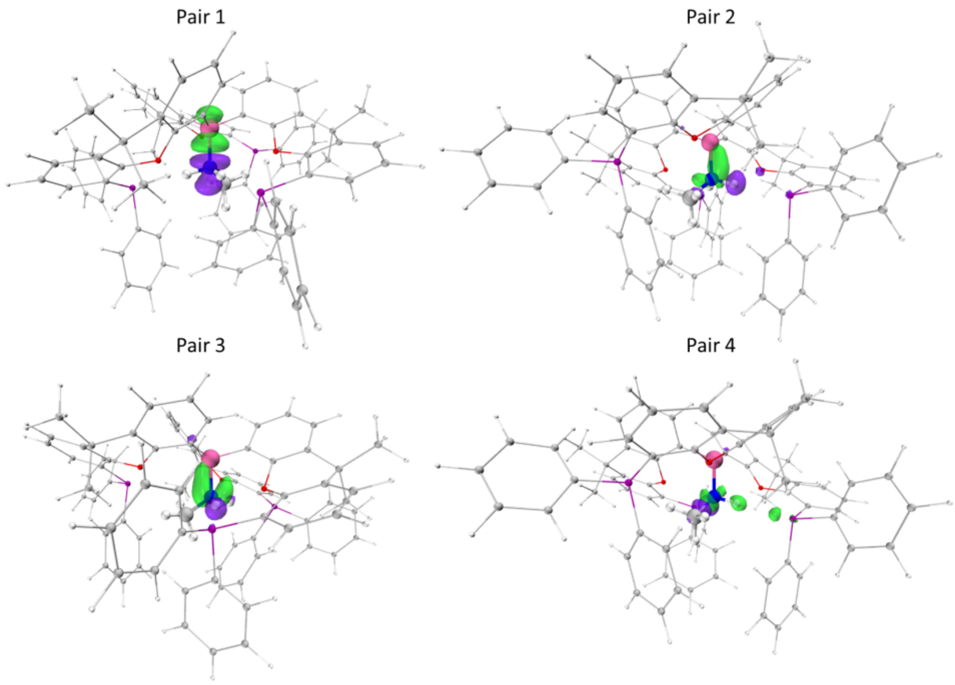

Figure S61: NOCV pair deformation densities for complex **4,** isosurface set to 0.005 for pair 1 and 0.002 a.u for pairs 2-4. Electron donation from purple to green.

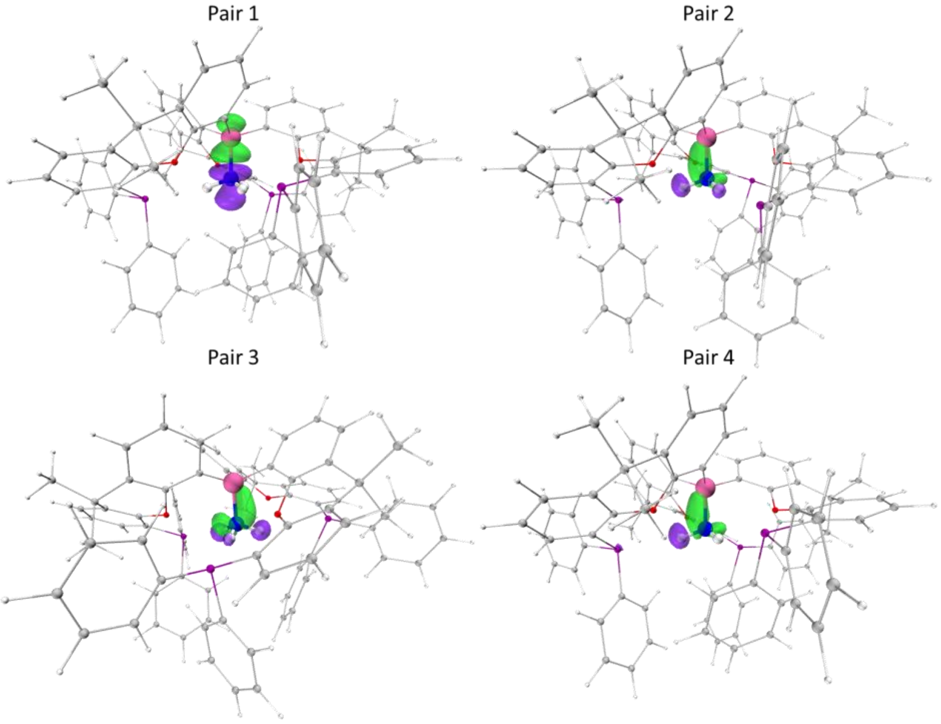

Figure S62: NOCV pair deformation densities for complex **5,** isosurface set to 0.005 for pair 1 and 0.002 a.u for pairs 2-4. Electron donation from purple to green.

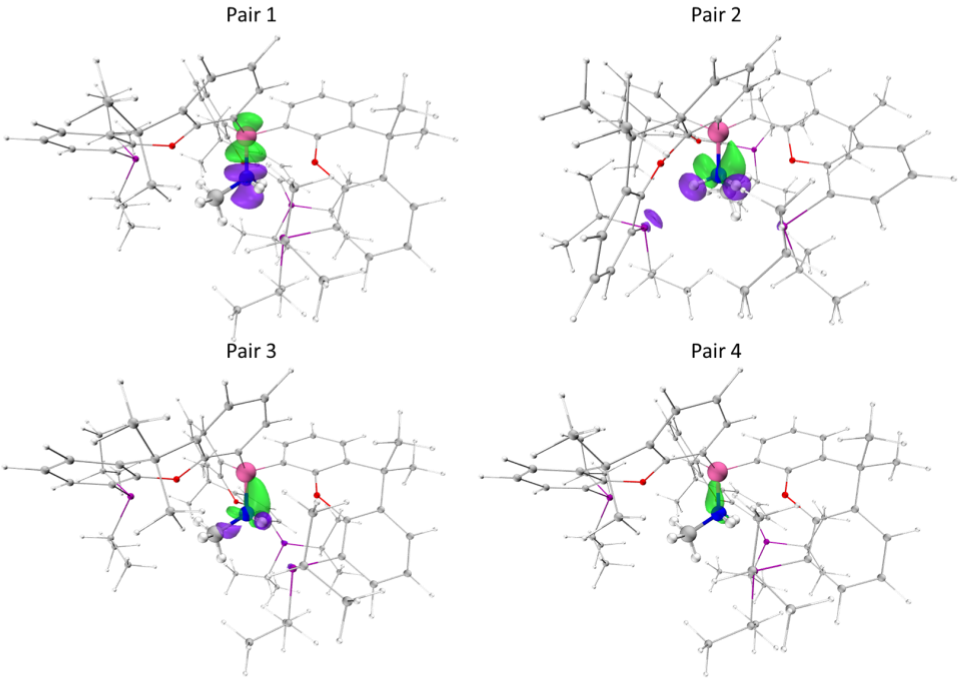

Figure S63: NOCV pair deformation densities for complex **6,** isosurface set to 0.005 for pair 1 and 0.002 a.u for pairs 2-4. Electron donation from purple to green.

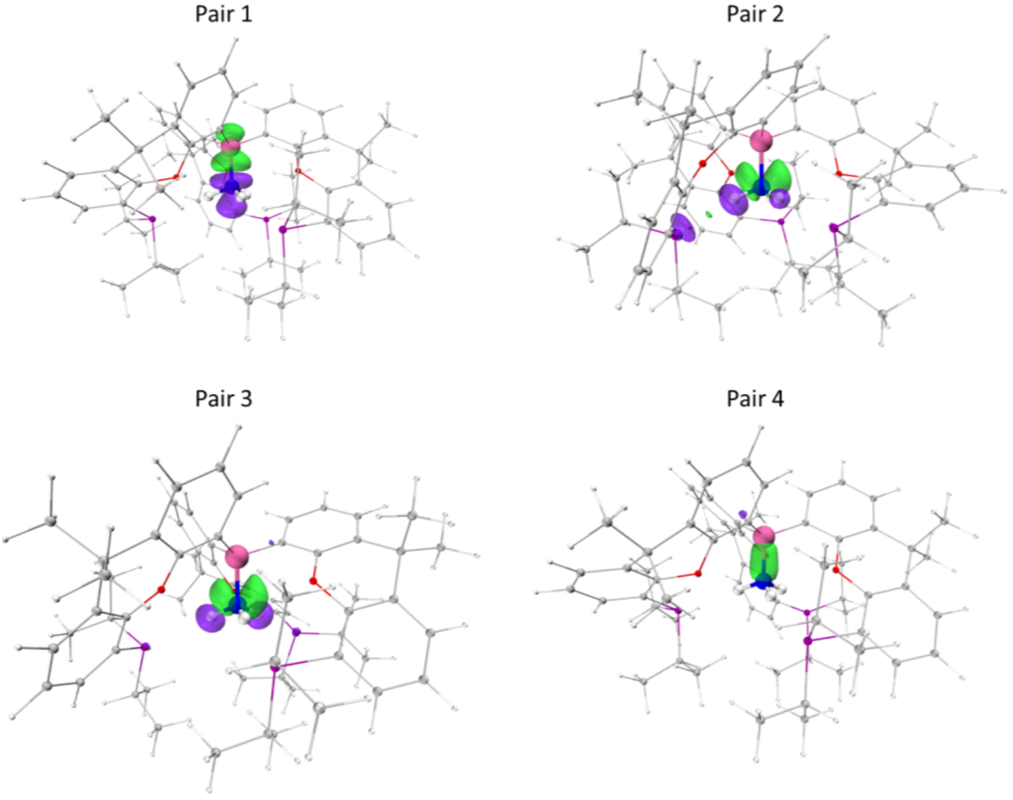

Figure S64: NOCV pair deformation densities for complex **7**, isosurface set to 0.005 for pair 1 and 0.002 a.u for pairs 2-4. Electron donation from purple to green.

Reverse Binding studies

In order to examine the effect of the cage architecture on the binding of various substrates, the binding of the guest molecule outside the cage was investigated. All systems freely optimised, however dissociation was overserved for **3** and **9** so these system where constrained at the same bond length as the internally bound systems.

While **8-11** displayed similar values for ΔE_{orb} for the binding within and outside the cage, this is not true of the protic systems (**3-7**). This can be attributed to the greater effect of hydrogen bonding for these systems which is accounted for through the ΔE_{orb} term. Much more significant changes in the values of ΔG_B are also seen for the protic systems, with the stability afforded by the cage architecture exceeding 120 kcal mol^{-1}. The ΔE_{elst} term is also found to play a greater role in the protic systems, as might be expected by more polar substrates. While in **8-11** a change in the ΔE_{xrep} is seen to greatly stabilise the internally bound systems, however, this is not the case in the protic systems likely due to the greater degree of rearrangement within the cage framework to accommodate the required number of hydrogen bonding interactions.

Table S5: SobEDA decomposition analysis for **3-7** and **3'-7'** (in kcal mol^{-1})

	**3**	**3'**	**Δ**	**4**	**4'**	**Δ**	**5**	**5'**	**Δ**	**6**	**6'**	**Δ**	**7**	**7'**	**Δ**
Gibbs free energy of binding ΔG_B	-10.23	64.84	-75.07	-21.88	65.97	-87.84	-25.38	67.86	-93.24	-20.35	85.60	-105.95	-29.47	93.19	-122.66
Total interaction energy	-46.24	-14.02	-32.22	-72.51	-30.61	-41.90	-65.79	-27.77	-38.02	-73.87	-27.62	-46.25	-71.59	-25.67	-45.92
Electrostatic (ΔE_{elst})	-81.76	-71.16	-10.60	-124.30	-72.52	-51.78	-108.47	-76.56	-31.91	-131.78	-67.72	-64.06	-119.66	-74.09	-45.57
Exchange-repulsion (ΔE_{xrep})	147.77	171.14	-23.37	214.60	162.13	52.47	196.41	175.19	21.22	222.16	154.45	67.71	215.04	172.94	42.10
Orbital (ΔE_{orb})	-72.32	-66.26	-6.06	-101.71	-66.95	-34.76	-98.79	-73.26	-25.53	-107.77	-61.44	-46.33	-108.02	-70.56	-37.46
Coulomb correlation (ΔE_c)	-39.93	-47.74	7.81	-61.10	-53.27	-7.83	-54.94	-53.15	-1.79	-56.49	-52.90	-3.59	-58.95	-53.95	-5.00
DFT correlation (ΔE_{DFTc})	-38.66	-47.11	8.45	-58.48	-51.93	-6.55	-53.34	-52.34	-1.00	-53.92	-51.54	-2.38	-57.34	-53.12	-4.22
Dispersion correction (ΔE_{dc})	-1.27	-0.63	-0.64	-2.63	-1.34	-1.29	-1.61	-0.81	-0.80	-2.56	-1.37	-1.19	-1.62	-0.84	-0.78

Table S6: SobEDA decomposition analyis for **8-11** and **8'-11'** (in kcal mol^{-1})

	**8**	**8'**	**Δ**	**9**	**9'**	**Δ**	**10**	**10'**	**Δ**	**11**	**11'**	**Δ**
Gibbs free energy of binding ΔG_B	-6.06	53.44	-59.50	-6.96	55.82	-62.77	-10.16	46.49	-56.65	-0.88	19.36	-20.25
Total interaction energy	-34.17	-4.67	-29.5	-51.67	-14.43	-37.24	-66.44	-24.03	-42.41	-88.91	-69.96	-18.95
Electrostatic (ΔE_{elst})	-76.35	-66.64	-9.71	-103.37	-74.43	-28.94	-120.7	-80.11	-40.59	-95.57	-96.01	0.44
Exchange-repulsion (ΔE_{xrep})	211.26	237.47	-26.21	202.39	217.77	-15.38	230.55	242.54	-11.99	235.54	259.17	-23.63
Orbital (ΔE_{orb})	-115.08	-113	-2.08	-104.01	-100.82	-3.19	-125.49	-118.61	-6.88	-159.05	-155.7	-3.35
Coulomb correlation (ΔE_c)	-54	-62.5	8.5	-46.69	-56.96	10.27	-50.8	-67.85	17.05	-69.83	-77.41	7.58
DFT correlation (ΔE_{DFTc})	-52.92	-61.92	9	-44.38	-55.87	11.49	-46.06	-65.86	19.8	-68.74	-76.82	8.08
Dispersion correction (ΔE_{dc})	-1.08	-0.58	-0.5	-2.31	-1.08	-1.23	-4.75	-1.99	-2.76	-1.09	-0.6	-0.49

Table S7: EDA-NOCV decomposition analysis for **3-11** and **3'-11'**, eigenvalues are unitless and energies are in kcal mol^{-1}

		**pair 1 (E_{orb1})**		**pair 2 (E_{orb2})**		**pair 3 (E_{orb3})**		**pair 4 (E_{orb4})**	
	Total	eigenvalue	energy	eigenvalue	energy	eigenvalue	energy	eigenvalue	energy
**3**	-68.908	0.520376	-50.256	0.204207	-5.171	0.122998	-4.35	0.118998	-2.492
**3'**	-65.016	0.537999	-50.751	0.138071	-4.596	0.125929	-2.19	0.115288	-2.79
**4**	-95.097	0.628502	-76.216	0.14806	-3.796	0.144957	-4.015	0.130867	-3.126
**4'**	-72.64	0.637984	-61.724	0.133333	-4.363	0.098267	-1.343	0.098197	-1.341
**5**	-97.786	0.634393	-75.979	0.15877	-4.34	0.143403	-4.258	0.117546	-2.959
**5'**	-65.767	0.62852	-54.653	0.130122	-3.762	0.097503	-1.346	0.091144	-1.227
**6**	-104.132	0.640261	-79.61	0.187142	-5.164	0.157003	-4.256	0.144139	-5.128
**6'**	-60.313	0.619893	-50.2	0.122374	-3.241	0.092276	-1.191	0.085517	-1.051
**7**	-104.21	0.633548	-80.843	0.17822	-4.743	0.158678	-3.874	0.14064	-4.981
**7'**	-69.947	0.612548	-59.647	0.127987	-4.017	0.095745	-1.239	0.095031	-1.245
**8**	-112.619	0.64694	-83.526	0.305397	-9.495	0.299321	-9.106	0.171388	-7.46
**8'**	-110.562	0.710748	-80.084	0.322081	-9.899	0.321591	-9.875	0.173675	-7.921
**9**	-99.088	0.582712	-70.346	0.211771	-5.91	0.211249	-5.899	0.169625	-8.107
**9'**	-98.948	0.624549	-73.645	0.217473	-5.832	0.217395	-5.828	0.177035	-9.06
**10**	-121.474	0.681344	-93.782	0.222287	-6.177	0.221822	-6.157	0.173991	-7.593
**10'**	-116.249	0.75276	-92.579	0.234551	-6.126	0.234246	-6.112	0.181092	-8.346
**11**	-140.83	0.743028	-109.392	0.200718	-9.619	0.160081	-3.982	0.159655	-3.971
**11'**	-136.816	0.815478	-110.506	0.208395	-10.015	0.162977	-3.488	0.162812	-3.482

Fluoride Ion Affinity (FIA)

FIA calculations were performed on the binding models, considering the fluoride ion bound both inside and outside the cage. The starting structure for the cage was the *up,up,up* conformation, as this represents the ground-state and resting-state geometry.

FIA calculations were carried out following the optimization procedure described above, with single-point energies computed using the DSD-BLYP double-hybrid functional [33] in conjunction with D3 dispersion correction with Becke–Johnson damping.[34] The def2-QZVPP basis set was employed to ensure high accuracy. [19,20] This methodology has previously been demonstrated to yield highly accurate FIA values, showing excellent agreement with CCSD(T) extrapolated to the complete basis set (CBS) limit.[35]

Table S8: FIAs of **2** and $B(NMe_2)_3$ in kcal mol^{-1}

	**FIA**
**2** Inside cage	-82.1
**2** Outside Cage	-63.6
$B(NMe_2)_3$	-36.7

Xyz coordinates

MeCN

Energy: -132.8614743

C	8.54863607	11.25006612	16.21551116
H	8.91755462	10.58463537	17.00079088
H	8.77593108	10.81176141	15.23987396
H	9.05405682	12.21605790	16.29900984
N	5.97627990	11.56653528	16.46500637
C	7.11782369	11.42844533	16.35372500

CO

Energy: -113.3860758

O	6.97995094	11.42400442	16.42674429
C	5.85733906	11.54945558	16.46408571

Cage

Energy: -4404.017809

P	6.57410736	12.88251814	13.23258515
P	6.99463127	13.57496747	19.38885877
P	5.65971008	7.73766833	16.66481810
O	5.57217954	14.21807436	16.89201615
O	5.17325378	9.91546791	18.55134917
O	4.82289733	10.70234202	14.12747133
C	4.54013695	14.25946952	15.96962098
C	5.85509118	15.38064983	17.57731934
C	5.14140423	16.66835670	15.55325727
C	4.27780103	15.44546265	15.27908455
C	5.16552876	8.71574476	19.23474711
C	5.55485351	16.62566987	17.01525648
C	4.46036698	10.96687930	19.10185779
C	3.95700386	11.93035600	18.21095490
C	6.47529334	15.25269703	18.82778072
C	3.82689287	13.06727233	15.75852418
C	4.30756433	11.04768093	20.48837442
C	4.97674594	8.68395812	20.61784703
C	3.82660108	9.97724067	14.74283438
C	5.00553091	10.58899397	12.77299468
C	5.38907083	7.55655620	18.47742069
C	5.81275591	11.57318086	12.17578899
C	7.31643284	13.88005746	21.17963985
C	6.71039807	16.42037169	19.55958444
H	7.18682880	16.35054803	20.53274473
C	5.78048306	17.76196159	17.79015347
H	5.53912638	18.74265432	17.39451747
C	3.48388462	10.40871105	16.03824295
C	6.63753279	14.27140583	12.02074502
C	8.35396139	12.38597557	13.28596299
C	5.36314266	6.32937631	19.14389470
H	5.53829496	5.41554412	18.58495447
C	6.33773265	17.66204006	19.06113956
H	6.51147234	18.55785456	19.64991764
C	3.30223694	13.03145994	18.78811257
H	2.90373175	13.80356946	18.13465545
C	4.92689361	7.43566704	21.23940941
H	4.76585208	7.37027397	22.31009775
C	6.23042078	14.24044221	21.99103851
H	5.26423569	14.44344142	21.53581526
C	4.42536998	9.55301816	12.03553741
C	4.97587886	10.00349244	21.37231812
C	3.60549110	12.13912330	20.99777070
H	3.46052770	12.23455339	22.06866616
C	8.72228660	13.50279223	18.73700549
C	4.30406529	9.89101960	22.74349054
H	4.81846457	9.15195923	23.36311087
H	3.25015966	9.60617956	22.65570671
H	4.36984942	10.84176200	23.27898851
C	5.10869042	6.26652084	20.50901563
H	5.07709334	5.30361235	21.01032234
C	3.26584863	15.42675601	14.31996555
H	3.03618424	16.32736850	13.76049785
C	3.25283381	8.86752204	14.12682463
C	4.42881256	17.97247394	15.18579448
H	4.19221691	17.98987949	14.11876832
H	5.08021804	18.83080779	15.37044724
H	3.50243008	18.10243983	15.75536457
C	5.99527942	11.52395512	10.79255924
H	6.58535692	12.29670499	10.30784539
C	5.80053259	5.97603684	16.14264415
C	3.11034114	13.13139967	20.15776408
H	2.57703440	13.98051949	20.57584850
C	6.43480567	16.56503873	14.70589329
H	6.96964782	15.63238835	14.91221748
H	7.09696657	17.40670316	14.93826286
H	6.18691465	16.58545768	13.63963572
C	5.41740347	14.75621144	11.52831691
H	4.49238950	14.24517981	11.78636165
C	4.66044730	9.53180186	10.65949498
H	4.24056318	8.73027459	10.05977502
C	7.81230191	14.95349797	11.69420840
H	8.76912171	14.59794942	12.06624510
C	2.58084068	9.60641586	16.74879800
H	2.30117344	9.89978835	17.75754101
C	6.37311352	14.33574526	23.36950923
H	5.52077963	14.62358632	23.97940649
C	2.84530807	13.10175512	14.75254591
H	2.27363823	12.19941952	14.55161417
C	11.11055822	11.90293809	13.44099766
H	12.17990614	11.71796864	13.49802712
C	5.42513386	10.50917656	10.03485475
H	5.58087545	10.47552884	8.96078828
C	6.45543043	10.41302910	21.58516737
H	6.50687112	11.36448545	22.12468878
H	6.97036294	10.53464156	20.62677920
H	6.97328565	9.64159305	22.16641374
C	9.10651574	12.88893001	14.35394446
H	8.61602314	13.46675176	15.13556185
C	8.99516042	11.61359057	12.31184811
H	8.42602823	11.19724347	11.48601349
C	2.34888948	8.11640949	14.88198823
H	1.90646149	7.22261370	14.45142294
C	7.76778183	16.09569125	10.89557504
H	8.69108175	16.61465919	10.65209365
C	10.47824363	12.65857866	14.42409270
H	11.04530015	13.06102197	15.25781944
C	4.60692235	5.24630567	16.04018753
H	3.66684706	5.71600231	16.32464209
C	10.36412965	11.37278600	12.39013066
H	10.84901525	10.76984636	11.62691484
C	3.63563292	8.44350467	12.71546200
C	7.60055993	14.05453946	23.96964909
H	7.71032938	14.11966734	25.04847642
C	2.55864679	14.26080487	14.04705122
H	1.78151344	14.26256728	13.28805324
B	3.89414411	11.78485527	16.65595166
C	8.54513195	13.61036703	21.78916875
H	9.40258029	13.33006466	21.18409483
C	6.55350942	16.56757698	10.40820630
H	6.52120911	17.45723050	9.78555721
C	2.02134017	8.46742769	16.18510921
H	1.32421670	7.85868437	16.75363491
C	9.29722454	12.23419041	18.59934844
H	8.69745014	11.34555764	18.78849786
C	9.49418217	14.63265469	18.44776926
H	9.05801548	15.62398804	18.53670775
C	5.37665886	15.88771590	10.72409477
H	4.42196468	16.24599132	10.34788756
C	8.68278714	13.69338187	23.17415832
H	9.64568138	13.47784055	23.62959370

C	4.61225021	3.93513142	15.58078312
H	3.67920799	3.38147048	15.51839507
C	10.63446071	12.10143312	18.23523917
H	11.07053508	11.11127090	18.14781613
C	4.52788429	7.18282746	12.78827564
H	3.99105369	6.36261617	13.27722151
H	5.43573311	7.38312734	13.36628079
H	4.81766787	6.86398293	11.78087532
C	11.40216431	13.23213714	17.97146449
H	12.44550335	13.12823206	17.68497438
C	6.99480401	5.36774068	15.74704545
H	7.92996091	5.91677664	15.81245014
C	10.82435599	14.49689367	18.06062310
H	11.41445189	15.38253786	17.84026301
C	5.80900497	3.33734618	15.18601390
H	5.81338017	2.31631411	14.81482738
C	6.99583983	4.05888612	15.26661856
H	7.93265029	3.60141492	14.95973279
C	8.33089216	8.07537432	17.64884993
H	8.00623698	7.58724675	18.56450443
C	7.41591268	8.28803062	16.61260042
C	7.84674135	8.92796630	15.44535151
H	7.13615988	9.12761195	14.64520819
C	9.17580364	9.31287967	15.29983327
H	9.49836137	9.80519861	14.38798151
C	10.08370675	9.08257118	16.32913865
H	11.12015636	9.38897879	16.21409933
C	9.65739738	8.47356843	17.50786224
H	10.36056442	8.30029949	18.31876503
C	2.36324235	8.12225200	11.90562372
H	2.61258902	7.77300324	10.90039136
H	1.72636394	9.00850157	11.82062669
H	1.78809296	7.32606544	12.38439246

CageMeCN

Energy: -4536.914784

P	6.32318152	13.24740427	12.90932131
P	6.98357795	13.68135570	19.64317461
P	6.01740745	7.61934466	16.70938173
O	5.72947851	14.34689599	17.04907019
O	5.33903968	9.79446747	18.60149412
O	5.04011235	10.77290091	13.89951640
C	4.69222001	14.35598663	16.11180323
C	5.91205706	15.49222643	17.78742001
C	5.19679785	16.78996472	15.78138805
C	4.34753544	15.56113618	15.49492896
C	5.23914334	8.57669215	19.23143134
C	5.57712222	16.74036433	17.25108261
C	4.55590543	10.83637578	19.10693881
C	4.12086452	11.80380066	18.19401202
C	6.48368049	15.35663648	19.06050284
C	4.06240587	13.13410783	15.85026973
C	4.25801320	10.85899991	20.47158139
C	4.92700447	8.50808437	20.59375385
C	4.06376174	10.04399697	14.58527526
C	4.90885157	10.85434287	12.53315147
C	5.50895398	7.43023862	18.47064421
C	5.44442558	11.98304790	11.89733430
C	7.36264501	14.03548248	21.41375454
C	6.63825334	16.51144182	19.83300465
H	7.07015653	16.43531529	20.82599837
C	5.72080763	17.86342461	18.06293031
H	5.44670782	18.84247034	17.68438088
C	3.75792720	10.45822405	15.88616277
C	6.52717427	14.59615526	11.66660615
C	8.02598842	12.53973527	12.97405963
C	5.37708866	6.18617221	19.09438048
H	5.57358199	5.28207057	18.52676663
C	6.23472231	17.75044112	19.35119693
H	6.34423608	18.63463988	19.97208985
C	3.31534529	12.81697461	18.72310877
H	2.94383329	13.59000419	18.05502185
C	4.77726204	7.24882184	21.17051748
H	4.51255692	7.16261840	22.21916127
C	6.28263490	13.96848965	22.30359180
H	5.29443806	13.71304486	21.92496087
C	4.30305061	9.81477976	11.81881944
C	4.89884025	9.81309427	21.37111994
C	3.43541603	11.88333676	20.93950664
H	3.16644654	11.92905574	21.98978606
C	8.67676428	13.53299799	18.92464263
C	4.18855257	9.68028028	22.71850902
H	4.69400834	8.94189451	23.34729110
H	3.14128295	9.38314137	22.59792509
H	4.22428785	10.62987051	23.25957841
C	4.98692027	6.09307097	20.42427455
H	4.87036978	5.11801142	20.88793316
C	3.27747413	15.54949896	14.60051181
H	2.96529171	16.46749093	14.11309387
C	3.45782643	8.95886237	13.94714479
C	4.49418555	18.09286659	15.39860069
H	4.27142378	18.10180940	14.32795861
H	5.14481367	18.95102551	15.58966688
H	3.55956505	18.22843851	15.95323451
C	5.28279798	12.09053071	10.51288903
H	5.68102507	12.95765901	9.99529379
C	5.90731518	5.86909439	16.13670894
C	2.96420175	12.85866646	20.06841771
H	2.32575837	13.65528999	20.44084707
C	6.50924106	16.68593770	14.96117249
H	7.03934807	15.75365467	15.18222793
H	7.16629381	17.52922520	15.20329030
H	6.28362567	16.70299289	13.88948949
C	5.44490875	15.47437797	11.52247933
H	4.55482207	15.32915128	12.13210356
C	4.13628913	9.97281898	10.44477729
H	3.64787565	9.19550153	9.86647272
C	7.67025083	14.80145838	10.88920568
H	8.51699579	14.12689862	10.97894700
C	2.75448654	9.73055562	16.53361546
H	2.47393711	10.01603916	17.54440012
C	6.46342581	14.22137043	23.65898417
H	5.61437209	14.16971706	24.33519641
C	3.01045289	13.17937363	14.92993579
H	2.48336031	12.25787238	14.69603980
C	10.64129605	11.55081115	13.24849221
H	11.65277563	11.16829849	13.35323022
C	4.60959790	11.10946417	9.79573378
H	4.47570072	11.21831664	8.72357147
C	6.37126702	10.22331590	21.63336518
H	6.40507752	11.16918710	22.18463120
H	6.91383439	10.35746566	20.69187530
H	6.87523203	9.44809231	22.22217567
C	9.03330372	13.32464934	13.55536645
H	8.79833048	14.32713646	13.90952272
C	8.34734040	11.24769747	12.54619779
H	7.58475350	10.62142824	12.09096110
C	2.44946512	8.28296460	14.63310775
H	1.94042183	7.44548500	14.16687529
C	7.73074747	15.86554084	9.99154521
H	8.62680616	16.01392621	9.39486098
C	10.33116898	12.84179070	13.67506820
H	11.10027855	13.46966563	14.11688287
C	4.64190464	5.43360316	15.72086858
H	3.80225590	6.12584994	15.75193693
C	9.64418566	10.75579497	12.68996105
H	9.87466575	9.74844337	12.35312160
C	3.98227785	8.54123395	12.58234874
C	7.73205940	14.52506215	24.14944786
H	7.87747138	14.71190882	25.20978391
C	2.60994180	14.36320527	14.31924691
H	1.77909691	14.36190738	13.61882261

B	4.44994913	11.74484030	16.60210799
C	8.63000515	14.34913153	21.91288986
H	9.47907793	14.41425675	21.23835254
C	6.64762745	16.72835754	9.84927874
H	6.69701934	17.55571322	9.14685087
C	2.09711780	8.66837103	15.92153633
H	1.31181695	8.13524937	16.45071927
C	9.44593504	12.42300949	19.30521778
H	9.04705601	11.71430579	20.02902469
C	9.20171742	14.41908689	17.97896513
H	8.62606169	15.28752265	17.67017438
C	5.50017419	16.52547598	10.61406972
H	4.64991793	17.19429791	10.51118578
C	8.81270712	14.58592319	23.27371789
H	9.80383385	14.82725082	23.64844382
C	4.45241051	4.13248429	15.26919546
H	3.46343289	3.80938232	14.95561515
C	10.71619177	12.22345916	18.77792355
H	11.30141484	11.36421213	19.09443071
C	5.31691925	7.77737543	12.78542798
H	5.14170786	6.86397516	13.36363089
H	6.03736943	8.39368919	13.33281532
H	5.74771240	7.51137605	11.81321124
C	11.23099358	13.11178091	17.83423291
H	12.21981398	12.95083068	17.41380241
C	6.98081708	4.97640544	16.07810618
H	7.96822999	5.29025642	16.40445169
C	10.46727079	14.20473427	17.43396778
H	10.85909276	14.90445122	16.70037622
C	5.53043726	3.25124823	15.20547385
H	5.38675779	2.23745252	14.84222075
C	6.79259411	3.67770210	15.60896346
H	7.63665792	2.99444922	15.56751236
C	8.48110365	8.07953890	18.10189435
H	7.90971558	8.06307301	19.02619185
C	7.83978825	7.84917536	16.88068450
C	8.60515078	7.87427232	15.70482388
H	8.12025956	7.71463279	14.74314330
C	9.97700407	8.09228931	15.75349182
H	10.55426554	8.09672269	14.83276507
C	10.60711206	8.32423611	16.97577255
H	11.67747319	8.50662748	17.01346925
C	9.85359223	8.32216884	18.14638948
H	10.33409604	8.50087128	19.10476292
C	3.01287125	7.63068482	11.82923692
H	3.43724209	7.33373189	10.86592171
H	2.04903530	8.12037439	11.65323256
H	2.84029791	6.70961787	12.39321787
C	8.54519720	11.25038327	16.21638071
H	8.90703305	10.58383306	17.00418590
H	8.76425638	10.81234512	15.23839957
H	9.04342087	12.22026951	16.29992269
N	5.99893279	11.56363609	16.46229683
C	7.13144189	11.42703436	16.35273152

CageCO

Energy: -4517.432834

P	6.61904141	12.83254565	13.22115502
P	7.12116320	13.58180129	19.28235070
P	5.86186832	7.75912763	16.72096145
O	5.70454397	14.28281085	16.80221885
O	5.29142048	9.93021305	18.60926691
O	5.02278709	10.52416889	14.09074973
O	6.98007685	11.42399065	16.42673982
C	4.61243412	14.30880567	15.94176253
C	5.85721008	11.54947162	16.46408623
C	5.93797453	15.42212542	17.54301588
C	5.10094651	16.74215577	15.59178053
C	4.24771665	15.50888861	15.33015421
C	5.24267713	8.72083664	19.27185449
C	5.56767487	16.67248579	17.03751177
C	4.50591589	10.96109076	19.11312631
C	4.04340992	11.90064155	18.18683279
C	6.57226106	15.26725671	18.78068998
C	3.94393797	13.09693814	15.74814687
C	4.23909888	11.01943101	20.48170378
C	4.96491148	8.67789636	20.64009725
C	3.91903078	9.93817385	14.69478640
C	5.05111961	10.56447512	12.71685463
C	5.51229661	7.57057899	18.51873256
C	5.79032058	11.59888598	12.12911849
C	7.37795975	13.82478733	21.09076758
C	6.77940789	16.41453643	19.55289481
H	7.27028014	16.32443666	20.51723293
C	5.76384945	17.78594783	17.85153814
H	5.47115677	18.76996592	17.50087125
C	3.58783695	10.43116228	15.95880971
C	6.66387013	14.28315738	12.08663571
C	8.37985340	12.28338188	13.20812255
C	5.45548039	6.33736757	19.17367726
H	5.66234280	5.42847952	18.61749410
C	6.35555578	17.65917301	19.10557827
H	6.50882249	18.53849232	19.72415664
C	3.27133832	12.94801479	18.69378034
H	2.89419151	13.70467894	18.01066028
C	4.88610806	7.42520652	21.24767292
H	4.65656334	7.35099729	22.30536189
C	6.25041636	14.12267802	21.86984607
H	5.29051153	14.28906125	21.38587106
C	4.36983282	9.60021905	11.96660786
C	4.89730734	9.99561938	21.39654187
C	3.43693890	12.06743882	20.93311812
H	3.19754475	12.15192773	21.98796301
C	8.85463490	13.55834909	18.65002208
C	4.17357615	9.86646437	22.73798193
H	4.68679648	9.14875790	23.38364455
H	3.13428762	9.54562522	22.60935966
H	4.18437086	10.82370288	23.26624818
C	5.12323915	6.26283244	20.52094319
H	5.06788208	5.29581705	21.01205305
C	3.14470812	15.48479638	14.47646554
H	2.81978974	16.39450494	13.98231829
C	3.23370791	8.91104181	14.05001714
C	4.35670251	18.04034573	15.27670086
H	4.07224593	18.06666092	14.22125981
H	5.00442103	18.90488343	15.44669096
H	3.45444258	18.14887791	15.88797013
C	5.82160095	11.66627659	10.73309238
H	6.37735833	12.46613548	10.25220994
C	5.89571290	5.99694720	16.18714386
C	2.95713391	13.02615966	20.04712661
H	2.33893011	13.84059845	20.41458482
C	6.36880161	16.67414372	14.70194089
H	6.92093744	15.74476515	14.87298710
H	7.02691461	17.51905865	14.93376919
H	6.08998660	16.71416775	13.64436006
C	5.43154693	14.82430582	11.69299605
H	4.50720222	14.33630406	11.99546694
C	4.41478346	9.71741968	10.57810159
H	3.89433009	8.99478973	9.95869412
C	7.83861966	14.93972522	11.71396429
H	8.80372176	14.53856416	12.01117347
C	2.52730441	9.80616923	16.61419449
H	2.24120473	10.15081042	17.60470398
C	6.34455068	14.19581328	23.25351999
H	5.46166375	14.43492475	23.84053859
C	2.86509459	13.11961593	14.86038932
H	2.31686289	12.20024922	14.67320092
C	11.10812763	11.65069672	13.27685587
H	12.16691998	11.40686857	13.30046547
C	5.13152203	10.74015256	9.96270351
H	5.15567684	10.80858241	8.87919958
C	6.35768246	10.43901044	21.66762714

H	6.36875086	11.38709420	22.21469759
H	6.90545589	10.58049716	20.73017427
H	6.87153928	9.67506843	22.26211941
C	9.20229222	12.78599981	14.22461221
H	8.77723972	13.42167262	15.00005000
C	8.93498204	11.43553959	12.24458351
H	8.30812420	11.02380081	11.45839621
C	2.17026461	8.32712618	14.74176441
H	1.60952516	7.51522967	14.29030665
C	7.78252713	16.11241320	10.96106555
H	8.70539391	16.61251471	10.67959843
C	10.56010654	12.48130023	14.25089083
H	11.18308656	12.88443633	15.04373346
C	4.65480677	5.36986989	16.00690995
H	3.73963653	5.92456230	16.20930580
C	10.29094949	11.12071767	12.28050284
H	10.71051625	10.46163221	11.52501088
C	3.72303877	8.42616156	12.69045090
C	7.56248188	13.95042038	23.88819459
H	7.63430829	13.99727629	24.97116068
C	2.46245108	14.29724260	14.23691648
H	1.60974934	14.29018694	13.56368038
B	4.30345436	11.74713886	16.58634580
C	8.59565398	13.58872819	21.73287284
H	9.48232541	13.35730548	21.14925395
C	6.55660645	16.63635556	10.56395653
H	6.51585903	17.54811647	9.97467023
C	1.82589175	8.76222207	16.01681060
H	1.00025914	8.29143063	16.54302037
C	9.48063071	12.30875054	18.55970237
H	8.92638414	11.40670377	18.81315394
C	9.56725182	14.70157385	18.27535783
H	9.09241661	15.67753190	18.32564071
C	5.37911143	15.98304460	10.92962268
H	4.41535120	16.38492209	10.62806080
C	8.68376277	13.64619577	23.12344293
H	9.63831835	13.45767005	23.60742780
C	4.58319221	4.05303181	15.56965647
H	3.61385766	3.57844192	15.44318650
C	10.80708291	12.21060632	18.15180288
H	11.28140240	11.23549320	18.09830006
C	4.81970073	7.35573051	12.91295131
H	4.40510614	6.49470344	13.44741806
H	5.64262236	7.75850943	13.51120936
H	5.21849197	7.02236123	11.94813256
C	11.51485357	13.35613411	17.79648335
H	12.54944377	13.27964290	17.47236648
C	7.06098283	5.28467228	15.89472594
H	8.03150674	5.75513281	16.02592890
C	10.88787898	14.59884485	17.84539193
H	11.43113147	15.49495097	17.55737412
C	5.75132462	3.34920046	15.27769562
H	5.69672285	2.32334396	14.92423815
C	6.98641254	3.96994785	15.43660659
H	7.90081613	3.42814195	15.21019829
C	8.47767802	8.13052599	17.83991740
H	8.07521946	7.78498491	18.78875386
C	7.64878204	8.20252979	16.71687443
C	8.18413127	8.66171640	15.50693976
H	7.54093039	8.76519111	14.63495802
C	9.52639236	9.00844838	15.41146620
H	9.92433655	9.36645470	14.46654841
C	10.34755774	8.92612263	16.53357598
H	11.39513742	9.20596273	16.46088287
C	9.81924229	8.49550950	17.74794619
H	10.45340846	8.43678692	18.62897530
C	2.59008177	7.80248635	11.86821258
H	2.96686500	7.42394457	10.91443915
H	1.79275708	8.52677684	11.67141324
H	2.16274413	6.94437569	12.39268055

CageH2O
Energy: -4480.534438

P	6.95130887	12.48619905	13.34126839
P	6.94342288	13.66956765	19.47490769
P	5.64729351	7.62745866	16.66588277
O	5.64107341	14.34204915	16.94767739
O	5.23377955	9.83470273	18.53994870
O	5.07110766	10.39008225	14.07790123
C	4.62756463	14.32999130	15.99109827
C	5.74212538	15.47376885	17.72512600
C	5.05754800	16.78492236	15.72712346
C	4.26604395	15.53392436	15.37775902
C	5.13261259	8.63660592	19.21423985
C	5.35985519	16.71643068	17.21393483
C	4.50401785	10.91426684	19.05509159
C	4.06414560	11.86828465	18.13308722
C	6.28830205	15.32583988	19.00587854
C	4.01637254	13.09570200	15.72440933
C	4.27810206	10.98227883	20.43041074
C	4.89381245	8.61628605	20.58999980
C	3.88882368	9.92457181	14.64877902
C	5.13988229	10.45925650	12.71491800
C	5.32403153	7.46671592	18.46722631
C	6.00160660	11.42345941	12.18010354
C	7.47096498	13.98918698	21.21361235
C	6.34771665	16.45864122	19.82129803
H	6.76679115	16.37661357	20.81931128
C	5.40581051	17.81794372	18.06646510
H	5.09226938	18.79312549	17.70886147
C	3.57447182	10.45914870	15.90166221
C	6.91332674	14.08281548	12.42312829
C	8.69591755	11.93931207	13.13706174
C	5.20298576	6.24212726	19.12878755
H	5.34488019	5.31887533	18.57582243
C	5.87763597	17.68679142	19.36935499
H	5.91384037	18.55324525	20.02315853
C	3.31637243	12.92269222	18.66652137
H	2.93854397	13.68876704	17.99330054
C	4.74987380	7.37456294	21.20834420
H	4.54309921	7.32132937	22.27197715
C	6.45842133	14.03615068	22.18327449
H	5.41907355	13.93039685	21.87641507
C	4.37630108	9.59795319	11.91995940
C	4.92993610	9.94247962	21.33060541
C	3.51652163	12.05096776	20.90334854
H	3.30282917	12.13946909	21.96353222
C	8.56643454	13.70576374	18.59077805
C	4.27007784	9.86348782	22.70779519
H	4.78052823	9.12815107	23.33602803
H	3.21152525	9.59184641	22.63528950
H	4.35208826	10.82512963	23.22194706
C	4.89287443	6.19598587	20.48307334
H	4.78538023	5.23664939	20.98052298
C	3.21424836	15.50801648	14.46315253
H	2.89028806	16.42399212	13.98000831
C	3.12776061	8.98002603	13.96461182
C	4.33259017	18.07007495	15.32948256
H	4.17537643	18.09678958	14.24729061
H	4.93992534	18.94442120	15.58040792
H	3.36171948	18.16111453	15.82835892
C	6.05990966	11.55261342	10.78925611
H	6.69888737	12.31372885	10.34924737
C	5.90232638	5.86474110	16.19269104
C	3.03420484	13.01542995	20.02552527
H	2.44301995	13.84477912	20.40448857
C	6.41796016	16.74008183	14.98351690
H	6.97294011	15.82748269	15.22380834
H	7.02749647	17.60340747	15.27440870
H	6.25470910	16.76434253	13.90189013
C	5.65760235	14.60550996	12.08991441
H	4.75433034	14.06260701	12.35765567

C	4.46000478	9.76448452	10.53918316
H	3.87895161	9.12587232	9.88212839
C	8.06099578	14.80849367	12.09780709
H	9.04369579	14.41967124	12.34961608
C	2.43283176	9.92521965	16.50749116
H	2.13858736	10.29745474	17.48607900
C	6.76852157	14.21159658	23.52630008
H	5.97026596	14.25514096	24.26247350
C	2.98953833	13.12994431	14.77029125
H	2.47245910	12.20488267	14.52979913
C	11.39269113	11.19993300	12.99460602
H	12.43870143	10.91142327	12.93852449
C	5.28781833	10.73496938	9.97529385
H	5.33131296	10.84673375	8.89615505
C	6.42204293	10.31928097	21.52331234
H	6.50253265	11.27515972	22.05129899
H	6.92978617	10.41934489	20.55866318
H	6.92832441	9.54310096	22.10873505
C	9.60770943	12.38615380	14.10261638
H	9.26564087	13.00963743	14.92755709
C	9.14540555	11.10007312	12.11379016
H	8.44804370	10.72553809	11.37042784
C	1.99139388	8.49385648	14.61508165
H	1.36492824	7.74857065	14.13578522
C	7.95399602	16.02898528	11.43271943
H	8.85493295	16.58109727	11.17953921
C	10.94971344	12.02955682	14.02194371
H	11.64531218	12.38712333	14.77501698
C	4.75850188	5.13712617	15.84029656
H	3.78571610	5.62587923	15.85968453
C	10.48670665	10.73056951	12.04665836
H	10.82324314	10.07280032	11.24993506
C	3.60166245	8.47771248	12.60354898
C	8.09956161	14.31659603	23.92943282
H	8.34419040	14.44186878	24.98050958
C	2.58236106	14.30942856	14.15483231
H	1.76758874	14.29547390	13.43588247
B	4.33665803	11.73471747	16.54861026
C	8.80118407	14.10075547	21.62608006
H	9.60203449	14.06838437	20.89289416
C	6.70483426	16.53244186	11.08281057
H	6.62488821	17.48029838	10.55802160
C	1.65660718	8.95063035	15.88542308
H	0.77590216	8.55589901	16.38455644
C	9.26206998	12.49643346	18.47410132
H	8.81484237	11.57835928	18.85142002
C	9.13940039	14.86755561	18.06330582
H	8.60992746	15.81360919	18.13709953
C	5.55617320	15.81561282	11.41559648
H	4.57446444	16.20382638	11.15750197
C	9.11150886	14.25681412	22.97658906
H	10.15146736	14.33899405	23.28131224
C	4.85602182	3.79986296	15.47105669
H	3.95940993	3.24538068	15.20791867
C	10.52509334	12.46032909	17.89050105
H	11.06038484	11.51796207	17.82458773
C	4.58269985	7.30087756	12.82900610
H	4.06438419	6.46927576	13.31800772
H	5.41334679	7.60226339	13.47390241
H	4.98737022	6.95649821	11.87049298
C	11.09581268	13.62621935	17.38559890
H	12.08438491	13.60050285	16.93497997
C	7.14835946	5.23382834	16.14507951
H	8.04606385	5.78450051	16.41246041
C	10.39283111	14.82600186	17.45840099
H	10.82697485	15.73771806	17.05669285
C	6.10254266	3.17844947	15.42483642
H	6.18174642	2.13702061	15.12564108
C	7.24625791	3.89914805	15.75803082
H	8.22090694	3.41995070	15.72215246
C	8.19780747	8.33660594	17.78897249
H	7.80846141	8.03414575	18.75757404
C	7.38048118	8.26270034	16.65552667
C	7.90203298	8.66634958	15.41807648
H	7.26977619	8.64535540	14.53269221
C	9.22345856	9.08554888	15.30804304
H	9.61672820	9.38319759	14.34090633
C	10.03343897	9.13742129	16.43896973
H	11.06471381	9.46781632	16.35048849
C	9.51451608	8.77623580	17.67978207
H	10.13911407	8.82578581	18.56814271
C	2.43666144	7.98473990	11.73935775
H	2.80177267	7.58847494	10.78781182
H	1.71997649	8.78743052	11.53611763
H	1.91155098	7.16450693	12.23462454
O	5.96640795	11.53681835	16.41142215
H	6.24302325	10.61743792	16.57319021
H	6.27644639	11.79150647	15.50317728

CageMeNH2

Energy: -4500.020826

P	10.43843551	2.38084628	9.23601670
P	8.78540744	1.61528806	2.23663517
P	9.73236509	7.51109088	5.67977257
O	10.57770573	1.27978246	6.53990819
O	11.47398516	5.80782981	7.30994153
O	10.25331449	4.19507639	2.58381781
N	10.09600469	4.00540917	5.86690759
C	12.59866991	3.85793545	6.54397895
C	10.62019103	0.24956773	7.44915761
C	12.54130736	4.91656377	7.46147415
C	11.19607326	1.07334091	5.29993459
C	11.67659134	2.21017085	4.64255505
C	12.34590782	1.95997699	3.43815202
H	12.76011829	2.80298359	2.89055323
C	11.28243877	-0.22770561	4.79824296
C	11.96719445	4.85375363	4.13221706
C	10.54623732	0.59780488	8.80506283
C	10.68696834	-1.07753999	7.01913977
C	11.97447883	-0.41339789	3.60250385
H	12.08646493	-1.40980251	3.18752943
C	11.66367209	7.11582661	7.66344783
C	13.65721865	2.96561446	6.74360483
H	13.76215621	2.12192605	6.06566335
C	11.31400405	5.05349230	2.90447592
C	13.45270205	5.13800110	8.49251256
C	12.51462190	0.67711749	2.92986337
H	13.05547374	0.52612135	1.99935999
C	13.21894824	6.30314498	9.44944997
C	9.74306274	8.95539325	4.53694100
C	9.39485628	2.73343013	0.90576711
C	11.66229289	6.05212689	1.98575209
C	8.69334322	2.77782519	8.78787417
C	10.54396509	-1.33887036	5.52920820
C	10.36201853	2.31274575	11.07300799
C	8.20071055	0.16456458	1.26236121
C	7.19017175	2.43714263	2.67746171
C	12.57859481	7.44430661	8.66841340
C	10.35078382	4.90042027	0.28420298
C	10.02207822	3.94231823	1.24947393
C	10.60696766	-0.43029168	9.74875511
H	10.55281994	-0.18978820	10.80612048
C	13.49761547	6.64412051	3.42703330
H	14.37601710	7.24935635	3.63541831
C	14.58900171	3.13399800	7.76465531
H	15.39998079	2.42054651	7.88357583
C	13.07453040	5.69103926	4.34556540
H	13.64463480	5.57051831	5.26146999
C	14.49430387	4.21860670	8.62931111
H	15.23332748	4.34106871	9.41450978
C	8.85163650	3.55242951	5.19962745
H	8.67926682	4.15731955	4.31124427

H	8.00674576	3.66146674	5.88677575
H	8.95405504	2.51042295	4.90052054
C	10.77488632	-2.07349097	7.99132166
H	10.85012969	-3.11406676	7.69341729
C	8.58455032	9.61912739	4.12567843
H	7.62281465	9.35938757	4.55878488
C	10.89431507	8.07571639	6.99102498
C	6.77412361	3.66815096	2.16281164
H	7.35171451	4.15273888	1.38031672
C	9.17359234	2.29210525	11.80802602
H	8.21684998	2.30098381	11.29289663
C	10.74494961	-1.75382411	9.34541101
H	10.80685810	-2.54193988	10.08991383
C	9.13244395	-0.85678929	1.03673743
H	10.14195154	-0.75362088	1.43029288
C	5.62219261	4.28633535	2.64724135
H	5.31749269	5.24604152	2.23775194
C	4.86243168	3.67788385	3.64159324
H	3.96300373	4.15919944	4.01581855
C	12.78414848	6.83320284	2.25210032
H	13.09356262	7.59985044	1.54968713
C	9.22433156	2.44992958	-0.45230304
H	8.75320351	1.51677544	-0.74507092
C	7.75714219	1.83263839	8.35832992
H	8.03117025	0.78200143	8.30898091
C	6.90052172	0.01508372	0.76990965
H	6.16606238	0.79869951	0.93486459
C	6.42027514	1.83550431	3.68344589
H	6.73741623	0.88456112	4.10843086
C	10.75813977	6.27329132	0.78536732
C	7.47822331	-2.13632262	-0.16489798
H	7.19609528	-3.02875531	-0.71664824
C	8.06805917	7.63646997	6.45854011
C	10.18650859	4.57021546	-1.05962266
H	10.46276840	5.28345730	-1.82892335
C	9.64836129	3.34213916	-1.42928214
H	9.52582045	3.09495027	-2.47961483
C	9.03693090	-1.21908271	5.17912871
H	8.89279998	-1.34686127	4.10019638
H	8.65025293	-0.23467655	5.46252494
H	8.46473861	-1.98681385	5.71277511
C	8.77727574	-1.99399792	0.31878695
H	9.51239324	-2.77537290	0.14601556
C	5.26141939	2.44269371	4.15169261
H	4.67525334	1.95803240	4.92777833
C	6.54189077	-1.13158099	0.06538298
H	5.52768677	-1.23648848	-0.31059792
C	9.20937853	2.25802933	13.20010801
H	8.27915005	2.24462664	13.76164165
C	11.58515826	2.30304407	11.75558550
H	12.51491664	2.33299587	11.18987153
C	9.47796866	7.00289411	1.27276983
H	8.97960000	6.44034295	2.06906473
H	8.77633394	7.12548482	0.43953699
H	9.74180743	7.98997718	1.66766170
C	10.97010575	9.31264584	3.96185462
H	11.87687629	8.79033657	4.25628336
C	8.65303590	10.62184919	3.15951990
H	7.74351066	11.13025129	2.85112087
B	11.59308615	3.71170197	5.25248264
C	11.03586511	10.32053401	3.00838338
H	11.99662789	10.58818802	2.57652858
C	7.79954529	8.28944623	7.66468217
H	8.60722827	8.76084430	8.21792574
C	8.31841640	4.12787934	8.83822044
H	9.04433702	4.87900416	9.14588383
C	11.40757125	7.12809133	-0.30226816
H	11.65220158	8.11934205	0.09005148
H	10.71012727	7.28066799	-1.13089442
H	12.32221435	6.66851785	-0.69223178
C	7.03113548	4.52024012	8.49039645

H	6.75794717	5.57022048	8.53357362
C	7.01475523	7.01536671	5.77295672
H	7.21526810	6.47378193	4.84979701
C	11.02706240	-2.73196887	5.12635759
H	10.45425347	-3.50343182	5.64857124
H	12.09046805	-2.87122572	5.34818913
H	10.86749205	-2.89210416	4.05640870
C	6.10497214	3.57094183	8.06154591
H	5.10459936	3.88032078	7.77085654
C	10.42925724	2.23425059	13.87098699
H	10.45392679	2.20556640	14.95676698
C	14.50609689	6.74007150	10.15334585
H	14.91593386	5.91797627	10.74555059
H	14.30506506	7.55659440	10.85212682
H	15.26521540	7.06800363	9.43545740
C	9.87565736	10.97716475	2.59877570
H	9.92634367	11.76042560	1.84771648
C	6.47217822	2.22967347	7.99392902
H	5.75647297	1.48470580	7.65664923
C	12.19850532	5.84559225	10.52207304
H	11.26465787	5.51623262	10.05606304
H	11.97614472	6.66906213	11.21039751
H	12.60727443	5.00344869	11.09033103
C	11.61862402	2.25197881	13.14487451
H	12.57406623	2.23746861	13.66200864
C	12.75572090	8.79612695	8.95637067
H	13.46489313	9.09805283	9.71996131
C	5.71337043	7.08764519	6.25824999
H	4.90735362	6.60848947	5.70907016
C	11.08961859	9.41904087	7.32608106
H	10.51841979	10.18518592	6.80869477
C	6.49801203	8.34348008	8.15933589
H	6.30112781	8.85308247	9.09867940
C	5.45147287	7.75412319	7.45350829
H	4.43737135	7.80361460	7.84044667
C	12.02241853	9.77759151	8.29061305
H	12.17520199	10.82456874	8.53475013
H	10.10181283	3.61827169	6.81056952
H	10.03417961	5.02075671	5.98373983

CageMeNH_ipr

Generated by Multiwfn

P	2.14022237	30.95218813	14.89361817
P	4.45558016	37.37697215	17.22979588
P	6.48570807	32.28072799	12.47707687
O	1.60853528	33.77977190	15.59788355
O	3.83889548	32.96128063	11.32149360
O	5.23072317	36.82027811	14.30616718
N	3.93824130	33.85202804	14.03568512
C	2.46399584	33.22769873	11.36025413
C	1.36610627	35.14470441	15.40626335
C	5.06278910	36.75810907	12.92266190
C	2.11329446	35.78146978	14.40867515
C	3.98386790	35.99902576	12.43589895
C	2.02375033	34.15295696	12.32039796
C	1.63517115	32.59963489	10.42747445
C	5.97736364	37.84547991	14.84236148
C	0.64017834	34.37733802	12.32360798
H	0.22938989	35.08628565	13.03670870
C	0.54575031	32.99141790	15.97045740
C	3.75323436	36.13559079	11.05745454
H	2.90612019	35.61392998	10.62215095
C	5.45830514	31.21902938	11.37243390
C	6.97122266	38.47503261	14.07573709
C	4.23679657	31.71673425	10.89042623
C	3.44682414	31.00920334	9.97622818
C	0.38502620	35.76750390	16.18007249
C	5.68363414	37.52637981	10.73655435
H	6.34931044	38.06758866	10.07275472
C	-0.50670495	33.54739242	16.70650018
C	1.83008566	37.14098773	14.23018564

H	2.37556578	37.68534620	13.46233174
C	5.97209692	37.43319289	12.09621921
C	4.55051376	36.90982607	10.22339209
H	4.31203195	36.99729840	9.16683602
C	0.58784583	31.63320137	15.62125581
C	4.70567597	34.12273658	15.27651750
H	5.64790291	34.61054272	15.02669568
H	4.92417056	33.17475734	15.77956041
H	4.13443573	34.77811166	15.93636723
C	2.27678972	31.74527199	9.34539752
C	5.73568276	38.21094487	16.17676016
C	-0.22999306	33.73388496	11.44857526
H	-1.29701208	33.93290834	11.49957421
C	0.26595137	32.85487765	10.49589475
H	-0.41523430	32.37250310	9.80287952
C	-1.61087502	32.74203369	16.97477438
H	-2.45676290	33.14667113	17.52073081
C	5.82795578	29.92914419	10.97430781
H	6.75911963	29.50064557	11.33153574
C	7.68614551	39.52520062	14.64403770
H	8.44450427	40.03801429	14.06247242
C	-0.30618387	34.94989464	17.26077527
C	0.84681253	37.79958285	14.96128582
H	0.63723652	38.84922625	14.77295040
C	7.28161405	37.90346665	12.70608469
C	3.84175061	29.71954807	9.63006375
H	3.23717421	29.13406735	8.94558961
C	0.11980133	37.11332445	15.92769687
H	-0.65016024	37.63256622	16.48890656
C	7.73172371	32.99873553	11.26357351
H	8.39856107	33.59630628	11.90283535
C	-0.53533566	30.85737147	15.92759743
H	-0.55453739	29.80703534	15.65368446
C	1.55257973	29.47171589	13.91245251
H	0.97879614	28.79671143	14.56068955
C	6.50998291	39.24524380	16.71503037
H	6.34448147	39.54980260	17.74496924
C	2.85259448	32.69795268	8.26470496
H	3.35930065	32.11919427	7.48410866
H	3.57136862	33.39861731	8.70165107
H	2.03983571	33.27535480	7.81047883
C	3.65104072	38.87344434	18.06687819
H	4.34392209	39.27791647	18.82055347
C	-1.63804181	31.41065524	16.56724310
H	-2.50869768	30.79655336	16.77759052
C	2.91224402	30.16799462	16.43952775
H	3.91236579	29.85977238	16.10042942
C	0.66442855	29.93081051	12.75565518
H	-0.22396216	30.46666825	13.10451159
H	0.33417497	29.06383004	12.17008025
H	1.21453323	30.60109254	12.08684072
C	5.01794326	29.17384730	10.13637121
H	5.31426279	28.16720530	9.85659953
C	7.46846529	31.03844542	13.47272525
H	7.96396060	30.31480684	12.81240956
C	7.46068755	39.91463009	15.96018580
H	8.03244200	40.72930510	16.39488850
C	5.63278567	36.75738305	18.57619675
H	6.26893695	37.59854491	18.88753891
C	2.78106945	28.72121750	13.38465129
H	3.38261300	29.37077767	12.73715058
H	2.46382601	27.85711939	12.78859045
H	3.42497674	28.35326961	14.19101373
C	1.28286451	30.79494330	8.67688271
H	0.47843462	31.35945198	8.19822044
H	0.84131854	30.09835630	9.39789350
H	1.77513424	30.21987693	7.88748890
C	8.56542235	31.98038693	10.48953031
H	9.13987101	31.32268901	11.14978515
H	9.27777236	32.50212522	9.83812547
H	7.92838166	31.35656460	9.85308187
B	3.02933798	34.99665308	13.32361685
C	0.65262872	34.83664305	18.47469770
H	0.83581016	35.83034846	18.89606625
H	1.61554061	34.40797559	18.17865001
H	0.20822785	34.19720999	19.24618979
C	6.99485654	33.95420062	10.32071330
H	6.32110631	33.40068897	9.65521643
H	7.71411306	34.49292293	9.69192143
H	6.39732655	34.69098370	10.86779138
C	8.54523118	31.78140063	14.27095916
H	8.09852947	32.56123489	14.90056715
H	9.28924009	32.25398236	13.62178053
H	9.07282747	31.08060125	14.92867506
C	8.17944664	36.65354670	12.90783484
H	8.40325912	36.19834917	11.93651368
H	7.67667228	35.90551311	13.53047731
H	9.11892699	36.93891829	13.39514004
C	2.18852337	28.95075094	17.01237484
H	1.17125757	29.21082527	17.32583821
H	2.72477270	28.59018931	17.89934274
H	2.13103420	28.12103212	16.30207631
C	-1.61438870	35.58353710	17.73389632
H	-2.06314745	34.98303102	18.53037258
H	-2.33641861	35.68024635	16.91609135
H	-1.42693829	36.57538642	18.15447475
C	3.08319635	31.23816456	17.52271514
H	3.55689457	32.14740717	17.14056127
H	3.70044950	30.84846871	18.34061360
H	2.11032225	31.52047738	17.94129784
C	3.30852098	39.96552708	17.04843770
H	4.19646407	40.42823154	16.61167237
H	2.71528471	40.75060012	17.53343698
H	2.70715414	39.54962138	16.23133041
C	2.35896405	38.42204880	18.76218717
H	1.63154599	38.08793105	18.01390974
H	1.91922277	39.26170143	19.31482248
H	2.51404984	37.60057564	19.46591116
C	6.52583207	35.66023548	17.98933166
H	5.93298069	34.77644557	17.72673814
H	7.27745377	35.35272386	18.72633740
H	7.05094276	35.99829463	17.08932305
C	8.03150109	38.89163715	11.81163153
H	8.99345918	39.15958906	12.25711937
H	7.45328844	39.80660068	11.64455467
H	8.25268132	38.43455582	10.84337512
C	6.51410581	30.29071654	14.40863707
H	7.06611466	29.55755961	15.00906520
H	5.73157140	29.76089811	13.85574362
H	6.02427659	30.99050900	15.09729090
C	4.87738160	36.23340052	19.79906103
H	4.38216041	37.03612057	20.35220040
H	5.57717915	35.74182467	20.48630409
H	4.11894762	35.49517150	19.50866336
H	4.61442245	33.48760211	13.35006223
H	3.31355435	33.05643231	14.22762223

CageNH3

Energy: -4460.688154

P	6.68786648	12.76838265	13.19077210
P	7.27124324	13.58953079	19.18019879
P	5.67513677	7.64801792	16.74635111
O	5.76278389	14.31198593	16.77632469
O	5.21205833	9.84985730	18.61659453
O	5.02077228	10.52474547	14.05142965
C	4.63024542	14.33455018	15.95523550
C	6.02099770	15.44077421	17.51320861
C	5.12536162	16.77169974	15.60351061
C	4.25431608	15.54392368	15.36716977
C	5.13859816	8.65416435	19.29459212
C	5.63617190	16.69719207	17.03359757
C	4.46263328	10.92062057	19.11818589

C	4.02394975	11.86865809	18.18471851
C	6.69733782	15.27523268	18.72669670
C	3.95158636	13.12053287	15.79615854
C	4.21109166	10.98471313	20.49081237
C	4.88402501	8.63097052	20.66734290
C	3.89780826	9.96574163	14.66311406
C	5.07510367	10.53880416	12.68476822
C	5.36827501	7.48673153	18.55211371
C	5.85899193	11.54194934	12.09684647
C	7.43200108	13.74966164	21.00672957
C	6.93038579	16.41147327	19.50789310
H	7.44956557	16.30747917	20.45629037
C	5.86057982	17.80058688	17.85312834
H	5.55748932	18.78901325	17.52366272
C	3.58577634	10.47257984	15.92977467
C	6.65548477	14.25077954	12.10245945
C	8.46353045	12.27897019	13.15772708
C	5.28307284	6.26210865	19.21910497
H	5.45627887	5.34156821	18.67045781
C	6.49264235	17.66053333	19.08753221
H	6.66397762	18.53329555	19.71052984
C	3.25161970	12.91081045	18.70943375
H	2.87750259	13.67316726	18.02980778
C	4.77519963	7.38751875	21.28966785
H	4.55913652	7.33146870	22.35134867
C	6.26947935	14.04436305	21.73246195
H	5.33883246	14.24185335	21.20558676
C	4.38009072	9.58718063	11.92999785
C	4.86995717	9.95977601	21.40450753
C	3.41569040	12.03545525	20.94932921
H	3.18172308	12.12023732	22.00540012
C	9.02645327	13.61280505	18.61613332
C	4.18485070	9.86643085	22.76867503
H	4.70106760	9.14550131	23.40872047
H	3.13516168	9.56779681	22.67610901
H	4.23292700	10.83089129	23.28181100
C	4.96550008	6.21125216	20.57159080
H	4.88654335	5.25107313	21.07279450
C	3.11283941	15.54000170	14.56490091
H	2.77520820	16.45613377	14.09146769
C	3.19202308	8.95714383	14.00917906
C	4.38395849	18.07732920	15.31471187
H	4.06839588	18.11072860	14.26832472
H	5.04392885	18.93555556	15.47025311
H	3.50072302	18.19065371	15.95234337
C	5.92030016	11.59622013	10.70153681
H	6.50218483	12.37972652	10.22372004
C	5.89658726	5.88552654	16.25921053
C	2.93479381	12.99205339	20.06278703
H	2.31924831	13.80881647	20.43064174
C	6.36572034	16.69529285	14.67655952
H	6.91649554	15.76237166	14.83242881
H	7.03675350	17.53607283	14.88610423
H	6.05563897	16.73524413	13.62780586
C	5.39604237	14.75437473	11.74857142
H	4.49558575	14.23461371	12.06964625
C	4.46236460	9.68538236	10.54182223
H	3.93434245	8.97119176	9.91889667
C	7.79996288	14.94700532	11.70922688
H	8.78427316	14.57345845	11.97832118
C	2.48980364	9.87435118	16.55790082
H	2.19892049	10.22677267	17.54488378
C	6.29251650	14.07337361	23.12084097
H	5.38340287	14.30875130	23.66785960
C	2.82700592	13.17828178	14.96315789
H	2.25379334	12.26873328	14.80120765
C	11.19521161	11.67046558	13.26896789
H	12.25474934	11.43275565	13.31015117
C	5.22198506	10.67890043	9.92817967
H	5.26889432	10.73314256	8.84468765
C	6.34771256	10.37346612	21.62809515
H	6.39329268	11.32163222	22.17286053
H	6.86943008	10.50312423	20.67393641
H	6.86554508	9.60133772	22.20873707
C	9.26207341	12.77266790	14.19793049
H	8.81591865	13.39012345	14.97687374
C	9.04527025	11.45880354	12.18645070
H	8.43652385	11.05309721	11.38318278
C	2.10479711	8.40197856	14.68820420
H	1.52524493	7.60660244	14.23116592
C	7.68725287	16.12287266	10.96810138
H	8.58590465	16.65474538	10.66742576
C	10.62202258	12.48099801	14.24583591
H	11.22603595	12.87513941	15.05812865
C	4.73114660	5.16069113	15.97592712
H	3.76158151	5.64838625	16.06529102
C	10.40325124	11.15511780	12.24459712
H	10.84403357	10.51372807	11.48611574
C	3.67374071	8.44820499	12.65403265
C	7.47166992	13.78927981	23.80973476
H	7.48751879	13.80337541	24.89586797
C	2.40714914	14.36036912	14.35863826
H	1.52256363	14.36395855	13.72725430
B	4.34731131	11.74939903	16.59264587
C	8.61106776	13.47242652	21.70159941
H	9.52378253	13.24419762	21.15829833
C	6.43490676	16.60957237	10.60707868
H	6.35006769	17.52392608	10.02660958
C	1.76059915	8.85156801	15.95752801
H	0.91540703	8.40716837	16.47624594
C	9.68568255	12.37792323	18.56375537
H	9.14665451	11.46771982	18.82150709
C	9.71713099	14.76673348	18.23391457
H	9.21569107	15.73029729	18.25153651
C	5.28816242	15.91770858	10.99802621
H	4.30483379	16.29231717	10.72642907
C	8.62712556	13.48844670	23.09574947
H	9.55209419	13.27065591	23.62280820
C	4.80332443	3.82876137	15.58445164
H	3.89063190	3.27719416	15.37619430
C	11.02390726	12.30587094	18.19087369
H	11.52824185	11.34407599	18.17438777
C	4.71377974	7.32514752	12.89029687
H	4.25270672	6.48975630	13.42785042
H	5.55153314	7.68885692	13.49252998
H	5.10330678	6.96395933	11.93153081
C	11.70971267	13.46223550	17.82692060
H	12.75308002	13.40680589	17.52802093
C	7.13585042	5.25623515	16.11663835
H	8.04995040	5.80423315	16.32784295
C	11.04918495	14.68846716	17.83486844
H	11.57544096	15.59199262	17.53887753
C	6.04384038	3.20853480	15.44410153
H	6.10213039	2.17113296	15.12672795
C	7.20720221	3.92642172	15.70620894
H	8.17742177	3.44939850	15.59655009
C	8.26721127	8.28194985	17.81556765
H	7.91516075	7.90825110	18.77379266
C	7.41238526	8.26926543	16.70830679
C	7.88362888	8.76428237	15.48449205
H	7.22025077	8.79192855	14.62246700
C	9.18772741	9.23116443	15.36074144
H	9.53690232	9.61193983	14.40544246
C	10.03452233	9.22747615	16.46588173
H	11.05092566	9.59886951	16.36951450
C	9.56983653	8.75971534	17.69296054
H	10.22489982	8.76154953	18.56068428
C	2.52175768	7.87719580	11.81866016
H	2.89087151	7.47880269	10.86986510
H	1.76335092	8.63924265	11.61090011
H	2.04531929	7.04234908	12.33799120
N	5.96405695	11.54440069	16.46473117

H	6.30418302	11.88915244	15.56204693
H	6.44412352	12.06250825	17.20589857
H	6.23443141	10.56611667	16.55049936

CageNH3_ipr

Energy: -3781.667867

P	16.43627014	5.42161141	5.98950499
P	12.81417574	9.24053023	7.06746579
P	13.93437116	4.80215152	10.75390113
O	13.62428060	8.96845560	9.89453625
O	16.72205158	5.52009678	10.17526911
O	17.10467499	8.24423221	6.49318831
N	15.34962221	7.32739164	8.58581181
H	15.80098970	6.70866171	7.89766918
H	14.65022134	7.88477919	8.08013535
H	14.84808854	6.72072342	9.24300993
C	17.92702845	6.13089456	9.84678064
C	14.48982843	9.09834720	10.98398632
C	16.96301093	9.52987464	7.02063542
C	17.82881892	7.48340685	9.47260756
C	15.82508483	8.73681418	10.77075381
C	16.61506613	9.61146629	8.37627167
C	17.78718830	3.35182899	10.30351495
C	15.35945411	3.63820019	10.56512221
C	16.83126585	8.06737041	5.15908454
C	12.59052149	9.86650361	9.79273883
C	16.43127350	6.78538402	4.74822377
C	17.19352179	10.63244187	6.19612963
C	13.98286838	9.58572941	12.19047451
C	16.64992190	4.16338637	10.33708254
C	12.11863157	10.16678417	8.50576654
C	19.04290005	8.13593448	9.24203031
H	19.02115908	9.18543990	8.95751402
C	13.91336674	5.02095740	12.63332491
H	13.04575274	5.67426630	12.80398271
C	17.60295474	1.97240073	10.42560590
H	18.46802388	1.31624166	10.40766929
C	14.87946329	9.76006761	13.24503471
H	14.53354238	10.14964100	14.19685425
C	16.98688537	9.13086095	4.26235400
C	19.12913778	5.42554371	9.91158282
C	15.18380723	4.20575908	5.32276154
H	15.38750363	3.95959168	4.27230747
C	16.49217509	10.91127597	8.88074553
H	16.22617117	11.04007776	9.92705510
C	15.23250567	2.25347051	10.67788346
H	14.25588507	1.81242838	10.84790157
C	16.67243264	8.91455702	11.87173553
H	17.72315053	8.65464824	11.76262918
C	19.18870149	3.94025785	10.23769057
C	18.10193661	4.61327205	5.65046071
H	18.12234081	3.76902364	6.35576221
C	16.34161216	1.41910239	10.59011945
H	16.22372602	0.34324561	10.67825602
C	16.07823382	6.62169325	3.40468007
H	15.75041932	5.65000940	3.04793162
C	12.58932187	10.41547240	5.63028196
H	11.54369820	10.74345266	5.56183142
C	17.04861239	11.90239511	6.75646483
H	17.21110058	12.78774312	6.15066062
C	12.03682983	10.41952110	10.95265245
C	11.11531753	11.13648027	8.40433535
H	10.73445797	11.41593464	7.42667917
C	20.30726767	6.14105749	9.67512569
H	21.26582845	5.63434036	9.74237079
C	20.27106696	7.48978093	9.35820799
H	21.19594440	8.03293714	9.18443030
C	15.16428817	5.78068634	13.07687051
H	16.06383278	5.16885481	12.93884878
H	15.09092277	6.02987310	14.14229718
H	15.29777574	6.70924405	12.51636661
C	12.43980846	3.70517832	10.46775057
H	12.50187764	2.80580137	11.09335653
C	16.60484871	8.92594826	2.93831527
H	16.68574227	9.73510142	2.21998923
C	16.22048759	9.42702871	13.08422200
H	16.91246519	9.56139201	13.91130628
C	12.48517471	9.83916554	12.28668789
C	17.67178616	10.38942780	4.77223902
C	16.13262646	7.68692220	2.51369705
H	15.83622796	7.54531448	1.47853476
C	10.60445667	11.76270484	9.53529399
H	9.83835473	12.52570349	9.43339544
C	16.69628419	12.04111076	8.09252945
H	16.58462021	13.03286601	8.52246947
C	11.05110061	11.39184148	10.80154629
H	10.61383533	11.86159981	11.67650933
B	16.41689972	8.28980991	9.32259564
C	11.45620564	7.95401620	6.79530012
H	11.83536527	7.34957950	5.95793353
C	13.79164173	4.83608287	5.43100070
H	13.56856191	5.09831959	6.47255780
H	13.02496193	4.13004648	5.08961150
H	13.71061436	5.74788010	4.82982193
C	13.73933345	3.73626834	13.44050758
H	12.79891976	3.22118597	13.22130134
H	13.74425530	3.97287666	14.51190971
H	14.56435162	3.04072695	13.24976322
C	18.30448878	4.08243103	4.23319803
H	17.54797333	3.34292314	3.95155855
H	19.28703230	3.60035990	4.15425626
H	18.27544385	4.89892431	3.50305174
C	20.02033416	3.20995158	9.16194911
H	20.10077510	2.14143501	9.38081129
H	21.03683754	3.60978533	9.11819152
H	19.56060112	3.33046653	8.17629043
C	15.27301251	2.92036944	6.15317706
H	16.22068883	2.39463978	5.99838066
H	14.46070519	2.23714044	5.87786686
H	15.18003840	3.13944833	7.22460240
C	19.85970498	3.74822001	11.61812667
H	19.28535231	4.26071767	12.39668745
H	20.87461597	4.15900687	11.60947971
H	19.91825261	2.68430538	11.87289106
C	11.78063372	8.46845231	12.46588029
H	12.02964957	7.78893037	11.64399837
H	10.69300175	8.60140129	12.49212003
H	12.10772033	8.00760214	13.40489405
C	11.17835304	4.47778641	10.87213987
H	11.15840134	4.70808517	11.94191207
H	10.28720960	3.88188524	10.64165059
H	11.10262708	5.42197034	10.32096596
C	19.21292937	5.59103915	6.04444905
H	19.24145979	6.44329653	5.35463356
H	20.18804626	5.09128566	5.99698398
H	19.07592097	5.97946596	7.05869021
C	13.48826603	11.63740895	5.82594186
H	13.23852737	12.19027445	6.73738757
H	13.38586335	12.32063931	4.97373847
H	14.53937270	11.33662350	5.89641570
C	12.11403126	10.73527321	13.46766416
H	12.42071189	10.27083418	14.40893027
H	11.02978440	10.86953888	13.52093833
H	12.58729760	11.72014184	13.39301662
C	12.95846138	9.67081037	4.34152210
H	13.98542271	9.28787583	4.39477660
H	12.90106474	10.35351752	3.48517723
H	12.28973988	8.82678057	4.14238035
C	11.37918458	7.05474766	8.03165364
H	12.35783650	6.64804949	8.31035058
H	10.70018596	6.21346555	7.84699110
H	10.99386049	7.61313091	8.89382416

C	19.19509288	10.10110519	4.82559205
H	19.72463055	10.97887053	5.21228763
H	19.57173393	9.86872094	3.82291285
H	19.40792667	9.25269661	5.48390013
C	17.43196400	11.59180237	3.85799719
H	16.36683052	11.83824899	3.78694854
H	17.81559140	11.39184749	2.85355041
H	17.96961955	12.46799473	4.22923724
C	12.36841305	3.28117364	8.99882566
H	12.27518142	4.15646273	8.34681433
H	11.49101285	2.64429507	8.83270554
H	13.25665457	2.72297366	8.68788386
C	10.07431228	8.49259369	6.43194865
H	9.67241832	9.11469551	7.23933461
H	9.37937565	7.65697030	6.27977031
H	10.08443089	9.08580540	5.51243839

CagetBuNC

Energy: -4654.950525

P	6.41506294	13.20587207	12.83864831
P	7.23947677	13.63304409	19.62947854
P	6.15241593	7.55787358	16.72931035
O	5.74067459	14.32422777	17.05765056
O	5.31302749	9.78218343	18.64023652
O	4.93444645	10.76036924	13.91003555
C	4.61677493	14.38063945	16.22761469
C	6.02850039	15.44672123	17.80184337
C	5.13030123	16.81013359	15.90824705
C	4.21232984	15.61549454	15.71011779
C	5.26488318	8.54428997	19.24258192
C	5.67123110	16.71773872	17.32321607
C	4.44764540	10.76233041	19.13569232
C	3.99759826	11.73246891	18.23299903
C	6.68746161	15.28688218	19.02835547
C	3.94997667	13.17670262	15.97375939
C	4.06603873	10.71666980	20.48049064
C	4.91243239	8.43534862	20.59761706
C	3.89068274	10.10276627	14.56847321
C	4.84856848	10.85213255	12.53827140
C	5.58545723	7.41485504	18.47788810
C	5.44520746	11.94837807	11.90125486
C	6.81572794	13.81459613	21.41920381
C	6.92304832	16.42793147	19.80518252
H	7.41957749	16.31501551	20.76527479
C	5.90750842	17.82213116	18.13523566
H	5.61945972	18.81121128	17.79531107
C	3.59721410	10.51487927	15.87340555
C	5.90589261	14.72378308	11.91492355
C	8.12131794	12.91266562	12.20282010
C	5.48012462	6.15642359	19.08275421
H	5.71369488	5.27156686	18.49674554
C	6.52313303	17.68317112	19.37690212
H	6.70050777	18.55534042	19.99894591
C	3.06908529	12.64970719	18.74107057
H	2.67901269	13.41746096	18.07812808
C	4.80055351	7.16511022	21.15324291
H	4.50875637	7.05600321	22.19246783
C	5.45154742	13.91497294	21.71755995
H	4.72334258	13.93662638	20.91062718
C	4.18428394	9.85345243	11.80833228
C	4.76810228	9.72199612	21.38957700
C	3.09910972	11.62041511	20.91662983
H	2.75582770	11.59544608	21.94523364
C	9.07383459	13.82353570	19.63711747
C	4.04120699	9.52320732	22.71883992
H	4.59165957	8.82528368	23.35607900
H	3.02422439	9.14237142	22.57542763
H	3.98842235	10.47106862	23.26239141
C	5.07284530	6.02527052	20.40053865
H	4.98163557	5.04052784	20.84896259
C	3.01714295	15.66377269	14.99488027
H	2.65783660	16.60824471	14.60076084
C	3.19372858	9.09150946	13.89874289
C	4.43398539	18.14169338	15.62728356
H	4.08230488	18.17130159	14.59199154
H	5.13472631	18.97325700	15.74423282
H	3.58030555	18.30262632	16.29434121
C	5.30330169	12.06208253	10.51278364
H	5.75055696	12.91392494	10.00751097
C	5.30852873	6.07451990	16.02059335
C	2.59601849	12.58015354	20.04732324
H	1.84870595	13.28898092	20.39406263
C	6.32917946	16.67481217	14.93407393
H	6.84096003	15.71589314	15.06801797
H	7.04655516	17.48400289	15.11288881
H	5.97611312	16.72990175	13.89922748
C	4.55992999	15.09178146	12.03287999
H	3.87671925	14.46226688	12.59725952
C	4.04898122	10.01591380	10.43354072
H	3.52096835	9.26701072	9.85278996
C	6.77036868	15.55299402	11.19731093
H	7.81532175	15.28378404	11.07649060
C	2.49337818	9.89478388	16.47225133
H	2.21640088	10.18761732	17.48149493
C	5.01602799	13.97131581	23.03499445
H	3.95235209	14.04869041	23.24466373
C	2.78387489	13.27779477	15.20472228
H	2.22383524	12.37197321	14.98825576
C	10.80823690	12.46734987	11.51378078
H	11.84711172	12.29366744	11.24775710
C	4.59622319	11.11970788	9.78370158
H	4.48294480	11.23234437	8.70965209
C	6.19409421	10.25168144	21.69235851
H	6.13225742	11.19539699	22.24376715
H	6.75249130	10.43275668	20.76799833
H	6.74141496	9.51763327	22.29482806
C	9.13195832	13.77650828	12.65169495
H	8.87630306	14.62356307	13.28617767
C	8.48291454	11.81606883	11.41393856
H	7.72191253	11.12382242	11.06645429
C	2.07313960	8.54679599	14.52273385
H	1.49101785	7.78056128	14.02188204
C	6.29919797	16.73226242	10.62099757
H	6.98466913	17.36609597	10.06474207
C	10.45971975	13.56434555	12.30006843
H	11.22547930	14.25084291	12.65080475
C	3.90941949	6.12175757	15.99461442
H	3.39152860	6.97535359	16.42458320
C	9.81674156	11.59441960	11.07721562
H	10.07861933	10.73390285	10.46746422
C	3.73860267	8.61457456	12.56303080
C	5.93663163	13.91609660	24.08097294
H	5.59701778	13.95174027	25.11232954
C	2.29934778	14.49966396	14.75026912
H	1.37204648	14.54217293	14.18502468
B	4.35803066	11.74184963	16.63948504
C	7.73443569	13.77408997	22.47003608
H	8.79909847	13.71218720	22.26611795
C	4.96127544	17.09293946	10.74610702
H	4.59749180	18.01219711	10.29564206
C	1.71795537	8.95354969	15.80295297
H	0.84765805	8.52057772	16.28897696
C	9.82807993	12.75313171	20.14163251
H	9.32170727	11.89122186	20.57286182
C	9.75059745	14.90262535	19.05992860
H	9.18993237	15.73607461	18.64730216
C	4.09043092	16.26141498	11.44968222
H	3.04224692	16.52920122	11.55469359
C	7.29439341	13.82032567	23.79245931
H	8.02187877	13.78797262	24.59914040
C	3.17553230	5.09756425	15.41097289
H	2.09008936	5.15383778	15.40178994

C	11.21691888	12.77517899	20.09616082
H	11.78174420	11.94007651	20.50144039
C	4.99689153	7.74607734	12.82569756
H	4.71830040	6.83987964	13.37363102
H	5.73434497	8.29236369	13.42293915
H	5.45680939	7.46233300	11.87214442
C	11.88061599	13.85683569	19.51952221
H	12.96590164	13.87043580	19.47285174
C	5.95627186	4.97714243	15.44953515
H	7.03961664	4.90676644	15.46997164
C	11.14264606	14.91507831	18.99866900
H	11.65034407	15.76060367	18.54244122
C	3.82790787	4.01061878	14.82982315
H	3.25578486	3.21233000	14.36536127
C	5.21789371	3.95483761	14.85400411
H	5.73692639	3.10735139	14.41423895
C	8.57179311	6.78329104	18.06503090
H	8.03386203	6.87200498	19.00419410
C	7.90266405	6.99126500	16.85483147
C	8.63194172	6.88788906	15.66043089
H	8.13625144	7.06676926	14.70775819
C	9.98264184	6.56131954	15.67531819
H	10.52615508	6.47743699	14.73819910
C	10.63935857	6.35687922	16.88781132
H	11.69776202	6.11243110	16.90185069
C	9.93018257	6.47308006	18.07921832
H	10.43381816	6.32038657	19.02995068
C	2.73181593	7.77326318	11.77975584
H	3.17546829	7.41663598	10.84569694
H	1.82255425	8.33707190	11.54472215
H	2.45563873	6.88494395	12.35532715
C	8.47946876	11.24936762	16.19674655
C	5.91498039	11.54113259	16.47076634
N	7.05164058	11.40278639	16.33843772
C	8.98574022	10.50479300	17.43479293
H	8.57415304	9.49354065	17.47271348
H	10.07690301	10.43981588	17.38386534
H	8.70573100	11.04324191	18.34357432
C	9.08150783	12.65409817	16.10206369
H	10.15975763	12.56430760	15.93835515
H	8.64085856	13.20038455	15.26461776
H	8.91142706	13.21218854	17.02568818
C	8.73513589	10.44621926	14.91916336
H	9.81096276	10.26986757	14.82486413
H	8.22273254	9.48213896	14.96542837
H	8.39119258	10.99462046	14.03921000

H2O
Energy: -76.47985644

O	5.96158259	11.54754347	16.41310444
H	6.24743477	10.63775328	16.54611580
H	6.27686023	11.76046599	15.52856939

MeNH2
Energy: -95.94252194

N	10.02386468	3.94538165	5.74571813
C	8.78616681	3.48633962	5.09300117
H	8.69993958	3.95505867	4.10681144
H	7.86011804	3.70094951	5.65115598
H	8.83913311	2.40391295	4.93404745
H	10.09302989	3.52513258	6.66680037
H	9.96624789	4.94542501	5.90846546

NH3
Energy: -56.60686768

N	5.94246392	11.54719867	16.46745443
H	6.31244117	11.88063693	15.58321761
H	6.44876126	12.05326093	17.18680687
H	6.24312854	10.58108151	16.54569712

OpenCage
Energy: -4403.964741

P	-5.64757722	1.40157518	-0.28831858
P	3.62944903	1.59689244	0.38951000
P	2.78181250	-2.76955406	-1.18404387
O	1.02852978	2.62759079	-0.78661778
O	1.24063617	-1.59068480	1.14448227
O	-3.54577234	-0.80657742	-0.21443605
C	-0.31982219	2.96356023	-0.69601888
C	1.91479897	3.65664958	-0.54763973
C	0.27993930	5.16716625	-1.68610773
C	-0.74410372	4.24406336	-1.05924500
C	1.92639417	-2.72282702	1.53539818
C	1.56436349	4.97122858	-0.89598453
C	0.56822068	-0.83260642	2.07383918
C	-0.16865286	0.27425825	1.57575768
C	3.13852819	3.35168334	0.05154018
C	-1.18387708	1.97572926	-0.23523155
C	0.65615568	-1.10515411	3.43944307
C	1.93028588	-3.16792588	2.86136444
C	-2.42720625	-1.35738894	-0.84934329
C	-4.78978932	-1.27590823	-0.59917908
C	2.60874803	-3.43284252	0.52955662
C	-5.88198048	-0.39437914	-0.58680255
C	4.04431601	1.78321165	2.18151334
C	3.99552926	4.41052538	0.37061604
H	4.94634964	4.19280838	0.84968531
C	2.44034312	5.99562236	-0.55274696
H	2.18822557	7.02343206	-0.79140339
C	-1.21067911	-0.66643780	-0.79248269
C	-6.99130778	1.74474371	0.89866532
C	-6.24491845	2.12787360	-1.86521732
C	3.17776998	-4.66615041	0.85698193
H	3.65450839	-5.24798953	0.07314916
C	3.64703045	5.72191734	0.08963985
H	4.31729879	6.53368897	0.35595905
C	-0.67555344	1.17314639	2.53544440
H	-1.20673800	2.05399174	2.19044405
C	2.54586288	-4.38746410	3.14564202
H	2.55539131	-4.74872665	4.16978798
C	2.95085292	1.69219791	3.05398891
H	1.95857915	1.50289635	2.65297963
C	-4.94342022	-2.60314390	-1.03131872
C	1.34478760	-2.34152191	3.99017352
C	0.07529215	-0.20203081	4.33432978
H	0.14526663	-0.39781986	5.40048208
C	5.28572696	1.54652336	-0.41316865
C	0.31144815	-3.17731217	4.77712734
H	0.77841600	-4.05986461	5.22410798
H	-0.49671017	-3.50727989	4.11647349
H	-0.12456829	-2.58757970	5.58878404
C	3.14182724	-5.15367105	2.15463463
H	3.58975441	-6.11290031	2.39540582
C	-2.08298036	4.57372288	-0.86311434
H	-2.44215378	5.56672060	-1.11142225
C	-2.55008813	-2.58878756	-1.50335278
C	-0.17631554	6.62549366	-1.71185648
H	-1.08846159	6.72583001	-2.30677310
H	0.58180145	7.25450832	-2.18723214
H	-0.37049316	7.00874858	-0.70442018
C	-7.15116493	-0.89744026	-0.88936515
H	-8.00408592	-0.22485258	-0.85602502
C	2.42950486	-4.33067613	-2.11854086
C	-0.55992094	0.94720579	3.89927226
H	-0.97375297	1.65403376	4.61187027
C	0.53117922	4.70604105	-3.14525908
H	0.86015504	3.66356791	-3.18147681
H	1.30406444	5.33154047	-3.60537330
H	-0.39320554	4.79732700	-3.72572981
C	-6.67819568	1.56913381	2.25381600
H	-5.67334959	1.25982389	2.53538230

C	-6.21852409	-3.05387290	-1.36114510
H	-6.35524312	-4.07738360	-1.69314329
C	-8.28208237	2.16434371	0.55684091
H	-8.54457270	2.31400166	-0.48701312
C	-0.20401804	-1.10404328	-1.66477330
H	0.74995330	-0.58390612	-1.67611821
C	3.11334086	1.85256830	4.42547684
H	2.24691503	1.77787757	5.07690977
C	-2.52119973	2.34325304	-0.08309406
H	-3.22727876	1.61184769	0.28504804
C	-6.89201606	3.40524168	-4.28102416
H	-7.14656349	3.89791880	-5.21509258
C	-7.32723122	-2.21862021	-1.26531453
H	-8.31731820	-2.59403327	-1.50578254
C	2.49827539	-1.91490086	4.92812229
H	2.11869753	-1.33238985	5.77343409
H	3.22277698	-1.29951599	4.38540562
H	3.01440271	-2.79329486	5.32824543
C	-6.53922744	3.49978644	-1.89538715
H	-6.51634868	4.07936895	-0.97477056
C	-6.25545283	1.41411082	-3.06829419
H	-6.01548641	0.35480632	-3.07274273
C	-1.53215940	-2.97448495	-2.37113908
H	-1.61338025	-3.89936169	-2.93207770
C	-9.23454218	2.39833979	1.54505678
H	-10.23163146	2.72605802	1.26330813
C	-6.87278239	4.12802829	-3.08884673
H	-7.11210867	5.18794232	-3.08929883
C	1.24325984	-5.01817597	-1.82250619
H	0.61017052	-4.67543985	-1.00785850
C	-6.57585827	2.05061651	-4.26555394
H	-6.58011339	1.47942469	-5.18993216
C	-3.71009755	-3.48203894	-1.09455339
C	4.37823257	2.08740402	4.95779695
H	4.51016875	2.20234365	6.03014153
C	-2.96672640	3.62771984	-0.35916739
H	-4.01178925	3.87863850	-0.20237653
B	-0.79074197	0.51363226	0.16475602
C	5.30735493	2.02869165	2.72777832
H	6.17417097	2.11171988	2.07798078
C	-8.91670980	2.20631689	2.88709964
H	-9.66210002	2.38800150	3.65628658
C	-0.38679377	-2.19767552	-2.50010548
H	0.39789302	-2.49368346	-3.18825700
C	6.21275864	0.58342059	0.00984452
H	5.96257310	-0.09062630	0.82587919
C	5.61484547	2.35682401	-1.50558811
H	4.90589841	3.09583780	-1.86715728
C	-7.63554829	1.78644825	3.23857183
H	-7.37663082	1.64056962	4.28363866
C	5.47321226	2.17397375	4.10283945
H	6.46526851	2.36334794	4.50414083
C	0.86824318	-6.13694648	-2.55587578
H	-0.05183372	-6.65852071	-2.30488931
C	7.46065690	0.48917374	-0.59340993
H	8.17238776	-0.24866069	-0.23613289
C	-3.88552696	-4.67782526	-2.03068300
H	-2.97136130	-5.27829499	-2.05015849
H	-4.11897523	-4.36222554	-3.05308444
H	-4.68404890	-5.33444156	-1.67479132
C	7.78992049	1.31853004	-1.66293547
H	8.76381233	1.23848094	-2.13796488
C	3.22244850	-4.79062713	-3.17350861
H	4.15040490	-4.28336876	-3.42115214
C	6.85537995	2.23832182	-2.12764227
H	7.09344883	2.87980613	-2.97192255
C	1.66579430	-6.58934747	-3.60620745
H	1.37287486	-7.46451870	-4.17928983
C	2.84237625	-5.91191347	-3.90946722
H	3.47758150	-6.25888374	-4.72010083
C	5.59975498	-3.11829162	-0.56390005
H	5.32952460	-3.57387415	0.38334893
C	4.60907340	-2.63061727	-1.42046196
C	5.00182714	-1.99777293	-2.60910952
H	4.24520901	-1.56935721	-3.26396422
C	6.34300766	-1.92538146	-2.96861651
H	6.62655890	-1.44080021	-3.89869738
C	7.31808861	-2.45441754	-2.12827250
H	8.36752631	-2.39261381	-2.40305873
C	6.94370061	-3.03122331	-0.91857548
H	7.70148069	-3.41978887	-0.24309128
C	-3.41195287	-4.01371008	0.33182473
H	-4.23379650	-4.65471650	0.67007219
H	-3.29922098	-3.18973176	1.04293762
H	-2.48306524	-4.59517261	0.32719537

tBuNC
Energy: -250.8898552

C	8.46772060	11.25179992	16.19810578
C	5.88374023	11.53174719	16.46702379
N	7.03830549	11.40281847	16.33748774
C	8.98746187	10.50334316	17.43101850
H	8.52586390	9.51456163	17.50584492
H	10.07097555	10.37965495	17.34287195
H	8.77095175	11.06524019	18.34411939
C	9.08574634	12.65197723	16.10710767
H	10.17030535	12.55670529	15.99927160
H	8.69254084	13.19227440	15.24119017
H	8.87170426	13.22888982	17.01139026
C	8.73840978	10.45179152	14.91863596
H	9.81864496	10.32911511	14.79636448
H	8.27645795	9.46197720	14.97583291
H	8.34336299	10.97660711	14.04402220

Cage_ipr
Energy: -3724.869859

P	16.28908538	5.51304780	6.19935964
P	12.98344040	9.25050189	6.91969051
P	13.95161220	4.90745963	10.76430239
O	13.83147032	8.97595794	9.79002824
O	16.78566296	5.63563429	10.14298427
O	17.10281901	8.30739238	6.59893310
C	17.99779387	6.20203114	9.80378943
C	14.58872585	9.03556993	10.94707599
C	16.95665603	9.58402574	7.10003183
C	17.96411280	7.56199930	9.42913460
C	15.93834627	8.67003104	10.83841635
C	16.63331059	9.69661475	8.46350632
C	17.78727834	3.42659940	10.17048081
C	15.36977708	3.75665536	10.46141318
C	16.79566199	8.10792376	5.26735954
C	12.77401057	9.86035286	9.67429244
C	16.34890359	6.82917770	4.90330258
C	17.11309377	10.68229241	6.25253147
C	14.00243135	9.43247929	12.15074308
C	16.67003538	4.26437266	10.24734320
C	12.33337844	10.18581010	8.38049204
C	19.18955817	8.16377849	9.10250238
H	19.19430355	9.20856090	8.80208059
C	14.01336503	4.97940170	12.66288954
H	13.22750216	5.70505846	12.91629380
C	17.57374757	2.04643676	10.21159655
H	18.42273954	1.37281765	10.15752900
C	14.81634841	9.49468673	13.28328751
H	14.39747439	9.81795797	14.23039987
C	16.95639833	9.14978877	4.34993058
C	19.18149894	5.46085622	9.81972006
C	15.08770325	4.26224328	5.49652398
H	15.35370780	3.98996524	4.46729986
C	16.38169915	10.99710174	8.93493419
H	16.12023222	11.13228771	9.98144664
C	15.21561277	2.36912705	10.49800580

H	14.23206897	1.94140604	10.65783709
C	16.69971593	8.69922196	12.01928798
H	17.74828400	8.41435126	11.97202000
C	19.20264497	3.98395121	10.17721621
C	17.96777822	4.68770828	5.90459981
H	18.02172153	3.93460274	6.70494029
C	16.30090597	1.51438556	10.34919579
H	16.15728979	0.43824633	10.37621363
C	15.99541206	6.63772277	3.56313215
H	15.63543621	5.66812976	3.23332590
C	12.59547123	10.38177450	5.47653654
H	11.53653773	10.66795489	5.48443620
C	16.86686581	11.94992381	6.77986073
H	16.96530327	12.82664908	6.14799582
C	12.16469863	10.36865240	10.82809569
C	11.33449455	11.16111670	8.27806451
H	10.97049444	11.45606187	7.29933514
C	20.37549854	6.13221312	9.53940770
H	21.31469310	5.59028792	9.58960572
C	20.39121530	7.47327827	9.18898009
H	21.33078853	7.97124533	8.96854721
C	15.35220638	5.56209560	13.11483346
H	16.16821959	4.85173639	12.93426747
H	15.32410438	5.77284407	14.19078686
H	15.58920084	6.49099690	12.59174350
C	12.44320440	3.81778304	10.49973066
H	12.52132988	2.91190851	11.11316653
C	16.57623890	8.91749248	3.02913916
H	16.66817038	9.70709662	2.29059835
C	16.15721234	9.13043199	13.22294418
H	16.77181248	9.17161142	14.11751900
C	12.51188875	9.72369168	12.16267448
C	17.62028249	10.42752442	4.84060766
C	16.08710887	7.67372667	2.63968826
H	15.79419571	7.50659672	1.60714403
C	10.79220782	11.76513392	9.40578131
H	10.03174318	12.53314161	9.29824845
C	16.48789954	12.10901979	8.10937299
H	16.29244830	13.10254879	8.50228881
C	11.18736600	11.34964025	10.67344027
H	10.71239529	11.78433731	11.54639331
B	16.74081780	8.53584711	9.50702295
C	11.61918543	7.94268255	6.77149127
H	11.96007612	7.30984069	5.93977696
C	13.67856487	4.85955592	5.50764869
H	13.39638361	5.16125266	6.52278002
H	12.94809927	4.11923952	5.15898490
H	13.60485290	5.74158547	4.86265502
C	13.73053176	3.66603445	13.39045909
H	12.72095983	3.28760959	13.20757820
H	13.83410732	3.82066283	14.47198723
H	14.44840305	2.89123837	13.09681152
C	18.14143228	3.99766086	4.55337919
H	17.42100370	3.19038860	4.39363685
H	19.14716117	3.56256149	4.49053602
H	18.04044322	4.71656123	3.73194763
C	20.09912416	3.21055743	9.18914395
H	20.15765308	2.15290934	9.45704925
H	21.12130514	3.59575440	9.20392174
H	19.71230016	3.29146883	8.16843772
C	15.15856831	3.00362011	6.36989067
H	16.12201741	2.49119257	6.28061919
H	14.37303076	2.29709957	6.07589766
H	15.00810337	3.25448141	7.42711130
C	19.77132055	3.82527544	11.60757554
H	19.14980349	4.36519425	12.32996054
H	20.79057578	4.22415233	11.65659901
H	19.79508402	2.76691549	11.88972167
C	11.76244065	8.36845864	12.23454529
H	12.05791396	7.71052419	11.41062242
H	10.68059890	8.53408412	12.17751095
H	11.99583745	7.86496481	13.17958313
C	11.20539317	4.59757972	10.96308294
H	11.24889467	4.85510826	12.02630492
H	10.30540226	3.99239235	10.80132803
H	11.09250618	5.52747096	10.39449977
C	19.08519492	5.70848698	6.13165997
H	19.10442474	6.44283020	5.31750899
H	20.05768062	5.20150974	6.14921736
H	18.96415193	6.24865293	7.07398831
C	13.45415183	11.64496746	5.54899323
H	13.27980436	12.21162096	6.46981692
H	13.23248882	12.30021154	4.69764294
H	14.51801100	11.38865480	5.51189915
C	12.08928545	10.58035558	13.35664933
H	12.31958678	10.06846135	14.29472488
H	11.00806529	10.74222817	13.34585929
H	12.59136641	11.55380010	13.35418918
C	12.88095411	9.61037709	4.18203583
H	13.92239592	9.26635148	4.15579442
H	12.72105575	10.26320992	3.31531280
H	12.23134823	8.73647647	4.06667529
C	11.60226030	7.08847801	8.03931540
H	12.59784339	6.71500779	8.30279595
H	10.93736812	6.22806724	7.89896847
H	11.22401870	7.66515953	8.89249640
C	19.14709788	10.16570838	4.92594344
H	19.65604141	11.05927582	5.30416566
H	19.54010930	9.92550305	3.93183324
H	19.36664606	9.33028977	5.59854887
C	17.38178387	11.61055277	3.90212292
H	16.31542708	11.83719204	3.79631710
H	17.79483325	11.39791592	2.91230357
H	17.89584753	12.50129988	4.27328800
C	12.29878153	3.40573641	9.03399835
H	12.22192118	4.28467815	8.38688868
H	11.38609257	2.81137181	8.90295663
H	13.14429868	2.80565292	8.68734003
C	10.21595960	8.45756569	6.45703763
H	9.85952801	9.13239481	7.24353403
H	9.51941771	7.61110521	6.40488525
H	10.16556010	8.98635440	5.50071694

OpenCage_ipr

Energy: -3724.849915

P	-4.59985256	0.60082963	1.10083657
P	3.28471913	1.62396076	0.84973450
P	2.49181130	-2.98542076	-1.14745726
O	1.22513365	2.65325114	-1.05447698
O	0.42200637	-1.68450519	0.62934163
O	-3.01659489	-1.33235215	-0.48965363
C	0.05865770	2.88559313	-1.75672784
C	1.72957542	3.68486407	-0.28621362
C	0.73605116	5.29524910	-1.93357228
C	-0.23719838	4.16543736	-2.22782644
C	1.40014095	-2.32985393	1.37625941
C	1.38989033	5.00860313	-0.58767470
C	-0.15838129	-0.57104546	1.21975425
C	-0.54347606	0.49436966	0.38440379
C	2.59811878	3.34257471	0.76286716
C	-0.77365753	1.77381897	-1.97606204
C	-0.29137218	-0.53130714	2.61020171
C	1.28437000	-2.35995068	2.77280417
C	-2.27319530	-1.68791873	-1.60314304
C	-4.33611514	-1.74840689	-0.43250524
C	2.47378512	-2.93069967	0.69892808
C	-5.22065279	-0.95965296	0.32124578
C	2.98752554	4.37579451	1.62230803
H	3.64340147	4.16320043	2.46024129
C	1.79237935	6.00049583	0.30495556
H	1.52659744	7.03608784	0.12238991
C	-1.21048346	-0.84025067	-1.95177702

C	3.48112284	-3.50207793	1.48777517
H	4.32771965	-3.97426308	1.00485542
C	2.55692911	5.68234745	1.42331162
H	2.85711259	6.46371374	2.11541409
C	-0.91165777	1.68197651	1.04601559
H	-1.17675873	2.54691394	0.44737075
C	2.32032880	-2.92307224	3.51279431
H	2.26659011	-2.93835208	4.59627911
C	-4.74271602	-2.90665013	-1.10157726
C	0.00627465	-1.80682519	3.37941434
C	-0.69623560	0.66316739	3.20466693
H	-0.78024542	0.72820177	4.28453256
C	-1.14096948	-2.81761519	3.12125626
H	-0.92485227	-3.76301133	3.63094219
H	-1.26290890	-3.01873728	2.05278067
H	-2.08349684	-2.41274122	3.50654610
C	3.42360361	-3.48068119	2.87420637
H	4.22811023	-3.91771265	3.45877439
C	-1.38493796	4.32117802	-3.00815813
H	-1.64526173	5.30128952	-3.39432187
C	-2.63139932	-2.81561111	-2.35015782
C	0.06261475	6.66872344	-1.97285380
H	-0.35473147	6.86389580	-2.96375470
H	0.79445923	7.45853257	-1.78362894
H	-0.74002967	6.74620038	-1.23164303
C	-6.54908119	-1.38969951	0.40143317
H	-7.26744595	-0.81857703	0.97998835
C	-0.97224561	1.78087249	2.42682015
H	-1.26372310	2.71602921	2.89627521
C	1.84800973	5.25553524	-3.01350138
H	2.32928949	4.27306426	-3.04165633
H	2.61000747	6.01252863	-2.79722647
H	1.41376329	5.45673554	-3.99915117
C	-6.08500486	-3.27597114	-1.01877134
H	-6.43382616	-4.16584648	-1.53135754
C	-0.55515509	-1.12545532	-3.16133865
H	0.25046036	-0.47048987	-3.48529960
C	-1.87974826	1.97701633	-2.82094706
H	-2.53280491	1.13513971	-3.03690916
C	-6.98250770	-2.52958626	-0.26472357
H	-8.02261690	-2.83527235	-0.19741986
C	0.11620983	-1.57947032	4.88575943
H	-0.82772605	-1.18964705	5.27771240
H	0.91720573	-0.87569537	5.13633084
H	0.30861960	-2.52601955	5.39886569
C	-1.94286499	-3.05136929	-3.53988342
H	-2.20289705	-3.90615986	-4.15521106
C	-3.68529123	-3.75688978	-1.78488233
C	-2.18944057	3.23255807	-3.32685637
H	-3.06524335	3.37018640	-3.95429359
B	-0.75956723	0.43825041	-1.15947777
C	-0.91154760	-2.21130218	-3.94907180
H	-0.38730231	-2.40900534	-4.87957422
C	-4.27506673	-4.68091646	-2.85233422
H	-3.48910860	-5.29755218	-3.29641936
H	-4.77189977	-4.11529872	-3.64774588
H	-4.99679243	-5.37022896	-2.40683692
C	-3.02122941	-4.63294255	-0.69221259
H	-3.77810010	-5.26705800	-0.21702531
H	-2.55176361	-4.01204362	0.07777352
H	-2.25301348	-5.26955711	-1.14068798
C	-5.91432095	0.93684834	2.39720149
H	-6.92057213	0.89761495	1.96090412
C	-4.99026958	1.77106048	-0.34299148
H	-4.52999631	1.24501382	-1.19378417
C	-6.47695328	1.96097901	-0.63898155
H	-6.59969425	2.53967448	-1.56312304
H	-6.99667366	1.00661890	-0.76857293
H	-6.97328908	2.51677899	0.16442975
C	-4.27721874	3.11896193	-0.19628219
H	-3.20041555	2.99049333	-0.06109579
H	-4.43182240	3.71732316	-1.10232071
H	-4.66251250	3.69437738	0.65099088
C	-5.79030744	-0.12929990	3.49291732
H	-6.54638524	0.04044551	4.26901220
H	-5.92343832	-1.14412569	3.10530533
H	-4.80242012	-0.07608964	3.96713828
C	-5.71045397	2.33147080	3.00121486
H	-5.94452992	3.12925766	2.29169230
H	-6.36923728	2.45556929	3.86923738
H	-4.67658028	2.46809046	3.34249022
C	4.30468350	-2.88218507	-1.67841245
H	4.16917619	-2.63931299	-2.74401174
C	4.94154309	-1.65713472	-1.01853669
H	5.18344766	-1.85205529	0.03189856
H	5.86905932	-1.38289208	-1.53554796
H	4.26466234	-0.79685522	-1.04796817
C	5.23319072	-4.09773666	-1.63016835
H	4.81041622	-4.95912904	-2.15466337
H	6.17572239	-3.83860577	-2.12992643
H	5.48422271	-4.40145447	-0.60984198
C	1.98137537	-4.78153133	-1.44189556
H	2.31203404	-4.97699755	-2.47374247
C	0.45260564	-4.84013559	-1.39346372
H	-0.00989908	-4.14421355	-2.09830137
H	0.10244354	-5.85406189	-1.62482431
H	0.09120646	-4.58868243	-0.38788453
C	2.55710128	-5.85371630	-0.51455564
H	2.23671936	-6.84333866	-0.86491351
H	3.64739679	-5.85107947	-0.46895485
H	2.17635615	-5.72313927	0.50405095
C	4.86594252	1.89464250	-0.16216554
H	5.37985917	0.92648243	-0.08896301
C	3.95149899	1.51352313	2.59849317
H	4.58132924	2.38123820	2.83071023
C	4.50279290	2.13070004	-1.63039281
H	4.03392104	3.11316392	-1.75773849
H	5.40843386	2.11187749	-2.24829262
H	3.80959398	1.37339221	-2.01219271
C	5.80942249	2.98441729	0.34197313
H	5.32707811	3.96775950	0.31274168
H	6.15256784	2.80117607	1.36482680
H	6.69669142	3.02882100	-0.30243382
C	2.78880473	1.45556403	3.59119352
H	2.17834331	0.56327478	3.41312762
H	3.17206175	1.40516922	4.61773725
H	2.13187354	2.32836510	3.51382101
C	4.81229889	0.24955052	2.70962172
H	5.12747928	0.10540159	3.75015084
H	4.24478095	-0.63904290	2.40536495
H	5.71421380	0.30662503	2.09182870

F_CO

Energy: -4526.23970241 Eh

P	6.97035600	12.83005200	13.35887200
P	7.02255400	13.59673900	19.50937000
P	5.80341400	7.75614400	16.53889300
O	5.63789200	14.22413100	16.94420900
O	5.25358600	9.92677100	18.47513300
O	4.97035600	10.82163100	14.18898000
C	4.55068900	14.25816700	16.08595900
C	5.91825800	15.38653000	17.63655600
C	5.08925300	16.66756200	15.65416500
C	4.19029000	15.45539400	15.46063200
C	5.26238900	8.71033100	19.12580200
C	5.58783100	16.63112700	17.08870200
C	4.47539200	10.93097100	19.02464600
C	3.95190200	11.88429300	18.14111600
C	6.56178200	15.26484600	18.87492500
C	3.83213800	13.06735400	15.91296500
C	4.23606700	10.96098600	20.39926400
C	5.03097900	8.64000500	20.50319200

C	3.92485700	10.12895800	14.75176600
C	5.36485600	10.54841500	12.90674700
C	5.53889300	7.57400800	18.35346900
C	6.35419800	11.39243700	12.37651300
C	7.26643300	13.98871800	21.29786800
C	6.82972000	16.43694000	19.58910000
H	7.32969700	16.36928600	20.55035100
C	5.84028400	17.77088700	17.84884800
H	5.57649500	18.74849000	17.45995200
C	3.54268500	10.57308100	16.02828300
C	6.54288600	14.15675600	12.14524900
C	8.79721300	12.68969100	13.21873100
C	5.54931100	6.33383200	18.99901500
H	5.76327900	5.43776200	18.42506900
C	6.45125300	17.67752200	19.09509500
H	6.65059400	18.57576100	19.67188100
C	3.14777700	12.88279800	18.68331600
H	2.71993500	13.63190700	18.02403600
C	5.01526600	7.38040800	21.09979600
H	4.82285600	7.28859900	22.16341100
C	6.10435600	14.15382300	22.06487100
H	5.13065600	14.07693700	21.58509000
C	4.80395500	9.50916200	12.15806800
C	4.94502700	9.94275100	21.28123700
C	3.41252600	11.97114900	20.89654300
H	3.19806000	12.02269000	21.95847800
C	8.75662500	13.44082100	18.90519300
C	4.25997600	9.77326500	22.63851000
H	4.80696100	9.05748400	23.25774200
H	3.22577400	9.42902200	22.53091300
H	4.26136900	10.72014100	23.18514800
C	5.26950700	6.23333400	20.35530000
H	5.26511700	5.26046600	20.83781300
C	3.04473900	15.46222200	14.66559400
H	2.72980100	16.37688100	14.17559400
C	3.29694000	9.06993400	14.10380500
C	4.38023900	17.97872700	15.31095300
H	4.06951400	17.97753600	14.26266100
H	5.06167500	18.82502200	15.43240900
H	3.49907300	18.14143500	15.94067600
C	6.78037600	11.17943300	11.06556400
H	7.52230800	11.84730000	10.63523400
C	5.63142400	5.99703100	16.00878500
C	2.87404500	12.92783200	20.04607400
H	2.23885400	13.71348600	20.44470000
C	6.32753100	16.52862400	14.73376300
H	6.84788300	15.58298700	14.91604800
H	7.02260800	17.35403400	14.92518900
H	6.02187000	16.55197800	13.68291200
C	5.17710600	14.44356600	12.02016100
H	4.45046900	13.88063500	12.60319500
C	5.28483100	9.31567200	10.86210700
H	4.87414500	8.50945300	10.26100400
C	7.46129500	14.90648300	11.40861000
H	8.52420800	14.69583400	11.48329000
C	2.50940500	9.89933100	16.66459300
H	2.19243000	10.22552400	17.65057800
C	6.18362600	14.40714200	23.42871900
H	5.27237800	14.53877300	24.00609900
C	2.70343700	13.11908600	15.09637100
H	2.11696500	12.21663300	14.95534500
C	11.59814000	12.52795600	13.35439800
H	12.68152200	12.46521200	13.40463000
C	6.26320600	10.13501100	10.31180500
H	6.60657400	9.96889800	9.29528000
C	6.39832700	10.42236100	21.52076400
H	6.39885300	11.37758200	22.05598500
H	6.91931600	10.56230000	20.56878000
H	6.94131700	9.67685700	22.11286200
C	9.55760800	13.76899900	13.69442200
H	9.05854100	14.67450100	14.03469700
C	9.46285000	11.52084100	12.83916200
H	8.89664700	10.65972300	12.49780900
C	2.26878900	8.41540800	14.78827100
H	1.76646800	7.57415500	14.31881200
C	7.02143800	15.92989000	10.56958800
H	7.74762200	16.50667700	10.00289300
C	10.94374400	13.69476100	13.74589000
H	11.51479300	14.54589400	14.10627600
C	4.32458500	5.49710400	15.92439400
H	3.48772600	6.14278400	16.18513600
C	10.85201800	11.44207000	12.90899200
H	11.35160400	10.52343700	12.61279400
C	3.69963100	8.61895100	12.70735100
C	7.42702800	14.47870400	24.05630400
H	7.49048800	14.66526400	25.12470300
C	2.30515400	14.30156500	14.48332200
H	1.41605000	14.31537200	13.85969300
C	8.50776800	14.07236800	21.93229600
H	9.42174200	13.95753000	21.35703700
C	5.66334400	16.20811400	10.44895800
H	5.32454300	17.00614400	9.79423500
C	1.88039300	8.81518300	16.05704700
H	1.08021800	8.29239000	16.57252500
C	9.47767400	12.29894100	19.28484500
H	9.02260500	11.56762600	19.94978200
C	9.35320000	14.35007400	18.02731900
H	8.81181800	15.23864600	17.71458600
C	4.74038500	15.45406600	11.17332000
H	3.67697700	15.66258400	11.08973300
C	8.58516600	14.30967400	23.30425000
H	9.55890200	14.37053400	23.78296700
C	4.08936300	4.19343100	15.50631900
H	3.07036200	3.81939100	15.45343600
C	10.77053300	12.08967100	18.82195000
H	11.31617300	11.20298500	19.13285600
C	4.20073400	7.15978700	12.75391100
H	3.42895300	6.49659100	13.15639300
H	5.08312900	7.07569500	13.39510600
H	4.46169300	6.80877500	11.75023400
C	11.35548500	13.00019500	17.94379400
H	12.35944800	12.82787300	17.56603200
C	6.69474500	5.16689900	15.64603000
H	7.71546100	5.53346500	15.70743100
C	10.64147000	14.12604100	17.54695100
H	11.08754100	14.83866500	16.85890900
C	5.15595100	3.37303300	15.13980800
H	4.97272400	2.35708900	14.80119000
C	6.45574300	3.86509000	15.20772600
H	7.29314600	3.23163200	14.92753500
C	8.47314600	8.09612700	17.52620300
H	8.07197900	7.97554700	18.52910400
C	7.62105500	8.01393600	16.42160500
C	8.15749700	8.18018100	15.13675700
H	7.50005000	8.14603700	14.26883600
C	9.52053800	8.38255100	14.96158000
H	9.92370100	8.50019100	13.95985800
C	10.36421600	8.46207300	16.06838000
H	11.42697900	8.64152800	15.93145100
C	9.83588700	8.32591300	17.34823500
H	10.48630100	8.39353000	18.21619700
C	2.46843800	8.70926000	11.77713900
H	2.71902700	8.37600100	10.76546800
H	2.10374400	9.74010600	11.72398100
H	1.65869900	8.07357000	12.14730100
C	4.22562100	11.78776100	16.64580400
O	8.98842400	11.44040800	16.07911400
C	7.93468400	11.37283800	16.48683200
H	5.30958100	11.65690300	16.52260000

R_acn

Energy: -4531.77380382 Eh

P	6.17948000	13.20195200	13.14312900
P	6.82307600	13.48387800	19.48863700
P	5.90423200	7.89097100	16.62432700
O	5.29890400	14.19359000	17.04395400
O	4.92372700	9.98041000	18.48304000
O	4.61494200	10.88080100	14.10915200
C	4.40825200	14.29408400	15.98372500
C	5.98575300	15.29633100	17.48496500
C	5.16735800	16.70378500	15.52365800
C	4.36130900	15.45543300	15.20008000
C	5.38055800	8.91004700	19.20899100
C	5.93322300	16.52543100	16.82033900
C	4.30578100	11.05035800	19.11519400
C	3.69057200	12.00599900	18.28575600
C	6.76235000	15.12982700	18.65171000
C	3.59413200	13.17399700	15.73238300
C	4.33329500	11.16607800	20.51195000
C	5.36344600	8.89147900	20.60686400
C	3.77147400	9.99312200	14.76190800
C	4.98582400	10.66220500	12.80674700
C	5.88039800	7.81618500	18.46989800
C	5.74271600	11.68115100	12.18936900
C	7.15820300	14.00800800	21.22457300
C	7.45118300	16.23478900	19.15449100
H	8.01877500	16.12978000	20.07421700
C	6.65597000	17.59500300	17.35277000
H	6.63574800	18.55316700	16.84188700
C	3.28479400	10.39253300	16.02047100
C	6.31916500	14.42568900	11.77135000
C	7.95997200	12.92144000	13.55893000
C	6.32550700	6.69162500	19.16649700
H	6.67581500	5.82821700	18.60892300
C	7.40304500	17.46553500	18.51383400
H	7.94802800	18.31503600	18.91447200
C	3.21953400	13.15743700	18.93134200
H	2.76735200	13.94037000	18.32448800
C	5.83627100	7.74962400	21.25722300
H	5.84069800	7.72277400	22.34296700
C	6.21810000	14.83440800	21.85894800
H	5.38922900	15.24130300	21.28486100
C	4.64491700	9.49496900	12.11682800
C	4.90279300	10.08636600	21.41925700
C	3.82310800	12.32752400	21.08734300
H	3.86605900	12.45469500	22.16528700
C	8.48314100	12.84100500	18.98225200
C	3.81011800	9.64561500	22.41883600
H	4.18313300	8.87483500	23.09988400
H	2.94374400	9.24516700	21.88231700
H	3.47792200	10.49108000	23.02791900
C	6.30557900	6.65027800	20.55415300
H	6.65850300	5.76972000	21.08257900
C	3.54822200	15.45460300	14.06919500
H	3.52307800	16.32998100	13.42642500
C	3.45919900	8.75323400	14.18728200
C	4.20575800	17.90671800	15.64785900
H	3.65742400	18.06199700	14.71463500
H	4.75305600	18.82864800	15.86497500
H	3.48107300	17.73564800	16.45064900
C	6.11741300	11.51505700	10.85528400
H	6.66750600	12.30810100	10.35769200
C	5.78148200	6.09543200	16.21918100
C	3.29592900	13.34140100	20.30256800
H	2.92847900	14.25607200	20.75997000
C	6.18413500	16.97236500	14.39163700
H	6.88810200	16.13882200	14.30931400
H	6.75177000	17.88801100	14.59005800
H	5.67759500	17.08170800	13.42743300
C	5.18450100	14.66410800	10.98080000
H	4.29994800	14.04562100	11.11327000
C	5.05726100	9.36979200	10.78848500
H	4.81002900	8.46486000	10.24102000

C	7.43801200	15.24495300	11.59009700
H	8.32677600	15.09115900	12.19520200
C	2.56660700	9.42021500	16.72972000
H	2.19858500	9.67218800	17.72303500
C	6.33571500	15.14433100	23.20788100
H	5.60143800	15.79466600	23.67612800
C	2.81975600	13.23558600	14.56580100
H	2.20052300	12.37674500	14.31153600
C	10.64792000	12.74823300	14.32657200
H	11.69146800	12.68257100	14.62355000
C	5.77899700	10.36755300	10.15074000
H	6.08027500	10.25065100	9.11402500
C	6.11815900	10.64514200	22.19111200
H	5.84289300	11.52981000	22.77386000
H	6.90827800	10.93430100	21.49150000
H	6.52061500	9.89168100	22.87669500
C	8.34849500	13.22997700	14.86566500
H	7.59732800	13.54350400	15.58808400
C	8.93399200	12.50492100	12.64334100
H	8.65140800	12.25378600	11.62461400
C	2.71949600	7.84521200	14.94109700
H	2.49502100	6.86556900	14.52889200
C	7.42724900	16.26565400	10.64086000
H	8.30856100	16.88943500	10.51657900
C	9.68526300	13.14307800	15.24889900
H	9.96731100	13.38164800	16.26937000
C	4.64722900	5.39396400	16.65515200
H	3.93471000	5.88406300	17.31433900
C	10.26987700	12.42599800	13.02263200
H	11.01806500	12.10858000	12.30074900
C	3.90056400	8.35639900	12.78690900
C	7.38770900	14.62130600	23.96023300
H	7.47792400	14.86001600	25.01625100
C	2.80102700	14.33833600	13.72709200
H	2.19486800	14.33801200	12.82519700
B	3.26282300	11.88879900	16.70266200
C	8.20765800	13.48826400	21.98890000
H	8.94477400	12.83745300	21.52752100
C	6.30057700	16.48535000	9.85528600
H	6.29443900	17.27908200	9.11351600
C	2.29830500	8.15821800	16.22421200
H	1.74789500	7.43089200	16.81497900
C	8.54234800	11.49718800	18.60283500
H	7.62609600	10.91207700	18.55290400
C	9.66915400	13.58406200	19.02716000
H	9.64326400	14.63235100	19.31141400
C	5.17783800	15.67555100	10.02867100
H	4.29121500	15.83564600	9.42075200
C	8.31985700	13.79247200	23.34462000
H	9.14441900	13.37835800	23.91897700
C	4.42545100	4.08049600	16.26081100
H	3.54521800	3.55307800	16.61911800
C	9.76186300	10.89979300	18.29066500
H	9.78632600	9.85708000	17.99097300
C	4.84774600	7.13842400	12.86438000
H	4.37243600	6.30111100	13.38482400
H	5.75889100	7.40027300	13.41107600
H	5.13095600	6.80499700	11.86026000
C	10.93617000	11.64123500	18.35704400
H	11.88820900	11.17574100	18.11536800
C	6.67468200	5.44530800	15.36146300
H	7.55563700	5.96737000	14.99952100
C	10.88802500	12.98654300	18.72370500
H	11.80242100	13.57262200	18.76875800
C	5.32403300	3.44167200	15.40593600
H	5.14856000	2.41597100	15.09377600
C	6.44643500	4.13067300	14.95852100
H	7.15446500	3.64507100	14.29206900
C	8.75735300	7.53012600	16.84576100
H	8.56969700	6.80521300	17.63301000
C	7.68823300	8.21448400	16.25495500

C	7.95662700	9.15149600	15.25306600
H	7.13427700	9.69851700	14.79572700
C	9.26422700	9.38742000	14.83303700
H	9.45406600	10.12280800	14.05773400
C	10.31701000	8.68660900	15.41087400
H	11.33704700	8.86768700	15.08199000
C	10.06203900	7.75912400	16.42160100
H	10.88277900	7.21567800	16.88280000
C	2.65250700	7.98861900	11.95300400
H	2.93041700	7.69043800	10.93777100
H	1.97174000	8.84380100	11.89041300
H	2.11567900	7.15150900	12.40807600
C	0.57991000	12.20742500	16.95587900
C	-0.84197700	12.37360300	17.09030000
H	-1.34297000	11.42437600	16.87760600
H	-1.07908000	12.68918600	18.11080100
H	-1.18947800	13.13416300	16.38455300
N	1.71464000	12.07510200	16.84680200

R_cn

Energy: -4492.03491718 Eh

P	6.17577300	13.21751600	13.13824000
P	6.80635700	13.48438400	19.50880800
P	5.87843100	7.87233500	16.62306700
O	5.30390800	14.19639500	17.05769700
O	4.92411100	9.96502300	18.48290600
O	4.62370100	10.88818100	14.09941100
C	4.41607400	14.29486800	15.98393300
C	5.99800600	15.28967500	17.49064300
C	5.19464400	16.70186500	15.52651200
C	4.38828400	15.45496400	15.19707600
C	5.39656200	8.90783500	19.20604000
C	5.96210200	16.51868000	16.82105000
C	4.31066100	11.04683000	19.11803900
C	3.68792200	11.99548600	18.28800800
C	6.77422900	15.12448400	18.66010600
C	3.59596000	13.17923800	15.73873500
C	4.35601000	11.16248900	20.51461400
C	5.39879700	8.89239400	20.60577300
C	3.78064500	9.99060700	14.75777500
C	5.00849400	10.66571100	12.80852500
C	5.89882800	7.81152300	18.46851600
C	5.76792500	11.68511200	12.19042300
C	7.15421600	14.01826500	21.23967300
C	7.47743200	16.22289400	19.15637500
H	8.04324000	16.11541900	20.07707800
C	6.69703400	17.58199700	17.34846900
H	6.68503300	18.53809600	16.83294100
C	3.28453300	10.39428500	16.01003600
C	6.31565300	14.43023500	11.75628200
C	7.95854100	12.95860000	13.56847800
C	6.36925000	6.69703700	19.16397400
H	6.72138200	5.83494000	18.60529500
C	7.44417500	17.45126700	18.50980700
H	7.99827800	18.29700100	18.90640600
C	3.22087300	13.14493900	18.94237900
H	2.73664200	13.91108300	18.33995600
C	5.89314100	7.75920100	21.25458300
H	5.90719300	7.73736800	22.34059100
C	6.21919600	14.85445900	21.86955100
H	5.38874000	15.25602000	21.29411600
C	4.68464900	9.49241200	12.11684600
C	4.92612300	10.08088600	21.41974100
C	3.85229000	12.32391900	21.09519400
H	3.90036500	12.45076200	22.17325200
C	8.45815300	12.81359300	19.00470000
C	3.82809000	9.62635300	22.40795300
H	4.19845200	8.84885600	23.08385700
H	2.96694000	9.23069100	21.85984400
H	3.48834400	10.46634500	23.02059800
C	6.36912400	6.66186600	20.55211200
H	6.73908100	5.78804700	21.08046100
C	3.58447700	15.45873300	14.05990400
H	3.56691000	16.33381900	13.41595300
C	3.48618900	8.74761200	14.18002900
C	4.23190400	17.90344300	15.66026900
H	3.68390500	18.06365700	14.72759400
H	4.77738900	18.82472900	15.88755800
H	3.50578500	17.72167000	16.45922000
C	6.16157500	11.51366500	10.86275800
H	6.71153500	12.30829300	10.36734400
C	5.76649500	6.07175900	16.23740900
C	3.31463600	13.33343100	20.31121600
H	2.93754100	14.24378900	20.77021100
C	6.20941700	16.98097400	14.39507500
H	6.91427000	16.14864100	14.30591500
H	6.77676300	17.89653600	14.59691000
H	5.70085500	17.09470300	13.43254800
C	5.18350700	14.64818000	10.95561000
H	4.30438700	14.02289100	11.09151200
C	5.11490600	9.36298000	10.79504400
H	4.87724200	8.45394400	10.24971400
C	7.42482900	15.26285300	11.57440900
H	8.31039500	15.12750100	12.18853500
C	2.56635900	9.41381500	16.71013300
H	2.16563500	9.67713700	17.68705200
C	6.34057500	15.17514200	23.21542200
H	5.60805000	15.83069900	23.67926100
C	2.82778400	13.25015800	14.56728400
H	2.18017700	12.40783300	14.33175800
C	10.63704800	12.79151400	14.37432400
H	11.67620100	12.72672300	14.68701400
C	5.83984000	10.36039200	10.15961700
H	6.15396200	10.23993800	9.12693400
C	6.13172300	10.64042900	22.20648100
H	5.84634400	11.52033700	22.79135100
H	6.92600700	10.93891500	21.51531300
H	6.53368200	9.88616800	22.89202500
C	8.32500700	13.24253900	14.88703200
H	7.55949700	13.53257400	15.60420800
C	8.95118500	12.57113900	12.65986900
H	8.68593100	12.33805600	11.63219600
C	2.74978000	7.83258200	14.92824100
H	2.53166200	6.85200400	14.51405900
C	7.40839900	16.27425700	10.61516900
H	8.28240300	16.90888500	10.49258100
C	9.65653600	13.15865700	15.28931200
H	9.91973100	13.37636700	16.31943900
C	4.64346000	5.36887600	16.70077500
H	3.93790100	5.86506900	17.36287700
C	10.28197600	12.49530300	13.05771500
H	11.04375800	12.19881300	12.34090900
C	3.93267100	8.35528500	12.77981700
C	7.39132700	14.65375100	23.97113200
H	7.48338300	14.89991000	25.02543700
C	2.82819700	14.34693800	13.72161900
H	2.21604100	14.35166300	12.82337500
B	3.23079600	11.89132700	16.70483800
C	8.20151000	13.49944500	22.00791500
H	8.93342300	12.83938400	21.55138400
C	6.28486100	16.47234800	9.81939600
H	6.27332400	17.25975100	9.07075600
C	2.31597400	8.14791800	16.20694500
H	1.75528100	7.42285800	16.79141900
C	8.48903400	11.47467000	18.60509600
H	7.55960100	10.91170600	18.54545300
C	9.66016900	13.52985000	19.06082200
H	9.65560100	14.57449600	19.35970500
C	5.17094800	15.65030100	9.99401300
H	4.28496900	15.79497600	9.38118900
C	8.31771100	13.81443500	23.36099800
H	9.14009200	13.39896800	23.93785600

C	4.42140400	4.05023300	16.32509000
H	3.54833200	3.52429000	16.70275400
C	9.69557700	10.85600200	18.28348700
H	9.69773400	9.81783300	17.96718000
C	4.87268400	7.13143900	12.85389600
H	4.38903800	6.29424000	13.36653500
H	5.78118200	7.38516500	13.40908700
H	5.16152600	6.80145500	11.84982200
C	10.88560600	11.57099100	18.36062400
H	11.82724100	11.08892900	18.11014400
C	6.64640000	5.41580800	15.37011800
H	7.51667000	5.93897800	14.98464800
C	10.86633200	12.91119200	18.74837300
H	11.79309400	13.47719600	18.80129500
C	5.30791800	3.40540800	15.46170200
H	5.13118500	2.37572100	15.16315900
C	6.41806900	4.09537800	14.98602900
H	7.11567600	3.60675900	14.31053800
C	8.74338800	7.52394000	16.75793000
H	8.58232600	6.77126100	17.52500400
C	7.65234900	8.21345200	16.21456900
C	7.88741400	9.18625200	15.23891200
H	7.04784300	9.73767700	14.81984200
C	9.18224500	9.45436100	14.79913300
H	9.34492200	10.21859700	14.04596100
C	10.25655900	8.74906900	15.32987000
H	11.26686800	8.95646800	14.98627300
C	10.03554100	7.78386700	16.31309800
H	10.87320500	7.23649800	16.73831500
C	2.68657600	7.99816300	11.93757800
H	2.96656000	7.70655300	10.92034200
H	2.00961300	8.85664200	11.88114800
H	2.14464000	7.16136900	12.38723900
C	1.61940800	12.08540200	16.85422300
N	0.46988600	12.22325300	16.96125800

R_CO

Energy: -4512.39126994 Eh

P	6.18834100	13.20269800	13.12821800
P	6.82188400	13.49948200	19.50171000
P	5.89511700	7.86860900	16.63632100
O	5.32170300	14.20546800	17.05100900
O	4.94387300	9.96236700	18.49261600
O	4.64027900	10.87627000	14.09113000
C	4.43683900	14.30610900	15.99333600
C	6.00790100	15.31186600	17.49226100
C	5.17864700	16.71657000	15.53246500
C	4.37144700	15.46762200	15.21494200
C	5.40282900	8.89190400	19.22247400
C	5.95314900	16.53816200	16.82493600
C	4.32782900	11.02736700	19.12287600
C	3.73535700	11.99407100	18.28725800
C	6.77922300	15.14410400	18.66065000
C	3.64353400	13.17270700	15.72792500
C	4.33477200	11.14347800	20.51783500
C	5.38767200	8.87991800	20.61937200
C	3.79914500	9.99063800	14.73804700
C	5.01013900	10.65840000	12.78538700
C	5.89791500	7.79827700	18.48243100
C	5.76360400	11.68004900	12.17125500
C	7.15504200	14.02338100	21.23718600
C	7.47050200	16.24815400	19.16170300
H	8.03619700	16.14441400	20.08277900
C	6.67883300	17.60701900	17.35519200
H	6.65961800	18.56496900	16.84399600
C	3.33375800	10.38168400	16.00874600
C	6.31765400	14.43100700	11.76053900
C	7.96808000	12.93298900	13.55094700
C	6.35172100	6.67798600	19.18001500
H	6.70025600	5.81295400	18.62381100
C	7.42627400	17.47667900	18.51609200
H	7.97359000	18.32570500	18.91443100
C	3.23227500	13.13788700	18.92370300
H	2.78872400	13.92229400	18.31089200
C	5.87006900	7.74263600	21.27099700
H	5.87860400	7.71809300	22.35669400
C	6.21111400	14.84818900	21.86793400
H	5.38205200	15.25229900	21.29179900
C	4.66773700	9.49127600	12.09798900
C	4.91301200	10.07209700	21.42914400
C	3.80257000	12.30024100	21.08531800
H	3.82580700	12.42507800	22.16398500
C	8.47198700	12.83514700	18.99463200
C	3.82318200	9.62166900	22.42707300
H	4.20477800	8.85662000	23.10952400
H	2.96195100	9.21052400	21.89064600
H	3.48103000	10.46374800	23.03522500
C	6.34194100	6.64407400	20.56814000
H	6.70261500	5.76758700	21.09800700
C	3.54393600	15.46267000	14.09313900
H	3.50185300	16.34185400	13.45679100
C	3.46837800	8.75793400	14.16335100
C	4.21766300	17.91898000	15.66273600
H	3.66304500	18.07473400	14.73336100
H	4.76763100	18.84024300	15.87500100
H	3.49870000	17.74923600	16.47074800
C	6.14008500	11.51457000	10.83766400
H	6.68783700	12.30896600	10.33961800
C	5.76740500	6.07344900	16.23584500
C	3.27534500	13.31134800	20.29760700
H	2.88284000	14.21585300	20.75289300
C	6.18673700	16.98171600	14.39208700
H	6.88983200	16.14781900	14.30580000
H	6.75572700	17.89754300	14.58486000
H	5.67244300	17.08991500	13.43179000
C	5.17830100	14.66512800	10.97555000
H	4.29717000	14.04195100	11.10985100
C	5.08327700	9.36595700	10.77054400
H	4.83749000	8.46136200	10.22203100
C	7.43175300	15.25615100	11.57754800
H	8.32348900	15.10554100	12.17905200
C	2.59015600	9.42639800	16.71632400
H	2.23201600	9.67932600	17.71394000
C	6.32537000	15.16040600	23.21663100
H	5.58856400	15.80933800	23.68268400
C	2.84582900	13.23135700	14.57623400
H	2.23679900	12.36478800	14.32001400
C	10.65199900	12.76158800	14.33231600
H	11.69401900	12.69645600	14.63458800
C	5.80546000	10.36482600	10.13474300
H	6.10884000	10.24746400	9.09874600
C	6.11993700	10.64933800	22.20042200
H	5.83158300	11.53267000	22.77891200
H	6.90834000	10.94538000	21.50170900
H	6.52975100	9.90384100	22.89017900
C	8.34818600	13.23163400	14.86246700
H	7.59228600	13.53632600	15.58365100
C	8.94867000	12.52850300	12.63688600
H	8.67289100	12.28571600	11.61431000
C	2.71195700	7.86172900	14.91685600
H	2.46948700	6.88804700	14.50119700
C	7.41185100	16.27890200	10.63069900
H	8.28930600	16.90760600	10.50430300
C	9.68299300	13.14580200	15.25254200
H	9.95838700	13.37622500	16.27673000
C	4.63332200	5.37782900	16.68169800
H	3.92615700	5.87250800	17.34353700
C	10.28255100	12.45016600	13.02330800
H	11.03584900	12.14172200	12.30295200
C	3.91597800	8.35556700	12.76675800
C	7.37719900	14.64074900	23.97146600
H	7.46454200	14.88095700	25.02734700

C	2.80042900	14.34399900	13.75197000
H	2.17692700	14.34774000	12.86275700
B	3.39183100	11.87098700	16.69010700
C	8.20419000	13.50668600	22.00369700
H	8.94362400	12.85690200	21.54455900
C	6.28090400	16.49434600	9.85014100
H	6.26777700	17.28979300	9.11035600
C	2.29221100	8.17568300	16.19991500
H	1.72106300	7.45924400	16.78306700
C	8.51223900	11.49226100	18.60918200
H	7.58805800	10.91995400	18.55834300
C	9.66754900	13.56264100	19.04050300
H	9.65606600	14.60979400	19.32995300
C	5.16281200	15.67874300	10.02582200
H	4.27305900	15.83601300	9.42187400
C	8.31262400	13.81313200	23.35922700
H	9.13674800	13.40177600	23.93611500
C	4.40492500	4.06366900	16.29354000
H	3.52523800	3.54033200	16.65888800
C	9.72293700	10.88025400	18.29146700
H	9.73356600	9.83848600	17.98748800
C	4.85946500	7.13573400	12.85763600
H	4.37584400	6.30102700	13.37473900
H	5.76578100	7.39628200	13.41301400
H	5.15161600	6.79959600	11.85711000
C	10.90688000	11.60627200	18.35801300
H	11.85209700	11.12944300	18.11184300
C	6.65304800	5.41829900	15.37436700
H	7.53306700	5.93657700	15.00476400
C	10.87756500	12.95031600	18.73109500
H	11.79979100	13.52387000	18.77671200
C	5.29625100	3.41929400	15.43529900
H	5.11549000	2.39312000	15.12788300
C	6.41785200	4.10293100	14.97780900
H	7.11973500	3.61250700	14.30845000
C	8.75205200	7.51686500	16.80401200
H	8.58079500	6.77289500	17.57716100
C	7.67010600	8.20558000	16.24207700
C	7.91790800	9.16597700	15.25721900
H	7.08537900	9.71612300	14.82281500
C	9.21784500	9.42230200	14.82606900
H	9.39172000	10.17562700	14.06443100
C	10.28355000	8.71828800	15.37557100
H	11.29779500	8.91540500	15.03817000
C	10.04920200	7.76631600	16.36835200
H	10.88017300	7.21968300	16.80680600
C	2.67228800	7.98851900	11.92673500
H	2.95696700	7.68493500	10.91520600
H	1.99429500	8.84504500	11.85539600
H	2.13001200	7.15445400	12.38067300
C	1.79507700	12.06129600	16.83611300
O	0.67106200	12.19402400	16.93824300

R_h2o_2
Energy: -4475.49421046 Eh

P	6.19568500	13.20797900	13.14876500
P	6.84640300	13.49390100	19.47002300
P	5.90646900	7.87646700	16.62846900
O	5.32433200	14.19104500	17.02098300
O	4.94026700	9.96515500	18.48374000
O	4.64870600	10.87678400	14.11268800
C	4.41257100	14.28853700	15.98079000
C	5.99324300	15.30296800	17.46926900
C	5.13594800	16.70739900	15.52229700
C	4.33551300	15.45333000	15.20657300
C	5.38201000	8.88937600	19.21271600
C	5.91577600	16.53432200	16.81219500
C	4.33243100	11.03914700	19.11121700
C	3.74184800	12.00224500	18.27200700
C	6.77606700	15.13973000	18.63192600
C	3.61246300	13.15525200	15.73862600

C	4.34764300	11.15575700	20.50707200
C	5.35601700	8.87189400	20.60977300
C	3.79022000	9.99689700	14.75716300
C	5.00568100	10.66921700	12.80395100
C	5.87383900	7.79283400	18.47392300
C	5.75474300	11.69395300	12.18748700
C	7.17727000	14.01663700	21.20698900
C	7.45578100	16.25082600	19.13385400
H	8.03027900	16.14967700	20.04970500
C	6.62897300	17.61040100	17.34445100
H	6.59243400	18.57123300	16.83963800
C	3.29867700	10.40102900	16.01350600
C	6.33710800	14.44068400	11.78546700
C	7.97476300	12.91884900	13.56227400
C	6.30265700	6.66271700	19.17151100
H	6.64746300	5.79624800	18.61515500
C	7.38828500	17.48323900	18.49798200
H	7.92681800	18.33722600	18.89773000
C	3.29155200	13.16659000	18.91352800
H	2.90768700	13.98758600	18.30426800
C	5.81309800	7.72446800	21.26173200
H	5.81160700	7.69597600	22.34743200
C	6.24293600	14.85084000	21.83955500
H	5.42139900	15.26906900	21.26313600
C	4.65929300	9.50663300	12.10931400
C	4.90424200	10.07318200	21.41860900
C	3.84185200	12.32263500	21.07729500
H	3.87326400	12.44889800	22.15556100
C	8.50737700	12.85296000	18.96741400
C	3.80660600	9.64615300	22.41815100
H	4.17268200	8.87524600	23.10253400
H	2.93752300	9.25067200	21.88252300
H	3.48084000	10.49616900	23.02430300
C	6.27413200	6.62107500	20.55924400
H	6.61443800	5.73625900	21.08879200
C	3.50329400	15.44631500	14.08873200
H	3.45492900	16.32464700	13.45161500
C	3.46866400	8.76339800	14.17564200
C	4.16827400	17.90432500	15.65343000
H	3.61147100	18.05638900	14.72478600
H	4.71210200	18.82942800	15.86464600
H	3.45138600	17.73052700	16.46257000
C	6.12030000	11.53726300	10.84969200
H	6.66530300	12.33459500	10.35328100
C	5.77927100	6.08360400	16.21441500
C	3.34151100	13.34515100	20.28817600
H	2.98763100	14.26746000	20.74003200
C	6.14107000	16.97912000	14.38062500
H	6.85191300	16.15169700	14.29668000
H	6.70185000	17.90050900	14.57154200
H	5.62606200	17.07969900	13.41978200
C	5.19836100	14.69565000	11.00623500
H	4.30899400	14.08445100	11.14111800
C	5.06359600	9.38998000	10.77763900
H	4.81475100	8.48796800	10.22621900
C	7.46251400	15.25007300	11.60139500
H	8.35470900	15.08267800	12.19785200
C	2.56175100	9.43511100	16.71454700
H	2.18580200	9.69045600	17.70254100
C	6.35769300	15.15556300	23.18995200
H	5.62827100	15.81226700	23.65683600
C	2.82831700	13.20853700	14.57620400
H	2.25485900	12.32395500	14.29149900
C	10.66399500	12.72880100	14.32001000
H	11.70813200	12.65630100	14.61325000
C	5.77974800	10.39276100	10.14130300
H	6.07470700	10.28215500	9.10210200
C	6.12411800	10.62629900	22.18800300
H	5.85543800	11.51467000	22.76835700
H	6.91599300	10.90768800	21.48702000
H	6.52093600	9.87149800	22.87526100

C	8.37160400	13.23801900	14.86389800
H	7.62655000	13.56555300	15.58632800
C	8.94050300	12.48317000	12.64696400
H	8.65057900	12.22280000	11.63260800
C	2.71432000	7.86201300	14.92307600
H	2.48322100	6.88522500	14.50769400
C	7.45376600	16.27807000	10.65998400
H	8.33998900	16.89423700	10.53288900
C	9.70924000	13.14291700	15.24207300
H	9.99824600	13.38971000	16.25861900
C	4.63326900	5.38967700	16.63133800
H	3.91377000	5.88350100	17.28018900
C	10.27720900	12.39573800	13.02138900
H	11.01913100	12.06311400	12.29992000
C	3.91862900	8.36489700	12.77843300
C	7.40094600	14.61962900	23.94525100
H	7.48899200	14.85448100	25.00228700
C	2.77217100	14.31940600	13.74801800
H	2.16007500	14.31091000	12.85049100
B	3.31885600	11.87395000	16.70367700
C	8.21809400	13.48392300	21.97435700
H	8.95061900	12.82712700	21.51413000
C	6.32295100	16.51428300	9.88526600
H	6.31849100	17.31355700	9.14949100
C	2.28430300	8.17756500	16.20240100
H	1.71901800	7.45583100	16.78571100
C	8.56954900	11.50743600	18.59450300
H	7.65441200	10.92042100	18.54527800
C	9.69184300	13.59827500	19.01321800
H	9.66346600	14.64750000	19.29386800
C	5.19385500	15.71399700	10.06146500
H	4.30415900	15.88702400	9.46168000
C	8.32740400	13.78319000	23.33132700
H	9.14528200	13.35928300	23.90803600
C	4.40834900	4.07871200	16.23058400
H	3.51888100	3.55686700	16.57382100
C	9.79090700	10.91049000	18.28897500
H	9.81781700	9.86630900	17.99454300
C	4.87209000	7.15235600	12.86381900
H	4.39722400	6.31379100	13.38288200
H	5.77869600	7.41930600	13.41566900
H	5.16302500	6.81915900	11.86174800
C	10.96374700	11.65430600	18.35554200
H	11.91735000	11.18925700	18.11935400
C	6.68074500	5.42974500	15.36850700
H	7.57060700	5.94652400	15.02103700
C	10.91246300	13.00120700	18.71600200
H	11.82591300	13.58866500	18.76162500
C	5.31531600	3.43576300	15.38773400
H	5.13719300	2.41218200	15.07019400
C	6.44909200	4.11775500	14.95875000
H	7.16338600	3.62870000	14.30160000
C	8.75667900	7.51451000	16.87455500
H	8.56123700	6.79430300	17.66428800
C	7.69348600	8.19727400	16.27145600
C	7.97141400	9.12878500	15.26684800
H	7.15386400	9.67561600	14.80079100
C	9.28262700	9.35927600	14.85510100
H	9.48004400	10.08987900	14.07707800
C	10.32948800	8.65906200	15.44443300
H	11.35231400	8.83568600	15.12188000
C	10.06502000	7.73836400	16.45888100
H	10.88110800	7.19577300	16.92921200
C	2.67722900	7.98746100	11.93917800
H	2.96174600	7.68650700	10.92675200
H	1.99189000	8.83839400	11.86957200
H	2.14240800	7.14897000	12.39368700
O	1.69541200	12.03778700	16.92771900
H	1.23099200	12.25011800	16.09962100
H	1.49594300	12.75474800	17.55521700

R_menh3

Energy: -4494.88175558 Eh

P	6.21594800	13.18460300	13.14186800
P	6.84303700	13.49727700	19.46599700
P	5.95903700	7.89577400	16.60308400
O	5.30811600	14.20014300	17.02554500
O	4.96496200	9.98216700	18.46050800
O	4.65900900	10.85963000	14.10449400
C	4.41491400	14.28992200	15.96647800
C	5.97492900	15.31095400	17.47535100
C	5.14089600	16.71107700	15.51614500
C	4.35452400	15.45173900	15.18599800
C	5.39015000	8.89808200	19.18577700
C	5.90087300	16.54286400	16.81813100
C	4.33705200	11.04900700	19.08642900
C	3.74216500	12.01109800	18.24518800
C	6.75493400	15.14851700	18.64062100
C	3.62854500	13.14821700	15.70662600
C	4.34539500	11.15805500	20.48361200
C	5.34502300	8.86998000	20.58269300
C	3.80264300	9.98380200	14.75444900
C	5.00618900	10.65448100	12.79364500
C	5.88801700	7.80331300	18.44698800
C	5.76331000	11.67430800	12.17814000
C	7.17186600	14.00888000	21.20680700
C	7.42506500	16.26197000	19.15021800
H	7.99637600	16.16062800	20.06805700
C	6.60366600	17.62153600	17.35872100
H	6.56731700	18.58281200	16.85463900
C	3.33186700	10.38862200	16.01856500
C	6.37715400	14.41839700	11.78105500
C	7.99213000	12.88650700	13.55971300
C	6.29980400	6.66597600	19.14362800
H	6.64997100	5.80212900	18.58660900
C	7.35336000	17.49644300	18.51879800
H	7.88335300	18.35256100	18.92538600
C	3.29725700	13.17474800	18.89313000
H	2.90538500	13.99216400	18.28869800
C	5.78496300	7.71524000	21.23330100
H	5.76922800	7.67994800	22.31868200
C	6.23535600	14.83478300	21.84699600
H	5.41230000	15.25520700	21.27452900
C	4.64325400	9.49819800	12.09713800
C	4.89840500	10.07172100	21.39317800
C	3.84084100	12.32325400	21.05676300
H	3.86952800	12.44636900	22.13554200
C	8.51213500	12.87947100	18.95888800
C	3.80014500	9.64861300	22.39370000
H	4.16342200	8.87789900	23.07975700
H	2.92971300	9.25469100	21.85889300
H	3.47671800	10.50025300	22.99866300
C	6.24917700	6.61369100	20.53019200
H	6.57654700	5.72308600	21.05819600
C	3.55658300	15.43582500	14.04373800
H	3.51692600	16.31345900	13.40477000
C	3.47340400	8.75085500	14.17538600
C	4.16360300	17.90175000	15.63029600
H	3.62183600	18.04977300	14.69206000
H	4.69706600	18.83076600	15.85131400
H	3.43362800	17.72262100	16.42651800
C	6.12112400	11.51725700	10.83826300
H	6.67456000	12.30914200	10.34247400
C	5.83582900	6.10569100	16.17259100
C	3.34932700	13.35028500	20.26712800
H	2.99908600	14.27366500	20.72007900
C	6.16463900	16.98913300	14.39215500
H	6.88159600	16.16556700	14.32155300
H	6.71692200	17.91397200	14.59151100
H	5.66660300	17.08531800	13.42205200
C	5.25367600	14.67605000	10.98073600
H	4.36269200	14.06345900	11.09491000

C	5.03671800	9.38314100	10.76209500
H	4.77364700	8.48673700	10.20802800
C	7.50552300	15.22918900	11.62119000
H	8.38656500	15.05956700	12.23338500
C	2.63792200	9.40616600	16.73957000
H	2.30634600	9.63650200	17.75140000
C	6.35003500	15.12871300	23.19983900
H	5.61879900	15.77929000	23.67246900
C	2.90457700	13.18476000	14.50638900
H	2.36595700	12.29169800	14.20372500
C	10.68247400	12.71296300	14.31589800
H	11.72737200	12.64758500	14.60814700
C	5.76178600	10.38022900	10.12664700
H	6.04989800	10.27067100	9.08540300
C	6.12164000	10.61678500	22.16307600
H	5.85703700	11.50448400	22.74637900
H	6.91371200	10.89756100	21.46206100
H	6.51607300	9.85758800	22.84701600
C	8.39099300	13.23158400	14.85410300
H	7.64672200	13.57375400	15.57072900
C	8.95577600	12.43212700	12.65159100
H	8.66332200	12.15277800	11.64291600
C	2.73754800	7.84421900	14.93494500
H	2.49796200	6.86902600	14.52083100
C	7.51425000	16.26110800	10.68418300
H	8.40257000	16.87797300	10.57645700
C	9.72948900	13.14537300	15.23137400
H	10.02049300	13.41383600	16.24183600
C	4.69445900	5.39844900	16.57913800
H	3.97293800	5.87744000	17.23641300
C	10.29314400	12.35258500	13.02530400
H	11.03406700	12.00591400	12.30934500
C	3.90187300	8.35829600	12.76968900
C	7.39563600	14.59032400	23.95008900
H	7.48381400	14.81700900	25.00890600
C	2.86167200	14.29476100	13.67653500
H	2.28659000	14.27417500	12.75473300
B	3.32619600	11.87542400	16.67177700
C	8.21516300	13.47369900	21.96930100
H	8.94970200	12.82325900	21.50328000
C	6.39813100	16.50047300	9.88933000
H	6.40714100	17.30289800	9.15701800
C	2.34892700	8.15008800	16.23018300
H	1.81038800	7.42092900	16.82940200
C	8.59152600	11.53630400	18.58067500
H	7.68346800	10.93870200	18.52606100
C	9.68757700	13.63860700	19.00992300
H	9.64587200	14.68647200	19.29401300
C	5.26661200	15.69864500	10.04056800
H	4.38835900	15.87348300	9.42461600
C	8.32456400	13.76221200	23.32854200
H	9.14454300	13.33663500	23.90105200
C	4.47735500	4.09211400	16.15910300
H	3.59136100	3.55982500	16.49530400
C	9.82090300	10.95496500	18.27673500
H	9.86101000	9.91236900	17.97811500
C	4.84975700	7.13989500	12.83659300
H	4.37966100	6.30185700	13.36092800
H	5.76612400	7.40100400	13.37485800
H	5.12297800	6.80855600	11.82901000
C	10.98478200	11.71203800	18.35038100
H	11.94461900	11.25910200	18.11583700
C	6.74181800	5.46883900	15.31821800
H	7.62912600	5.99547400	14.97904800
C	10.91617100	13.05718100	18.71459500
H	11.82237000	13.65540300	18.76497200
C	5.38803200	3.46688200	15.30712800
H	5.21621400	2.44692100	14.97480600
C	6.51812800	4.16177000	14.88914700
H	7.23600300	3.68650200	14.22582400
C	8.80037100	7.52883200	16.92311300
H	8.58379000	6.83444600	17.73020400
C	7.75497600	8.20166300	16.27956400
C	8.05942200	9.09899500	15.25177600
H	7.25552800	9.63745200	14.75300200
C	9.38006800	9.30387800	14.85671000
H	9.59814600	10.00658600	14.05884600
C	10.40964800	8.61440600	15.48768000
H	11.43973900	8.77159900	15.17855900
C	10.11824000	7.72911800	16.52589700
H	10.92059900	7.19501700	17.02844500
C	2.64662100	7.99327100	11.94609200
H	2.91537600	7.69606100	10.92825000
H	1.96603000	8.84910600	11.89040300
H	2.11272800	7.15577700	12.40386300
N	1.54811300	12.08157900	16.93817600
H	1.41668200	13.05839300	17.19260800
C	0.50786900	11.71171400	15.93829200
H	-0.46509300	11.61076800	16.43022700
H	0.43990900	12.48794800	15.17606600
H	0.77809600	10.76134500	15.47250000
H	1.37134300	11.56158100	17.79529900

R_menh3_ipr

Energy: -3816.26393554 Eh

P	1.97870300	31.26044200	14.52643100
P	4.49239200	36.60923100	17.11199900
P	6.42898200	32.78466500	12.70320100
O	1.24495300	34.05740100	14.97355900
O	3.68365400	33.12437100	11.42702200
O	4.98468500	36.68994400	14.06660800
C	2.29604000	33.26461200	11.39283600
C	1.17348900	35.42740800	15.08090900
C	4.91494100	36.73365600	12.67649200
C	1.95931800	36.16136400	14.17071900
C	3.80750500	36.12463000	12.05393300
C	1.70304800	34.30409300	12.12426700
C	1.55170300	32.36159900	10.61418600
C	5.75371000	37.61840400	14.73690900
C	0.30181300	34.21600200	12.22757600
H	-0.22135800	34.94557900	12.84429500
C	0.29976600	33.26212800	15.56604600
C	3.86203700	36.07660700	10.64985800
H	3.08258900	35.52991600	10.12801500
C	5.41839900	31.51260400	11.81519300
C	6.75510400	38.33594900	14.06356400
C	4.19455300	31.85722500	11.21388100
C	3.50969400	30.96119500	10.37975600
C	0.35365700	36.02991900	16.04290900
C	5.87550500	37.35852700	10.55797000
H	6.65478600	37.83746300	9.97608400
C	-0.71512800	33.79035000	16.36534100
C	2.07662900	37.53064200	14.45182400
H	2.73184800	38.13982100	13.83012300
C	5.94936800	37.34812900	11.94874300
C	4.84533200	36.70033400	9.90164600
H	4.82770900	36.65307100	8.81603500
C	0.43072400	31.87764000	15.33368900
C	2.31514700	31.50341700	9.62363700
C	5.55647700	37.76758100	16.12310400
C	-0.45757900	33.24137100	11.60146100
H	-1.53427700	33.20852900	11.74276700
C	0.16876600	32.34039500	10.74715200
H	-0.42356600	31.62223900	10.19022700
C	-1.69665600	32.91282300	16.83398000
H	-2.50087800	33.29374100	17.45673300
C	5.87020900	30.19875500	11.63819700
H	6.79812900	29.88735700	12.10523300
C	7.45211900	39.32118800	14.75894600
H	8.21019500	39.90708900	14.25140400
C	-0.70167200	35.24384000	16.81142000
C	1.36532600	38.15312100	15.46747000

H	1.47391500	39.22028800	15.63667000
C	7.17059100	37.87694800	12.67898100
C	3.99299100	29.66353400	10.24171800
H	3.45971000	28.95093300	9.62128000
C	0.47266400	37.40805900	16.22629000
H	-0.13720000	37.90424800	16.97668500
C	7.64586800	33.20962300	11.30749800
H	8.27389900	33.99454000	11.75066100
C	-0.58095700	31.04678100	15.81429600
H	-0.52865500	29.97693800	15.64318800
C	1.57565700	29.45997300	14.18364100
H	1.09600300	28.98668900	15.05015500
C	6.32892600	38.72717200	16.78711400
H	6.22507100	38.84078200	17.86173200
C	2.83855200	32.41881700	8.48446100
H	3.43818500	31.83188900	7.77953900
H	3.46233200	33.22699200	8.87828700
H	1.99285000	32.86333500	7.94756300
C	3.97858200	37.67875800	18.59703400
H	4.85546700	37.79561600	19.25171000
C	-1.65510000	31.55916800	16.53576200
H	-2.43459100	30.89543800	16.89812100
C	3.06468100	31.12074400	16.07033800
H	3.95973200	30.60773500	15.69395300
C	0.63390700	29.38532000	12.97825600
H	-0.28612900	29.95924100	13.13225100
H	0.36013100	28.34332500	12.77074300
H	1.13115900	29.79284900	12.09099900
C	5.16138800	29.27330800	10.88481500
H	5.53045500	28.25701000	10.78125500
C	7.51650000	31.74321000	13.82648000
H	8.01723500	30.95199400	13.25496900
C	7.23176500	39.53478500	16.11359100
H	7.79592700	40.29358200	16.64788600
C	5.95994100	35.68804800	17.89230400
H	6.65376800	36.45487200	18.26885200
C	2.87887900	28.70890900	13.88713300
H	3.46123500	29.22114900	13.11153600
H	2.65281600	27.69955900	13.52162600
H	3.50933300	28.60730400	14.77611200
C	1.45813300	30.40885800	8.99050400
H	0.61635600	30.85436900	8.45223700
H	1.06408100	29.70872700	9.73452600
H	2.04210700	29.84511800	8.25696600
C	8.54997300	32.08405600	10.80934000
H	9.19246500	31.67522200	11.59511900
H	9.20390300	32.46624400	10.01484500
H	7.96002400	31.26454400	10.38444900
B	2.38159400	35.67086500	12.67898300
C	-0.39557900	35.27664400	18.32517800
H	-0.38015600	36.30755100	18.69442500
H	0.57874400	34.82051100	18.52737800
H	-1.15972500	34.72461400	18.88262600
C	6.86578500	33.82665100	10.14738900
H	6.26807700	33.06322000	9.63405100
H	7.55347800	34.26380600	9.41273100
H	6.18755300	34.61206500	10.48828300
C	8.59817400	32.64919300	14.42545900
H	8.15013800	33.49761200	14.95358400
H	9.27328600	33.04662400	13.66057200
H	9.20343300	32.08564400	15.14595700
C	8.15138300	36.69000200	12.86739600
H	8.48129200	36.32786600	11.88708400
H	7.66586400	35.86215400	13.39590500
H	9.02671200	37.01014200	13.44444800
C	2.48295300	30.30907900	17.22558100
H	1.57749800	30.78320800	17.62039900
H	3.21312200	30.24771500	18.04307600
H	2.22862500	29.28548100	16.93207000
C	-2.08199800	35.88879200	16.55733500
H	-2.86904700	35.35971800	17.10230200
H	-2.32437300	35.86660800	15.48978200
H	-2.09152000	36.92940000	16.89341200
C	3.49543000	32.51054800	16.54216000
H	3.87532800	33.12592500	15.71952000
H	4.28641500	32.41443800	17.29558700
H	2.66144200	33.05212700	17.00391400
C	3.43719700	39.06173400	18.22722100
H	4.14737100	39.67098600	17.66416500
H	3.17167200	39.60534400	19.14275300
H	2.53019500	38.95939400	17.62612200
C	2.87945600	36.92527600	19.36039100
H	1.97420000	36.87949400	18.74682900
H	2.63561000	37.45469500	20.29014400
H	3.15359500	35.89934600	19.61733700
C	6.67079700	34.87395300	16.81126900
H	5.99358800	34.14070000	16.35962400
H	7.51840100	34.32946200	17.24592200
H	7.05573900	35.51137000	16.00812500
C	7.89202500	38.97830200	11.89446600
H	8.79139700	39.30101500	12.42452000
H	7.24542700	39.84752000	11.73417600
H	8.22910600	38.60650300	10.92443000
C	6.67086700	31.09582000	14.92322700
H	7.30685100	30.51630300	15.60379900
H	5.91771500	30.42083300	14.50326300
H	6.14921700	31.85742500	15.51343400
C	5.54128300	34.79488500	19.06150900
H	5.22336100	35.37789800	19.93026100
H	6.39056500	34.17414300	19.37332800
H	4.72285800	34.12273000	18.78006900
N	1.28738200	36.89648400	11.82480300
H	1.74529100	37.79506300	11.96265200
H	0.46321400	36.92891300	12.42092600
C	0.84414600	36.80258600	10.40677900
H	0.59802600	35.76443500	10.17153100
H	-0.04144900	37.42717200	10.24837600
H	1.64148200	37.14795700	9.74847600

R_nh3

Energy: -4455.62500396 Eh

P	6.18748700	13.19896700	13.14252600
P	6.83909000	13.49021800	19.48190900
P	5.92649800	7.89189800	16.62348800
O	5.31666000	14.19406700	17.03439400
O	4.95169500	9.98240600	18.48382200
O	4.63323300	10.87473700	14.11020600
C	4.41641400	14.29159100	15.98218900
C	5.98904200	15.30276300	17.48315800
C	5.15476500	16.70824500	15.52749900
C	4.35351700	15.45629000	15.20562100
C	5.38869000	8.90292100	19.20873300
C	5.92204600	16.53412000	16.82428400
C	4.32667900	11.04963400	19.11329500
C	3.72366700	12.00783500	18.27551000
C	6.76649000	15.13844300	18.64959200
C	3.61547500	13.15964300	15.73277200
C	4.34079200	11.15956000	20.51023700
C	5.35714300	8.87879600	20.60616700
C	3.78535100	9.99239400	14.76377000
C	4.98730800	10.66395100	12.80173300
C	5.88371200	7.80767600	18.46864100
C	5.74088100	11.68451600	12.18339400
C	7.17292800	14.00864500	21.21973000
C	7.44515000	16.24804400	19.15615700
H	8.01454500	16.14507500	20.07499900
C	6.63440300	17.60857300	17.36088600
H	6.60456900	18.56882100	16.85448900
C	3.31559600	10.39422000	16.02968800
C	6.33652700	14.42877500	11.77723900
C	7.96550500	12.90615900	13.55817800
C	6.31026100	6.67541600	19.16449200

H	6.65759900	5.81098300	18.60660500
C	7.38434700	17.48081500	18.52037900
H	7.92175700	18.33375600	18.92388700
C	3.26195900	13.16383400	18.92303100
H	2.85024000	13.97127200	18.31827300
C	5.81202200	7.72926300	21.25582500
H	5.80700700	7.69741600	22.34141500
C	6.23451300	14.83423600	21.85757500
H	5.40744300	15.24681900	21.28502300
C	4.63377600	9.50253600	12.10887100
C	4.90445300	10.07735400	21.41816800
C	3.82959200	12.32150400	21.08441100
H	3.86271600	12.44604300	22.16293100
C	8.50201300	12.85745500	18.97303200
C	3.81155600	9.64528500	22.42093200
H	4.18172100	8.87518400	23.10405900
H	2.94207400	9.24716000	21.88770700
H	3.48459200	10.49378100	23.02846300
C	6.27643900	6.62839500	20.55177300
H	6.61542500	5.74195400	21.07946000
C	3.53526000	15.45174400	14.07801600
H	3.49708800	16.32957400	13.43939400
C	3.45909800	8.75855900	14.18494500
C	4.18988100	17.90854600	15.64887300
H	3.64082800	18.06063300	14.71563100
H	4.73456700	18.83254900	15.86307200
H	3.46591600	17.73811500	16.45245700
C	6.10296200	11.52439700	10.84510400
H	6.65235400	12.31780700	10.34726400
C	5.79999700	6.09950100	16.20503700
C	3.32015500	13.34152200	20.29671800
H	2.95903400	14.25978800	20.75166900
C	6.17092900	16.97652000	14.39459400
H	6.87793300	16.14525500	14.31519500
H	6.73513300	17.89477200	14.59079100
H	5.66416000	17.08072500	13.42982700
C	5.20291900	14.68367800	10.99040800
H	4.31236000	14.07326400	11.12025400
C	5.03283000	9.38345300	10.77582400
H	4.77690800	8.48284100	10.22527100
C	7.46335300	15.23776800	11.59949800
H	8.35164200	15.07074900	12.20182500
C	2.60771100	9.41539900	16.74330400
H	2.28014700	9.64292300	17.75716500
C	6.35190600	15.13695800	23.20818000
H	5.61911100	15.78698000	23.67914700
C	2.84535400	13.21712100	14.56144500
H	2.26152500	12.34194300	14.27807500
C	10.65467800	12.72318700	14.31760300
H	11.69890200	12.65380300	14.61132900
C	5.75323700	10.38196400	10.13756400
H	6.04490000	10.26949000	9.09762800
C	6.12501500	10.63097500	22.18591800
H	5.85561100	11.51773100	22.76839800
H	6.91463900	10.91553900	21.48375200
H	6.52517000	9.87555100	22.87071800
C	8.36142000	13.23119400	14.85868100
H	7.61560400	13.56104700	15.57934800
C	8.93200300	12.46756700	12.64512600
H	8.64265100	12.20332600	11.63154700
C	2.71945800	7.85194900	14.94109400
H	2.48359800	6.87607300	14.52653400
C	7.46112800	16.26483600	10.65706000
H	8.34841100	16.88054900	10.53518700
C	9.69907500	13.13966900	15.23771800
H	9.98742500	13.39155800	16.25322800
C	4.66158100	5.39684400	16.62783300
H	3.94622900	5.88233000	17.28725600
C	10.26871200	12.38372900	13.02036100
H	11.01146800	12.04930700	12.30053400
C	3.89277200	8.36307100	12.78171700
C	7.40196800	14.60738100	23.95857600
H	7.49206300	14.84058700	25.01581700
C	2.80519600	14.32629400	13.73076500
H	2.20149300	14.31952500	12.82737800
B	3.31289500	11.88206400	16.70136600
C	8.22056900	13.48232000	21.98213900
H	8.95645800	12.83215700	21.51787200
C	6.33542700	16.50082700	9.87485900
H	6.33598500	17.29940500	9.13830500
C	2.31938400	8.15948100	16.23175500
H	1.77631900	7.43099500	16.82766500
C	8.56950200	11.51377900	18.59443100
H	7.65687100	10.92289400	18.54473600
C	9.68324100	13.60801600	19.01757900
H	9.65067000	14.65624000	19.30146900
C	5.20492500	15.70117400	10.04471600
H	4.31904200	15.87406300	9.43925500
C	8.33250600	13.77935000	23.33943600
H	9.15567400	13.36040000	23.91225400
C	4.43955900	4.08725900	16.22084700
H	3.55611000	3.55862300	16.56927100
C	9.79280100	10.92379300	18.28293100
H	9.82375800	9.88105200	17.98389000
C	4.84158600	7.14587300	12.85511300
H	4.36898000	6.30774100	13.37689800
H	5.75450600	7.40812300	13.39870400
H	5.12121400	6.81385600	11.84952500
C	10.96247300	11.67255600	18.34928200
H	11.91750000	11.21282400	18.10841600
C	6.69746200	5.45483500	15.34764900
H	7.58206000	5.97790400	14.99615800
C	10.90585100	13.01787500	18.71475200
H	11.81659000	13.60960000	18.75973000
C	5.34211100	3.45398700	15.36607300
H	5.16645900	2.43139200	15.04401300
C	6.46885500	4.14428500	14.93182800
H	7.18018500	3.66276900	14.26595500
C	8.77404200	7.52667200	16.88920800
H	8.57284900	6.81892800	17.68864100
C	7.71595800	8.20492200	16.27236600
C	8.00109600	9.11982300	15.25467900
H	7.18736200	9.66226300	14.77676300
C	9.31474300	9.33835400	14.84405200
H	9.51781400	10.05563900	14.05522500
C	10.35671500	8.64354100	15.44823500
H	11.38140500	8.81119800	15.12685900
C	10.08483200	7.73943600	16.47552600
H	10.89701300	7.20096400	16.95720800
C	2.64122000	7.99429400	11.95390500
H	2.91470000	7.69491200	10.93795200
H	1.95986000	8.84912600	11.89291700
H	2.10636500	7.15736000	12.41148500
N	1.58445700	12.08527700	16.87071100
H	1.34152500	13.05945800	17.03539800
H	1.09315300	11.78176400	16.03309600
H	1.22563100	11.54195200	17.65252800

R_nh3_ipr

Energy: -3777.00682298 Eh

P	1.95739300	31.29279700	14.50236800
P	4.49422200	36.60039100	17.11242100
P	6.41574700	32.81195200	12.70169200
O	1.22311800	34.08913900	14.96837800
O	3.67035300	33.15725000	11.43008600
O	4.98767600	36.68462900	14.06511000
C	2.28315800	33.30088100	11.38490500
C	1.14911800	35.45949800	15.08258800
C	4.91321300	36.74460500	12.67573000
C	1.92866900	36.19913600	14.17348200
C	3.79368500	36.16430900	12.05097200
C	1.68644000	34.34052200	12.11294100

C	1.54265300	32.40289900	10.59683500
C	5.76118800	37.60709300	14.74018800
C	0.28200600	34.26496800	12.19199600
H	-0.24302500	34.99174700	12.81081800
C	0.28376800	33.28925200	15.56402100
C	3.82247000	36.17046800	10.64319800
H	3.01846300	35.66859800	10.10657200
C	5.40159500	31.54106800	11.81760100
C	6.76766000	38.32072500	14.07011200
C	4.18014200	31.88980100	11.21398500
C	3.49856800	30.99759000	10.37308200
C	0.32669900	36.05684600	16.04586900
C	5.86370800	37.40814000	10.56297000
H	6.64487000	37.88912200	9.98553100
C	-0.72877100	33.81165200	16.37054300
C	2.02664600	37.57136300	14.44262700
H	2.66660000	38.18432600	13.80855600
C	5.95083800	37.36027900	11.95141900
C	4.80981400	36.79402700	9.90084300
H	4.77344200	36.78583300	8.81490000
C	0.41659100	31.90541200	15.32681400
C	2.31090800	31.54454100	9.61034400
C	5.56352200	37.75501600	16.12601700
C	-0.47503400	33.29427400	11.55586800
H	-1.55356300	33.26679200	11.68362600
C	0.15800800	32.38871200	10.71218300
H	-0.43060400	31.67340500	10.14776500
C	-1.70371300	32.92948500	16.84413400
H	-2.50535000	33.30635500	17.47262900
C	5.85194800	30.22702100	11.63880200
H	6.77865900	29.91329800	12.10659500
C	7.46713500	39.30320100	14.76659800
H	8.22748200	39.88709200	14.26014700
C	-0.72206300	35.26491300	16.81706000
C	1.31238600	38.18974300	15.45894000
H	1.40567600	39.25939600	15.62266500
C	7.18130500	37.85936400	12.68617800
C	3.98029800	29.69958800	10.23274600
H	3.44895300	28.99030900	9.60693300
C	0.43354000	37.43643200	16.22594800
H	-0.17814000	37.92841400	16.97761900
C	7.61377100	33.24422200	11.29227700
H	8.25990200	34.01270200	11.73749400
C	-0.58900400	31.07010200	15.81251700
H	-0.53475700	30.00095800	15.63792400
C	1.55140300	29.49365100	14.15585900
H	1.07789200	29.01732200	15.02405800
C	6.33901800	38.71073500	16.79227500
H	6.23414700	38.82312200	17.86695000
C	2.84497400	32.46150000	8.47731200
H	3.45116000	31.87558800	7.77715300
H	3.46497800	33.26907100	8.87838000
H	2.00433600	32.90670700	7.93309000
C	3.97329000	37.67454500	18.59024900
H	4.84465300	37.79387100	19.25184600
C	-1.65945800	31.57666200	16.54328000
H	-2.43391700	30.90934500	16.90981600
C	3.05971100	31.14495500	16.03356300
H	3.94970500	30.63189000	15.64526600
C	0.60066100	29.42346800	12.95727100
H	-0.31909600	29.99515900	13.12130300
H	0.32662600	28.38209400	12.74699900
H	1.08997100	29.83605700	12.06797200
C	5.14468400	29.30486900	10.88012700
H	5.51253700	28.28830400	10.77494800
C	7.51991300	31.77028800	13.80853300
H	8.01693700	30.98269200	13.22875400
C	7.24537400	39.51625800	16.12116900
H	7.81106400	40.27275900	16.65709600
C	5.95667800	35.67814600	17.90042200
H	6.64738700	36.44466300	18.28326800

C	2.85236200	28.74324200	13.84823900
H	3.43231100	29.26010700	13.07406900
H	2.62363400	27.73669600	13.47666300
H	3.48628800	28.63471700	14.73390400
C	1.45603300	30.45372500	8.96798400
H	0.61951500	30.90241400	8.42426300
H	1.05465800	29.75261100	9.70719000
H	2.04417300	29.89045600	8.23744700
C	8.49557600	32.11552900	10.76279200
H	9.14416300	31.68688600	11.53301100
H	9.14282500	32.50198000	9.96490500
H	7.88948500	31.30965500	10.33457300
B	2.35784900	35.71722700	12.67948200
C	-0.41326700	35.29965900	18.33021800
H	-0.39763500	36.33113000	18.69793200
H	0.56153400	34.84411800	18.53109500
H	-1.17605400	34.74792500	18.88985200
C	6.82346100	33.89093900	10.15539800
H	6.20487000	33.14550900	9.64035100
H	7.50551800	34.32821700	9.41554600
H	6.16395600	34.68178100	10.51974800
C	8.60559300	32.67715900	14.39904900
H	8.16153300	33.52336100	14.93414000
H	9.27238500	33.07780100	13.62852300
H	9.21921700	32.11266800	15.11165700
C	8.13566900	36.65140200	12.87419000
H	8.46679700	36.29096900	11.89382400
H	7.62902400	35.82955900	13.39193300
H	9.01236600	36.94940600	13.46092700
C	2.48900700	30.32986300	17.19183000
H	1.59193900	30.80717000	17.60171700
H	3.22984200	30.25946500	17.99893900
H	2.22416400	29.30954900	16.89615300
C	-2.10676400	35.90179400	16.56607100
H	-2.88986300	35.36594800	17.11004800
H	-2.34986200	35.88124800	15.49866700
H	-2.12304300	36.94129700	16.90528500
C	3.49844700	32.53183800	16.50601000
H	3.87821600	33.14632800	15.68258600
H	4.29202400	32.43072400	17.25605000
H	2.66903400	33.07669500	16.97194700
C	3.43620700	39.05463500	18.20463700
H	4.16332500	39.67054700	17.67136100
H	3.13094400	39.59364300	19.11046900
H	2.55464200	38.94638300	17.56747000
C	2.86666600	36.92533300	19.34652600
H	1.96595300	36.88156000	18.72616700
H	2.61740300	37.45669600	20.27371400
H	3.13625700	35.89881000	19.60665700
C	6.67555000	34.86693800	16.82240600
H	6.00096600	34.13712800	16.36146400
H	7.51847400	34.31896600	17.26178000
H	7.06836200	35.50743200	16.02552700
C	7.93025600	38.94703500	11.90937900
H	8.83309800	39.24844300	12.44621100
H	7.30406300	39.83081900	11.74751500
H	8.26502600	38.57025200	10.94034300
C	6.68873000	31.11590400	14.91238400
H	7.33506600	30.53943700	15.58570700
H	5.93575700	30.43663400	14.49906700
H	6.16790800	31.87308100	15.50901400
C	5.52938700	34.78381200	19.06554600
H	5.20337300	35.36646300	19.93160000
H	6.37677000	34.16410900	19.38446400
H	4.71397100	34.11079500	18.77746100
N	1.34470100	36.86484700	11.79113000
H	1.77537800	37.78646700	11.77305400
H	1.19993000	36.57048500	10.82817000
H	0.43092700	36.96122700	12.22757700

R_tbu
Energy: -4649.58887552 Eh

P	6.22140400	13.16401900	13.10283300
P	6.82518000	13.50691000	19.47501800
P	5.94992600	7.85178000	16.64425500
O	5.32722500	14.18404600	17.01039600
O	4.97216000	9.95585500	18.48062300
O	4.68468300	10.83116700	14.07949500
C	4.44535400	14.26935400	15.94259400
C	6.00675800	15.29453400	17.44367100
C	5.18730400	16.68016000	15.46670600
C	4.38805600	15.42507800	15.15246100
C	5.43975800	8.89825500	19.21878000
C	5.95320500	16.51601100	16.76560900
C	4.34675600	11.02732900	19.10139300
C	3.73887900	11.97560000	18.25773400
C	6.77571300	15.14250000	18.61712000
C	3.65084400	13.13517500	15.69073500
C	4.35976800	11.15343100	20.49694300
C	5.42523500	8.89625200	20.61662600
C	3.84395900	9.94203700	14.73267400
C	5.06611700	10.60505100	12.78123300
C	5.94743600	7.80083200	18.49082100
C	5.81626500	11.62687000	12.16066100
C	7.15137600	14.05194700	21.20580400
C	7.46167800	16.25345300	19.11011300
H	8.02480400	16.15982500	20.03383900
C	6.67284400	17.59226600	17.28893900
H	6.65313400	18.54491700	16.76779900
C	3.35650800	10.34589400	15.98973400
C	6.35417600	14.37562400	11.72006700
C	8.00093000	12.91311900	13.54050800
C	6.41124600	6.69133300	19.19893700
H	6.76915500	5.82519000	18.65045300
C	7.41597700	17.47629700	18.45402700
H	7.95908800	18.33073100	18.84663600
C	3.23982500	13.12252600	18.89144700
H	2.78424700	13.89540900	18.27336100
C	5.91681000	7.76916000	21.27882500
H	5.92467600	7.75528000	22.36478700
C	6.20303900	14.88083100	21.82466200
H	5.37442500	15.27523100	21.24127300
C	4.74114700	9.42837900	12.09983300
C	4.94283700	10.09135900	21.41638500
C	3.82662400	12.31154100	21.05920200
H	3.85567800	12.44616200	22.13666700
C	8.48296100	12.84968500	18.98153600
C	3.85468900	9.64146200	22.41679100
H	4.23895700	8.88257100	23.10485900
H	2.99627700	9.22237000	21.88177200
H	3.50737000	10.48627000	23.01828700
C	6.39967300	6.66831100	20.58721400
H	6.76780300	5.79982600	21.12515700
C	3.57394900	15.41035300	14.02175400
H	3.53947600	16.28261100	13.37526800
C	3.53605800	8.69910000	14.16383400
C	4.22009900	17.87946300	15.58143300
H	3.67250400	18.02679900	14.64648200
H	4.76339500	18.80487900	15.79363700
H	3.49496900	17.71052900	16.38426100
C	6.20517500	11.45162400	10.83194100
H	6.75027200	12.24583700	10.33071100
C	5.84177600	6.05066900	16.26361700
C	3.29273300	13.31279800	20.26284800
H	2.90257200	14.22253600	20.71095000
C	6.20284200	16.94387100	14.33250000
H	6.91188300	16.11379500	14.25879300
H	6.76499400	17.86466400	14.52244900
H	5.69610700	17.04094700	13.36709800
C	5.22066000	14.59067700	10.92107000
H	4.34352900	13.96196300	11.05534100
C	5.16808200	9.29425000	10.77694300
H	4.93503500	8.38190300	10.23558800
C	7.46271900	15.20833500	11.53670600
H	8.34978600	15.07340600	12.14873300
C	2.62457300	9.38132300	16.69667000
H	2.24879600	9.64198200	17.68543500
C	6.31160100	15.20843900	23.17013300
H	5.57106000	15.86024600	23.62627500
C	2.87089000	13.18496600	14.52657900
H	2.26094400	12.31727100	14.27772000
C	10.68021400	12.77194900	14.34488200
H	11.72025500	12.71847900	14.65620700
C	5.88640700	10.29286300	10.13655900
H	6.19931900	10.16837300	9.10418600
C	6.14547700	10.67925500	22.18652100
H	5.85170600	11.56470900	22.75886400
H	6.93301700	10.97542100	21.48691300
H	6.55916100	9.94073300	22.88167400
C	8.36754000	13.22246300	14.85336000
H	7.60256500	13.52354700	15.56649700
C	8.99315200	12.51324200	12.63697500
H	8.72816500	12.26221700	11.61353600
C	2.78738100	7.79709200	14.91700800
H	2.56413300	6.81558500	14.50869500
C	7.44360600	16.21866800	10.57654400
H	8.31702200	16.85315700	10.45076800
C	9.69988300	13.15171100	15.25488600
H	9.96438600	13.39058400	16.27999100
C	4.71341500	5.34865700	16.71424300
H	3.99975900	5.84430300	17.36828400
C	10.32452900	12.44991300	13.03462100
H	11.08666800	12.14473400	12.32214700
C	3.99672000	8.29107100	12.77291000
C	7.36235000	14.70070700	23.93467200
H	7.44536200	14.95306300	24.98810100
C	2.83638700	14.28576600	13.68586400
H	2.22566100	14.27697400	12.78718800
B	3.35209600	11.84518100	16.66533700
C	8.19925000	13.54736700	21.98227400
H	8.94227800	12.89457200	21.53334400
C	6.31864100	16.41466300	9.78236500
H	6.30606100	17.20040300	9.03222600
C	2.35344700	8.11894400	16.19371000
H	1.79144300	7.39826200	16.78152600
C	8.53266000	11.50565200	18.60133600
H	7.61151900	10.92880100	18.54642000
C	9.67474600	13.58311200	19.03228600
H	9.65616500	14.63132500	19.31759300
C	5.20580200	15.59182900	9.95826700
H	4.32048400	15.73380900	9.34399600
C	8.30219000	13.86914600	23.33471200
H	9.12554800	13.46645100	23.91883100
C	4.49841700	4.02825500	16.34002700
H	3.62275800	3.50066500	16.70907500
C	9.74844700	10.89851300	18.29362300
H	9.76603400	9.85575700	17.99338400
C	4.95139500	7.08090600	12.87645100
H	4.47400500	6.24522300	13.39763300
H	5.85232900	7.35461600	13.43424300
H	5.25231600	6.74027300	11.87992100
C	10.92836800	11.63055300	18.36516100
H	11.87752400	11.15754700	18.12677000
C	6.73538700	5.39408700	15.41137900
H	7.61119000	5.91663100	15.03782600
C	10.88987000	12.97581700	18.73314800
H	11.80889700	13.55425800	18.78240500
C	5.39782300	3.38272900	15.49098200
H	5.22763900	2.35151800	15.19447100
C	6.51381300	4.07222500	15.02863800
H	7.22198500	3.58143100	14.36615200
C	8.81034100	7.52311100	16.82138800

H	8.64308200	6.78219900	17.59837800
C	7.72411600	8.19973000	16.25297500
C	7.96695100	9.15708000	15.26390500
H	7.13107200	9.69865000	14.82509900
C	9.26594700	9.42162800	14.83475100
H	9.43562700	10.17266700	14.06993500
C	10.33589400	8.72894300	15.39032600
H	11.34939300	8.93254800	15.05443600
C	10.10661500	7.78044100	16.38764300
H	10.94084300	7.24297100	16.83129800
C	2.76251100	7.90518800	11.92701400
H	3.05616900	7.59785900	10.91899900
H	2.07653000	8.75458000	11.84567600
H	2.22553700	7.06907800	12.38363500
C	1.74767400	12.02913900	16.80017900
N	0.60168700	12.16029800	16.89923600
C	-0.82911900	12.32588300	16.97930600
C	-1.25820100	13.20456400	15.79919100
H	-0.76774300	14.18124100	15.84469800
H	-1.00758700	12.72535700	14.84841600
H	-2.34125000	13.35090400	15.84532500
C	-1.46124400	10.93246900	16.89524800
H	-1.19550700	10.44301600	15.95382100
H	-1.12938700	10.30538300	17.72778200
H	-2.54894500	11.03670200	16.94450500
C	-1.14257900	13.00115600	18.31895500
H	-0.79425900	12.38639600	19.15390900
H	-0.66505400	13.98344700	18.37721000
H	-2.22542100	13.13008400	18.40378400

References

1. Holtkamp, P.; Schwabedissen, J.; Neumann, B.; Stammler, H.-G.; Koptyug, I. V; Zhivonitko, V. V; Mitzel, N. W. A Zwitterionic Phosphonium Stannate(II) via Hydrogen Splitting by a Sn/P Frustrated Lewis-Pair and Reductive Elimination. *Chem.-Eur. J.* **2020**, *26* (72), 17381-17385.

2. Mo, Z.; Kolychev, E. L.; Rit, A.; Campos, J.; Niu, H.; Aldridge, S. Facile Reversibility by Design: Tuning Small Molecule Capture and Activation by Single Component Frustrated Lewis Pairs. *J. Am. Chem. Soc.* **2015**, *137* (38), 12227-12230.

3. Hünig, S.; Kiessel, M. Spezifische Protonenacceptoren Als Hilfsbasen Bei Alkylierungs- Und Dehydrohalogenierungsreaktionen. *Chem. Ber.* **1958**, *91* (2), 380–392.

4. Cosier, J.; Glazer, A. M. A Nitrogen-Gas-Stream Cryostat for General X-ray Diffraction Studies. *J. Appl. Cryst.* **1986**, *19*, 105–107.

5. CrysAlisPRO, Oxford Diffraction/Agilent Technologies UK Ltd, Yarnton, England.

6. Sheldrick, G. M. SHELXT - Integrated space-group and crystal-structure determination. *Acta Crystallogr. A* **2015**, *71*, 3–8.

7. Sheldrick, G. M. Crystal structure refinement with SHELXL. *Acta Crystallogr. C* **2015**, *71*, 3–8.

8. Dolomanov, O. V.; Bourhis, L. J.; Gildea, R. J.; Howard, J. A. K.; Puschmann, H. OLEX2: A complete structure solution, refinement and analysis program. *J Appl. Crystallogr.* **2009**, *42*, 339–341.

9. Neese, F. The ORCA program system. *Wiley Interdiscip. Rev. Comput. Mol. Sci.* **2012**, *2*, 73–78.

10. Neese, F. Software update: the ORCA program system, version 4.0. *Wiley Interdiscip. Rev. Comput. Mol. Sci.* **2018**, *8*, e1327.

11. Neese, F.; Wennmohs, F.; Becker, U.; Riplinger, C. The ORCA quantum chemistry program package. *J. Chem. Phys.* **2020**, *152*, 224108.

12. Furness, J. W.; Kaplan, A. D.; Ning, J.; Perdew, J. P.; Sun, J. Accurate and Numerically Efficient r^2SCAN Meta-Generalized Gradient Approximation. *J. Chem. Phys. Lett.* **2020**, *11*, 8208–8215.

13. Grimme, S.; Hansen, A.; Ehlert, S.; Mewes, J. M. R^2SCAN-3c: A 'swiss army knife' composite electronic-structure method. *J. Chem. Phys.* **2021**, *154*, 64103.

14. Caldeweyher, E.*;* Ehlert, S.; Hansen, A.; Neugebauer, H.; Spicher, S.; Bannwarth, C.; Grimme, S. A generally applicable atomic-charge dependent London dispersion correction. *J. Chem. Phys.* **2019**, *150*, 154122.

15. Kruse, H.; Grimme, S. A geometrical correction for the inter- and intra-molecular basis set superposition error in Hartree-Fock and density functional theory calculations for large systems. *J. Chem. Phys.* **2012**, *136*, 154101.

16. Hanwell, M. D.; Curtis, D. E.; Lonie, D. C.; Vandermeersch T.; Zurek, E.; Hutchinson, G. R. Avogadro: An advanced semantic chemical editor, visualization, and analysis platform. *J. Cheminform.* **2012**, *4*, 1–17.

17. Chai, J.-D.; Head-Gordon, M. Systematic optimization of long-range corrected hybrid density functionals. *J. Chem. Phys.* **2008**, *128*, 084106.

18. Chai, J.-D.; Head-Gordon, M. Long-range corrected hybrid density functionals with damped atom–atom dispersion corrections. *Phys. Chem. Chem. Phys.* **2008**, *10*, 6615–6620.

19. Weigend, F.; Ahlrichs, R. Balanced basis sets of split valence, triple zeta valence and quadruple zeta valence quality for H to Rn: Design and assessment of accuracy. *Phys. Chem. Chem. Phys.* **2005**, *7*, 3297–3305.

20. Weigend, F. Accurate Coulomb-fitting basis sets for H to Rn. *Phys. Chem. Chem. Phys.* **2006**, *8*, 1057–1065.

21. Barone, V.; Cossi, M. Quantum calculation of molecular energies and energy gradients in solution by a conductor solvent model. *J. Phys. Chem. A* **1998**, *102*, 1995–2001.

22. Glendening, E. D.; Badenhoop, J. K.; Reed, A. E.; Carpenter, J. E.; Bohmann, J. A.; Morales, C. M.; Karafiloglou, P.; Landis, C. R.; Weinhold, F. NBO 7.0. Theoretical Chemistry Institute, University of Wisconsin, Madison (2018).

23. Lu, T.; Chen, F. Multiwfn: A multifunctional wavefunction analyzer. *J. Comput. Chem.* **2012**, *33*, 580–592.

24. Glendening, E. D.; Landis, C. R.; Weinhold, F. NBO 7.0: New Vistas in Localized and Delocalized Chemical Bonding Theory. *J. Comput. Chem.* **2019**, *40* (25), 2234–2241.

25. Johnson, E. R.; Keinan, S.; Mori-Sánchez, P.; Contreras-García, J.; Cohen, A. J.; Yang, W. Revealing noncovalent interactions. *J. Am. Chem. Soc.* **2010**, *132*, 6498–6506.

26. Cortés-Guzmán, F.; Bader, R. F. W. Complementarity of QTAIM and MO theory in the study of bonding in donor-acceptor complexes. *Coord. Chem. Rev.* **2005**, *249*, 633–662.

27. Contreras-García, J.; Yang, W.; Johnson, E. R. Analysis of hydrogen-bond interaction potentials from the electron density: Integration of noncovalent interaction regions. *J. Phys. Chem. A* **2011**, *115*, 12983–12990.

28. Zhao, L.; von Hopffgarten, M.; Andrada, D. M.; Frenking, G. Energy decomposition analysis. *Wiley Interdiscip. Rev. Comput. Mol. Sci.* **2018**, *8*, e1345.

29. Lu, T.; Chen, Q. Simple, Efficient, and Universal Energy Decomposition Analysis Method Based on Dispersion-Corrected Density Functional Theory. *J. Phys. Chem. A* **2023**, *127*, 7023–7035.

30. Altun, A.; Neese, F.; Bistoni, G. Effect of Electron Correlation on Intermolecular Interactions: A Pair Natural Orbitals Coupled Cluster Based Local Energy Decomposition Study. *J. Chem. Theory Comput.* **2019**, *15*, 215–228.

31. Mitoraj, M. P.; Michalak, A.; Ziegler, T. A combined charge and energy decomposition scheme for bond analysis. *J. Chem. Theory Comput.* **2009**, *5*, 962–975.

32. Gilli, P.; Pretto, L.; Bertolasi, V.; Gilli, G. Predicting Hydrogen-Bond strengths from Acid-Base molecular properties. the pKa slide rule: Toward the solution of a Long-Lasting problem. *Acc. Chem. Res.* **2009**, *42*, 33–44.

33. Kozuch, S.; Gruzman, D.; Martin, J. M. L. DSD-BLYP: A general purpose double hybrid density functional including spin component scaling and dispersion correction. *J. Phys. Chem. C* **2010**, *114*, 20801–20808.

34. Grimme, S.; Ehrlich, S.; Goerigk, L. Effect of the damping function in dispersion corrected density functional theory. *J. Comput. Chem.* **2011**, *32*, 1456–1465.

35. Erdmann, P.; Leitner, J.; Schwarz, J.; Greb, L. An Extensive Set of Accurate Fluoride Ion Affinities for *p*-Block Element Lewis Acids and Basic Design Principles for Strong Fluoride Ion Acceptors. *ChemPhysChem* **2020**, *21*, 987–994.
